# Charge-transfer regulated visible light driven photocatalytic H_2_ production and CO_2_ reduction in tetrathiafulvalene based coordination polymer gel

**DOI:** 10.1038/s41467-021-27457-4

**Published:** 2021-12-16

**Authors:** Parul Verma, Ashish Singh, Faruk Ahamed Rahimi, Pallavi Sarkar, Sukhendu Nath, Swapan Kumar Pati, Tapas Kumar Maji

**Affiliations:** 1 Molecular Materials Laboratory, Chemistry and Physics of Materials Unit, School of Advanced Materials (SAMat), Jawaharlal Nehru Centre for Advanced Scientific Research, Jakkur, Bangalore, 560 064 India; 2 Theoretical Sciences Unit, School of Advanced Materials (SAMat), Jawaharlal Nehru Centre for Advanced Scientific Research, Jakkur, Bangalore, 560 064 India; 3 Ultrafast Spectroscopy Section, Radiation & Photochemistry Division, Bhabha Atomic Research Centre, Mumbai, 400 085 India

**Keywords:** Energy, Gels and hydrogels, Photocatalysis, Photocatalysis

## Abstract

The much-needed renewable alternatives to fossil fuel can be achieved efficiently and sustainably by converting solar energy to fuels via hydrogen generation from water or CO_2_ reduction. Herein, a soft processable metal-organic hybrid material is developed and studied for photocatalytic activity towards H_2_ production and CO_2_ reduction to CO and CH_4_ under visible light as well as direct sunlight irradiation. A tetrapodal low molecular weight gelator (LMWG) is synthesized by integrating tetrathiafulvalene (TTF) and terpyridine (TPY) derivatives through amide linkages and results in TPY-TTF LMWG. The TPY-TTF LMWG acts as a linker, and self-assembly of this gelator molecules with Zn^II^ ions results in a coordination polymer gel (CPG); Zn-TPY-TTF. The Zn-TPY-TTF CPG shows high photocatalytic activity towards H_2_ production (530 μmol g^−1^h^−1^) and CO_2_ reduction to CO (438 μmol g^−1^h^−1^, selectivity > 99%) regulated by charge-transfer interactions. Furthermore, in situ stabilization of Pt nanoparticles on CPG (Pt@Zn-TPY-TTF) enhances H_2_ evolution (14727 μmol g^−1^h^−1^). Importantly, Pt@Zn-TPY-TTF CPG produces CH_4_ (292 μmol g^−1^h^−1^, selectivity > 97%) as CO_2_ reduction product instead of CO. The real-time CO_2_ reduction reaction is monitored by in situ DRIFT study, and the plausible mechanism is derived computationally.

## Abstract (web summary)

Designing active materials for efficient photocatalysis are of great interests. Herein the authors report coordination polymer gel assembled by low molecular weight gelator as photocatalysts for visible light driven H_2_ production and CO_2_ reduction regulated by charge-transfer interactions.

## Introduction

Artificial photosynthesis, i.e. the conversion of sunlight into fuels, is a green approach and has the potential to solve the global energy crisis. In recent years, a significant amount of research has been carried out to develop artificial systems^1^ for mimicking the sophisticated methodology of nature’s water splitting^2,3^ as well as CO_2_ reduction^4,5^. Natural photosynthesis rely on the occurrence of precise sequences of proteins/enzymes to the several elementary steps of intercomponent light-absorption, charge separation, and migration^6,7^. The synthetic assimilation of these parameters precisely in a spatial organization of molecular components is indeed a challenging task^8,9^. The recent upsurge of converting CO_2_ into fuels like CH_3_OH, CH_4,_ or different chemical feedstock has gained widespread attention^10–12^. The conversion of CO_2_ to hydrocarbon fuels would mitigate not only the effect of CO_2_ concentration in the atmosphere but also reduce the dependency on fossil fuel-based economy. However, the photoreduction of CO_2_ molecules is a complex and challenging process due to the very high dissociation energy of the C=O bond (~750 kJ/mol)^13^. Only a handful of metal^14,15^, metal oxide^16–18^, and chalcogenides^19,20^ based heterogeneous catalysts were reported for photocatalytic CO_2_ reduction to CH_4_, but most of them suffer from a low conversion efficiency and poor selectivity^21,22^. CH_4_ formation is thermodynamically favourable (E^0^ = −0.24 V versus RHE at pH = 7) than CO formation (E^0^ = −0.53 V versus RHE at pH = 7)^23,24^ as the former reaction takes place at a lower potential. Nevertheless, from a kinetic point of view, the eight-electron reduction of CO_2_ to CH_4_ is more difficult, especially under photochemical condition than the two-electron reduction of CO_2_ to CO^25^. To address challenges associated with photochemical H_2_ production and CO_2_ reduction, a novel photocatalytic system needs to be developed by the innovative design of photosensitizer and catalytic moiety^26,27^. Recently, carbon-nitride based photocatalyst for H_2_ evolution and CO_2_ reduction to CO has been reported^28,29^. Moreover, there is a huge lacuna in designing and developing such versatile photocatalyst materials that can reduce both, water and CO_2_ efficiently.

To this end, developing soft hybrid materials, such as coordination polymer gel (CPG), assembled by the low molecular weight gelator (LMWG) based linker and suitable metal ions, could be an excellent design approach in the realm of photocatalysis^30,31^. Such hierarchical soft nanofibrous materials^1,32,33^ can facilitate the facile diffusion of reactants to the active sites and will show efficient electron transfer between different components^34–36^. These artificial hybrid synthetic systems can mimic the intricate functioning of the natural photosystem and can eventually show impressive H_2_ evolution from water^37–40^ or CO_2_ reduction. Extended face-to-face arrays of the donor–acceptor^38^ π-chromophoric systems would be an ideal candidate for light harvesting^41^. These systems will allow greater exciton mobility, which, in turn, leads to charge generation and subsequent electron transfer to the catalyst^36,40^. To this end, tetrathiafulvalene (TTF) moiety is a well-known p-type^42^ semiconductor possessing high electron donation capability with excellent photostability and good charge carrier mobility. Further, the integration of a suitable electron acceptor unit to the TTF moiety could result in a system with excellent charge-transfer characteristics^43^. Thus, designing a TTF containing donor–acceptor^44,45^ based LMWG could be an elegant approach for developing new photocatalyst material. Such systems are likely to show low energy charge transfer in the visible range, which will further reduce the bandgap, and corresponding visible-light photocatalyst can be realized^45^. Coordination driven array of donor–acceptor pairs would further improve photocatalytic performances by enhancing charge transfer to the catalytic centre. Thus, the introduction of a suitable metal-binding moiety like terpyridine on TTF containing LMWG could lead to the formation of CPG and provide an opportunity for further improving the photocatalytic activity^46^.

**Fig. 1:**
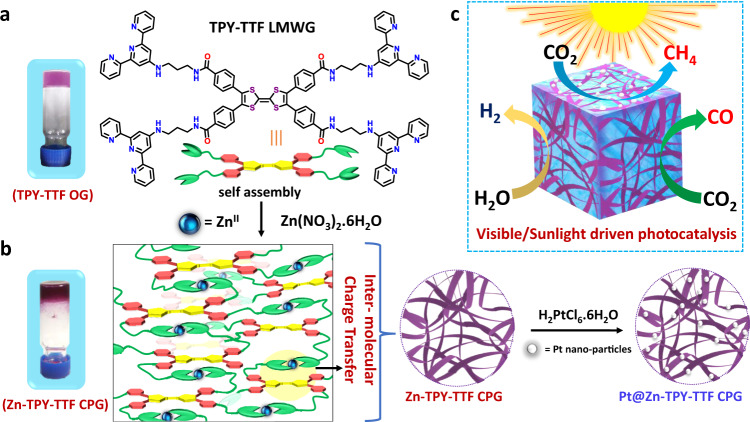
Schematic presentation of visible/sunlight-driven photocatalysis in TTF-based coordination polymer gel (CPG). **a** TPY-TTF low molecular weight gelator (LMWG) based linker and corresponding organogel (TPY-TTF OG). **b** Self-assembly with Zn^II^ toward the preparation of Zn-TPY-TTF CPG and preparation of Pt@Zn-TPY-TTF CPG by in situ stabilization of Pt nanoparticles on the Zn-TPY-TTF CPG. **c** Visible/sunlight-driven photocatalytic activity of Zn-TPY-TTF CPG and Pt@Zn-TPY-TTF CPG towards H_2_ evolution and CO_2_ reduction.

Here, we aim to report an emerging class of materials known as ‘coordination polymer gel’ by integrating Zn^II^ with TTF-based LMWG and explored as a photocatalyst for solar fuel production based on water and CO_2_ reduction. The intermolecular charge transfer is regulated by the innovative design of LMWG, where TTF derivative is connected with metal-binding terpyridine units (TPY) through a flexible alkyl amide chain. The coordination polymer gel (Zn-TPY-TTF CPG) provides a suitable platform for light harvesting as well as a catalytic center for H_2_ production from water (530 μmol g^−1^h^−1^) and CO_2_ reduction to CO (438 μmol g^−1^ h^−1^) with 99% selectivity (Fig. 1). Furthermore, Zn-TPY-TTF CPG with nanoribbon morphology is conjugated with Pt co-catalyst, and the Pt@Zn-TPY-TTF CPG shows many folds enhanced photocatalytic activity towards H_2_ production (14727 μmol g^−1^h^−1^). Interestingly, this Pt@Zn-TPY-TTF CPG produces CH_4_ (292 μmol g^−1^h^−1^) as a photoproduct with high selectivity (97%) and impressive quantum efficiency. Importantly, both Zn-TPY-TTF CPG and Pt@Zn-TPY-TTF CPG shows the potential to perform sunlight-driven photocatalytic activity under ambient conditions. The in situ DRIFT study and DFT calculations help to elucidate the plausible mechanism of charge transfer regulated CO_2_ reduction to CO/CH_4_ formation using CPG catalysts under visible light as well as direct sunlight irradiation.

## Results

### Preparation and characterizations of TPY-TTF OG and Zn-TPY-TTF CPG

**Fig. 2:**
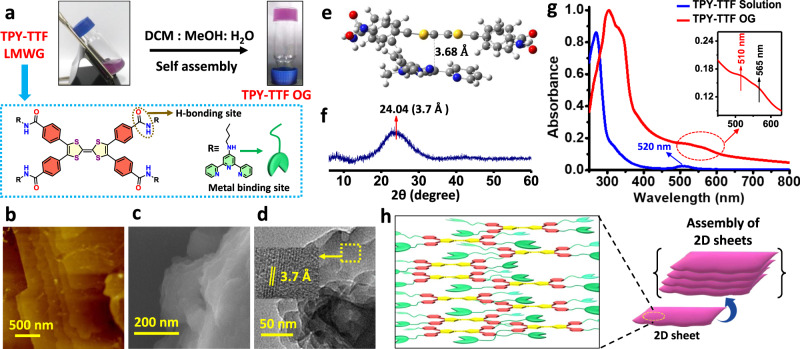
Preparation and characterizations of TPY-TTF OG. **a** Photograph for organogel formation. Morphological analysis for xerogel. **b** AFM image. **c** FE-SEM image. **d** HR-TEM image (inset: showing lattice fringes). **e** Interplanar spacing in the optimized structure of TTF---TPY stacked model obtained through DFT calculation. **f** PXRD pattern for xerogel. **g** Comparison of absorption spectra of TPY-TTF LMWG in solution (8 × 10^−6^ M) with TPY-TTF OG xerogel state. **h** Pictorial representation for self-assembly in TPY-TTF OG forming layered sheet-like morphology.

The TPY-TTF LMWG was synthesized by the amide coupling reaction between 2,2′:6′,2″-terpyridin-4′-yl-propane-1,3-diamine (TPY-NH_2_)^47^ and 1,3,6,8-tetrakis (benzoic acid) tetrathiafulvalene (TTF(COOH)_4_)^48^ (details are provided in method section and supplementary information (SI)), Supplementary Figs. 1–2). The newly synthesized TPY-TTF LMWG was characterized by ^1^H and ^13^C Nuclear Magnetic Resonance (NMR), Fourier Transform Infrared (FT-IR) Spectroscopy, and Matrix Assisted Laser Desorption/Ionization -Time of Flight (MALDI-TOF) mass spectrometric analysis (Supplementary Figs. 3–6). UV-vis absorption study was performed for a well-characterized TPY-TTF LMWG in methanol (10^−6^ M) and showed distinguished absorption bands at 270 nm and 320 nm corresponding to π→π* transition for TPY unit and TTF core, respectively (Fig. 2g). Notably, a low energy absorption band appeared at 520 nm that can be ascribed to intramolecular charge-transfer (CT) interaction between TTF core and benzo-amide moiety^48^. The CT property of TPY-TTF has also been supported by time dependant-density functional theoretical (TD-DFT) computation where the highest occupied molecular orbital (HOMO) and lowest unoccupied molecular orbital (LUMO) are centered in TTF and benzo-amide groups, respectively (Supplementary Fig. 7).

We have examined the gelation propensity of TPY-TTF LMWG in several solvent compositions (Supplementary Table 1). The purple-coloured opaque gel of TPY-TTF organogel (OG) was obtained in the solvent mixture (methanol (MeOH), dichloromethane (DCM) and water (H_2_O) in 2:1:1 ratio) upon heating at 60 °C followed by cooling to room temperature as shown in Fig. 2a and characterization of gel was performed by different techniques (Supplementary Figs. 8–11). The strain-sweep rheology experiments for TPY-TTF OG at 25 °C showed the values of storage modulus (Gʹ) and loss modulus (Gʺ) move constantly in the linear viscoelastic (LVE) region, consisting of the larger Gʹ value under less strain range as compared to the Gʺ, indicating the stable viscoelastic nature, which is a characteristic feature of a gel material (Supplementary Fig. 8). TPY-TTF OG was dried under vacuum at 80 °C to prepare the xerogel and studied the properties of the material (Supplementary Figs. 9–11). Morphology of the TPY-TTF OG xerogel was recorded by the Atomic Force Microscopy (AFM) and Field Emission Scanning Electron Microscopy (FE-SEM) that showed micron size staked layered type of morphology (Fig. 2b, c). We have also prepared aerogel using a critical point dryer (CPD) that also showed similar layered type morphology (Supplementary Fig. 11b). Transmission Electron Microscopy (TEM) images have further confirmed such morphologies (Fig. 2d). The distance between the layers in the stacked morphology was found to be 3.4 ± 0.4 nm based on AFM measurement. The high-resolution TEM analysis showed that the lattice fringes at ~3.7 Å (2d: inset) for the layered morphology suggesting the self-assembly in TPY-TTF OG is driven by the intermolecular π–π interactions. This was also supported by the powder X-ray diffraction (PXRD) study of the xerogel that exhibited a peak at 2θ = 24° with a d-spacing of 3.7 Å (Fig. 2f). Further, we have performed DFT calculations which also support that the self-assembly is driven by intermolecular π–π stacking interactions between TTF---TPY units at a distance of 3.68 Å (Fig. 2e) and TTF---TTF units at a distance of 4.02 Å (Supplementary Fig. 12)^49,50^. The UV-vis absorption study for TPY-TTF OG xerogel displayed slightly red-shifted absorption as compared to the methanolic solution of TPY-TTF LWMG (Fig. 2g). It showed absorption bands at 300 nm and 330 nm, which can be assigned for π→π* transitions of TPY and TTF units of LMWG, respectively. Notably, a broad absorption between 480 and 615 nm was also observed, indicating the existence of charge transfer (CT) in TPY-TTF OG in the xerogel state. The closer analysis of the CT band showed that it consists of two distinguishable adjacent bands with absorption maxima at 510 and 565 nm (Fig. 2g; inset). Further, in-depth analysis through TD-DFT calculations showed CT absorption band at 498 nm and 564 nm, displaying quite fair agreement with the experimental results. The theoretical absorption at 498 nm was consist of both intramolecular (TTF to PhCONH-) and intermolecular (TTF to TPY) CT transitions (Fig. 4a, Supplementary Table 2). Whereas absorbance at 564 nm was attributed to the intramolecular (TTF-PhCONH-) CT transition. Based on the experimental and theoretical observations, the supramolecular assembly of TPY-TTF OG is represented in Fig. 2h, which showed that for both, TTF---TPY as well as TTF---TTF stackings are feasible. The optical bandgap for TPY-TTF OG, calculated by the Kubelka-Munk plot derived from UV-Vis diffuse reflectance spectrometry, was found to be 2.26 eV (Supplementary Fig. 13).

**Fig. 3:**
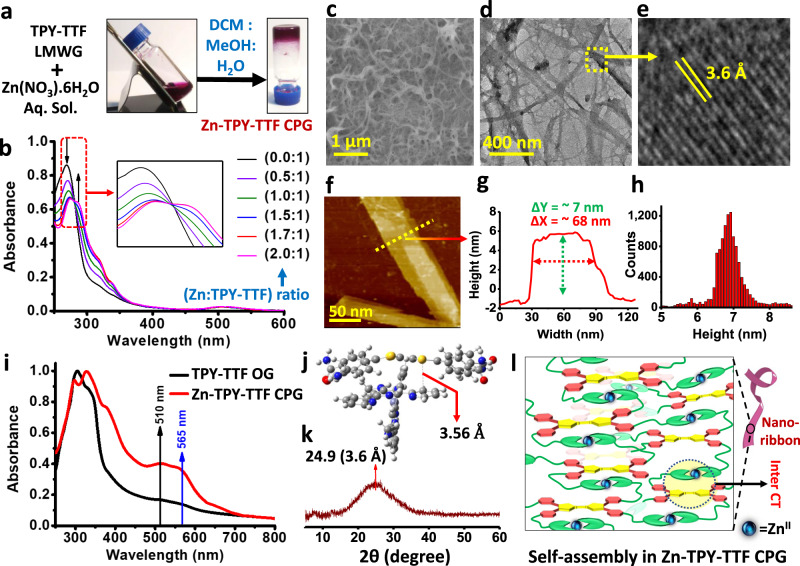
Preparation and characterizations of Zn-TPY-TTF CPG. **a** Photograph for Zn-TPY-TTF CPG formation. **b** UV-visible titration of TPY-TTF LMWG with Zn^II^ ion. **c** FE-SEM image of Zn-TPY-TTF CPG xerogel. **d** HR-TEM image of Zn-TPY-TTF CPG xerogel. **e** Lattice fringes of the selected region. **f** AFM image of Zn-TPY-TTF CPG xerogel and corresponding, **g** height profile and **h** height histogram. **i** Comparison of absorption spectra of TPY-TTF OG and Zn-TPY-TTF CPG. **j** Interplanar spacing in the optimized structure of TTF---[Zn(TPY)_2_]^2+^ stacked model obtained through DFT calculation. **k** PXRD of Zn-TPY-TTF CPG xerogel. **l** Schematic representation for self-assembly of Zn-TPY-TTF CPG.

**Fig. 4:**
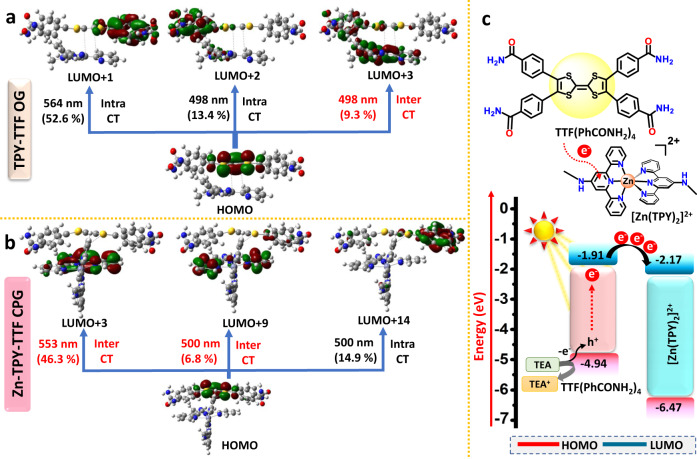
DFT calculations for CT interaction and band alignments in TPY-TTF OG and Zn-TPY-TTF CPG. **a**, **b** HOMO-LUMO charge-transfer transitions with corresponding contributions from intramolecular and intermolecular charge transfer (CT) for TPY-TTF OG and Zn-TPY-TTF CPG, respectively. **c** HOMO-LUMO band alignments of TTF(PhCONH_2_)_4_ and [Zn(TPY)_2_]^2+^ for thermodynamic feasibility of electron transfer in the aqueous medium.

Next, the presence of four terpyridine units in the TPY-TTF gelator has prompted us to investigate further their metal-binding ability to develop coordination polymer gel (CPG) for widening their applications. To this end, we have chosen Zn^II^ as a metal node for binding with TPY as such self-assembly is well explored due to soft acid-base interaction^46,51^. We have performed titration of TPY-TTF (8 × 10^−6^ M in MeOH) with a methanolic solution of Zn(NO_3_)_2_.6H_2_O (8 × 10^−4^ M), and corresponding UV-vis absorption spectra were recorded (Fig. 3b). Notably, the presence of isosbestic point in the Zn^II^ titration suggested the complex formation between Zn^II^ and TPY-TTF LMWG (Supplementary Fig. 14a, b)^52^. The UV-vis titration study illustrated the binding of Zn(NO_3_)_2_ and TPY-TTF in the ratio of 2:1^47^. The association constant (K_a_) of the Zn^II^ ion with TPY-TTF was calculated to be 2.8 × 10^4^ by the Benesi-Hildebrand plot (Supplementary Fig. 14c). Next, Zn(NO_3_)_2_ and TPY-TTF gelator was taken in a molar ratio of 2:1 in the solvent mixture of MeOH, DCM and H_2_O in 2:1:1 ratio, respectively. Heating the reaction mixture to 60 °C followed by cooling to room temperature has afforded a deep purple-coloured coordination polymer gel (Zn-TPY-TTF CPG) (Fig. 3a). Similar to OG, strain-sweep rheology experiments were performed for Zn-TPY-TTF CPG at 25 °C (Supplementary Fig. 15). The value of Gʹ and Gʺ upto 0.01% was found to be ~10 times larger for the Zn-TPY-TTF CPG than the OG, illustrating higher stability of the former one, which is possibly attained due to coordination of the Zn^II^ ion^53^. More importantly, in both cases, tan *δ* (=Gʺ/Gʹ) value was found to be lower than one before reaching yield strain point, which is the intrinsic property of the gel phase (Supplementary Fig. 16). Next, the morphology of the Zn-TPY-TTF CPG was evaluated by FE-SEM upon drying the sample at 80 °C under vacuum, which showed the 3D entangled nanofibrous morphology (Fig. 3c). The aerogel of CPG was also prepared by the CPD method, which showed a similar morphology suggesting that the fibrous morphology of Zn-TPY-TTF CPG is driven through metal coordination with LMWG (Supplementary Fig. 17). TEM studies revealed that nanoribbons form 3D interconnected fibrous morphology (Fig. 3d). The AFM images of Zn-TPY-TTF CPG revealed the height of nanoribbon is ~7 nm and the diameter is in the range of 40–80 nm (Fig. 3f–h). The elemental mapping of the xerogel exhibited the uniform distribution of Zn^II^ in a 3D network of the CPG (Supplementary Fig. 18). EDAX and elemental analyses also correlated the 2:1 ratio of Zn^II^: TPY-TTF in the CPG (Supplementary Fig. 19). The high-resolution TEM analysis exhibited ordering in the nanoribbon, and lattice fringes were observed with a distance of 3.6 Å, which could be attributed to the intermolecular π–π stacking between the TTF---TPY units from the [Zn(TPY)_2_]^2+^ (Fig. 3e). Further, the PXRD pattern for Zn-TPY-TTF CPG xerogel showed a peak at 2*θ* = 24.9° (3.6 Å), which was also observed in the gel state, justifying the presence of π–π stacking (Fig. 3k, Supplementary Fig. 20). In order to evaluate the nature of stacking, TD-DFT calculations were performed, which showed that the packing of TTF with [Zn(TPY)_2_]^2+^ on top of each other was stabilized with a distance of 3.56 Å (Fig. 3j, l) which is in good agreement with the experimental observations^50,54^. On the contrary, stacking through TTF---TTF units on the top of each other was optimized, which revealed the TTF---TTF distance >11 Å due to steric repulsion among the [Zn(TPY)_2_]^2+^ units attached to TTF core, and therefore, the possibility of TTF---TTF stacking was ruled out (Supplementary Fig. 22a). Further, the UV-vis absorption spectrum of Zn-TPY-TTF CPG in xerogel state was found to be similar to the TPY-TTF OG with an enhanced absorption in the visible region as shown in Fig. 3i. To analyse the reason behind enhanced absorption in the visible range, TD-DFT calculations were performed for the stacked model of Zn-TPY-TTF CPG (Fig. 4b). The result showed that the band observed at 510 nm was similar to TPY-TTF OG. Whereas the experimental band at 565 nm is mainly attributed to the theoretical band at 553 nm (Fig. 4b, Supplementary Fig. 22b). Importantly, the transition at 553 nm was observed due to the significant contribution of intermolecular CT from TTF core to [Zn(TPY)_2_]^2+^ unit as shown in Supplementary Table 2^55^. The dominated intermolecular CT transition in Zn-TPY-TTF CPG as compared to TTF-TPY OG is mainly triggered by the planarization of terpyridine ligand, which occurred after the complexation with Zn^II^ ion in CPG^56^. Additionally, Zn^II^ complexation also increased the electron-accepting tendency of terpyridine ligand from the TTF moiety, which further facilitated the intermolecular CT transition^57^. The optical bandgap for Zn-TPY-TTF CPG was calculated to be 2.27 eV, which is closer to the bandgap of TPY-TTF OG (Supplementary Fig. 13). Next, as a controlled study, we found energies of the LUMO for TTF(PhCONH_2_)_4_ and [Zn(TPY)_2_]^2+^ are −1.91 and −2.17 eV, respectively, in the aqueous medium (Fig. 4c), which indicates that excited-state electron transfer is energetically favourable from TTF(PhCONH_2_)_4_ core to [Zn(TPY)_2_]^2+^ centre.

**Fig. 5:**
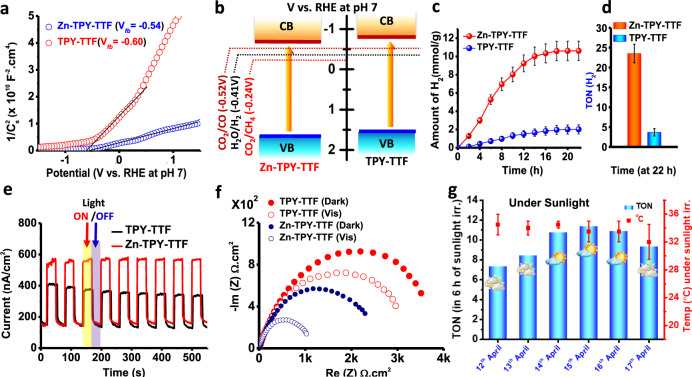
Electrochemical characterization and photocatalytic H_2_ production performances for TPY-TTF OG and Zn-TPY-TTF CPG. **a** Mott-Schottky plot w.r.t. RHE at pH = 7 (at 1000 Hz, −1.5 to +1.5 V). **b** Band alignment based on Mott-Schottky plot (V vs RHE at pH = 7; valence band (VB) and conduction band (CB)). **c** Amount of H_2_ evolution under visible light and **d** corresponding TON (for H_2_) at 22 h. (Error bars in panels **c** and **d** represents the standard deviations of three catalytic runs). **e** Photocurrent measurement in 0.5 M Na_2_SO_4_ at +0.8 V, pH ~7 upon visible-light irradiation in the time interval of 30 sec. **f** Nyquist plot under light and dark condition. **g** TON (for H_2_) for Zn-TPY-TTF CPG under direct sunlight irradiation from 12th to 17th April 2019 (10:00 am to 4:00 pm) and the error bars represents the temperature range of photocatalytic set-up during catalysis under the sunlight irradiation. (Note: all visible-light experiments were performed under 300 W Xenon lamp using visible bandpass filter; 400–750 nm).

Mott-Schottky analysis was performed for the xerogel of both TPY-TTF OG and Zn-TPY-TTF CPG to evaluate experimental feasibility for water and CO_2_ reduction (see SI for details). The M-S plots exhibited n-type nature with a positive slope for both TPY-TTF OG and Zn-TPY-TTF CPG (Fig. 5a). The flat band potentials (*V*_fb_) were found to be −0.60 and −0.54 V versus RHE (at pH = 7) for TPY-TTF OG and Zn-TPY-TTF CPG, respectively. Based on the bandgaps obtained using UV-vis diffuse reflectance spectrometry (Supplementary Fig. 13), the electronic band structures versus RHE at pH 7 could be elucidated and are displayed in Fig. 5b^58^. Interestingly, the band alignments are shown in Fig. 5b illustrate that both TPY-TTF OG and Zn-TPY-TTF CPG possess suitable band edge positions to perform water and CO_2_ reduction under visible-light irradiation.

### H_2_ production activity of OG and CPG under laboratory condition

We have examined the potential of Zn-TPY-TTF CPG for photocatalytic H_2_ production from water under visible-light (400–750 nm) irradiation using 300 W xenon lamp as the light source (Supplementary Figs. 23–25). The photocatalytic H_2_ production activity of Zn-TPY-TTF CPG was screened with different sacrificial electron donors, and the best activity was found with triethylamine (TEA) as a sacrificial agent (Supplementary Fig. 26). Photocatalytic activity of Zn-TPY-TTF CPG was examined in both gel and xerogel state, and similar H_2_ evolution was observed for both under similar conditions (Supplementary Fig. 27a). However, catalytic activities in different conditions were performed using the xerogel because of the ease of handling the catalyst in comparison to the gel state. After optimizing the catalyst loading (Supplementary Fig. 28), 1 mg of Zn-TPY-TTF CPG in xerogel state was dispersed in 38 ml of water for the photocatalytic H_2_ production, and 2 ml triethylamine (TEA) was added into it, which acted as a sacrificial electron donor. The photocatalytic activity of Zn-TPY-TTF CPG was monitored by gas chromatography (GC) analysis and showed 10.60 mmol g^−1^ of H_2_ evolution in 20 h (activity = 530 μmol g^−1^ h^−1^) upon visible-light irradiation as shown in Fig. 5c. The amount of H_2_ evolved was reached saturation in 20 h, and the turnover number (TON) was calculated to be 23.5, as shown in Fig. 5d. The activity is higher than many other transition metal-based photocatalysts (Supplementary Tables 6–8). The apparent quantum efficiency (AQE) for H_2_ production using Zn-TPY-TTF CPG catalyst was calculated to be 0.76% at 550 ± 10 nm. Further, the absence of H_2_ formation with Zn-TPY-TTF CPG under dark condition (absence of light) confirming light is an essential component for catalysis. Next, we have also performed the recyclability test by recollecting the catalyst followed by reusing the photocatalysis for four additional cycles of 6 h each time (Supplementary Fig. 27b). Interestingly, the amount of H_2_ evolution was found to be similar in every cycle (>99%). We also performed ICP analysis for as-synthesized Zn-TPY-TTF CPG sample as well as recovered sample after photocatalysis. In both cases, the amount of Zn content was calculated to be 5.9 (±0.2) wt%. Next, recycled catalyst (Zn-TPY-TTF) was collected at the end of the fourth cycle and analysed by FE-SEM and TEM studies and suggested no significant change in the structure and morphology after the catalytic reaction indicating high stability of the catalyst (Supplementary Fig. 27c, d).

Next, we have examined photocatalytic activity for the OG to compare the importance of morphology, i.e. the spatial arrangement of the chromophore and also the role of metal directed assembly in CPG. Experimental conditions employed for the TPY-TTF OG was similar to the CPG. Interestingly, the H_2_ evolution by the TPY-TTF OG upon visible-light irradiation was increased with irradiation time and reached saturation in 22 h (Fig. 5c). The maximum H_2_ evolution in 20 h was calculated to be 2 mmol g^−1^ (activity = 100 μmol g^−1^ h^−1^), which is albeit lesser than the CPG but higher than most of the reported metal-free photocatalysts for H_2_ evolution^59^. To understand the significant difference in photocatalytic activities between TPY-TTF OG and Zn-TPY-TTF CPG, photocurrent measurements were performed for both (Fig. 5e) in the presence and absence of light. The photocurrent for Zn-TPY-TTF CPG in the presence of light was found to be double as compared to TPY-TTF OG. This indicates the facile charge separation in Zn-TPY-TTF CPG under light irradiation; therefore, expected to show better photocatalytic activity as compared to TPY-TTF OG. This argument has been further validated by the EIS measurement, where the charge-transfer resistance for Zn-TPY-TTF CPG was observed to be significantly lesser as compared to TPY-TTF OG under both dark and light irradiated conditions (Fig. 5f). This can be attributed to the nanoribbon morphology of Zn-TPY-TTF CPG that provides a continuous charge-transfer pathway (via co-facial intermolecular charge delocalization) for the photogenerated electrons, which ultimately enhances the photocatalytic activity.

We have also performed the photocatalytic study with individual structural units of Zn-TPY-TTF CPG to evaluate the importance of coordination driven assembly of CPG in catalysis. Notably, individual components, such as TEA, TTF(COOH)_4_, TPY-NH_2_, and [Zn(TPY-NH_2_)_2_]^2+^ as well as the physical mixture of TTF(COOH)_4_ and [Zn(TPY-NH_2_)_2_]^2+^ were found to be not efficient in catalysing H_2_ evolution reaction (Supplementary Fig. 29). Next, the photocatalytic study has also been performed by making a blend of Zn^II^ salt with TPY-TTF (in 2:1 ratio) (details are given in SI). This showed aggregated spherical morphology (sphere diameter 80 ± 10 nm) as confirmed by FE-SEM study (Supplementary Fig. 30), and the corresponding H_2_ evolution within 6 h was found to be three times lesser as compared to the CPG (Supplementary Fig. 29). This signifies the impact of nano-structuring in photocatalytic performances. To validate the role of intermolecular CT interaction, we have also synthesized a coordination polymer of Zn^II^ (Zn-CP) with TTF(COOH)_4_ and characterized by FE-SEM, EDAX, elemental mapping, TGA, PXRD and UV-vis absorption study (Supplementary Figs. 31–34). Zn-CP showed micron-sized spherical particles with a diameter in the range of 2–3 µm. The Zn-CP showed 0.8 mmol g^−1^ of H_2_ production from water in 12 h which is eight times lesser in activity in comparison to Zn-TPY-TTF CPG photocatalyst under a similar condition (Supplementary Fig. 29). Overall, control experiments have unambiguously indicated that the coordination driven spatial arrangement of donor–acceptor chromophores and corresponding CT interaction have high significance in visible-light photocatalytic performances of CPG (Zn-TPY-TTF).

### Preparation and characterizations of Pt@Zn-TPY-TTF CPG

**Fig. 6:**
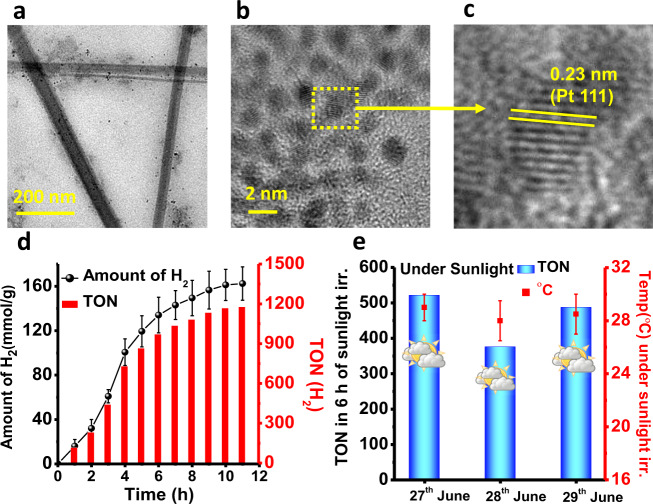
Characterizations and photocatalytic H_2_ production activity of Pt@Zn-TPY-TTF CPG. **a**, **b** HR-TEM images of Pt@Zn-TPY-TTF CPG. **c** Lattice fringes of the selected region. **d** Amount and TON (for H_2_) under visible-light irradiation and error bars represents the standard deviations of three catalytic runs. **e** TON (for H_2_) under direct sunlight irradiation from 27th June to 29th June 2019 (10:00 am to 4:00 pm) and the error bars represents the temperature range of photocatalytic set-up during catalysis under the sunlight irradiation.

Further, we envisioned that the entangled hierarchical fibrous structure of the coordination polymer gels could easily immobilize co-catalyst like Pt on the surface^60^, which would facilitate the separation of photogenerated charge carriers by decreasing diffusion length and eventually enhance the photocatalytic activity^61^. Thus, we have successfully executed in situ generation and stabilization of platinum nanoparticles (Pt NPs) in the self-assembled interconnected network of Zn-TPY-TTF CPG (Pt@Zn-TPY-TTF) (Fig. 6a). High-resolution TEM and FE-SEM analysis have confirmed the stabilization of Pt NPs in self-assembled networks of Pt@Zn-TPY-TTF CPG within the size range of 2–3 nm (Fig. 6b, Supplementary Fig. 36a). Lattice fringes were observed for Pt NPs with the d-spacing value of 0.23 nm, indicating the presence of Pt (111) planes (Fig. 6c). Inductively coupled plasma-optical emission spectroscopy (ICP-OES) measurement indicated the presence of ~2.7 wt% Pt in Pt@Zn-TPY-TTF CPG, which is also supported by EDAX analysis (Supplementary Fig. 36b). The elemental mapping was ensured for the uniform distribution of Pt NPs in the gel matrix (Supplementary Fig. 37). Further, the Mott-Schottky analysis for Pt@Zn-TPY-TTF CPG also revealed that the conduction band edge occurs at −0.51 V vs RHE at pH 7, which is lesser compared to the Zn-TPY-TTF CPG (−0.54 V) catalyst (Supplementary Fig. 38a).

### H_2_ production activity of Pt@Zn-TPY-TTF CPG

Next, photocatalytic activity towards water reduction was examined for Pt@Zn-TPY-TTF CPG under a similar condition as employed for CPG as well as OG. Interestingly, Pt@Zn-TPY-TTF CPG has shown remarkably enhanced catalytic activity under visible-light irradiation, and the amount of H_2_ was calculated to be 162.42 mmol g^−1^ in only 11 h (average activity = 14727 μmol g^−1^ h^−1^), and the corresponding TON value was found to be 1176.9 (w.r.t. 2.7 wt% of Pt, Supplementary Table 3) as shown in Fig. 6d. We have also investigated photocatalytic H_2_ production activity by varying loading amount of Pt NPs, which displayed the highest catalytic activity in the presence of 2.7 wt% of Pt to the CPG (Supplementary Fig. 35). Drastically increased H_2_ evolution after Pt NPs stabilization could be ascribed to the efficient charge separation in Pt@Zn-TPY-TTF CPG as Pt centre is a well-known electron acceptor that accumulates a pool of electrons and subsequently exhibits efficient water reduction. Here, the photoexcited electrons from Zn-TPY-TTF CPG using the harvested light energy transferred to Pt NPs surface to reduce water into H_2_. Furthermore, the recyclability and reusability of Pt@Zn-TPY-TTF CPG towards photocatalytic hydrogen evolution were evaluated upto four cycles similar to the Zn-TPY-TTF CPG (Supplementary Fig. 39). Photocatalytic activity of the recycled catalyst was found to be retained >95% after 4th cycle. The formation of the Schottky junction in Pt@Zn-TPY-TTF CPG was helpful to separate the photogenerated electron-hole pairs. This argument was further validated by the EIS measurement, where the charge-transfer resistance for Pt@Zn-TPY-TTF CPG was found to be almost half as compared to Zn-TPY-TTF CPG under both dark and visible-light irradiation (Supplementary Fig. 38b). Furthermore, approximately four-folds higher photocurrent was observed for Pt@Zn-TPY-TTF CPG as compared to Zn-TPY-TTF CPG (Supplementary Fig. 38c), which corroborated the facile electron transfer from the Zn-TPY-TTF CPG to the Pt centre. Further, the photoluminescence (PL) spectra of Zn-TPY-TTF CPG showed weak emission with a maximum at 581 nm due to the intermolecular charge-transfer interaction (λ_ex_ = 510 nm). (Supplementary Fig. 40). PL spectra for Pt@Zn-TPY-TTF CPG upon excitation at 510 nm showed significantly quenched emission compared to Zn-TPY-TTF CPG. Therefore, to gain more insight into the advantages of Pt NPs in the increased charge separation, the time-resolved photoluminescence (TRPL) decay was studied on the Zn-TPY-TTF CPG and Pt@Zn-TPY-TTF CPG, as shown in Supplementary Fig. 41 (Supplementary Table 4). A higher average lifetime Zn-TPY-TTF CPG system (1.95 ns) compared to Pt@Zn-TPY-TTF CPG system (0.22 ns) obtained by the TRPL studies confirms that migration of photoexcited electrons is much faster in Pt@Zn-TPY-TTF compared to Zn-TPY-TTF CPG^62^. The decrease in the lifetime is attributed to the enhanced separation of photogenerated electrons in Pt@Zn-TPY-TTF CPG, which play decisive roles in enhancing the efficiency of photocatalytic processes. To understand the charge-separation dynamics, transient absorption (TA) experiments were performed with Zn-TPY-TTF CPG and Pt@Zn-TPY-TTF CPG. The TA spectra of Zn-TPY-TTF CPG (Supplementary Fig. 42a) showed a broad excited-state absorption (ESA) band covering 450–700 nm region at early time delay. Such a broad ESA band is due to TTF cation and TPY anion radical formed by the electron transfer process by the photoexcitation. TTF cation has an absorption band at ~440 nm with a long tail extending upto 700 nm^63^, and Zn-TPY anion radical complex has a broad absorption band covering the range of 500–600 nm^64^. Thus, the appearance of a broad band at early time delay suggested that the electron transfer from TTF to Zn-TPY is very efficient in Zn-TPY-TTF CPG and takes place within the time resolution of the TA instrument (120 fs). The fitting of the decay trace at 570 nm due to TPY anion radical complex gives an average lifetime of 563 ps (Supplementary Fig. 42b). Pt@Zn-TPY-TTF CPG showed qualitatively similar TA spectra as of Zn-TPY-TTF CPG. However, the transient signal at 570 nm decays significantly faster in Pt@Zn-TPY-TTF CPG (average lifetime is 174 ps, see Supplementary Fig. 42c). Such faster decay kinetics in Pt@Zn-TPY-TTF CPG suggest electron transfer from reduced Zn-TPY to Pt NPs. Besides the TRPL and TA results, DFT calculations also revealed that in Pt@Zn-TPY-TTF CPG, the electron transfer could take place from TTF to Pt NPs via [Zn(TPY)_2_]^2+^ unit. We have investigated the possible loading positions of Pt NPs in the CPG theoretically and the details have been given in SI (Supplementary Figs. 43–44). We were able to perform the stabilization energy calculations upto four atoms Pt cluster due to computational limitation (Supplementary Fig. 44) and detailed experimental study related to Pt NPs stabilization will be carried out in future works. Furthermore, AQE for the water reduction to H_2_ was determined for the Pt@Zn-TPY-TTF CPG upon irradiating with monochromatic light of the wavelength of 400 ± 10 nm, 450 ± 10 nm, 500 ± 10 nm, 550 ± 10 nm, 600 ± 10 nm, 650 ± 10 nm, and 700 ± 10 nm (Supplementary Table 5, Supplementary Fig. 45). Notably, the highest AQE was obtained to be 14.47% at 550 ± 10 nm. These experiments suggested that the photocatalytic activity is mainly driven through intermolecular charge-transfer interaction. The H_2_ evolution using Pt@Zn-TPY-TTF CPG was examined under both light and dark conditions (Supplementary Fig. 39c). No H_2_ evolution was detected under dark condition, indicating the importance of light for water reduction. Next, photocatalysis was also performed for Pt@Zn-TPY-TTF and Zn-TPY-TTF CPG without any sacrificial donor (TEA). The aqueous dispersion of Zn-TPY-TTF CPG produced 0.92 mmol g^−1^ of H_2_ in 20 h, which is 11 times lesser than with TEA. Similarly, Pt@Zn-TPY-TTF CPG showed 30 times lesser activity without TEA (Supplementary Table 6).

### CO_2_ reduction activity of OG and CPG under laboratory condition

**Fig. 7:**
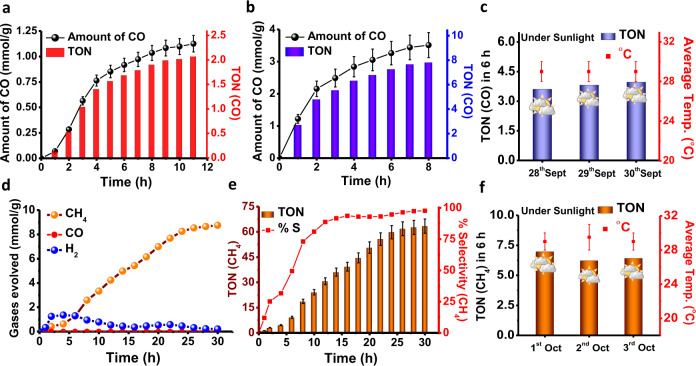
Photocatalytic activity towards CO_2_ reduction. **a** Amount and TON for CO formation in the presence of TPY-TTF OG under visible light. **b** Amount and TON for CO formation in the presence of Zn-TPY-TTF CPG under visible light. **c** TON for CO formation under sunlight from 28th to 30th Sept 2019 (10:00 am to 4:00 pm) in the presence of Zn-TPY-TTF CPG. **d** CO_2_ reduction by Pt@Zn-TPY-TTF CPG (amount of CH_4_ and H_2_ formation with time) under visible light. **e** TON value and % selectivity for CH_4_ formation using Pt@Zn-TPY-TTF CPG under visible light. **f** TON for CH_4_ formation from CO_2_ upon sunlight irradiation from 1st to 3rd Oct 2019 (10:00 am to 4:00 pm) in the presence of Pt@ Zn-TPY-TTF CPG. Error bars in panels **a**, **b** and **e** represents the standard deviations of three catalytic runs. Error bars in panels **c** and **f** represents the temperature range of photocatalytic set-up during catalysis under the sunlight irradiation.

As mentioned above, the theoretical and experimental bandgap alignment of Zn-TPY-TTF CPG and TPY-TTF OG has the potential to reduce CO_2_ as well. Therefore, we have performed visible-light-driven photocatalytic CO_2_ reduction with the xerogel of Zn-TPY-TTF CPG and also compared it with TPY-TTF OG. TEA was used as a sacrificial electron donor for CO_2_ reduction. First, screening of the solvent composition for CO_2_ reduction has been performed (Supplementary Figs. 47–48, Supplementary Table 10), and the mixture of acetonitrile: water (3:1) have shown the best activity which could be attributed to high solubility of CO_2_ in acetonitrile, whereas, water acted as a proton source^65^. Visible-light-driven CO_2_ reduction by Zn-TPY-TTF CPG yielded 3.51 mmol g^−1^ of CO in 8 h with >99% selectivity (activity = 438 μmol g^−1^ h^−1^) as shown in Fig. 7b. The AQE of CO_2_ photoreduction for Zn-TPY-TTF CPG at 550 ± 10 nm was calculated to be 0.96%. Such a selective CO production is noteworthy and one of the best results among various reported hybrid photocatalysts systems (Supplementary Table 12). The TON for Zn-TPY-TTF CPG in 8 h was calculated to be 7.8 (Fig. 7b). Recyclability test for the Zn-TPY-TTF CPG photocatalyst was performed for four cycles, similarly as employed for the water reduction. The photocatalytic performances of Zn-TPY-TTF CPG were found to be almost unchanged (Supplementary Fig. 49). Further, the photocatalytic activity of Zn-TPY-TTF CPG was examined upon isotopic labelling with ^13^CO_2_. This showed the formation of ^13^CO, which confirms that the produced CO was originated from CO_2_ (Supplementary Figs. 50–51)_._ Next, visible-light-driven photocatalytic CO_2_ reduction has also been investigated for TPY-TTF OG under a similar condition as employed for Zn-TPY-TTF CPG. The TPY-TTF OG has displayed 1.12 mmol g^−1^ CO formation in 11 h with >99% selectivity (activity = 140 μmol g^−1^ h^−1^), and the corresponding TON was estimated to be 2.1 as shown in Fig. 7a.

**Fig. 8:**
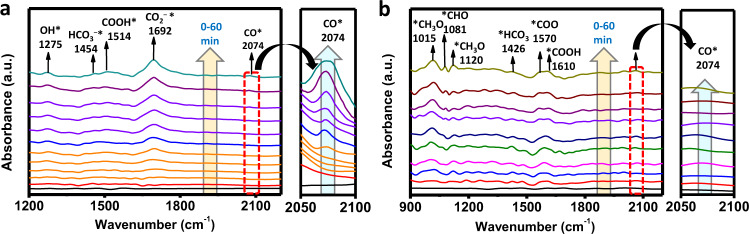
Real-time photocatalytic CO_2_ reduction monitoring through in situ DRIFT study. **a** For CO formation by Zn-TPY-TTF CPG. **b** For CH_4_ formation by Pt@Zn-TPY-TTF CPG.

We have performed in situ Diffuse Reflectance Infrared Fourier Transform (DRIFT) spectroscopic study for Zn-TPY-TTF CPG to track the reaction intermediates formed in the course of CO_2_ reduction to CO (Fig. 8a)^58^. Two peaks that appeared at 1514 and 1692 cm^−1^ were assigned for the COOH^*^ and COO^−*^ intermediates, respectively, which are the signature intermediates formed during CO_2_ reduction process (Fig. 8a)^66^. Peak observed at 1454 cm^−1^ could be attributed to symmetric stretching of the HCO_3_^−*^ ^67^. A noteworthy peak at 2074 cm^−1^ was indicated for the formation of the CO^*^. Most importantly, the peak intensity of the CO^*^ intermediate was substantially increased with reaction progress, suggesting CO^*^ formation increases with time. Based on the experimental results and in situ DRIFT study, we have computed a plausible mechanism for CO_2_ reduction, which is also in good agreement with the earlier report^68^ (Supplementary Figs. 65–66). The photocatalytic cycle was initiated by the light absorption due to CT interaction in Zn-TPY-TTF CPG, and electron transfer took place from the excited state of TTF^*^ to [Zn(TPY)_2_]^2+^ units followed by reductive quenching of TTF^+^ by TEA. As a result, the [Zn(TPY)_2_]^2+^ converted to the radical cation species [Zn(TPY˙^**−**^)(TPY)]^**+**^. Notably, the electron spin density distribution plot revealed that the electron was localized at the terpyridine unit of the catalyst that resulted in [Zn(TPY˙^**−**^)(TPY)]^**+**^ species with the stabilization energy of 0.52 eV. (Supplementary Fig. 65d and Supplementary Table 35). Next, as the reaction progress, the acetonitrile (CH_3_CN), solvent molecule replaced one ligating site of terpyridine to afford [Zn(TPY**˙**^**−**^)(η^2^-TPY)(CH_3_CN)]^**+**^ which is slightly uphill (ΔG = +0.77 eV) with a low activation barrier of 0.81 eV. This [Zn(TPY**˙**^**−**^)(η^2^-TPY)(CH_3_CN)]^**+**^ intermediate subsequently binds with CO_2_ molecule with a distance of 2.41 Å to Zn^II^ ion and yielded the [Zn(TPY)(η^2^-TPY)(COO^**−**^)]^**+**^ species (ΔG = +0.46 eV). The formulation of the intermediate as [Zn(TPY)(η^2^-TPY)(COO^**−**^)]^**+**^ rather than [Zn(TPY**˙**^**−**^)(η^2^-TPY)(COO)]^**+**^ is supported by the spin density distribution plot (Supplementary Table 38). Notably, the CO_2_ molecule acted as a monodentate ligand, and the ∠O–C–O angle was found to be 139.36°, keeping in mind that the free CO_2_ has linear geometry (Supplementary Fig. 65f and Supplementary Table 38). Here, it is worth mentioning that the one-electron charging over the [Zn(TPY)_2_]^2+^ complex favours the binding of CO_2_ molecule by increasing electron density around the metal centre, which is a prerequisite for the nucleophilic attack to the CO_2_. However, the complex [Zn(TPY)(η^2^-TPY)(COO^**−**^)]^**+**^ was further reduced to form the singlet species [Zn(TPY**˙**^**−**^)(η^2^-TPY)(COO^**−**^)] (ΔG = −0.79 eV) (Supplementary Fig. 65g and Supplementary Table 39). Next, the carboxylate centre of the singlet complex gets readily protonated and results in the formation of [Zn(TPY**˙**^**−**^)(η^2^-TPY)(COOH)]^**+**^ complex (ΔG = −2.88 eV) (Supplementary Fig. 65h and Supplementary Table 40). In the next step, subsequent protonation and water elimination from [Zn(TPY**˙**^**−**^)(η^2^-TPY)(COOH)]^**+**^ intermediate lead to the formation of [Zn(TPY)(η^2^-TPY)(CO)]^2**+**^ which is found to be a highly downhill process (ΔG = −2.55 eV). (Supplementary Fig. 65i and Supplementary Table 41). In this complex, the CO molecule is loosely attached to the Zn^II^ centre at a distance of 2.66 Å. As a result, the CO molecule can be easily released from the metal centre. Thus, the intermediate [Zn(TPY)(η^2^-TPY)(CO)]^2**+**^ gets readily reduced, leading to the subsequent removal of CO (ΔG = −1.51 eV) which regenerates the active species [Zn(TPY**˙**^**−**^)(TPY)]^**+**^ and re-enters into the catalytic cycle. Specifically, in this photocatalytic CO_2_ reduction mechanism, the Zn-terpyridine complex involves retention of the bis-terpyridine ligation to gain the original coordination environment similar to as-synthesized Zn-TPY-TTF CPG.

### CO_2_ reduction activity of Pt@Zn-TPY-TTF CPG

Next, the photocatalytic activity of Pt@Zn-TPY-TTF CPG towards CO_2_ reduction has also been examined under a similar experimental condition as applied for CPG and OG. Pt@Zn-TPY-TTF CPG showed excellent CO_2_ reduction activity, and more interestingly, CH_4_ was produced rather than CO as obtained for Zn-TPY-TTF CPG (Fig. 7d). It has already been reported in the literature that the presence of Pt NPs on the surface of semiconductor plays a key role in the formation of CH_4_^60^. The formation of CH_4_ has reached saturation in 30 h, and the corresponding yield was calculated to be 8.74 mmol g^−1^ (activity = 292 μmol g^−1^ h^−1^) (Fig. 7d). During CO_2_ reduction, a small amount of H_2_ evolution was also observed (~ 0.20 mmol g^−1^ in 30 h), which was more in the initial hours but significantly decreased as the reaction progressed with time. Thus, the selectivity of CO_2_ reduction to CH_4_ formation in 30 h was noted to be more than 97% (Fig. 7e). The TON for CH_4_ was calculated to be 63.4 (w.r.t. Pt loading on Zn-TPY-TTF CPG; Fig. 7e and Supplementary Table 3) in 30 h, which is better than many of the earlier reports (Supplementary Table 12). The recyclability of the catalyst, Pt@Zn-TPY-TTF CPG was examined for 6 h upto four cycles, and the amount of CH_4_ formation in the fourth cycle was found to be >95%, indicating high stability of the catalyst (Supplementary Fig. 56). The ICP-OES analysis of Pt@Zn-TPY-TTF CPG was also performed after the fourth catalytic cycle, which showed 2.52 wt% of Pt present in the recovered catalyst. The partial decrease in catalytic activity (<5%) could be attributed to the minimal loss of Pt NPs during the recycling process. The AQE of the Pt@Zn-TPY-TTF CPG towards CO_2_ reduction was calculated at different wavelengths using monochromatic lights (Supplementary Fig. 54 and Supplementary Table 9). The highest AQE for the CH_4_ formation was obtained as 0.81% at 550 ± 10 nm, which further confirms that the photocatalytic activity of Pt@Zn-TPY-TTF is attributed to the intermolecular charge-transfer interactions. The CO_2_ reduction was also performed under both light and dark conditions using Pt@Zn-TPY-TTF (Supplementary Fig. 55). The CH_4_ formation was not increased under dark condition, justifying the importance of light in photocatalysis. Further, photocatalysis was performed with labelled ^13^CO_2_ (isotopic labelling) using Pt@Zn-TPY-TTF (Supplementary Figs. 52–53). This showed the formation of labelled ^13^CH_4_ and confirming that CO_2_ is the actual source for the CH_4_ formation. Next, we have performed in situ DRIFT experiment for Pt@Zn-TPY-TTF in order to monitor the real-time progress of the CO_2_ reduction reaction (Fig. 8b). IR-stretching peaks observed at 1610, 1570, and 1426 cm^−1^ could be attributed to the intermediates COOH^*^, COO^*^, and HCO_3_^*^, respectively^58^. Weak intensity peak for the CO^*^ at 2062 cm^−1^ illustrated that the CO^*^ could be readily converted to other multi-electron reduction intermediates. Moreover, the characteristic intermediates for CH_4_ formation were observed at 1081 cm^−1^ (CHO^*^), 1015 and 1120 cm^−1^ (CH_3_O^*^)^58^. Based on the DRIFT study, the plausible mechanism for the CH_4_ formation was elucidated with the help of free energy calculation of different intermediates using DFT, which suggested that the Pt NPs is likely to be the catalytic centre during the CO_2_ reduction (Supplementary Figs. 67–70).

### Sunlight-driven H_2_ production

The above discussions have clearly shown that visible-light photocatalytic activity and stability of both Zn-TPY-TTF CPG and Pt@Zn-TPY-TTF CPG in the xerogel state is indeed impressive. Notably, the amount of H_2_ evolution using photocatalyst Zn-TPY-TTF CPG is higher than previously reported non-precious metal-based photocatalyst materials (Supplementary Table 8). Therefore, our next goal was to examine the potential of Zn-TPY-TTF towards H_2_ evolution upon direct sunlight irradiation at ambient condition (Supplementary Fig. 24). The experiment was performed with Zn-TPY-TTF CPG under sunlight from 10:00 am to 4:00 pm for one week from 12th to 17th April 2019 on the rooftop of our institute. The weather condition corresponding to the above-mentioned period can easily be found out on the web (www.timeanddate.com). Interestingly, maximum H_2_ evolution of 5.14 mmol g^−1^ in 6 h (activity = 857 μmol g^−1^ h^−1^) was observed on 15th April, which is comparable with the amount of H_2_ obtained under laboratory conditions (Xe-lamp irradiation). The TON for H_2_ evolution was calculated for the above-mentioned period (Fig. 5g). The highest TON value of 11.9 was obtained on 15th April 2019. Whereas the lowest TON was calculated to be 7.2 on 12th April 2019 because of partially cloudy weather (Supplementary Table 7). Next, similar to Zn-TPY-TTF CPG, we have also examined sunlight-driven photocatalytic H_2_ evolution for Pt@Zn-TPY-TTF CPG in xerogel state (Fig. 6e). The experimental condition for Pt@Zn-TPY-TTF CPG was similar to the Zn-TPY-TTF CPG. Nevertheless, experimental timing was different for the Pt@Zn- TPY-TTF CPG. The experiment with Pt@Zn-TPY-TTF CPG was performed from 27th to 29th June 2019. The highest H_2_ evolution was calculated to be 72 mmol g^−1^ in 6 h (activity = 12,000 μmol g^−1^ h^−1^) on 27th June 2019, and the corresponding TON was calculated to be 521.8 (w.r.t. Pt loading), which is indeed noteworthy (Fig. 6e; Supplementary Table 3). Whereas the lowest TON value for H_2_ evolution was found to be 376.48 (w.r.t. Pt loading) on 28th June due to partially cloudy weather.

### Sunlight-driven CO_2_ reduction

Interestingly, the potential of Zn-TPY-TTF CPG for CO_2_ reduction has also been examined under direct sunlight between 10:00 am to 4:00 pm for three days from 28th to 30th September 2019. The highest CO formation of 1.79 mmol g^−1^ was observed in 6 h (activity = 298 μmol g^−1^ h^−1^) on 30th September 2019, and the corresponding TON was calculated to be 3.9 (Fig. 7c). Next, sunlight-driven CO_2_ reduction has been performed with Pt@Zn-TPY-TTF CPG for three days from 1st to 3rd October 2019 (Fig. 7f). Similar to the laboratory conditions, Pt@Zn-TPY-TTF CPG upon sunlight irradiation has displayed CH_4_ formation (Supplementary Table 11). The highest CH_4_ formation of 0.96 mmol g^−1^ in 6 h (activity = 160 μmol g^−1^ h^−1^) was observed on 1st October 2019, and the corresponding TON value was calculated to be 6.9 (w.r.t. Pt loading; Supplementary Table 3). Whereas the lowest CH_4_ evolution with the TON value of 6.24 in 6 h (w.r.t. Pt loading) took place on 2nd October 2019. The CO or CH_4_ formation under direct sunlight irradiation is albeit lower than the laboratory condition, but the obtained amount under ambient condition is quite exciting and promising because of practical application. Furthermore, after performing sunlight-driven photocatalysis with Zn-TPY-TTF CPG and Pt@Zn-TPY-TTF CPG catalysts were recovered through centrifugation and washed with fresh water 3–4 times. The FE-SEM and TEM analysis, PXRD, FT-IR and UV-vis absorption experiments were performed for the recovered catalysts and found to be similar to the as-synthesized material, indicating that the structural integrity of the material remained intact after photocatalysis (Supplementary Figs. 57–64). Whereas the EDAX analysis ensured the presence of all the elements in similar quantity as obtained for as-synthesized Zn-TPY-TTF and Pt@Zn-TPY-TTF CPG (Supplementary Figs. 57 and 61). This unambiguously demonstrated the high stability of these catalysts during sunlight irradiation.

## Discussion

In a nutshell, we have successfully demonstrated a TTF-based soft processable metal-organic hybrid gel as a visible-light photocatalyst for H_2_ evolution and CO_2_ reduction to carbonaceous fuel such as CO/CH_4_. Here, charge-transfer-driven photocatalysis based on coordination polymer gel has been exploited, where earth-abundant metal ions play a crucial role in the spatial organization of donor–acceptor π-chromophores to drive the catalytic activity. Further, we have shown the catalytic activity of the CPG after decorating Pt NPs as co-catalyst. It enhances the rate of H_2_ production in 20 folds and dramatically changes the CO_2_ reduction product from CO to CH_4_. We have also demonstrated efficient catalytic activity of the CPG and Pt decorated CPG under sunlight with high selectivity. The real-time reaction progress of CO_2_ reduction was monitored by DRIFT studies, and based on that, a plausible mechanism of CO_2_ reduction was elucidated for CPG. The easy processability and structural tunability of LMWG offers a lot of room for designing efficient photocatalyst materials for practical application. Our work will pave the way toward designing coordination driven hybrid soft processable photocatalyst systems for solar energy driven fuel production.

## Methods

### Reagent

Tetrathiafulvalene (TTF), 1,3-diaminopropane, 4′-chloro-2,2′:6′,2′′-terpyridine, Zinc nitrate (Zn(NO_3_)_2_.6H_2_O), Ethyl-4-bromobenzoate (Br-C_6_H_4_COOEt), Cesium carbonate (Cs_2_CO_3_), Palladium acetate, Tri-tertbutyl-phosphonium tetrafluoroborate (P*tBu*_*3*_.HBF_4_), Thionyl chloride (SOCl_2_), Chloroplatinic acid (H_2_PtCl_6_.6H_2_O) were purchased from Sigma–Aldrich chemical Co. Ltd. Spectroscopic grade solvents were used for all spectroscopic studies without further purification.

### Synthesis of TPY-TTF LMWG

TTF(COOH)_4_ (634 mg, 1.65 mmol) was dissolved in 50 ml of dry tetrahydrofuran (THF), and thionyl chloride (SOCl_2_) (2.4 ml, 33 mmol) was added into it under inert condition. The reaction mixture was refluxed for 2 h at 65 °C. Then the reaction mixture was distilled at 120 °C to remove excess SOCl_2_ and yielded a solid precipitate of acid chloride. Next, the solid precipitate was dissolved in 40 ml of dry THF. Now the solution of TPY-NH_2_ (2.21 g, 7.26 mmol) in 10 ml of dry THF along with triethylamine (1.25 ml, 9 mmol) was added to the solution of acid chloride dropwise at 0 °C. The reaction was stirred at 0 °C for 12 h. The solid precipitate was formed, which was filtered and washed with chloroform and acetone to remove unreacted TPY-NH_2_. The yield of isolated dark red solid precipitate (TPY-TTF LMWG) was found to be 28%. ^1^H-NMR (600 MHz, DMSO -*d*_*6*_): *δ* = 8.80 (d, 8H), 8.71 (broad, 4H), 8.10 (m, 8H), 8.005 (d, 8H), 7.83 (s, 8H), 7.58 (m, 8H), 7.48 (d, 8 H), 7.11 (m, 8H), 4.42 (m, 4H), 3.52 (m, 8 H), 3.44 (m, 8H), 1.93 (m, 8H). ^13^C NMR {^1^H} (150 MHz, DMSO -*d*_*6*_): *δ* = 166.91, 156.25, 155.75, 155.32, 149.27, 137.42, 135.72, 131.72, 130.28, 129.52, 129.44, 127.29, 124.26, 120.98, 108.05, 37.22, 36.67, 26.82. Selected FT-IR (KBr, cm^−1^): 3376 (b), 3232 (w), 3036 (s), 2930 (w), 1635 (s), 1573 (s), 1475, 1362, 1301 (w), 1102 (w), 990 (w), 849(w), 786 (s), 611(b). MALDI-TOF (*m/z*): [M]^+^ Calcd. for C_106_H_88_N_20_O_4_S_4_, 1834.22; found [M + H]^+^, 1835.06; analysis (Calcd., found for C_106_H_88_N_20_O_4_S_4_): C (69.81, 69.41), H (4.82, 4.84), N (15.25, 15.27), S (6.78, 6.99).

### Preparation of TPY-TTF OG

TPY-TTF (10 mg) was dissolved in a mixture of MeOH, DCM and H_2_O (2:1:1 ratio) (300 μl) and sonicated for 2–3 min and then heated at 60 °C to get a homogenous viscous solution. The mixture was cooled to room temperature and kept for 2 h, which has resulted in an opaque organogel (TPY-TTF OG). The formation of the gel was confirmed by rheology measurements. Further, xerogel of TPY-TTF OG was synthesized by heating the gel at 80 °C under vacuum for 8 h. Selected FT-IR (KBr, cm^−1^) for xerogel state of TPY-TTF OG: 3392 (b), 3051 (s), 2945 (w), 1649 (s), 1581 (s), 1460 (s), 1362 (w), 1294–1096 (w), 976 (w), 839 (s), 786 (s), 619 (b).

### Preparation of Zn-TPY-TTF CPG

We have used Zn(NO_3_)_2_.6H_2_O salt to synthesize Zn-TPY-TTF CPG. TPY-TTF (10 mg, 5 μmol) was taken in the 300 μl solvent mixture of MeOH, DCM and H_2_O (2:1:1 ratio), and 10 μmol of Zn^II^ was added into it at 60 °C. The reaction mixture was heated for a few minutes to get a viscous solution and kept for 4 h at room temperature, which transformed into an opaque gel. The formation of Zn-TPY-TTF gel was confirmed by rheology test. Further, xerogel of Zn-TPY-TTF CPG was synthesized by heating the gel at 80 °C under vacuum for 8 h. Selected FT-IR for Zn-TPY-TTF CPG (KBr, cm^−1^): 3414 (b), 2922 (s), 2672 (s), 2490 (s), 1619 (s), 1468 (s), 1377 (w), 1233–1021 (w), 791 (s), 629 (b), 536 (b). Analysis (Calcd., found for C_106_H_88_N_24_O_16_S_4_Zn_2_): C (57.48, 57.34), H (4.10, 3.96), N (15.18, 15.12), S (5.79, 5.75).

### Preparation of Pt@Zn-TPY-TTF CPG

For in situ platinum nanoparticles (Pt NPs) stabilization, 1 mg of H_2_PtCl_6_·6H_2_O was dispersed in 40 ml of water containing 10 mg of Zn-TPY-TTF CPG. After continuous stirring for 1 h in a closed system, the well-dispersed solution was irradiated using 300 W xenon lamp (Newport) with a 6.0 cm long IR water filter for 2 h. Finally, the Pt nanoparticle stabilized CPG sample (Pt@Zn-TPY-TTF) was thoroughly washed with deionized (DI) water and dried under vacuum at 80 °C for 12 h. More detailed characterization of Pt@Zn-TPY-TTF CPG is provided in Supplementary Information.

### Characterization

#### General

^1^H-NMR spectra were recorded on a Bruker AVANCE-400 NMR spectrometer (at 400 MHz) and JEOL-ECZR NMR spectrometer (at 600 MHz) with chemical shifts recorded as ppm, and all spectra were calibrated against TMS. ^13^C-spectrum was recorded at 150 MHz frequency using a Varian Inova 600 MHz NMR spectrometer. UV-Vis spectra were recorded in a Perkin-Elmer Lambda 900 spectrometer. For the UV-vis absorption studies, TPY-TTF OG and Zn-TPY-TTF CPG and Pt@Zn-TPY-TTF CPG were coated on a quartz plate as a thin film. Time-resolved photoluminescence (TRPL) and photoluminescence (PL) studies were performed on an Edinburgh instrument (FLS 1000). Fourier transform infrared spectra (FT-IR) were recorded by making KBr pellets using Bruker IFS 66 v/S Spectrophotometer in the region 4000–400 cm^−1^. Thermal stability of the materials was studied using Mettler Toledo TGA 850 instrument in the temperature range of 30–800 °C with the heating rate of 5 °C/min in N_2_ atmosphere. Powder X-ray diffraction (PXRD) patterns were measured by a Bruker D8 Discover instrument using Cu Kα radiation. Atomic force microscopy (AFM) measurements were carried out with a Nasoscope model Multimode 8 Scanning Probe Microscope to analyze the morphologies of the sample surface. For this analysis, samples were dispersed in ethanol and then coated on Si wafer by a drop-casting method. The Field Emission Scanning Electron Microscopic (FE-SEM) images, elemental mapping, and Energy-dispersive X-ray spectroscopy (EDAX) analysis were recorded on a Nova Nanosem 600 FEI instrument. The xerogels were dispersed in ethanol and then drop-casted onto a small piece of silicon wafer followed by gold (Au) sputtering for FE-SEM measurements. Transmission Electron Microscopy (TEM) studies were done on JEOL JEM -3010 with an accelerating voltage of 300 kV. For this analysis, the xerogels were dispersed in ethanol and drop cast on a carbon copper grid. Elemental analyses were carried out using a Thermo Scientific Flash 2000 CHN analyzer. MALDI was performed on a Bruker daltonics Autoflex Speed MALDI-TOF System (GT0263G201) spectrometer. High-resolution mass spectrometry was carried out using Agilent Technologies 6538 UHD Accurate-Mass Q-TOFLC/MS. Metal contents in the CPGs were estimated by Inductively coupled plasma-optical emission spectrometry (ICP-OES) on Perkin-Elmer Optima 7000dv ICP-OES. For the determination of Zn and Pt, CPG samples were digested with HNO_3_ and HCl and analysed by ICP-OES.

#### Rheology

The rheological study was done in Anton Paar Rheometer MCR 302. Rheological measurements were operated in a 25 mm cone-and-plate configuration with a 0.5° cone angle. The rheology experiment was performed using the amplitude sweep method over strain % at a constant frequency (ω = 1 Hz). For each rheology measurement, the gel was prepared in 10 ml glass vial. Next, ~20 mg of gel sample was loaded onto the rheometer plate with the help of a spatula in a single shot to avoid any damage to the loaded sample. Further, data accuracy was ensured by repeating these experiments a minimum of three times.

#### Critical point drying

Tousimis Autosamdri@931 was used for critical-point drying (CPD) of the gel samples. After gel preparation, solvents present in CPG, were exchanged with ethanol using a gradient of ethanol/water mixtures (40–100 %). Next, the ethanol exchanged gel samples were then transferred to a stainless-steel cage with wire mesh followed by critically point dried with supercritical CO_2_.

#### Transient absorption

Femtosecond transient absorption (TA) experiments were performed using an amplified Ti:sapphire laser system (800 nm, 60 fs, 1 kHz, 3 mJ) from Amplitude Technologies, France, and a pump-probe set-up (Excipro) from CDP Corporation, Russia. A fraction of the fundamental laser beam was used to generate 400 nm pump pulse in a 0.2 mm thick BBO crystal. Another small fraction of 800 nm pulse was used to generate broad continuum pulse (350–750 nm) in a rotating calcium fluoride window. The time delay between the pump and probe pulses was maintained by using an optical delay stage in the probe path. To minimize the noise in the transient signal, a part of the probe beam, known as reference pulse, was passed through the unexcited region of the sample and detected simultaneously with the probe pulse using a dual diode array-based spectrometer. The probe pulse was focussed in the sample and spatially overlapped with the focussed pump pulse. To obtained the pump induced changes in the absorbance of the sample (ΔA), the alternate pump pulses were blocked with the help of a synchronized chopper. Each transient spectra were collected after averaging for 2 s at each delay time. Samples were dispersed in methanol and taken in a rotating cell to avoid their photodecomposition. The instrument response function of the TA set-up was 120 fs.

#### Mott-Schottky

The energy band structure of TPY-TTF OG and Zn-TPY-TTF CPG and Pt@Zn-TPY-TTF CPG was depicted by the Mott-Schottky (MS) analysis (at 1000 Hz, from −2.0 to +2.0 V) using ITO as a working electrode (WE) in N_2_-purged aqueous solution of 0.5 M Na_2_SO_4_ at pH = 7, Pt as a counter electrode (CE) and Ag/AgCl as a reference electrode (RE). An electrochemical ink was prepared by making a dispersion of a mixture of catalyst (2.0 mg) in the solvent mixture of isopropanol (500 μl), water (500 µl), and Nafion (14 µl). Upon sonication for 20 min, a well-dispersed ink (3.5 μl) was drop cast over the ITO electrode and allowed to dry for 3 h under ambient condition.

#### Photocurrent

The similar set-up was used for photocurrent measurements as employed for Mott-Schottky analysis. Here, the photocurrent study was performed for TPY-TTF OG, Zn-TPY-TTF CPG, and Pt@ Zn-TPY-TTF CPG upon consecutive light “ON-OFF” cycles for 30 s over 10 cycles.

#### Electrochemical impedance spectroscopy (EIS)

This experiment was performed in a three-electrode cell configuration with a glassy carbon electrode as the WE, platinum as a CE, and Ag/AgCl as a RE. 0.5 M Na_2_SO_4_ was used as an electrolyte at pH = 7. An electrochemical ink was prepared by making a dispersion of a mixture of catalyst (2.0 mg) in the solvent mixture of isopropanol (500 μl) and water (500 μl). Upon sonication for 30 min, a well-dispersed ink (3.5 μl) was drop cast over the GC electrode and allowed to dry for 3 h under ambient condition. EIS was recorded at −1.2 V_RHE_ applied bias from 0.1 Hz to 100 kHz (under the dark condition and visible-light irradiation).

### Photocatalytic experiments

#### Experimental set-up for photocatalytic water reduction

Photocatalytic H_2_ evolution experiments were carried out in an 80 mL self-designed borosilicate glass cell containing a magnetic stir bar sealed with a small septum (picture of photocatalytic cell is given in Supplementary Fig. 23). For photocatalytic experiment, 1 mg catalyst was dispersed in 38 mL water containing 2 ml of triethylamine (TEA) as a sacrificial agent. The suspension was ultrasonicated to make a homogeneous dispersion. The reaction mixture was then purged with N_2_ for 30 min to remove any traces of dissolved H_2_ gas, which was ensured by GC-analysis before performing the photocatalysis. The reaction mixture was irradiated with a 300 W Xenon lamp (Newport) fitted with a 12 cm path length of water filter for removal of IR radiation. A visible bandpass filter (400–750 nm) was used to block the UV light. The Headspace gases were sampled using Hamilton air-tight syringes by injecting 250 µL into the gas chromatograph (Agilent CN15343150). Gas Chromatography referencing was done against a standard (H_2_/N_2_) gas mixture with a known concentration of hydrogen for the calibration curve, where N_2_ was used as a carrier gas, and a thermal conductivity detector (TCD) was used for H_2_ detection (Supplementary Fig. 25). Notably, no hydrogen evolution was observed for a mixture of water/5 Vol % TEA under visible-light irradiation in the absence of a photocatalyst.

#### Experimental set-up for the photocatalytic CO_2_ reduction

The photocatalytic CO_2_ reduction reaction was carried out in a similar reaction vessel as discussed above for the water reduction. Notably, a mixture of acetonitrile (CH_3_CN) and H_2_O in 3:1 ratio was used as a solvent for the CO_2_ reduction. In short, 38 ml solvent mixture (CH_3_CN: H_2_O in 3:1), 2 ml of TEA as a sacrificial electron donor, and 1 mg of the catalyst were taken in a reaction flask and dispersed uniformly through sonication. The reaction vessel was sealed with a septum and then purged with CO_2_ of 99.9% purity for ~30 min in order to make CO_2_ saturated atmosphere. The reaction mixture was irradiated with visible light as employed for water reduction. During the CO_2_ reduction reaction, the gas in the headspace of the reaction vessel was analyzed qualitatively and quantitatively by GCMS-QP2020. During light exposure, the evolved gases in the headspace of the reaction vessel were collected by a hamilton syringe and injected in GC-MS at every 1 h time interval until product production ceased. The mass detector was used to analyze the mass of evolved products such as CO, CH_4_, CH_3_OH, HCOOH, and CO_3_^2−^. The H_2_, CO, and CH_4_ were detected by RT®-Msieve 5 A column (45 meters, 0.32 mmID, 30 μmdf). To detect the HCOOH, Stabilwax®-DA (30 meters, 0.18 mmID, 0.18 μmdf) column was used, and for Methanol, SH®-Rxi-5Sil MS (30 meters, 0.25 mmID, 0.32 μmdf) column was used in GCMS. The calibration was done by a standard gas mixture of H_2_, CO, and CH_4_ of different concentrations in ppm-level (Supplementary Fig. 46). Importantly, the GCMS has a detection limit of 1.0 ppm for H_2_, CO, and CH_4_. After the photocatalysis, the reaction mixture was filtered to remove the residual solid, and the solution was further analyzed to determine the amount of HCOOH/MeOH. All described data points are the average of at least 3 experiments. For isotopic labelling experiment, one litre ^13^CO_2_ gas cylinder was purchased from Sigma–Aldrich (details: 99.0% ATOM % ^13^C, <3 Atom % ^18^O; M.W. 45.00 g/mol). We have purged the ^13^CO_2_ for 10 min in a controlled manner to the photocatalytic reaction mixture of Zn-TPY-TTF CPG as well as Pt@Zn-TPY-TTF CPG.

#### In situ diffuse reflectance infrared Fourier transform (DRIFT) spectroscopy measurements

The in situ DRIFT measurements were carried out by FT-IR spectrophotometer (BRUKER, Pat. US, 034, 944) within a photoreactor. The 6 mg of catalyst was evenly spread over a glass disc of 1 cm diameter and placed inside the photoreactor for monitoring the reaction progress of photocatalytic CO_2_ reduction. Next, the air was removed using vacuum inside the cell, and then 99.99% CO_2_ gas along with water vapour was passed for 15 min inside the photoreactor. At last, the visible light was irradiated on catalyst by 150 W white LED light (>400 nm). In situ FT-IR signal was collected through MCT detector at regular time interval.

#### Lifetime calculations

Lifetime data for Zn-TPY-TTF CPG and Pt@Zn-TPY-TTF CPG were collected upon exciting at 510 nm. The average lifetime is calculated using following formula.

1 $${{{{{\tau }}}}}_{{{{{{\rm{avg}}}}}}}\,({{{{{\rm{ns}}}}}})=\frac{\Sigma {{{{{{\rm{A}}}}}}}_{{{{{{\rm{i}}}}}}}{{{{{\tau }}}}}_{{{{{{\rm{i}}}}}}}^{2}}{\Sigma {{{{{{\rm{A}}}}}}}_{{{{{{\rm{i}}}}}}}{{{{{\tau }}}}}_{{{{{{\rm{i}}}}}}}}$$

Where, τ_avg_ = average lifetime in nano-seconds, ΣA_i_ = sum of percentage of all the components that exist in the excited state, Στ_i_ = sum of excited-state lifetime of all the component. The details are provided in Supplementary Table 4.

#### Turnover Number

The turnover number (TON) is calculated by using the below formula 2.

2 $${\rm TON}={\rm Amount}\; {\rm of}\; {\rm product}\; {\rm evolved} \;(\mu {\rm mol})/{\rm amount}\; {\rm of}\; {\rm catalyst} \;(\mu {\rm mol})$$

For hydrogen production. 1.0 mg of TPY-TTF OG is equivalent to 0.545 μmol and the amount of H_2_ evolved was found to be 2 μmol from 1 mg of TPY-TTF in 20 h. Therefore, TON was calculated for TPY-TTF OG in 20 h is 3.6. The unit of Zn-TPY-TTF CPG was calculated based on a binding ratio of TPY-TTF LMWG with Zn(NO_3_)_2_ in CPG (i.e. 1:2). Therefore, unit formula weight of Zn-TPY-TTF CPG is 2212.94. Therefore, 1 mg Zn-TPY-TTF CPG catalyst contains 0.451 μmol and the corresponding TON was found to be 23.5 in 20 h. Similarly, TON was also calculated for CO formation in presence of Zn-TPY-TTF CPG photocatalyst. The detailed TON calculation for H_2_ production and CH_4_ formation in presence of Pt@Zn-TPY-TTF CPG are provided in Supplementary Table 3.

#### Apparent quantum efficiency

The apparent quantum efficiency (AQE) is defined by the ratio of the effective electron used for product formation to the total input photon flux. The AQE is calculated by using the below formula.

3 $${{{\mathrm{AQE}}}} ({\%})=\left[\frac{{{{{{\rm{Effectiveelectrons}}}}}}}{{{{{{\rm{Totalphotons}}}}}}}\right]\times 100 {\%} =\left[\frac{{{{{{\rm{n}}}}}}\times {{{{{\rm{Y}}}}}}\times {{{{{\rm{N}}}}}}}{{{{{{\rm{\theta }}}}}}\times {{{{{\rm{T}}}}}}\times {{{{{\rm{S}}}}}}}\right]\times 100 {\%}$$

Where, n is the number of electrons used in the photocatalysis process, *Y* is the yield of evolved gas from the sample (mol), *N* is the Avogadro’s number (6.022 × 10^23^ mol^−1^), *θ* is the photon flux, *T* is the irradiation time, and *S* is the illumination area. The photon flux was calculated at 400 ± 10 nm, 450 ± 10 nm, 500 ± 10 nm, 550 ± 10 nm, 600 ± 10 nm, 650 ± 10 nm, and 700 ± 10 nm by using separate bandpass filters with the help of power meter. The power meter (model: LaserCheck: 0623G19R) used for the experiment was purchased from Coherent.

Since the reduction of a water molecule to the H_2_ molecule requires two-electron (*n* = 2). The following calculation is based on data from water photoreduction with Zn-TPY-TTF CPG for 1 h, AQE was calculated as 0.76% at 550 ± 10 nm (the amount of H_2_ evolved in 1 h was found to be 0.094 μmol). Meanwhile, the AQE of H_2_ for the Pt@Zn-TPY-TTF CPG was also calculated at the various wavelength (400–700 nm), and the maximum was found at 550 ± 10 nm, which was calculated to be 14.47%. The detailed calculation for H_2_ production in presence of Pt@Zn-TPY-TTF CPG at different wavelength is provided in Supplementary Table 5.

The reduction of a CO_2_ molecule to the CO molecule requires two-electron (*n* = 2). The following calculation is based on data from CO_2_ photoreduction with Zn-TPY-TTF CPG at 550 ± 10 nm, AQE was calculated as 0.96%. (the amount of CO evolved was found to be 0.121 μmol in 1 h). Similarly, the reduction of a CO_2_ molecule to the CH_4_ molecule requires eight-electron (*n* = 8). Therefore, the AQE for CH_4_ formation by Pt@Zn-TTF-TPY CPG was calculated at various wavelengths (400–700 nm) and found the maximum efficiency at 550 ± 10 nm as 0.81%. The detailed calculation for CO_2_ photoreduction to CH_4_ in presence of Pt@Zn-TPY-TTF CPG at different wavelength is provided in Supplementary Table 9.

## Supplementary information

Supplementary Information

Peer review file

## Data Availability

All other data supporting the findings are available in the article as well as the supplementary information files and can be found from the authors on reasonable request.
