# Supplementary material for: Charge-transfer regulated visible light driven photocatalytic H2 production and CO2 reduction in tetrathiafulvalene based coordination polymer gel
Source: Nat Commun. 2021 Dec 16;12:7313. doi: 10.1038/s41467-021-27457-4 (PMC8677803; doi:10.1038/s41467-021-27457-4)
Supplement: Supplementary file 1 — Supplementary Information [file 41467_2021_27457_MOESM1_ESM.pdf]

Supporting Information

Charge-transfer regulated visible light driven photocatalytic H_2 production and CO_2 reduction in tetrathiafulvalene based coordination polymer gel

Verma et al.

S1. Synthesis and characterization of TPY-TTF LMWG.

S1. (i) Synthesis of 2,3,6,7-Tetra (4-carboxyphenyl) Tetrathiafulvalene [TTF(COOH)$_4$]. Synthesis and characterization of TTF(COOH)$_4$ are already reported in the literature.[1] We have followed a similar procedure and characterization data was in good agreement with the literature report. Briefly, Pd(OAc)$_2$ (39.6 mg, 0.176 mmol), P*t*Bu$_3$.HBF$_4$ (153.53 mg, 0.529 mmol), Cs$_2$CO$_3$ (1149.4 mg, 3.528 mmol) were placed in a 20 mL reaction flask under N$_2$ atmosphere. THF (5.0 mL) was added, and the reaction mixture was stirred for 10 min at reflux. Then, a solution of Tetrathiafulvalene (120 mg, 0.588 mmol) and Ethyl-4-bromobenzoate (673.46 mg, 2.94 mmol) in THF (5.0 mL) was added. The reaction mixture was heated to reflux for 15 h. The organic compound was extracted with chloroform three times. The combined organic layer was washed with brine, dried over anhydrous Na$_2$SO$_4$, and concentrated in vacuum. Chromatographic purification on silica gel using hexane-chloroform as eluent afforded tetrathiafulvalene-tetra-benzoate (Et$_4$TTFTB) as a red solid product (260 mg). Further, a 50 mL flask was charged with Et$_4$TTFTB (260 mg) and subjected to three cycles of evacuation and refilling with N$_2$. Next, degassed methanol (50 ml) and THF (20 ml) were added into it to generate a suspension. In a separate flask, sodium hydroxide aq. solution (6 g, 40 mL) was prepared, then degassed with N$_2$ and added to Et$_4$TTFTB solution under N$_2$ atmosphere. The reaction mixture was heated at 60°C for 12 h. The reaction was then cooled to room temperature, and the solvents (THF and MeOH) were removed in a rotary evaporator under reduced pressure. Further, 1M solution of HCl was added to the reaction mixture that afforded a maroon precipitate of TTF(COOH)$_4$, which was collected by filtration and washed with water followed by drying under high vacuum for 12 h. Yield: 189 mg (0.276 mmol, 84 %). ^{1}H-NMR (400 MHz, DMSO-d_6): δ = 8.13 (d, 8H, ArH), 7.63 (d, 8H, ArH). HRMS (m/z): [M]$^+$ calcd. for C$_{34}$H$_{20}$O$_8$S$_4$, 684.0041; found, 685.0045; analysis (Calcd., found for C$_{34}$H$_{20}$O$_8$S$_4$): C (58.65, 59.63), H (2.89, 2.94), S (18.71, 18.73).

S1. (ii) Synthesis of 2,2′:6′,2″-terpyridin-4′-yl-propane-1,3-diamine (TPY-NH$_2$). The 2′;6′,2″-terpyridin-4′-yl-propane-1,3-diamine was synthesized by following a reported procedure.[2] Briefly, 4′-chloro- 2,2′:6′,2″-terpyridine, (300 mg, 1.12 mmol) was dissolved in 1,3-diamino propane (2.16 ml). The reaction mixture was then refluxed at 120° C for 10 h. After cooling to room temperature, distilled water (25 mL) was added, which has yielded a white precipitate. The white solid was further dissolved in dichloromethane and washed twice with distilled water. The organic layer was combined and dried over anhydrous Na$_2$SO$_4$. The

solvent was removed under reduced pressure to yield a solid white product. Yield: 82%. ^{1}H-NMR (400 MHz, $CDCl_3$): δ = 8.53 (d, 2H, ArH), 8.52 (d, 2H, ArH), 7.76 (t, 2H, ArH), 7.60 (s, 2H, ArH), 7.25 (t, 2H, ArH), 5.16 (t, 1H, NH), 3.41 (m, 2H, $NHCH_2$), 2.84 (m, 2H, CH_2), 1.77 (m, 2H, NH_2CH_2). Selected FT-IR (KBr, cm^{-1}): 3340 (b), 2965 (m), 1610-1560 (s), 1464 (m), 1402 (m), 1261 (m), 1094-981 (s), 791 (s). HRMS (m/z): $[M]^+$ calcd. for $C_{18}H_{19}N_5$, 305.1640; found $[M+H]^+$, 306.1715; analysis (Calcd., found for $C_{18}H_{19}N_5$): C (70.81, 70.90), H (6.22, 6.11), N (22.95, 22.83).

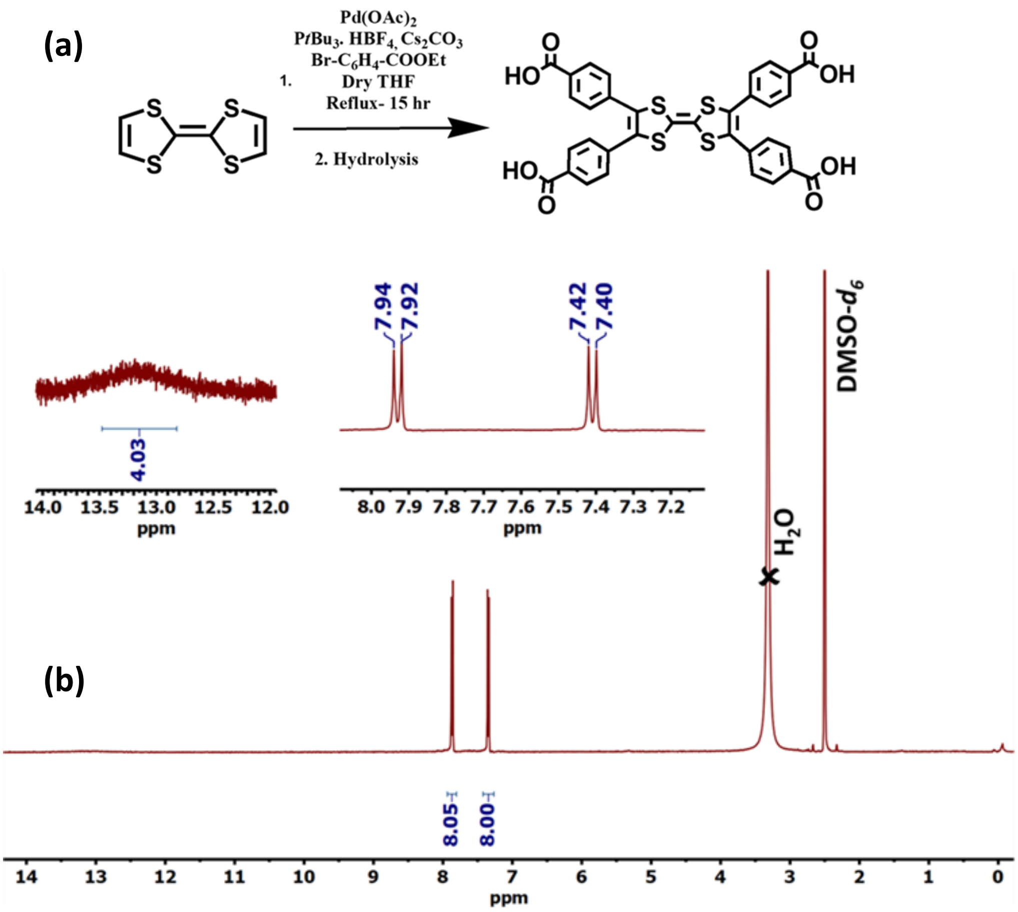

Supplementary Figure 1. (a) Synthetic scheme and (b) ^{1}H NMR of **TTF(COOH)$_4$** in DMSO-*d*$_6$.

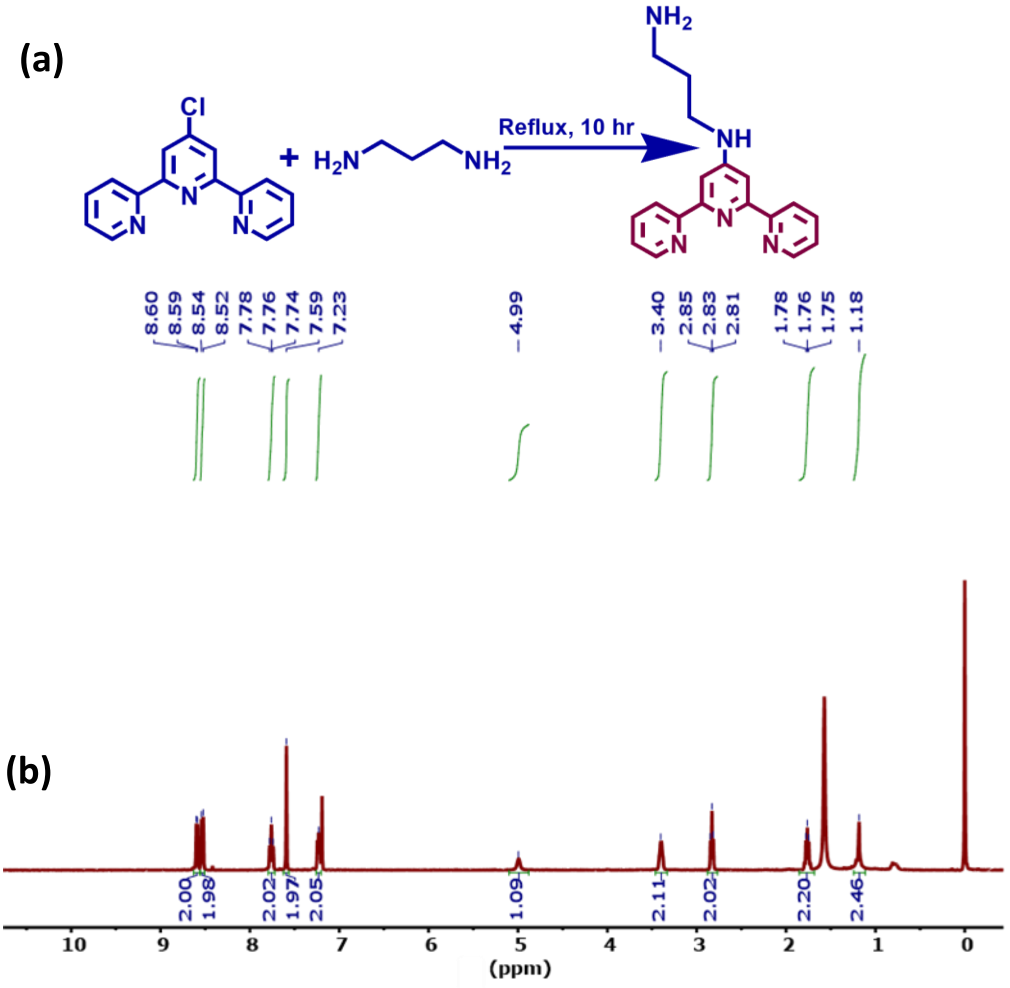

Supplementary Figure 2. (a) Synthetic scheme and (b) ^{1}H NMR of **TPY-NH$_2$** in $CDCl_3$.

S1. (iii) Characterization of TPY-TTF LMWG.

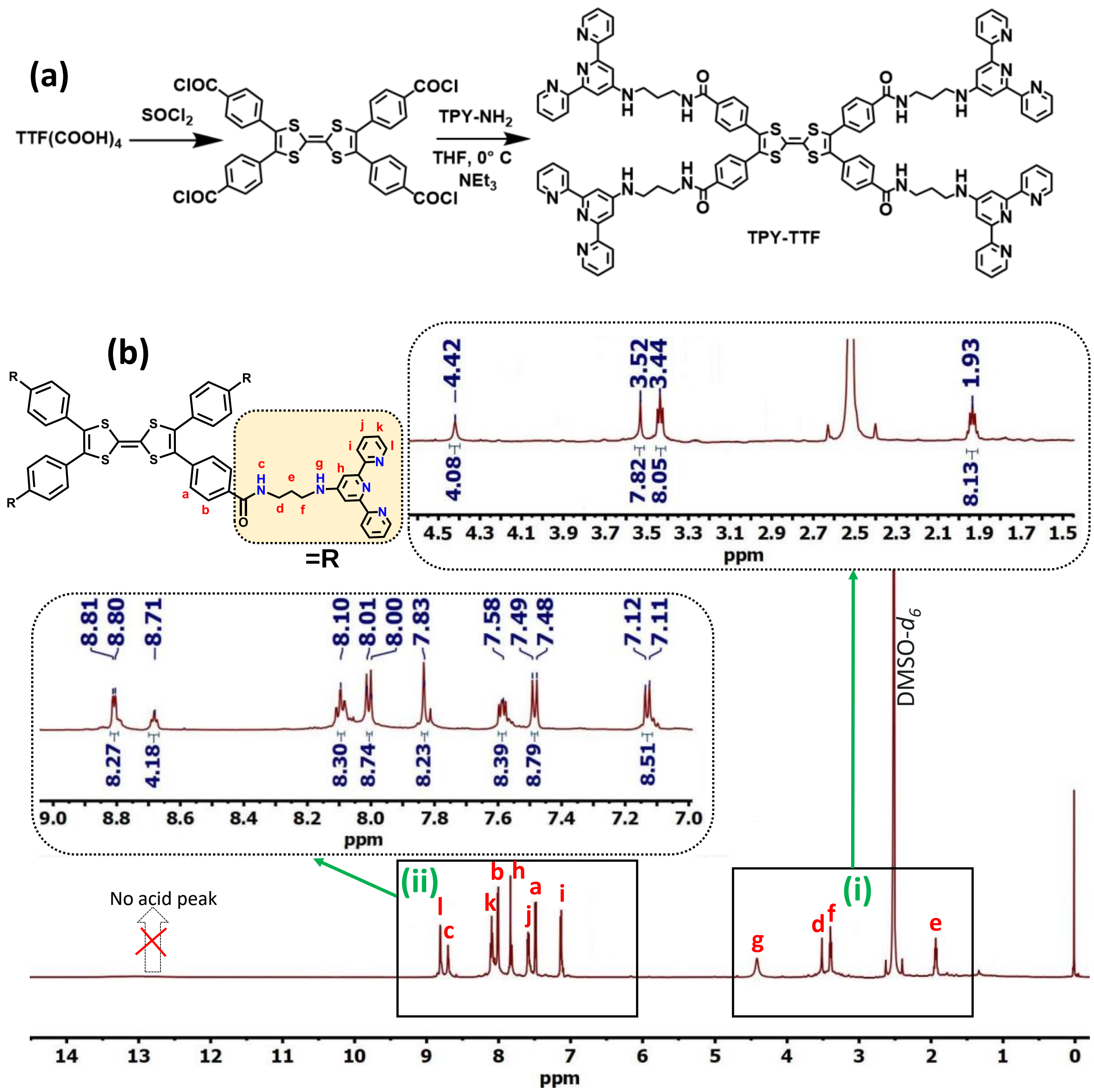

Supplementary Figure 3. (a) Synthetic scheme and (b) ^{1}H-NMR spectrum of **TPY-TTF** LMWG in DMSO-d_6.

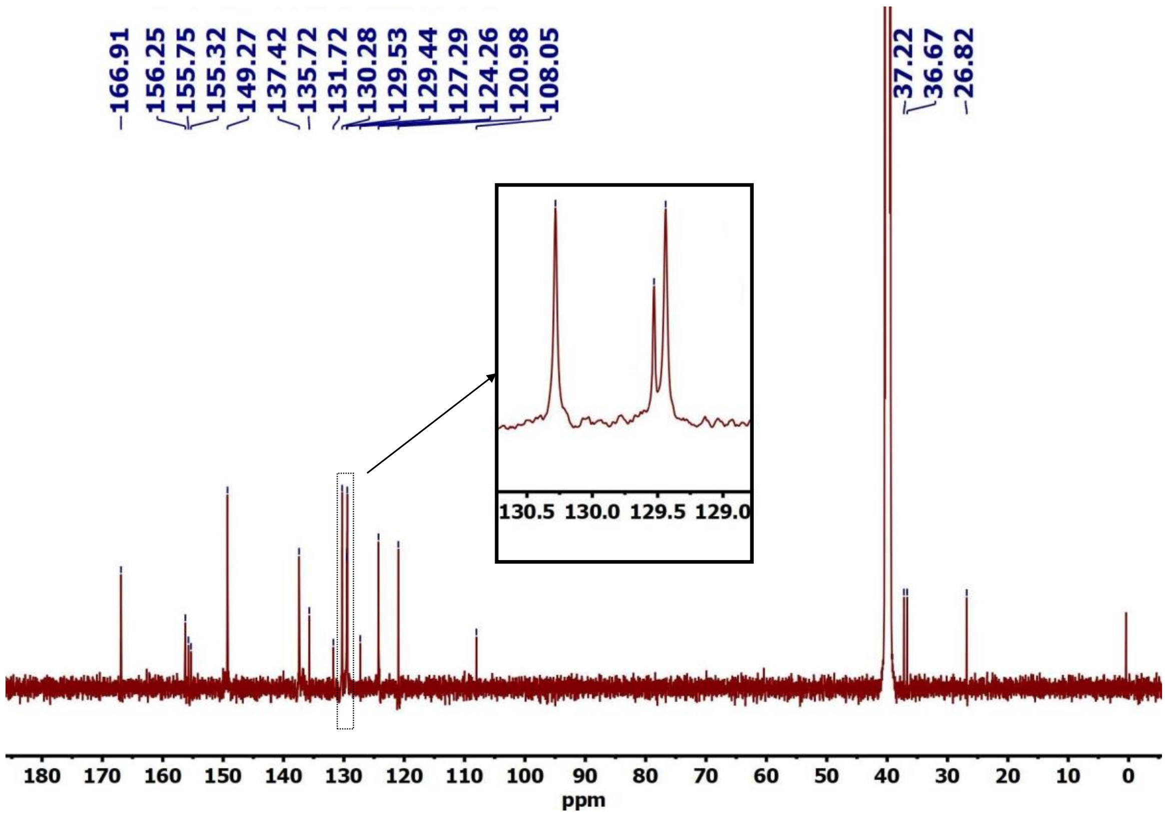

Supplementary Figure 4. ^{13}C-NMR spectrum of **TPY-TTF** LMWG in DMSO-d_6.

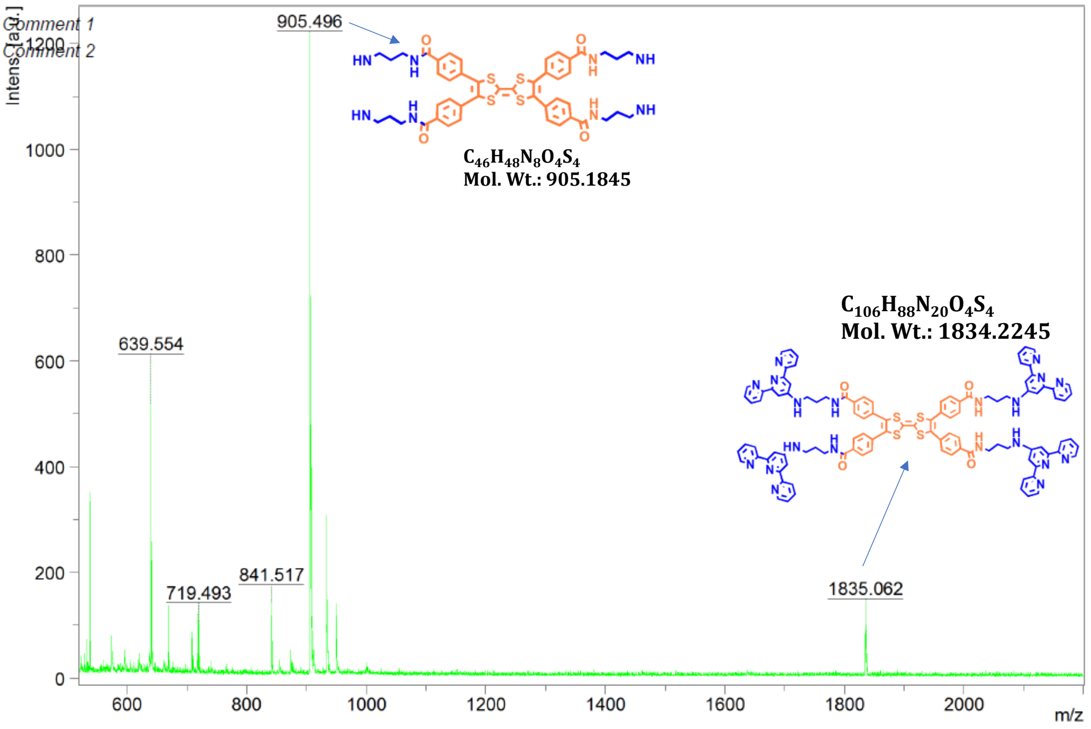

Supplementary Figure 5. MALDI-TOF mass spectrum for **TPY-TTF** LMWG.

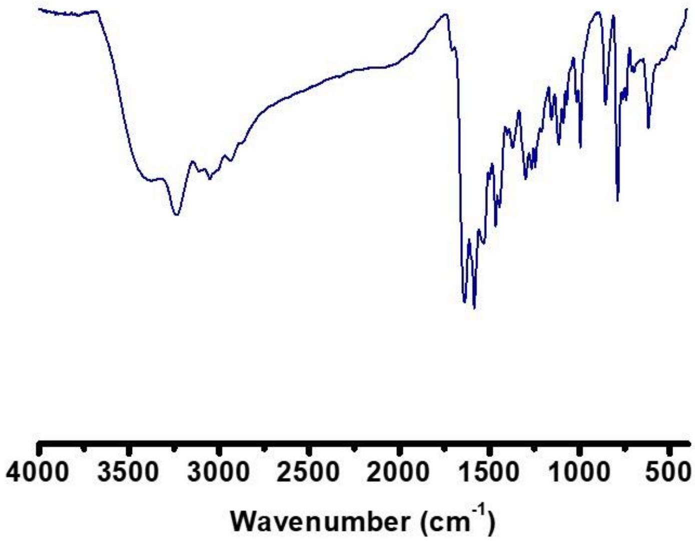

Supplementary Figure 6. FT-IR for **TPY-TTF** LMWG.

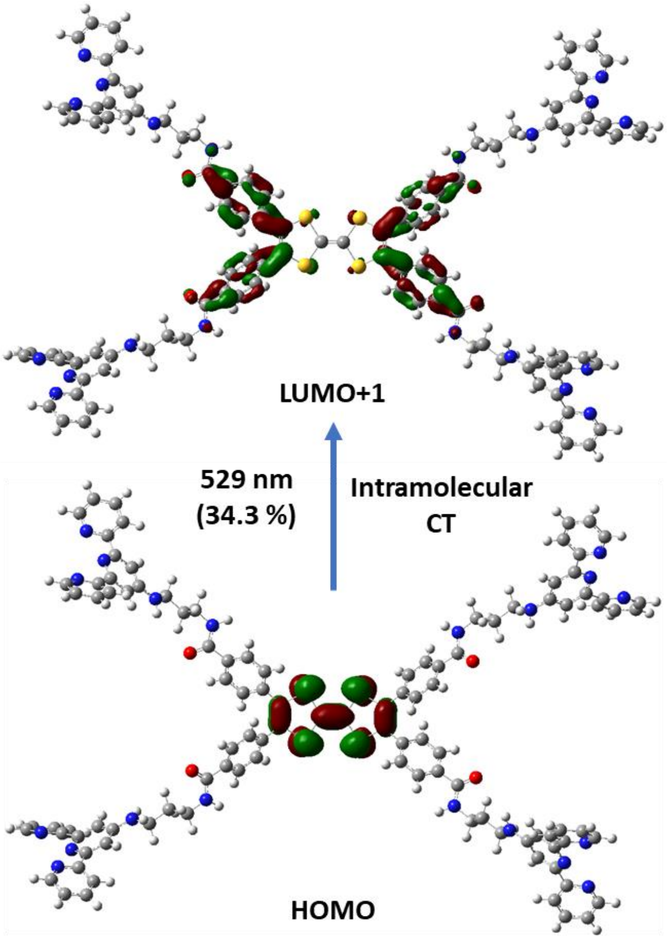

Supplementary Figure 7. Optimized structures by the TD-DFT calculations and respective contributions in intramolecular charge transfer (CT) transitions for **TPY-TTF** LMWG in methanol.

S2. Preparation and Characterization of TPY-TTF OG.

Supplementary Table 1: Gelation ability of **TPY-TTF** LMWG in different conditions:

No.	**Solvents Composition**	**Solvent Ratio**	Heating/Cooling	**Gelation ability**
1.	MeOH: DCM	(1:1)	60°/25° C	Solution
2.	MeOH: H_2O	(1:1)	60°/25° C	Precipitate
3.	DCM: H_2O	(1:1)	60°/25° C	Precipitate
4.	MeOH: DCM: H_2O	(1:1:1)	60°/25° C	Precipitate
5.	MeOH: DCM: H_2O	(2:2:1)	60°/25° C	Partial gel
6.	MeOH: DCM: H_2O	(2:1:1)	60°/25° C	**Gel***

*CGC (Critical Gelator Concentration) = 0.005 mmol; DCM= Dichloromethane; MeOH= Methanol; H_2O= Water.

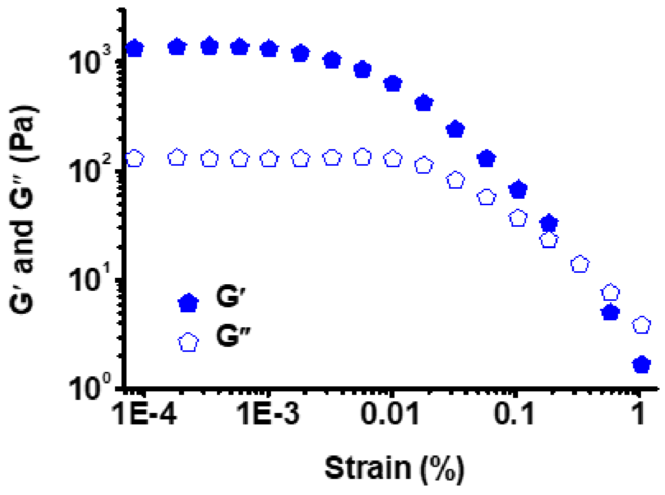

Supplementary Figure 8. Strain-sweep rheology plot for **TPY-TTF OG** at 25° C. The closed symbols and open symbols represent the storage modulus (G′) and the loss modulus (G″), respectively.

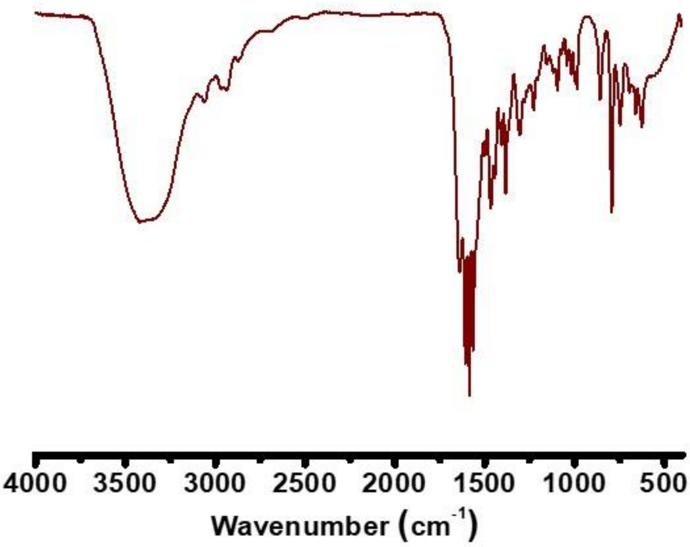

Supplementary Figure 9. FT-IR for **TPY-TTF OG**.

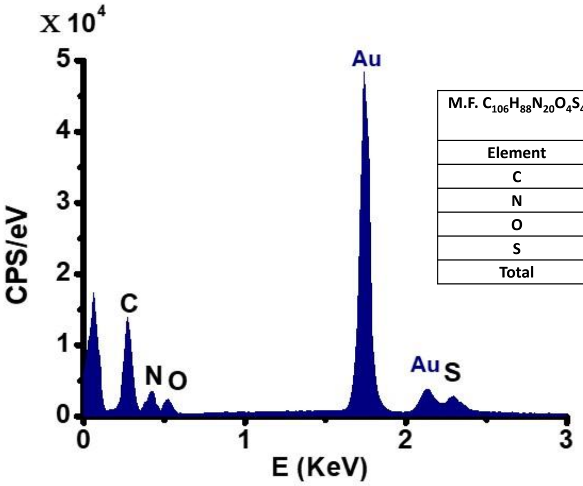

M.F. $C_{106}H_{88}N_{20}O_4S_4$	Wt%	
**Element**	**Exp.**	**Cal.**
**C**	**71.82**	**69.41**
**N**	**16.51**	**15.27**
**O**	**4.46**	**3.49**
**S**	**7.21**	**6.99**
**Total**	**100**	

Supplementary Figure 10. EDAX analysis for **TPY-TTF OG**.

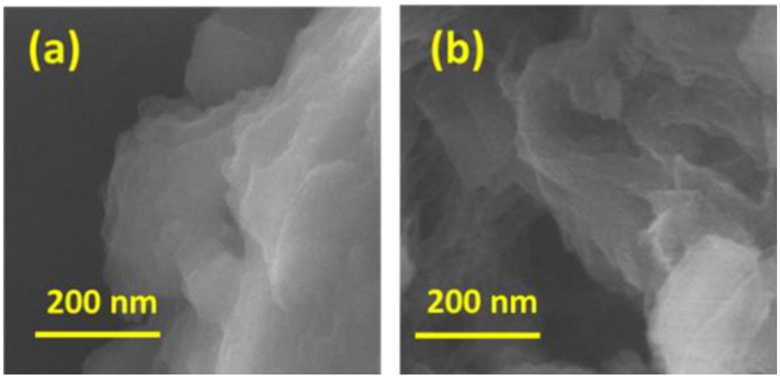

Supplementary Figure 11. SEM images of **TPY-TTF OG** after drying in different conditions: (a) under vacuum at 80°C and (b) under critical point drying (CPD).

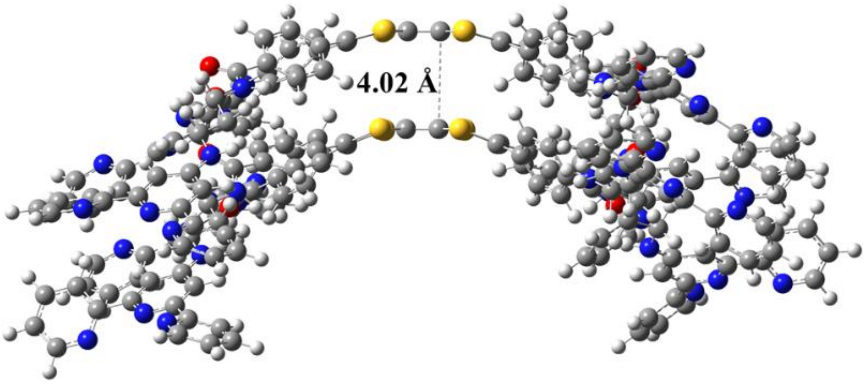

Supplementary Figure 12. Optimized structure for TTF---TTF stacked model in **TPY-TTF OG** self-assembly.

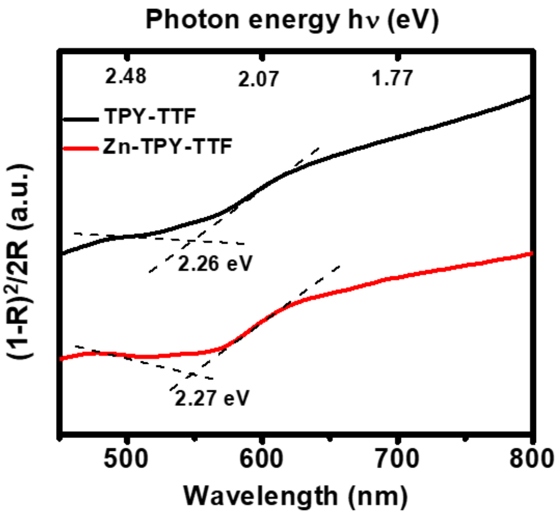

Supplementary Figure 13. Kubelka-Munk plot for **TPY-TTF OG** and **Zn-TPY-TTF CPG** obtained by UV-visible diffused reflectance spectrum.

S3. Characterization of Zn-TPY-TTF CPG.

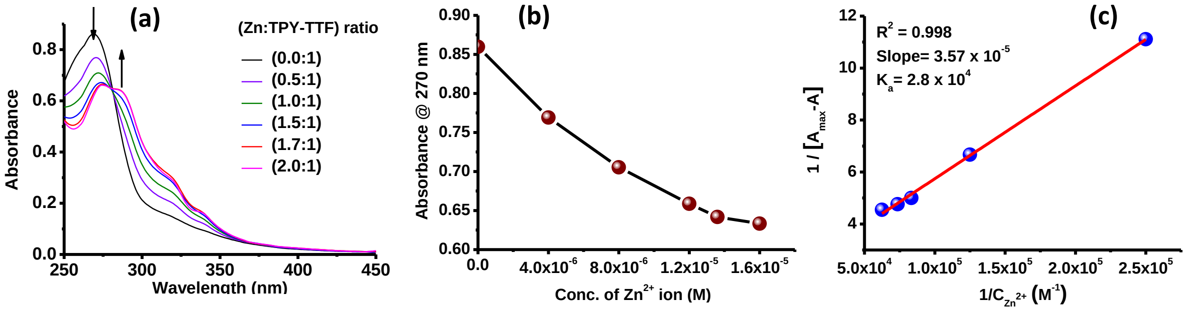

Supplementary Figure 14. Benesi-Hildebrand plot obtained from the titration experiment of **TPY-TTF** LMWG (8×10^{-6} M in methanol) with Zn^{II} (8×10^{-4} M in methanol: stock solution). (a) Titration of **TPY-TTF** LMWG with Zn^{II} ion. (b) Changes in absorption at 270 nm with increasing concentration of Zn^{II} ion. (c) Benesi-Hildebrand plot for the binding affinity of LMWG with Zn^{II} ion.

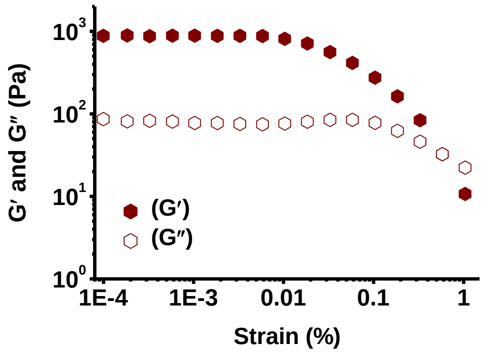

Supplementary Figure 15. Strain-sweep rheology plot for **Zn-TPY-TTF CPG** at 25° C. The closed and open symbols represent the storage modulus (G′) and the loss modulus (G″), respectively.

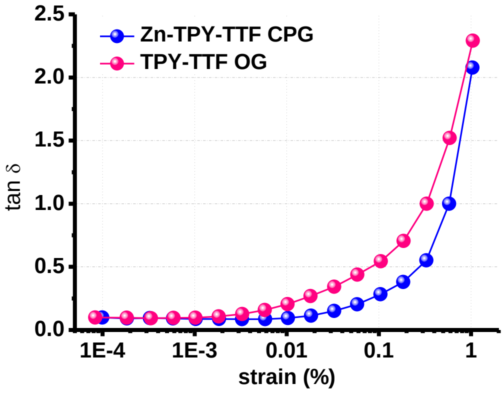

Supplementary Figure 16. Value of tan δ with strain amplitude sweep for both **TPY-TTF OG** and **Zn-TPY-TTF CPG**.

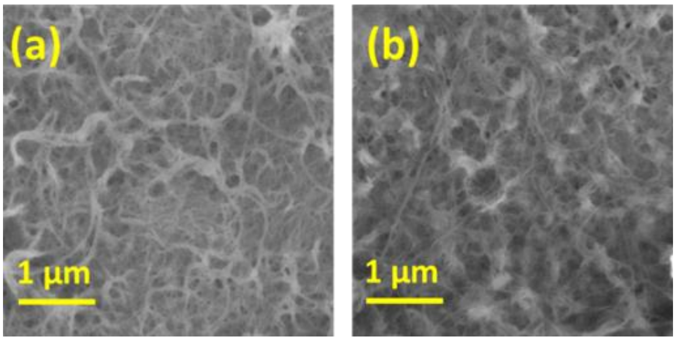

Supplementary Figure 17. FE-SEM images of **Zn-TPY-TTF CPG** after drying: (a) under vacuum at 80°C and (b) under Critical Point Drying (CPD).

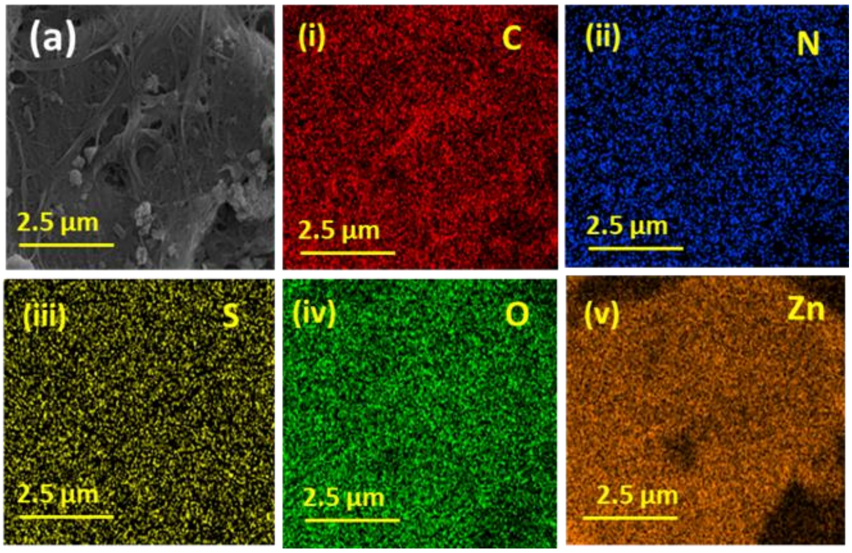

Supplementary Figure 18. Elemental mapping for **Zn-TPY-TTF CPG**.

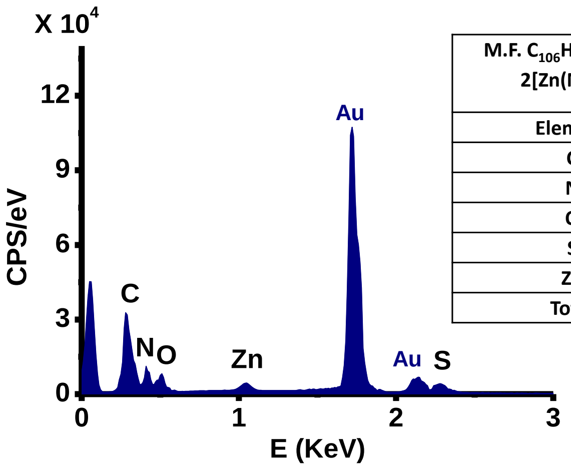

M.F. $C_{106}H_{88}N_{20}O_4S_4$. $2[Zn(NO_3)_2]$	Wt%	
Element	Exp.	Cal.
C	58.74	57.53
N	16.75	15.19
O	12.45	11.57
S	6.42	5.79
Zn	5.64	5.91
Total	100	

Supplementary Figure 19. EDAX analysis for **Zn-TPY-TTF CPG**.

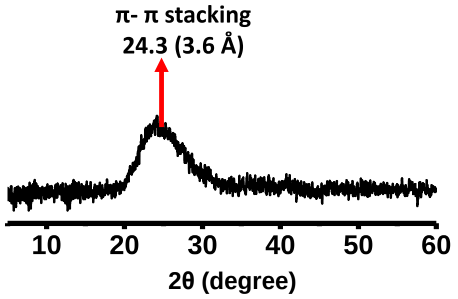

Supplementary Figure 20. PXRD pattern for **Zn-TPY-TTF CPG** in the gel state.

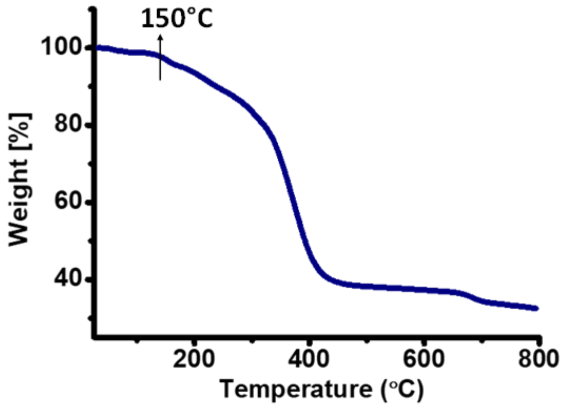

Supplementary Figure 21. TGA plot for **Zn-TPY-TTF CPG** xerogel under N_2 atmosphere.

Supplementary Table 2. Comparison of experimental and theoretical (TD-DFT) absorption peak for TPY-TTF LMWG (monomer), TPY-TTF (organogel-OG), and Zn-TPY-TTF (Coordination polymer gel-CPG).

Species	Exp. medium	Abs. λ (nm)		Type of CT Interactions		Conclusion
		Exp.	Theo.	Intra CT	Inter CT	
**TPY-TTF LMWG**	MeOH Sol. (monomer)	~520	529	HOMO to LUMO+1 34.3 %	--	Intra CT TTF→PhCONH-
**TPY-TTF OG**	Vacuum (stacked model)	~510	498	HOMO to LUMO+2 13.4 %	HOMO to LUMO+3 9.3%	Intra CT (TTF→PhCONH-) + Inter CT (TTF→TPY)
		~565	564	HOMO to LUMO+1 52.6 %	--	Intra CT TTF→PhCONH-
**Zn-TPY-TTF CPG**	Vacuum (stacked model)	~510	500	HOMO to LUMO+14 14.9 %	HOMO to LUMO+9 6.8 %	Intra CT (TTF→PhCONH) + Inter CT (TTF→Zn-TPY)
		~565	553	HOMO to LUMO+4 5.8 %	**HOMO to LUMO+3 46.3 %**	**Inter CT mainly** TTF→Zn-TPY

Note. The pure intra and intermolecular CT transitions have been considered to assess experimental absorption bands observed for **TPY-TTF** monomer, **TPY-TTF OG** and **Zn-TPY-TTF CPG**. (Other contributions besides the pure intra and inter CT, i.e., local excitation and partial CT have not been considered)

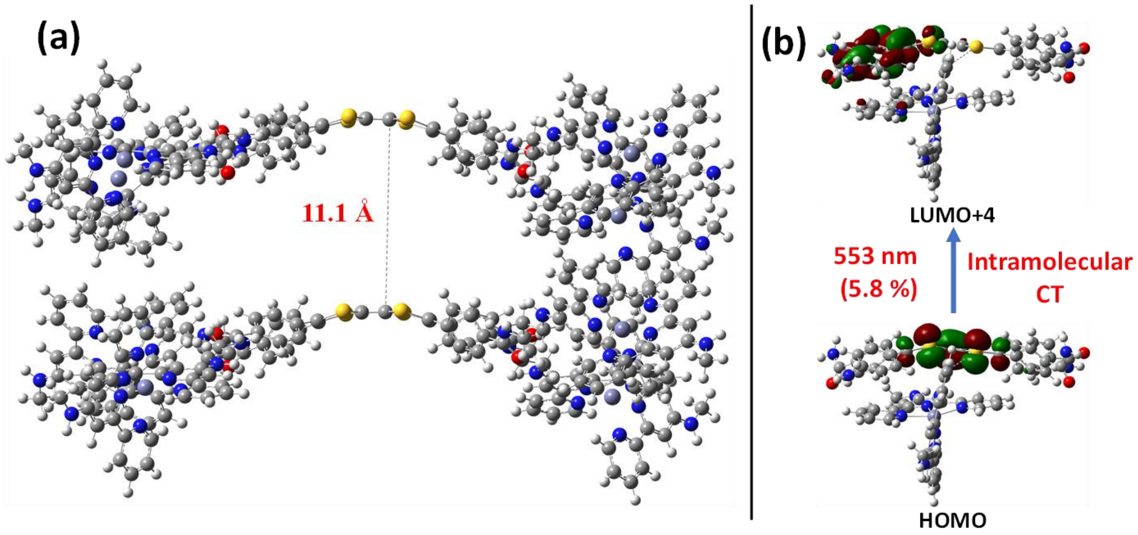

Supplementary Figure 22. (a) Optimized structure of TTF---TTF stacked model in case of **Zn-TPY-TTF CPG** (shows the distance between TTF---TTF unit was found to be >11 Å due to steric repulsion among the surrounding $[Zn(TPY)_2]^{2+}$ units attached to TTF core which rules out the possibility of π-π stacking between the TTF---TTF units). (b) Optimized structure and corresponding contributions in intramolecular charge transfer (CT) transitions for **Zn-TPY-TTF CPG.**

S4. Electrochemical characterizations of photocatalyst.

Mott-Schottky measurement details. The results were fitted by Eq. 1.[3]

$1/C^2 = (2/\varepsilon\varepsilon_0 A^2 e N_D)(V-V_{fb} - k_B T/e)$- Eq.1

Where C and A are the interfacial capacitance and area, respectively. ε is the dielectric constant of the semiconductor, and ε_0 is the permittivity of free space. k_B Boltzmann constant, *T* the absolute temperature, and *e* is the electronic charge. N_D the number of donors, *V* the applied voltage. Therefore, a plot of $1/C^2$ against *V* should yield a straight line from which V_{fb} can be determined from the intercept on the *V* axis.

Calculation of Conduction Band (E_{CB}) and Valence Band (E_{VB}) versus RHE (at pH=7). The conduction band edge (CB_{Edge}) potentials for TPY-TTF OG and Zn-TPY-TTF CPG were assigned based on flat band potentials, and valence band (VB) position was calculated by using equation 2.

$E_{VB} = E_{CB\ Edge}$ + Band Gap -Eq. 2

(i) For TPY-TTF OG

$E_{CB\ Edge\ (\mathbf{TPY\text{-}TTF})} = V_{fb}$= -0.60 V

$E_{VB\ (\mathbf{TPY\text{-}TTF})}$ = -0.60 + Band Gap $_{(\mathbf{TPY\text{-}TTF})}$ = -0.60 + 2.26= 1.66 V

(ii) Zn-TPY-TTF CPG

$E_{CB\ Edge\ (\mathbf{Zn\text{-}TPY\text{-}TTF})} = V_{fb}$= -0.54 V

$E_{VB\ (\mathbf{Zn\text{-}TPY\text{-}TTF})}$ = -0.54 + Band Gap $_{(\mathbf{Zn\text{-}TPY\text{-}TTF})}$ = -0.54 + 2.27= 1.73 V

S5. Photocatalytic water reduction experiments.

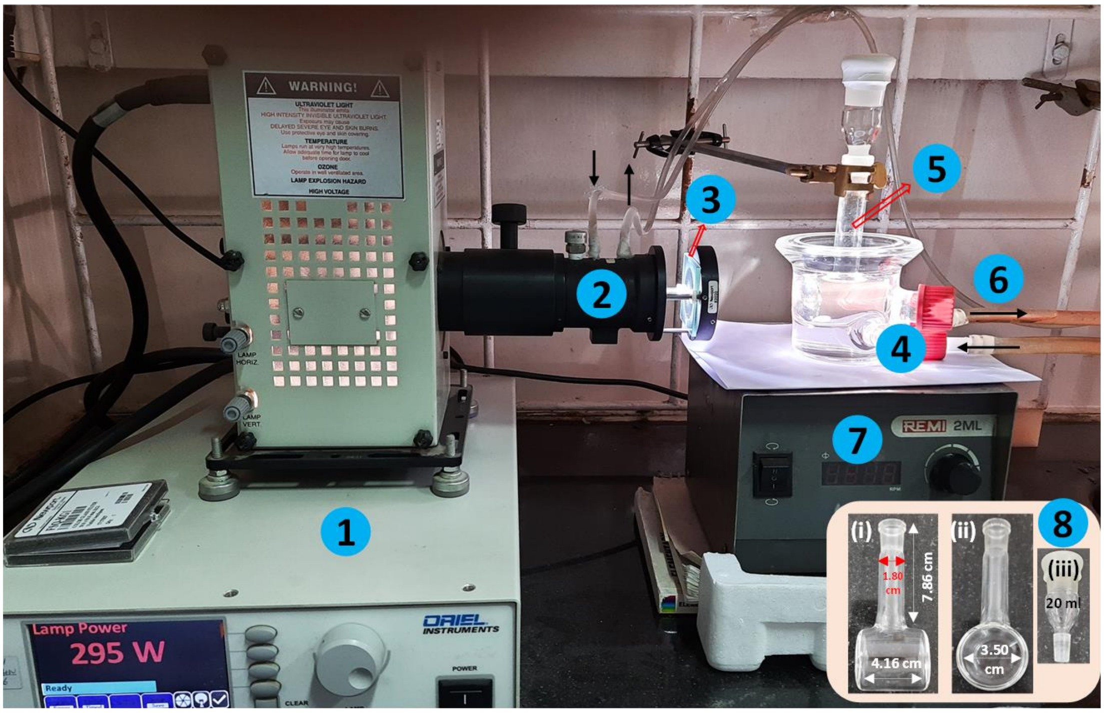

Supplementary Figure 23. Picture of setup used for controlled photocatalysis under laboratory condition; (1) Xenon lamp (Newport 300 W), (2) IR filter with cold water circulation, (3) Visible light band pass filter, (4) Cooling vessel, (5) Photocatalytic reaction cell, (6) Water circulation to maintain reaction temperature, (7) Magnetic stirrer, (8) Disassembled setup of photocatalytic cell with details; (i) front view, (ii) side view, (iii) adapter with air tight silicon rubber septum.

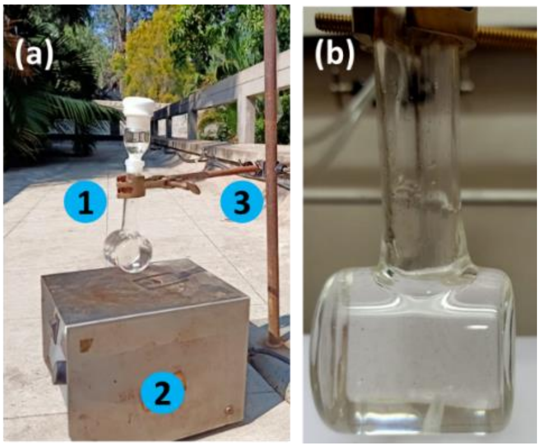

Supplementary Figure 24. (a) Setup used for photocatalysis under sunlight irradiation; 1. Photocatalytic cell with sample dispersion. 2. Magnetic stirrer. 3. Clamp with stand. (b) picture of sample (Zn-TPY-TTF CPG xerogel) dispersion at high magnification.

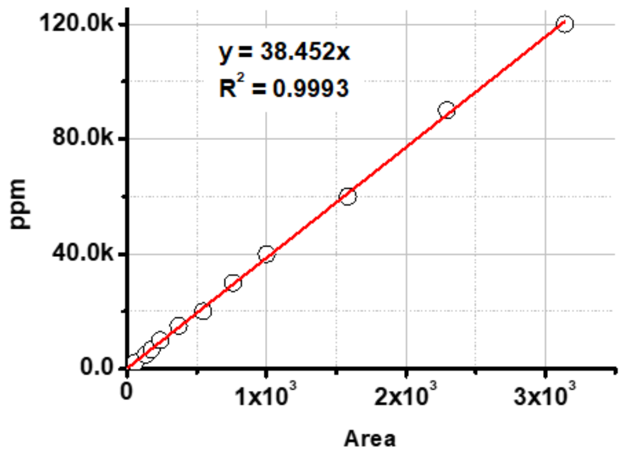

Supplementary Figure 25. Calibration plot for H_2. (This calibration was performed for different concentrations in Agilent GC-CN15343150 instrument; Nitrogen used as a carrier gas, and a thermal conductivity detector (TCD) was used for H_2 detection).

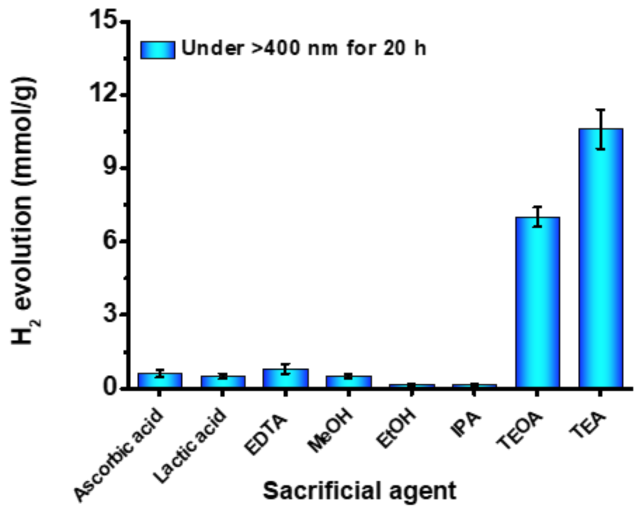

Supplementary Figure 26. Photocatalysis using **Zn-TPY-TTF CPG** in xerogel state under visible light irradiation for water reduction in the presence of equimolar concentrations (0.35 mol L^{-1}) of different sacrificial agents.

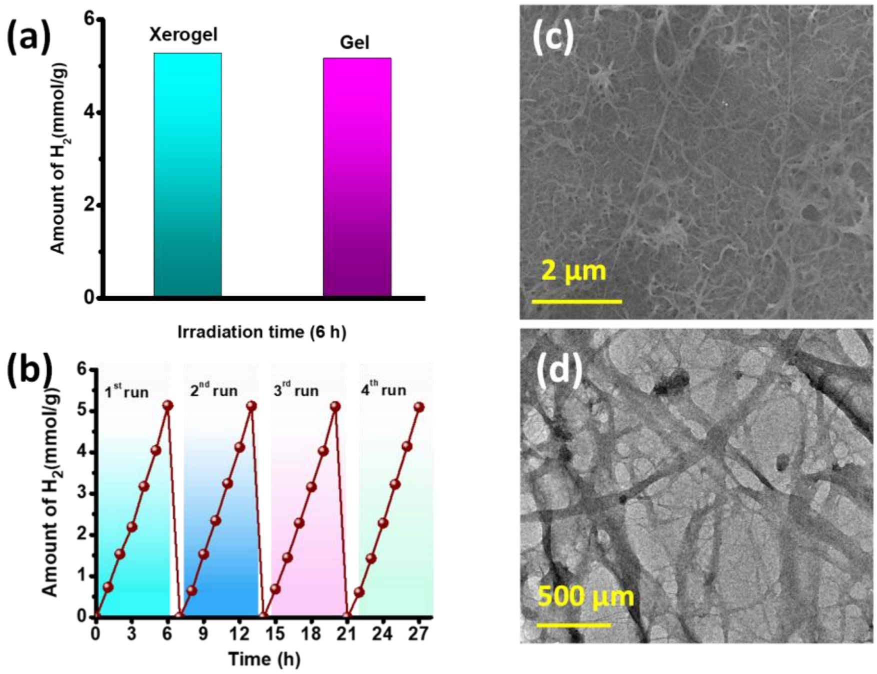

Supplementary Figure 27. (a) Photocatalysis by **Zn-TPY-TTF CPG** in xerogel and gel state. (Dispersion of the gel sample was ensured prior to the experiment). (b) Recyclability test for **Zn-TPY-TTF CPG** xerogel under visible light irradiation for water reduction (the photocatalyst was recovered and reused for next cycle under similar conditions). (c) FE-SEM image and (d) TEM image of **Zn-TPY-TTF CPG** after photocatalytic water reduction.

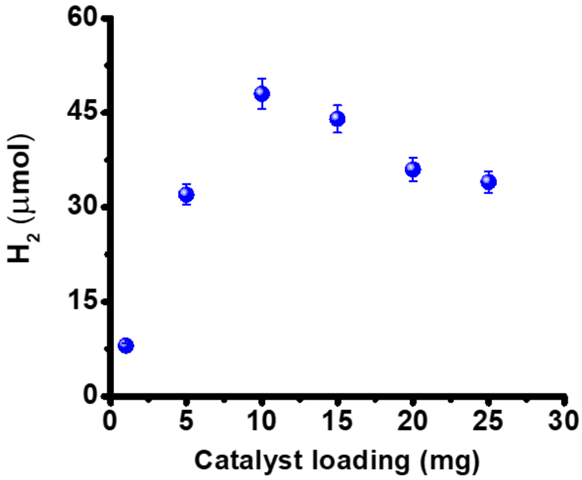

Supplementary Figure 28. Effect of **Zn-TPY-TTF CPG** loading (in mg) for photocatalytic water reduction under visible light irradiation for 10 h.

S6. Control experiments.

S6. (i) Photocatalytic activity for different structural units.

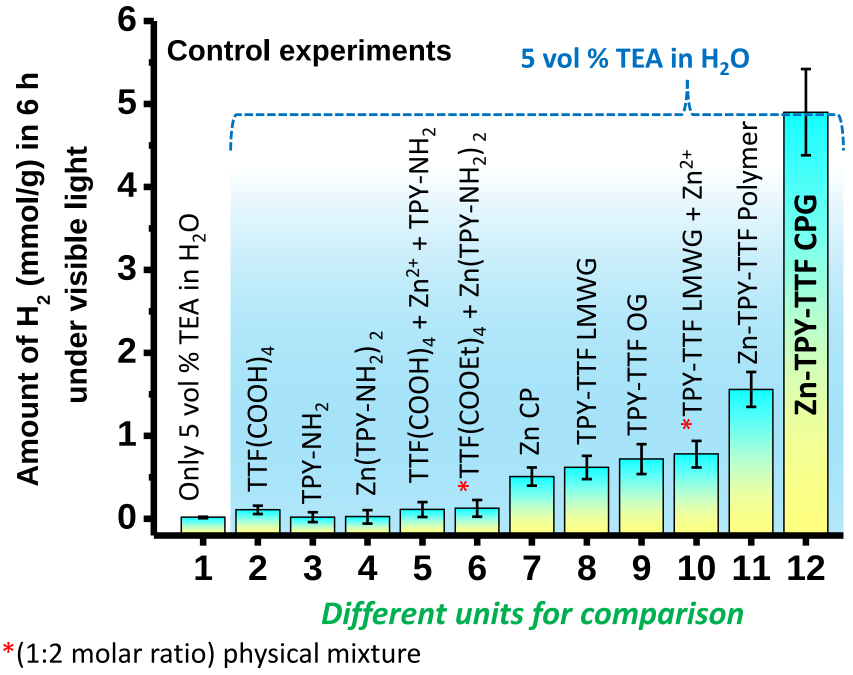

Supplementary Figure 29. TON for only triethylamine (**TEA**), **TTF(COOH)₄**, **TPY-TTF OG**, **Zn-TPY-TTF** (2:1 ratio) polymer, and **Zn-TPY-TTF CPG** (2:1 ratio) under visible light irradiation for 6 h.

S6. (ii) Synthesis of Zn-TPY-TTF (2:1) Polymer. We have synthesized Zn-TPY-TTF polymer using TPY-TTF LMWG and metal salt in the ratio of 1:2. TPY-TTF (10 mg, 5 μmol) was taken in 2 ml MeOH, and 500 μmol of Zn^{II} in MeOH was added to the TPY-TTF LMWG solution. The reaction mixture was stirred in a close vial for 2 h at 75°C, which resulted in a dark red precipitate.

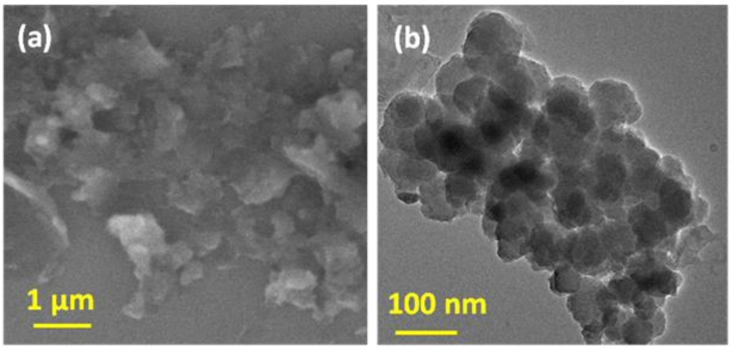

Supplementary Figure 30. (a) FE-SEM image, (b) TEM of **Zn-TPY-TTF polymer.**

S6. (iii) Synthesis and characterization of Zinc-TTF-TA (TTF-TA= TTF-Tetraacid; TTF(COOH)$_4$) Coordination Polymer (Zn-CP). Zn-TTF-TA coordination polymer was prepared based on a reported procedure in the literature.[4] In brief, TTF(COOH)$_4$ (100 mg, 0.146 mmol) was taken in a mixture of 12 ml dimethyl formamide (DMF) and 4 ml ethanol (EtOH). 8 ml aqueous solution of $Zn(NO_3)_2.6H_2O$ (0.5 mmol) was added into it, and the reaction mixture was heated at 75°C for 72 h in a closed vial and resulted into brown powder. The obtained precipitate was washed with DMF and EtOH for three times. The obtained product Zn-CP was dried under vacuum at 120°C for 2 h, and yield was found to be 78 mg. The thermal stability was analysed by TGA, which showed stability upto ~398°C.

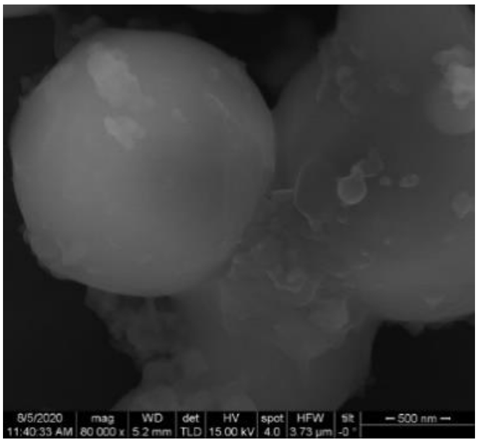

Supplementary Figure 31. FE-SEM image for **Zn-CP**.

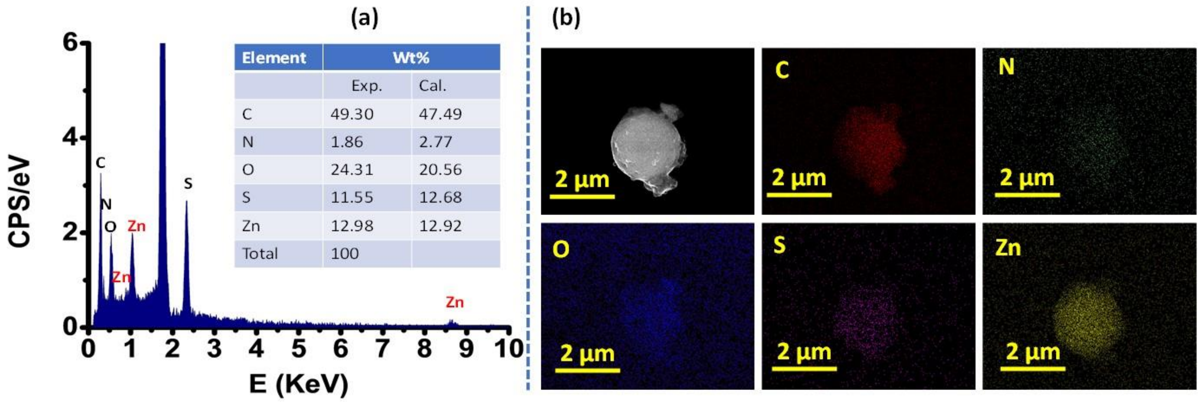

Element	Wt%	
	Exp.	Cal.
C	49.30	47.49
N	1.86	2.77
O	24.31	20.56
S	11.55	12.68
Zn	12.98	12.92
Total	100	

Supplementary Figure 32. (a) EDAX and (b) elemental mapping for **Zn-CP**.

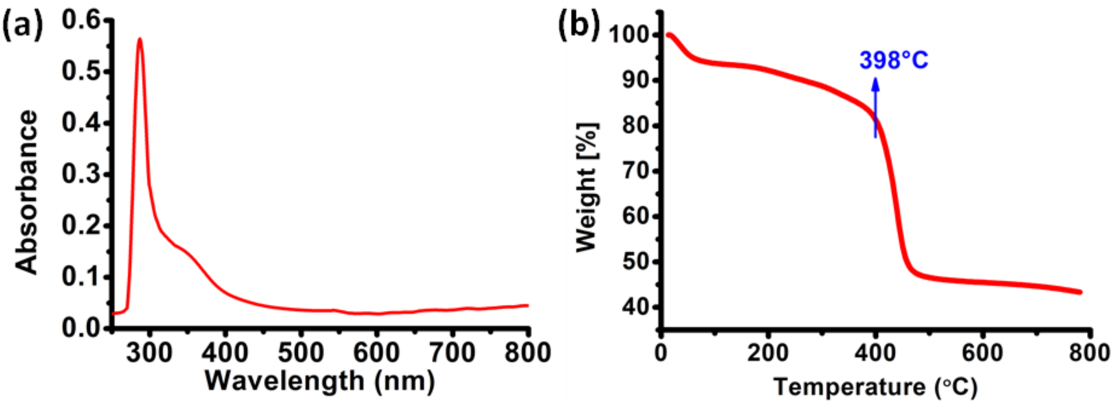

Supplementary Figure 33. (a) UV-Visible absorption spectrum for **Zn-CP** (thin film on quartz plate). (b) TGA plot for **Zn-CP** under N_2 atmosphere.

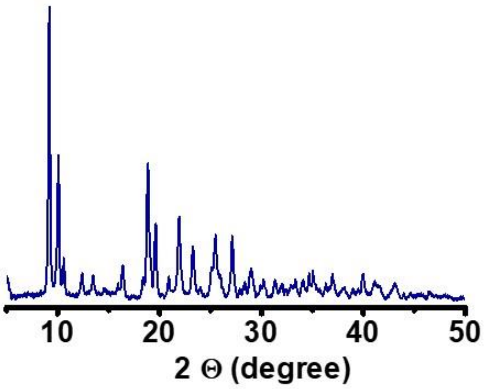

Supplementary Figure 34. PXRD for **Zn-CP** (as synthesized).

S7. Synthesis and characterization of Pt@Zn-TPY-TTF CPG.

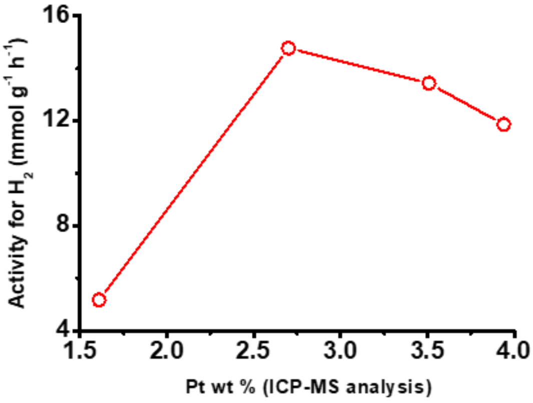

Supplementary Figure 35. Pt loading optimization in **Pt@Zn-TPY-TTF CPG** and photocatalytic activity toward H_2 production in different loading of Pt NP; 1.61 wt %; 2.70 wt%, 3.51 wt%, 3.94 wt% upon **Zn-TPY-TTF CPG**.

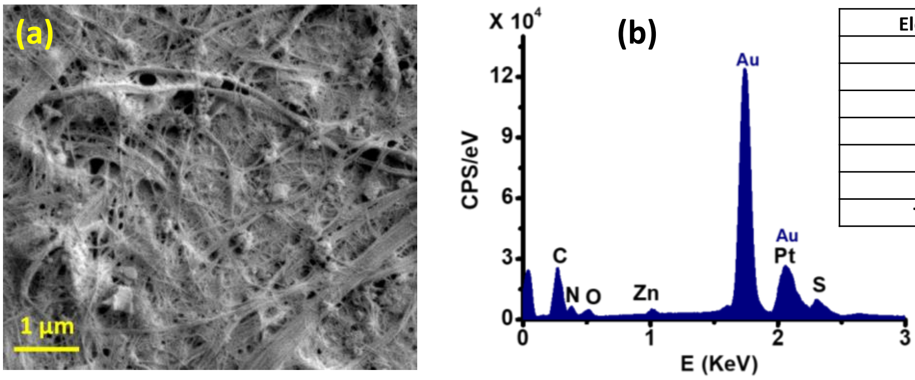

Element	Exp. Wt%
C	57.98
N	16.85
O	11.48
S	5.56
Zn	5.24
Pt	2.89
Total	100

Supplementary Figure 36. (a) FE-SEM image and (b) EDAX analysis for **Pt@Zn-TPY-TTF CPG**.

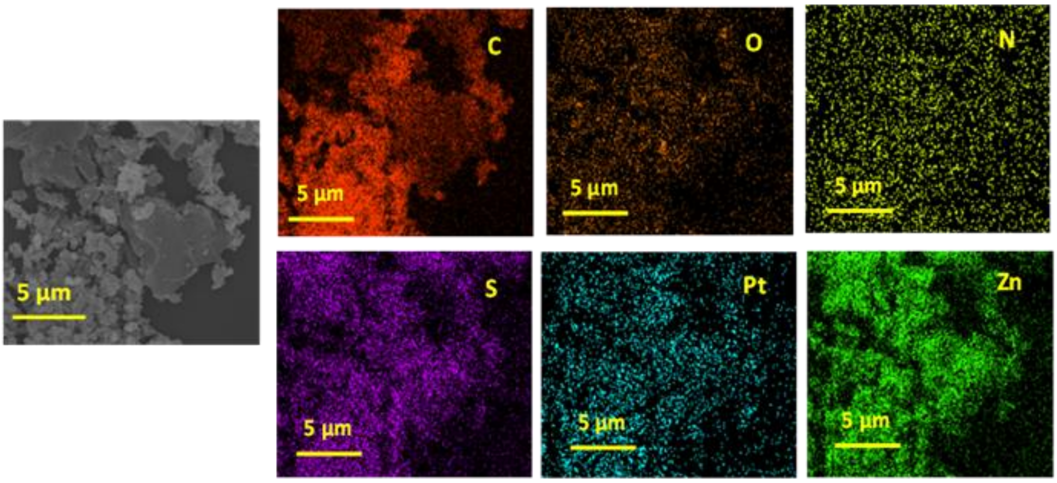

Supplementary Figure 37. Elemental mapping for **Pt@Zn-TPY-TTF CPG**.

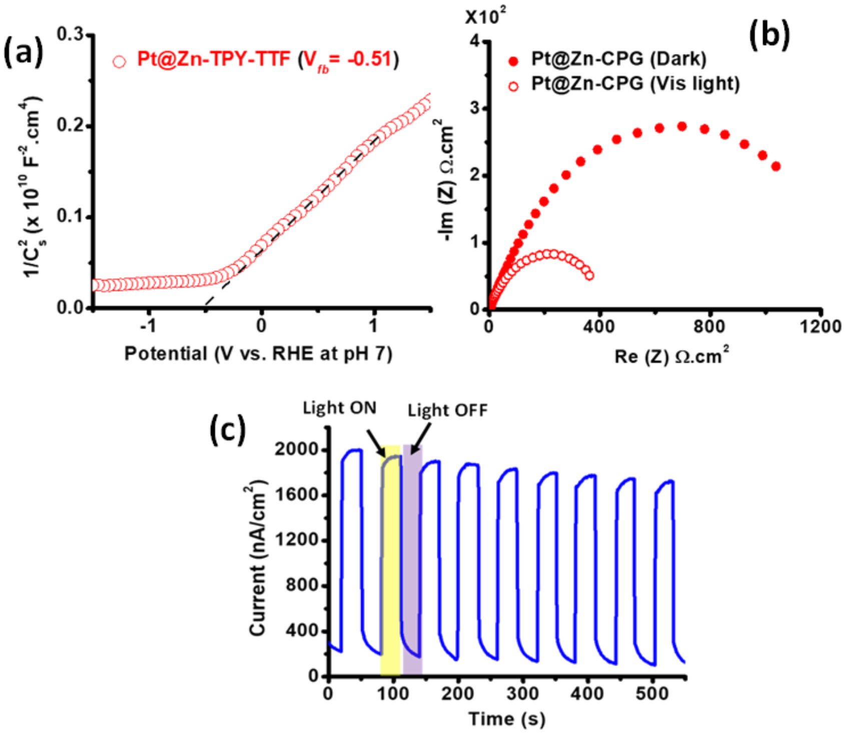

Supplementary Figure 38. (a) Mott-Schottky plot for **Pt@Zn-TPY-TTF CPG** (at 1000 Hz, from -1.5 V to + 1.5 V). (b) Nyquist plots for **Pt@Zn-TPY-TTF CPG** under visible light and the dark condition at -1.20 V_{RHE} applied bias (in 0.5 M Na_2SO_4). (c) Photocurrent study of **Pt@Zn-TPY-TTF CPG** based on light ON-OFF cycles.

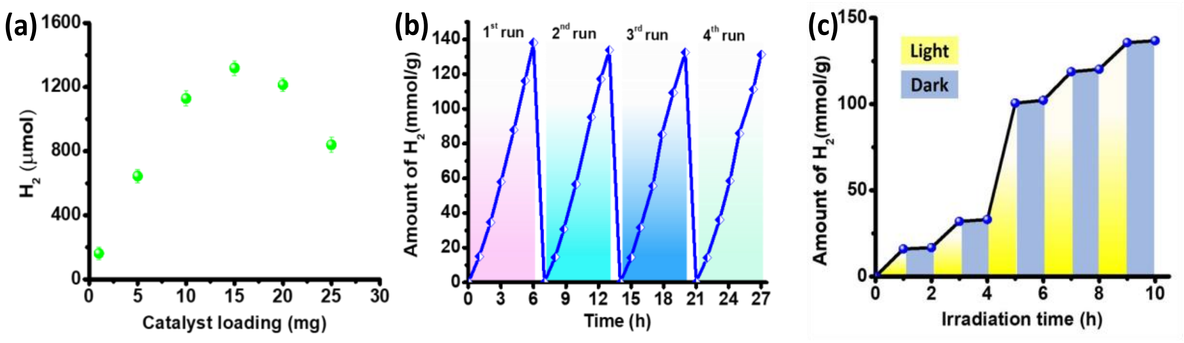

Supplementary Figure 39. (a) Effect of **Pt@Zn-TPY-TTF CPG** loading (in mg) for photocatalytic water reduction under visible light irradiation for 10 h. (b) Recyclability test for **Pt@Zn-TPY-TTF CPG** xerogel under visible light irradiation for water reduction (the photocatalyst was recovered and reused for next cycle under similar conditions). (c) Light On-Off experiments for visible light-driven H_2 formation during photocatalytic water reduction by **Pt@Zn-TPY-TTF CPG**.

S8. Calculation for turnover number (TON).

Supplementary Table 3. Calculation of TON and TOF (h^{-1}) w.r.t. to Pt loading of 2.7 wt% in **Pt@Zn-TPY-TTF CPG** photocatalyst.

**2.7 wt% Pt loading**	**Amount in mmol/g**	**Amount in µmol from 1 mg**	**Pt amount in 1mg of sample**	**TON**
**Under artificial light (Xenon lamp: visible range)**				
H_2 in 11 h	162.42	162.42 µmol	0.138 µmol	1176.9
CH_4 in 30 h	8.75	8.75 µmol	0.138 µmol	63.4
**Under sunlight (for highest amount)**				
H_2 in 6 h (27th June 2019)	72	72	0.138 µmol	521.7
CH_4 in 6 h (1st Oct 2019)	0.96	0.96	0.138 µmol	6.9

S9. Study of Time resolved photoluminescence (TRPL).

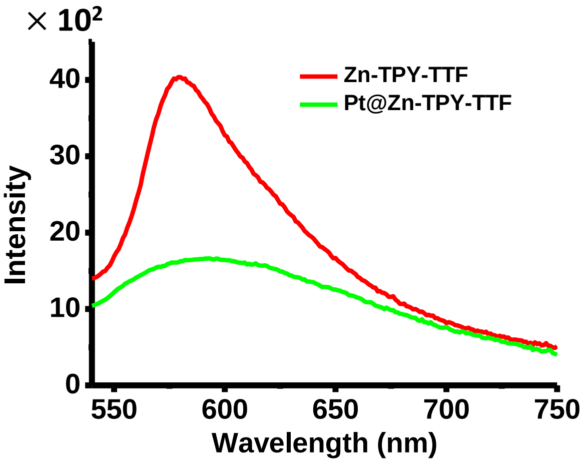

Supplementary Figure 40. PL spectra for **Zn-TPY-TTF CPG** and **Pt@Zn-TPY-TTF CPG**. (excitation wavelength was 510 nm; corresponding to CT band of the catalyst; emission maxima was observed at 581 nm).

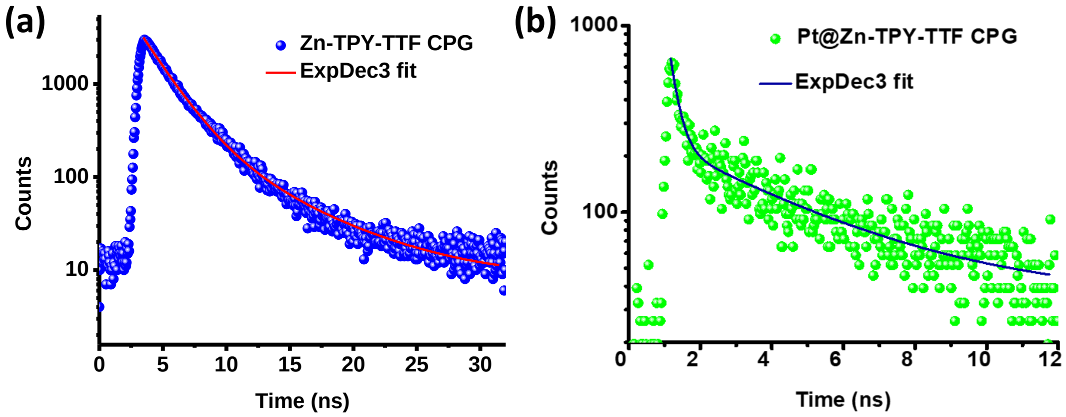

Supplementary Figure 41. TRPL decay spectra (a) For **Zn-TPY-TTF CPG**. (b) For **Pt@Zn-TPY-TTF CPG**. (510 nm laser used for excitation; decay was collected at 581 nm).

Supplementary Table 4. Average lifetime calculation.

CPG Samples	τ_1 (ns)	A1	τ_2 (ns)	A2	τ_3 (ns)	A3	τ_{av} (ns)
**Zn-TPY-TTF**	1.95	53.66%	1.95	42.31%	6.01	4.03%	**1.95**
**Pt@Zn-TPY-TTF**	0.22	42.74%	0.22	49.64%	3.82	7.62%	**0.22**

S10. Study of transient absorption.

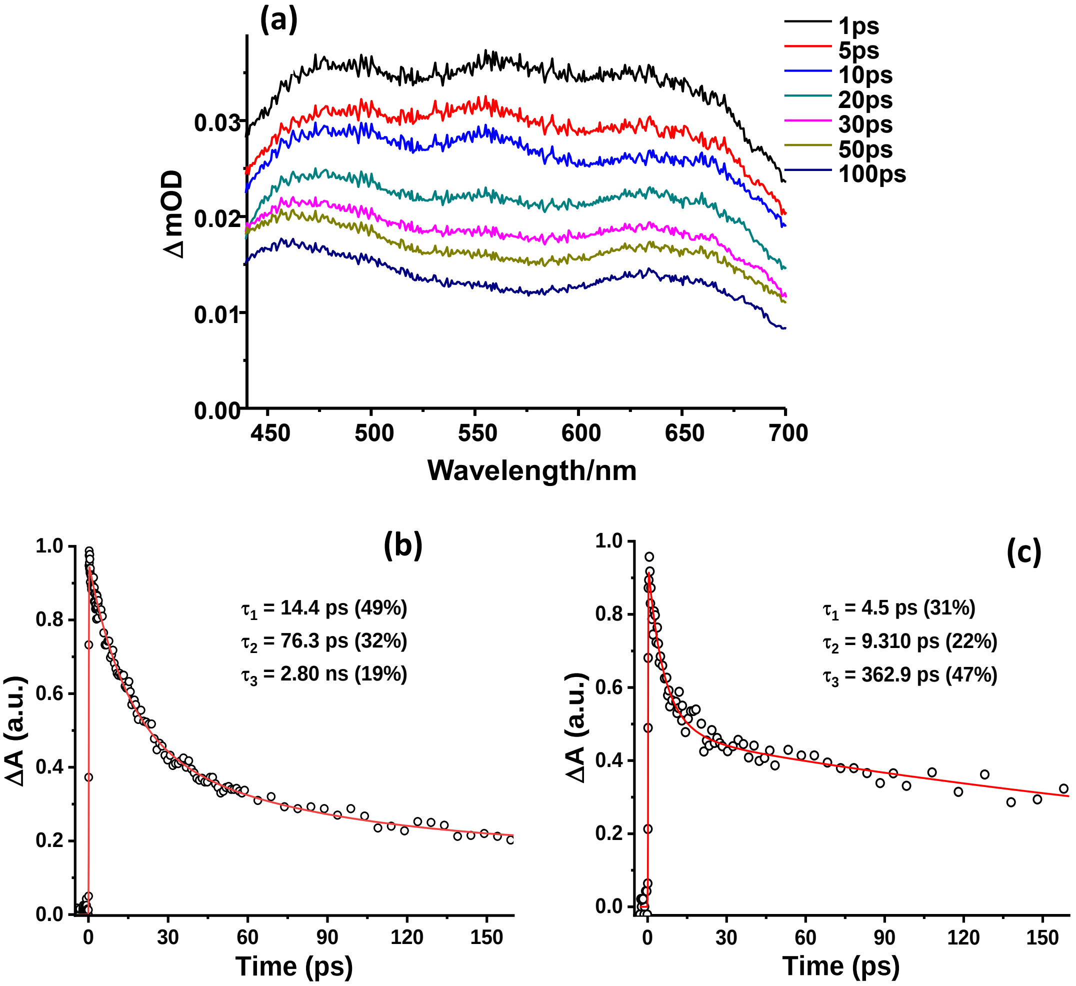

Supplementary Figure 42. (a) Transient absorption spectra of Zn-TPY-TTF CPG dispersed in methanol at different time delays (1-100 ps). Transient decay traces at 570 nm for Zn-TPY-TTF CPG (b) and Pt@Zn-TPY-TTF CPG (c). Solid lines in panels B and C are fitted curves.

S11. Study of Pt NPs stabilization through DFT calculations.

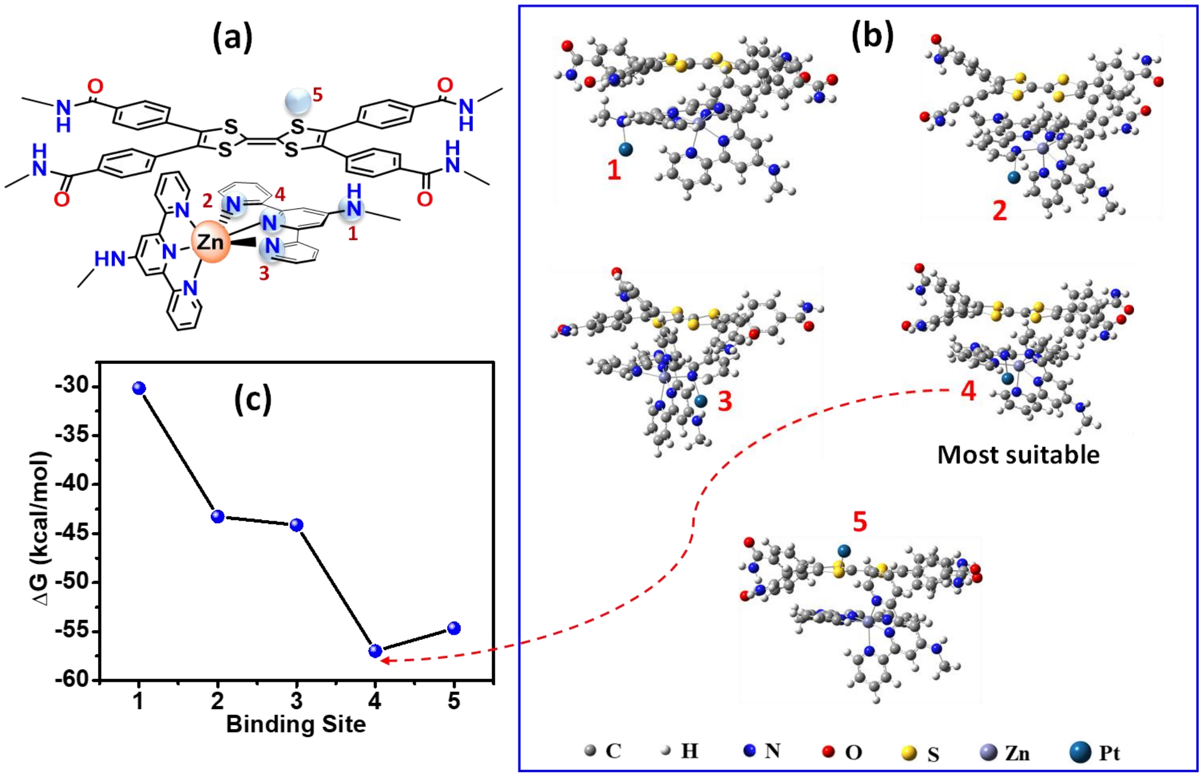

Supplementary Figure 43. (a) Schematic of the structure of TTF and $[Zn(TPY)_2]^{2+}$ stack system for possible loading positions of Pt nanoparticles. (b) Different optimized models were obtained from DFT calculations to illustrate the possible loading position of Pt on TTF and $[Zn(TPY)_2]^{2+}$ stack in **Zn-TPY-TTF CPG** system. (c) Stabilization energies of Pt in kcal/mol corresponding to loading positions. The most suitable loading position was observed in close proximity to 4th 'N' atom of the central pyridine ring of horizontal terpyridine unit.

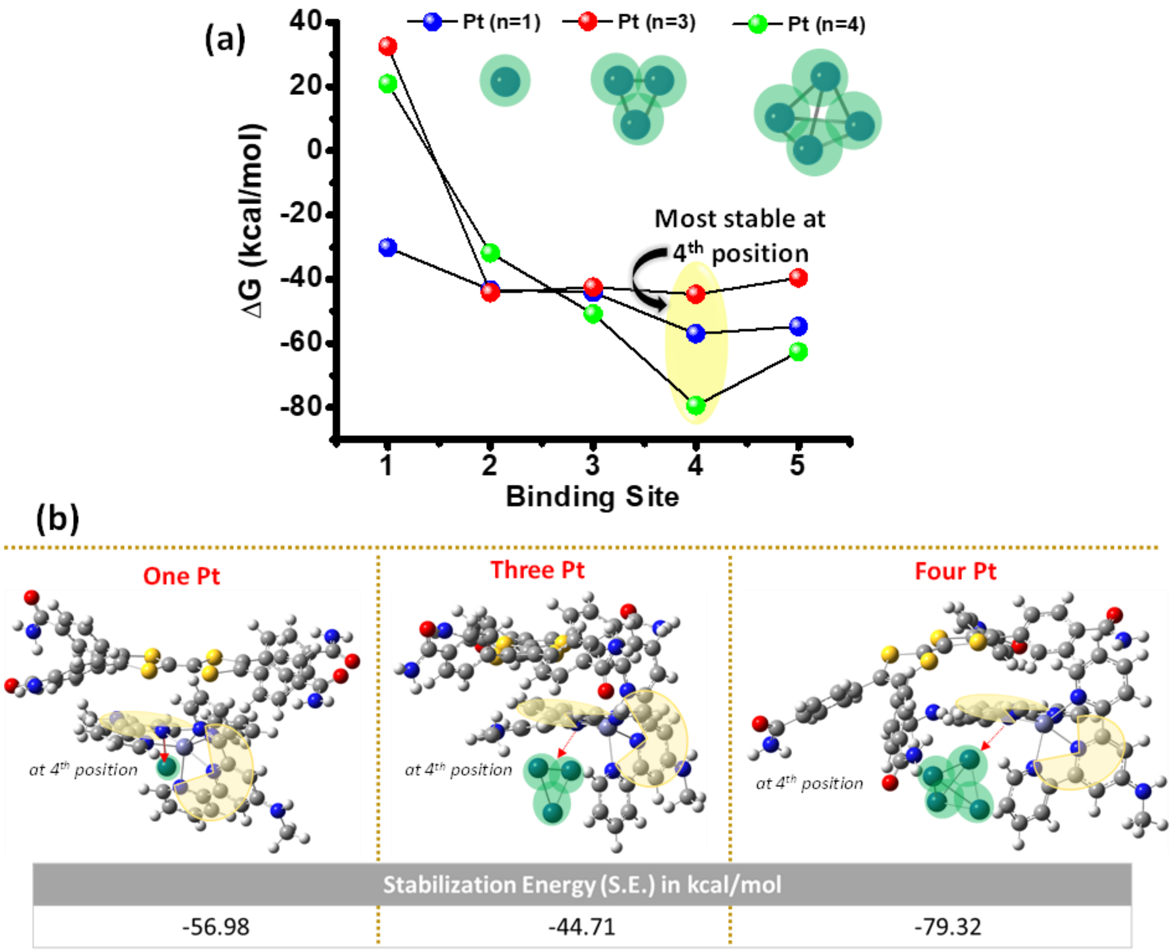

Supplementary Figure 44. (a) Stabilization energies of Pt in kcal/mol corresponding to loading positions. (b) Different optimized models were obtained from DFT calculations to illustrate the possible loading position of different Pt models on TTF and $[Zn(TPY)_2]^{2+}$ stack in **Zn-TPY-TTF CPG** system and corresponding stabilization energies in kcal/mol. The extensive theoretical studies clarified that the most suitable position for stabilization of Pt NPs is irrespective of the cluster size and found to be similar as observed for a single Pt atom.

S12. Apparent Quantum efficiency (AQE) measurement for H_2 production. The detailed calculation for H_2 production in case of Pt@Zn-TPY-TTF CPG is given in Supplementary Table 5.

Supplementary Table 5. For details of apparent quantum efficiency calculation for hydrogen production from Pt@Zn-TPY-TTF CPG photocatalyst.

**λ (nm)**	**Total H_2 from 1 mg of catalyst in 1 h (μmol)**	***No. of H_2 molecules[a] (×10^{18})***	**Power at λ (mW s^{-1} cm^{-2})**	**Total Power[b] in 1h (mW)**	**No. of photons[c] *(×10^{18})***	**AQE[d]= [(No. of H_2×2)/No. of Photons] ×100**
400 ±10	0.78	***0.472***	0.166	5746.67	11.61	8.14 %
450±10	0.98	***0.595***	0.162	5608.19	12.74	9.35 %
500±10	1.28	***0.773***	0.143	4950.44	12.50	12.38 %
550±10	1.80	***1.085***	0.156	5400.48	15.00	14.47 %
600±10	2.06	***1.244***	0.198	6854.46	20.77	11.98 %
650±10	1.07	***0.646***	0.186	6439.04	21.14	6.12 %
700±10	0.39	***0.239***	0.142	4915.82	17.38	2.76

(a) From **X** μmol to no. of product conversion = **X** ×10^{-6} × N_A (6.023 × 10^{23})

(b) Total power used in 1 h in the provided area of photocatalytic cell

= **P** × 60 × 60 × πr^2 (mW); where **r** = 1.75 cm

(c) No. of Photons (n) falling on photocatalytic cell in 1 h

n= $\left[\frac{P\times\lambda}{hc}\right]$; where P=total power used (mW) and λ= wavelength (nm) and h= plank constant and c = speed of light; both h and c is constant, and 1 *hc* is equivalent to 1240 eV. nm, which can be written as 1.98 × 10^{-13} mW. nm

(d) Calculation of AQE% = [No. of electrons used for product formation/ No. of photons] × 100

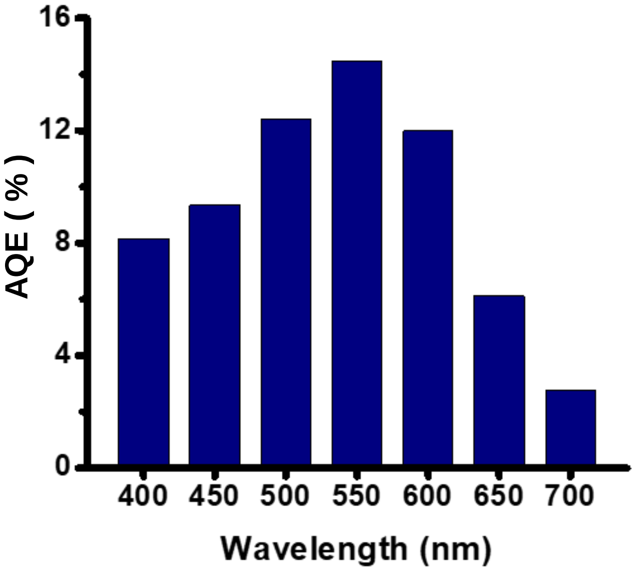

Supplementary Figure 45. AQE (%) for H_2 formation by **Pt@Zn-TPY-TTF CPG** at a different wavelength (400 nm to 700 nm) (for this experiment 1 mg of sample was dispersed in 12 ml H_2O with TEA (0.6 ml) as sacrificial donor).

S13. Comparison table for photocatalytic H_2 evolution.

Supplementary Table 6. Photocatalytic H_2 evolution activity of **TPY-TTF OG**, **Zn-TPY-TTF CPG,** and **Pt@Zn-TPY-TTF CPG** under visible light irradiation (from 400-750 nm).

Catalyst	Reaction medium	Amount of H_2 production (mmol/g)	Irradiation Time (h)	Activity (μmol g^{-1} h^{-1})	TON
TPY-TTF OG	H_2O/TEA	2.00 mmol/g	20 h	100 μmol g^{-1} h^{-1}	3.6 in 20 h
**Zn-TPY-TTF CPG**	H_2O/TEA	**10.60 mmol/g**	20 h	**530 μmol g^{-1} h^{-1}**	23.5 in 20 h
**Pt@Zn-TPY-TTF CPG**	H_2O/TEA	**162.42 mmol/g**	11 h	**14727 μmol g^{-1} h^{-1}**	1176.9 in 11 h (w.r.t. Pt)
Zn-TPY-TTF CPG	Only H_2O	0.92 mmol/g	20 h	46 μmol g^{-1} h^{-1}	2.0 in 20 h
Pt@Zn-TPY-TTF CPG	Only H_2O	2.93 mmol/g	6 h	488 μmol g^{-1} h^{-1}	21.23 in 6 h (w.r.t. Pt)

Supplementary Table 7. Photocatalytic H_2 evolution activity of **Zn-TPY-TTF CPG** and **Pt@Zn-TPY-TTF CPG** under direct sunlight irradiation.

Catalyst	Amount of H_2 evolution (mmol/g)	Irradiation Time (h)	Activity (μmol g^{-1} h^{-1})	TON
**Zn-TPY-TTF CPG**	5.14 mmol/g on 15th April	6 h (10 am to 4 pm)	857 μmol g^{-1} h^{-1}	11.9 in 6 h on 15th April 2019
**Pt@Zn-TPY-TTF CPG**	72.02 mmol/g on 27th June	6 h (10 am to 4 pm)	12000 μmol g^{-1} h^{-1}	521.8 in 6 h on 27th June (w.r.t. Pt)

Supplementary Table 8. Comparison table for H_2 evolution in case of **Zn-TPY-TTF CPG** and **Pt@Zn-TPY-TTF CPG** with some of the reported benchmark hybrid materials under visible light irradiation.

No.	Catalyst	Reaction medium	Activity for H_2	AQE%	ref
1.	**Zn-TPY-TTF CPG**	H_2O/ TEA	**530** μmol g^{-1} h^{-1}	0.76 % at 550 nm	**This work**
2.	**Pt@Zn-TPY-TTF CPG**	H_2O/ TEA	**14765** μmol g^{-1} h^{-1}	14.47 % at 550 nm	**This work**
3.	g-C_3N_4/ Pt Co-catalyst (urea-CN*x*)	H_2O/ MeOH	17 nmol h^{-1}	17.9 % at 400 nm	ref[5]
4.	NiFeSe]-hydrogenase (H_2ase)/ NiP+ CNx	H_2O/ TEOA	155 mol $(mol_{NiP})^{-1}$	0.005 % at 465 nm	ref[6]
5.	N_2-COF/ chloro(pyridine) cobaloxime co-catalyst	H_2O, acetonitrile/ TEOA	782 μmol g^{-1} h^{-1}	0.16% at 400 nm	ref[7]
6.	Thiazolo[5,4-d]thiazole-COF (TpDTz)+ Ni- cluster co-catalyst	H_2O/ TEOA	941 μmol g^{-1} h^{-1}	0.2% at 400 nm	ref[8]
7.	CN-ATZ-NaK/ 3wt% Pt NP	H_2O/ TEOA	630 μmol h^{-1}	0.65 at 420 nm	ref[9]

8.	FS-TEG	H_2O, MeOH/ TEA	2.9 mmol g^{-1} h^{-1}	10% at 420 nm	ref[10]
9.	dibenzo[*b,d*]thiophene sulfone-dibenzo[*b,d*]-thiophene co-polymer (P64)	H_2O, MeOH/ TEA	6038.5 µmol g^{-1} h^{-1}	20.7% at 420 nm	ref[11]
10.	27.5 wt % CdSe-loaded CdS / 1wt% Pt NP	H_2O/ Na_2S, Na_2SO_3	16353 µmol g^{-1} after 4 h	NP	ref[12]
11.	ZnSe Nanorods/ Ni-$(BF_4)_2$ co-catalyst	H_2O/ ascorbic acid	54 mmol $g_{ZnSe}^{-1}h^{-1}$	50% at 400 nm	ref[13]
12.	Tetraphenylethylene (TPE) Based CMP	H_2O/ MeOH/ Na_2S, Na_2SO_4	659.55 µmol g^{-1} h^{-1}	NP	ref[14]
13.	$^{NCN}CN_x$ / NiP	Aq. K_2HPO_4/ 4-methyl benzyl alcohol	0.061 µmol h^{-1}	NP	ref[15]
14.	C_3N_4 based gel (FD-CNB-G)/3 wt% Pt NP	H_2O/ TEOA	20 µmol	NP	ref[16]
15.	PTCDIs- C_3N_4 aggregates/ Pt NP	H_2O/ TEOA	3.8 µmol h^{-1}	0.31 % at 420 nm	ref[17]
16.	nano-TiO_2/ CdSe quantum dots/ Pt co-catalyst	H_2O /EtOH	16.7 mmol g^{-1} h^{-1}	NP	ref[18]
17.	Ir-complex (P1)/ $Co(bpy)_3Cl_2$	H_2O, Acetone/ TEOA	598 µmol	1.02% at 420 nm	ref[19]
18.	Cu-complex	H_2O, THF/ TEA	11.78 µmol g^{-1} h^{-1}	NP	ref[20]
19.	PBI-F gel/ 1 mol % PVP capped Pt	H_2O/ MeOH	3.0 µmol g^{-1} h^{-1}	0.018% at 365 nm	ref[21]
20.	Ni (1%): TiO_2	H_2O/ MeOH	3390 µmol g^{-1} h^{-1}	2.8 %	ref[22]

(* NP=Not Produced)

S14. Photocatalytic CO_2 reduction.

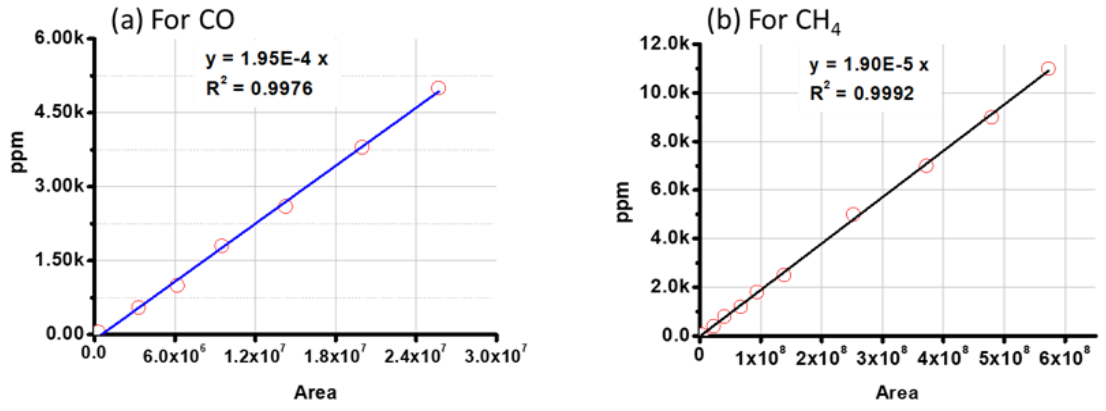

Supplementary Figure 46. Calibration plot for (a) CO and (b) CH_4. (This calibration was performed for different concentrations in Shimadzu GC-MS-QP2020; Argon was used as carrier gas).

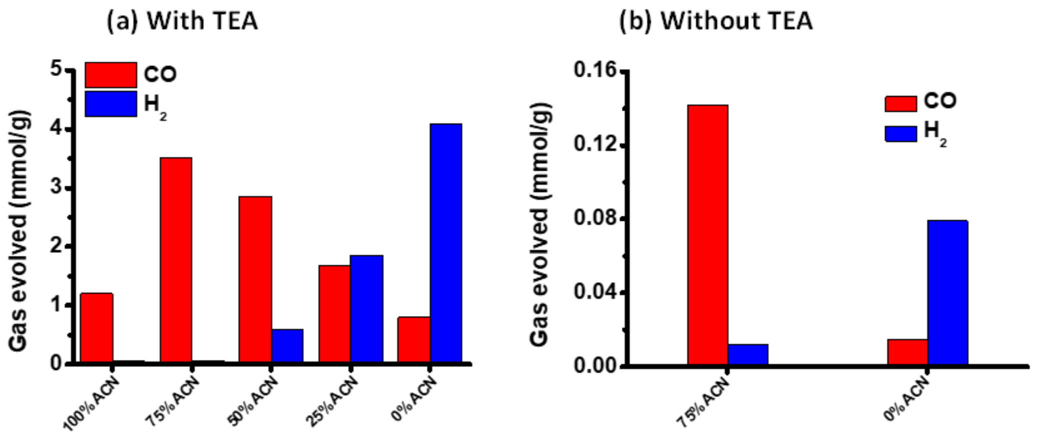

Supplementary Figure 47. Product distribution during the photocatalytic CO_2 reduction using **Zn-TPY-TTF CPG** in a different composition of acetonitrile/water as solvent under CO_2 atmosphere; 8 h visible light irradiation. (a) With TEA. (b) Without TEA.

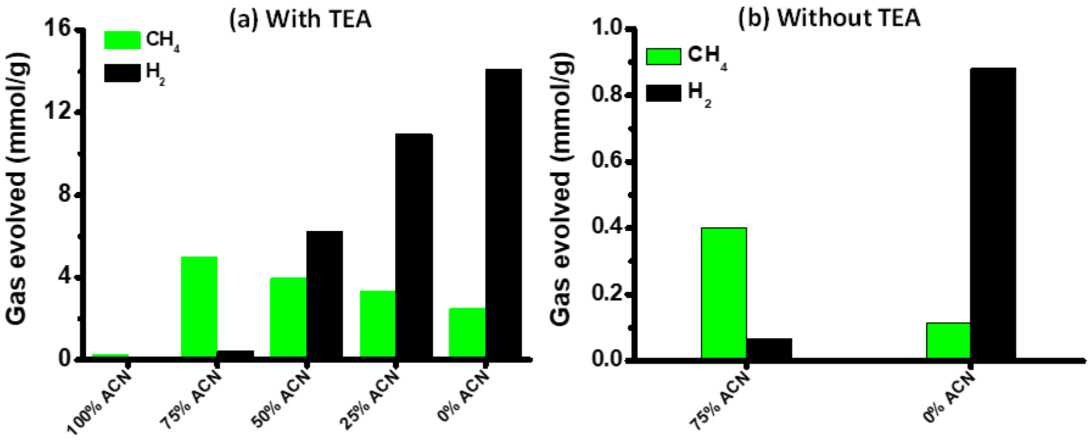

Supplementary Figure 48. Different compositions of acetonitrile/water for CO_2 reduction using **Pt@Zn-TPY-TTF CPG** under visible light irradiation for 12 h. (a) With TEA. (b) Without TEA.

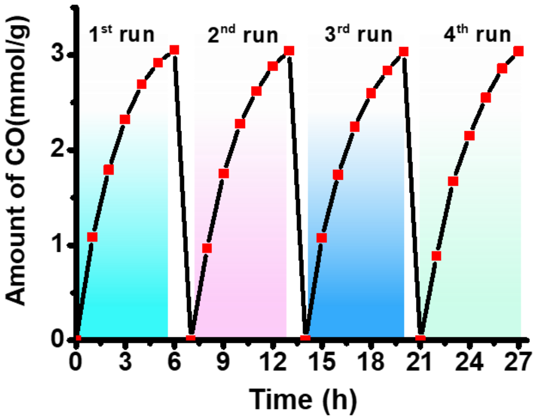

Supplementary Figure 49. Recyclability test for **Zn-TPY-TTF CPG** xerogel under visible light irradiation for CO_2 reduction to CO. (The photocatalyst was recovered and reused after each cycle under similar conditions).

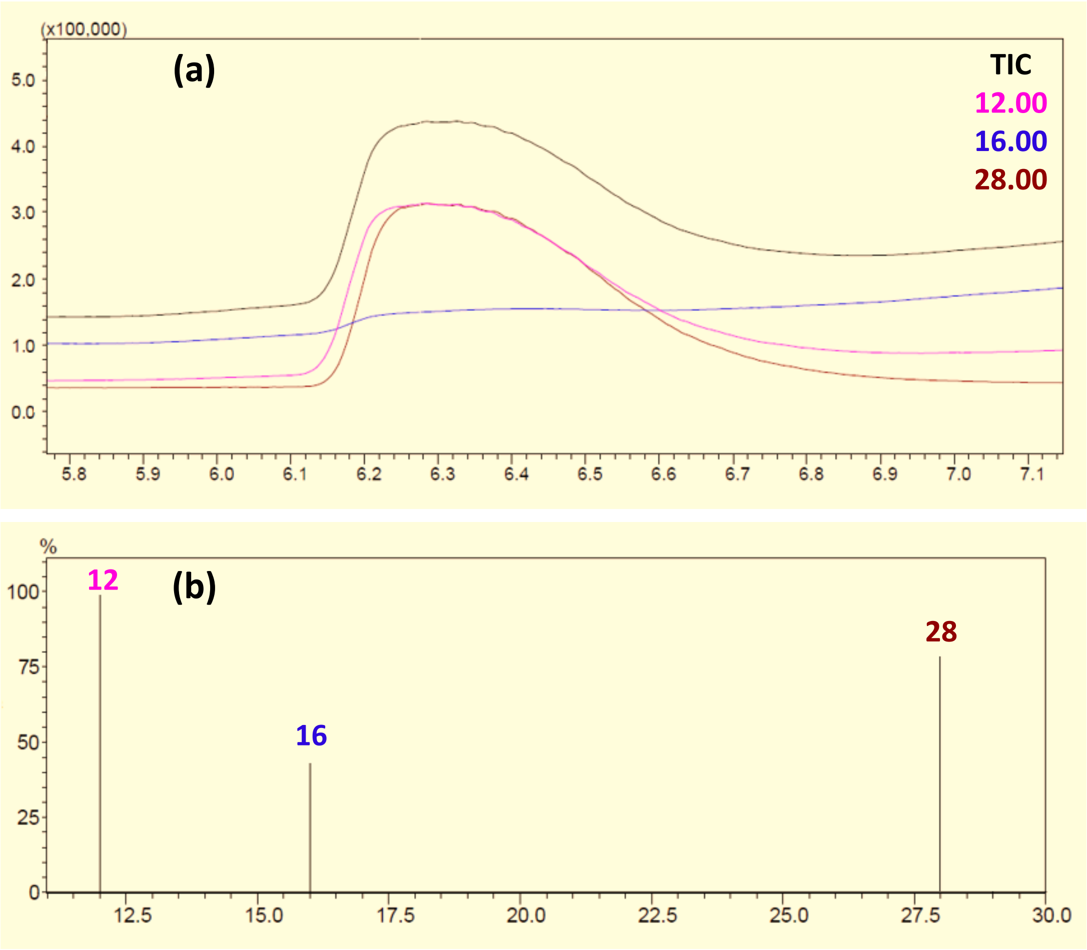

Supplementary Figure 50. (a) Chromatogram and (b) Mass analysis for ^{12}**CO** as a product obtained from $^{12}CO_2$ saturated sample using **Zn-TPY-TTF CPG** under visible light irradiation for 1 h (for this experiment, $^{12}CO_2$ gas was purged for 20 min in 10 ml solvent mixture of CH_3CN: H_2O containing 1 mg of catalyst and 0.5 ml TEA).

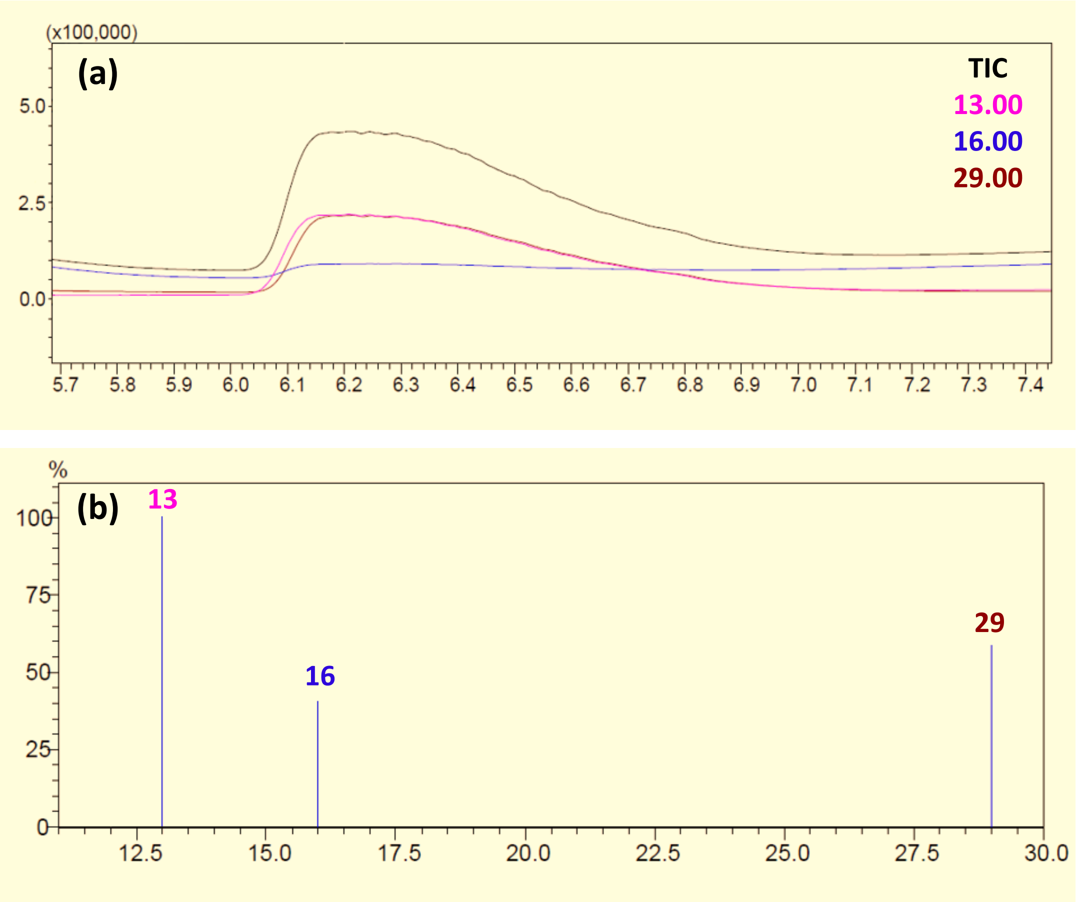

Supplementary Figure 51. (a) Chromatogram and (b) Mass analysis for **^{13}CO** as a product obtained from $^{13}CO_2$ saturated sample using **Zn-TPY-TTF CPG** under visible light irradiation for 1 h (for this experiment, $^{13}CO_2$ gas was purged for 10 min in 10 ml solvent mixture of CH_3CN: H_2O containing 1 mg of catalyst and 0.5 ml TEA).

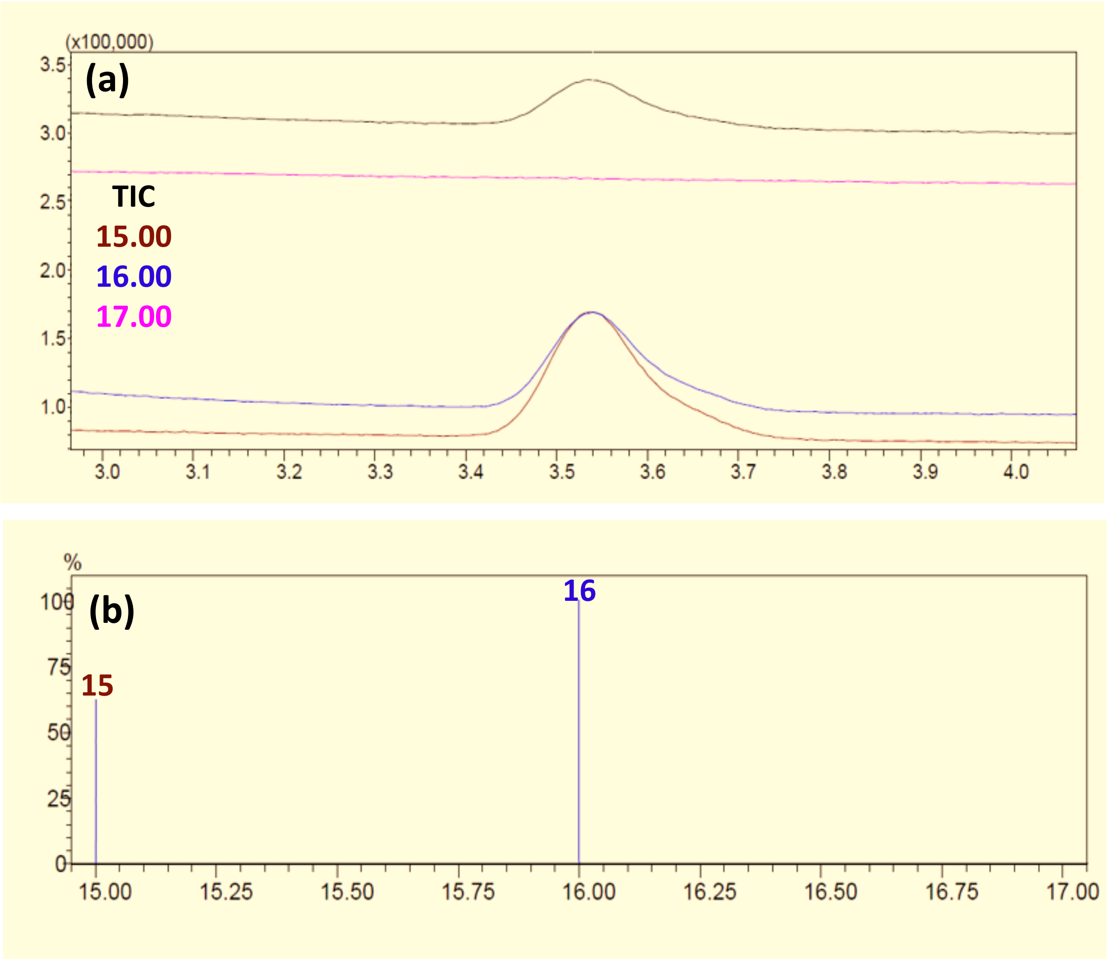

Supplementary Figure 52. (a) Chromatogram and (b) Mass analysis for **$^{12}CH_4$** as a product obtained from $^{12}CO_2$ saturated sample using **Pt@Zn-TPY-TTF CPG** under visible light irradiation for 1 h (for this experiment, $^{12}CO_2$ gas was purged for 10 min in 10 ml solvent mixture of CH_3CN: H_2O containing 1 mg of catalyst and 0.5 ml TEA).

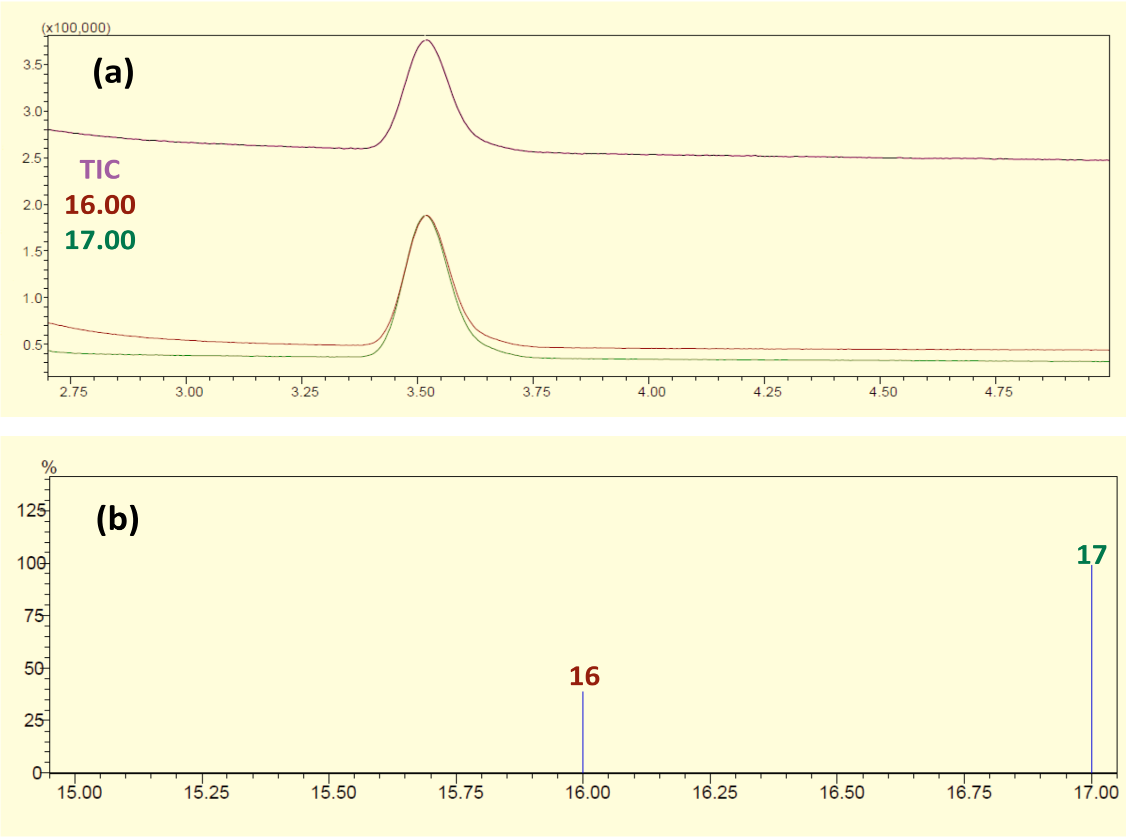

Supplementary Figure. 53. (a) Chromatogram and (b) Mass analysis for **$^{13}CH_4$** as a product obtained from $^{13}CO_2$ saturated sample using **Pt@Zn-TPY-TTF CPG** under visible light irradiation for 1 h (for this experiment, $^{13}CO_2$ gas was purged for 10 min in 10 ml solvent mixture of CH_3CN: H_2O containing 1 mg of catalyst and 0.5 ml TEA).

S15. Quantum efficiency measurements for CH_4 formation in case of Pt@Zn-TPY-TTF CPG.

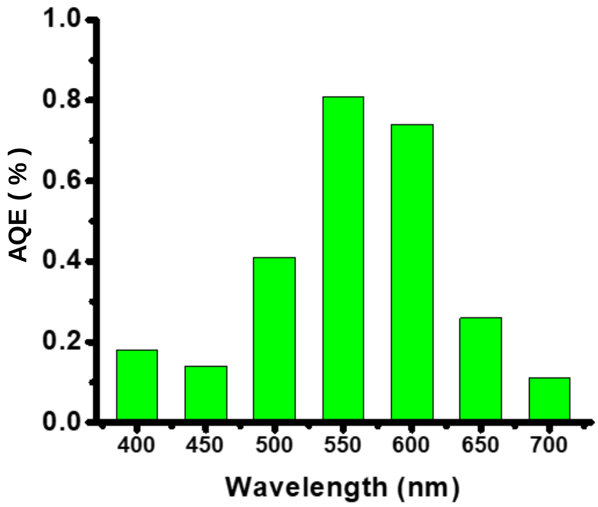

Supplementary Figure 54. AQE (%) for CH_4 formation by **Pt@Zn-TPY-TTF CPG** at a different wavelength (400 nm to 700 nm) (for this experiment 1 mg of sample was dispersed in CO_2 saturated 12 ml solvent mixture of CH_3CN: H_2O in 3:1 ratio with TEA (0.6 ml) as sacrificial donor).

Supplementary Table 9. Calculation details of AQE (%) for methane production in presence of **Pt@Zn-TPY-TTF CPG** as photocatalyst at different wavelength from 400 nm to 700 nm.

**λ (nm)**	**Total CH_4 from 1 mg of catalyst in 1 h (μmol)**	***No. of CH_4 molecules[a] ($\times 10^{16}$)***	**Power measured at λ (mW s^{-1} cm^{-2})**	**Total Power[b] in 1h used on total area of photocatalytic cell (mW)**	**No. of photons[c] *($\times 10^{18}$)***	**AQE[d] = [(No. of CH_4 ×8)/No. of Photons]×100**
400 ±10	0.004	*0.261*	0.166	5746.67	11.61	0.18 %
450±10	0.003	*0.223*	0.162	5608.19	12.74	0.14 %
500±10	0.011	*0.641*	0.143	4950.44	12.50	0.41 %
550±10	0.025	*1.519*	0.156	5400.48	15.00	0.81 %
600±10	0.032	*1.921*	0.198	6854.46	20.77	0.74 %
650±10	0.011	*0.687*	0.186	6439.04	21.14	0.26 %
700±10	0.004	*0.239*	0.142	4915.82	17.38	0.11 %

S16. Control experiments and stability test of photocatalyst after CO_2 reduction process.

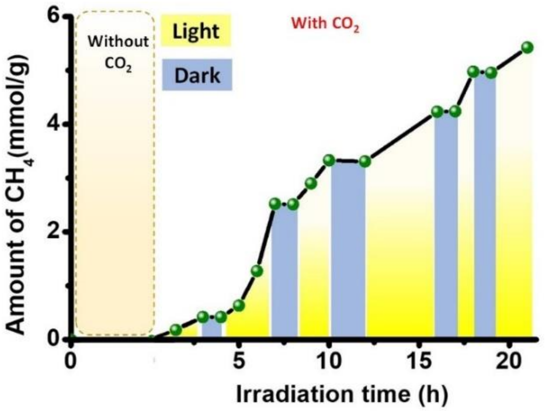

Supplementary Figure 55. Control experiment for visible light-driven CH_4 formation during photocatalytic CO_2 reduction by **Pt@Zn-TPY-TTF CPG**.

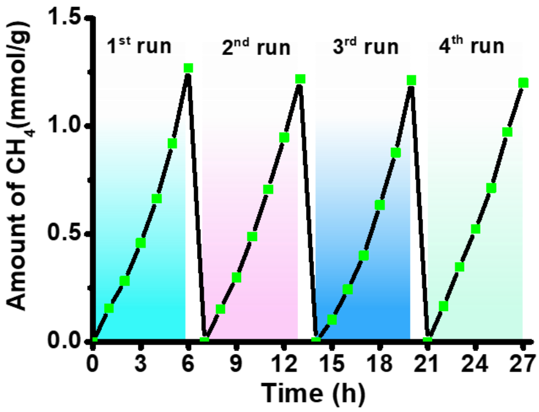

Supplementary Figure 56. Recyclability test for **Pt@Zn-TPY-TTF CPG** xerogel under visible light irradiation for CO_2 reduction. (The photocatalyst was recovered and reused after each cycle under similar condition).

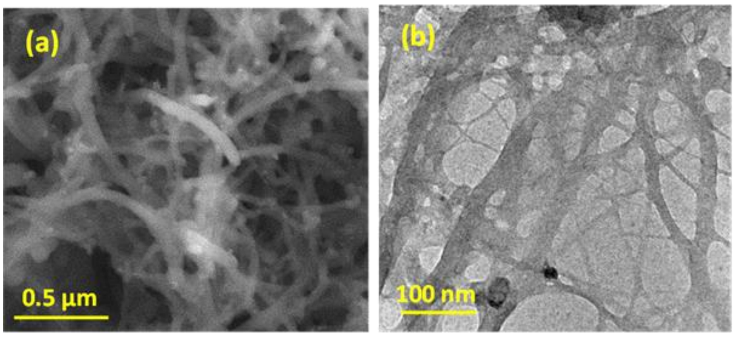

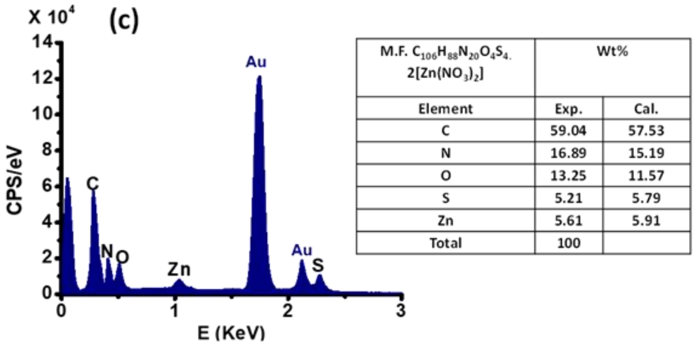

M.F. $C_{106}H_{88}N_{20}O_4S_4$. 2[$Zn(NO_3)_2$]	Wt%	
Element	Exp.	Cal.
C	59.04	57.53
N	16.89	15.19
O	13.25	11.57
S	5.21	5.79
Zn	5.61	5.91
Total	100	

Supplementary Figure 57. (a) FESEM image, (b)TEM image and (c) EDAX analysis for **Zn-TPY-TTF CPG** after performing photocatalytic CO_2 reduction experiment for 1 week.

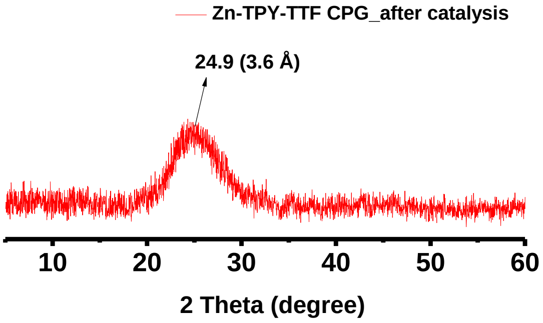

Supplementary Figure 58. PXRD analysis for **Zn-TPY-TTF CPG** xerogel after photocatalytic CO_2 reduction experiment for 1 week.

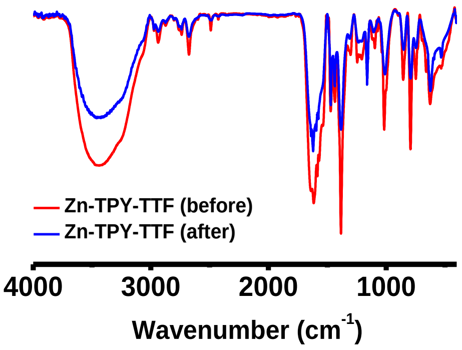

Supplementary Figure 59. FT-IR for **Zn-TPY-TTF CPG**.

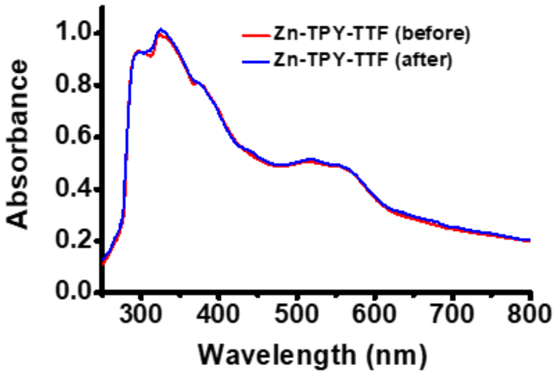

Supplementary Figure 60. UV-Visible absorption spectrum for **Zn-TPY-TTF CPG.**

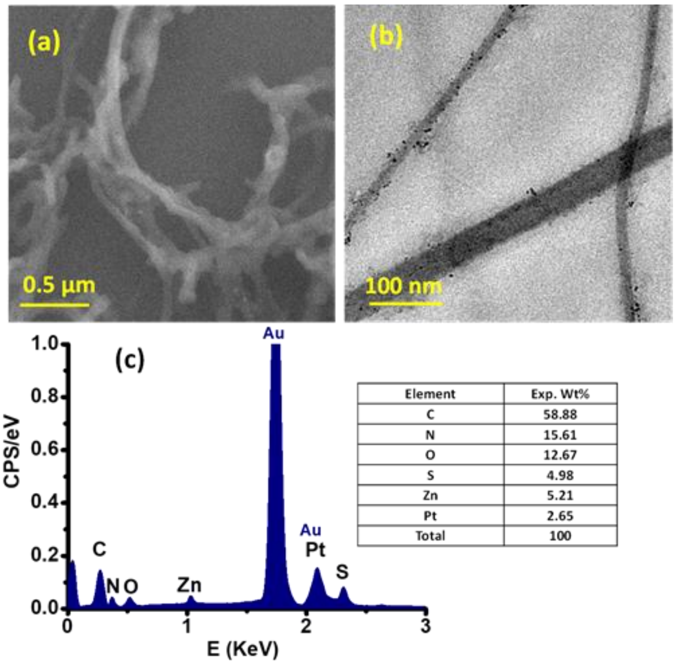

Element	Exp. Wt%
C	58.88
N	15.61
O	12.67
S	4.98
Zn	5.21
Pt	2.65
Total	100

Supplementary Figure 61. (a) FESEM image, (b) TEM image and (c) EDAX analysis for **Pt@Zn-TPY-TTF CPG** after performing photocatalytic CO_2 reduction experiment for 1 week.

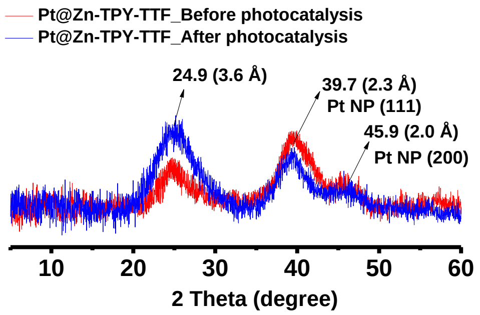

Supplementary Figure 62. PXRD analysis for **Pt@Zn-TPY-TTF CPG** before and after performing photocatalytic CO_2 reduction experiment for 1 week.

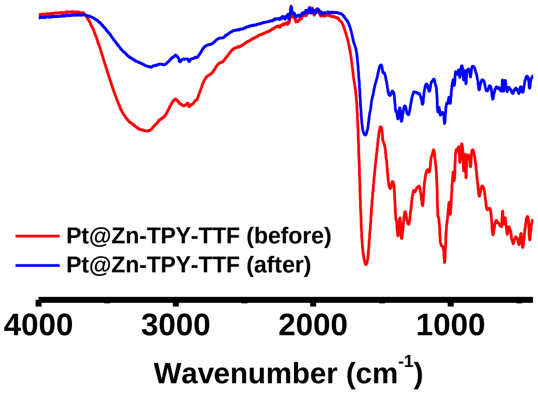

Supplementary Figure 63. FT-IR for **Pt@Zn-TPY-TTF CPG** before and after performing photocatalytic CO_2 reduction experiment for 1 week.

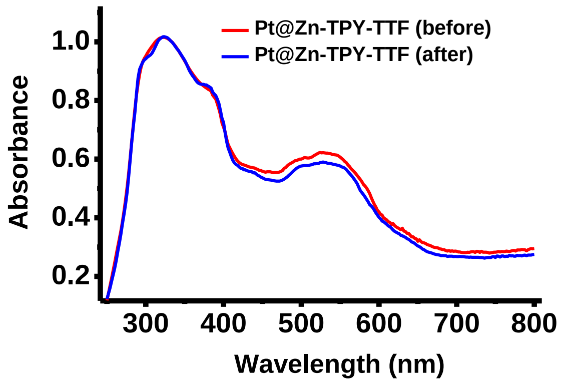

Supplementary Figure 64. UV-Visible absorption spectrum for **Pt@Zn-TPY-TTF CPG** before and after performing photocatalytic CO_2 reduction experiment for 1 week**.**

S17. Comparison Table for CO_2 reduction.

Supplementary Table 10. Comparison table for the activity of **TPY-TTF OG**, **Zn-TPY-TTF CPG** and **Pt@Zn-TPY-TTF CPG** towards the photocatalytic CO_2 reduction under visible light irradiation (400-750 nm).

Catalyst	Reaction medium	Selectivity of CO_2 reduction and amount of product	Irradiation Time (h)	Activity (μmol g^{-1} h^{-1})
TPY-TTF OG	CH_3CN: H_2O (3:1) + TEA	99 % CO, 1.12 mmol/g	11 h	140 μmol g^{-1} h^{-1}
**Zn-TPY-TTF CPG**	CH_3CN: H_2O (3:1) + TEA	99 % CO, 3.51 mmol/g	8 h	438 μmol g^{-1} h^{-1}
**Pt@Zn-TPY-TTF CPG**	CH_3CN: H_2O (3:1) + TEA	97 % CH_4, 8.74 mmol/g	30 h	292 μmol g^{-1} h^{-1}
Zn-TPY-TTF CPG	CH_3CN: H_2O (3:1)	92 % CO, 0.14 mmol/g	8 h	18 μmol g^{-1} h^{-1}
Zn-TPY-TTF CPG	H_2O	16 % CO, 0.01 mmol/g	8 h	2 μmol g^{-1} h^{-1}
Pt@Zn-TPY-TTF CPG	CH_3CN: H_2O (3:1)	86 % CH_4, 0.40 mmol/g	12 h	33 μmol g^{-1} h^{-1}
Pt@Zn-TPY-TTF CPG	H_2O	11 % CH_4, 0.11 mmol/g	12 h	9 μmol g^{-1} h^{-1}

Supplementary Table 11. Photocatalytic CO_2 reduction activity of **Zn-TPY-TTF CPG** and **Pt@Zn-TPY-TTF CPG** under sunlight irradiation.

Catalyst	Amount of CO_2 reduction Product (mmol/g)	Irradiation Time (h)	Activity (μmol g^{-1} h^{-1})	TON
**Zn-TPY-TTF CPG**	CO: 1.79 mmol/g on 30th sept	6 h	298 μmol g^{-1} h^{-1}	3.9 in 6 h
**Pt@Zn-TPY-TTF CPG**	CH_4: 0.96 mmol/g on 1st Oct	6 h	160 μmol g^{-1} h^{-1}	6.9 in 6 h (w.r.t Pt)

Supplementary Table 12. Comparison table of visible light driven photocatalytic CO_2 reduction of **Zn-TPY-TTF CPG** and **Pt@Zn-TPY-TTF CPG** with some of the reported benchmark hybrid materials reported in the literature.

No.	Catalyst	Reaction medium	Activity for CH_4	Activity for CO	Activity for H_2	AQE%	ref
1.	**Zn-TPY-TTF CPG**	CO_2/ H_2O, CH_3CN/ TEA	--	438 µmol g^{-1} h^{-1}	--	at 550 nm=0.96%	This work
2.	**Pt@Zn-TPY-TTF CPG**	CO_2/ H_2O, CH_3CN/ TEA	292 µmol g^{-1} h^{-1}	--	>3%	at 550 nm=0.81 %	This work
3.	(CoPPc)/ (mpg-CN_x)	CO_2/ CH_3CN/ TEOA	--	450 µmol g^{-1} in 24 hr	--	0.03% at 400 nm	ref[23]
4.	Zn-Porphyrin-TTF-COF (TTCOF-Zn)	CO_2/ H_2O	--	123.3 µmol g^{-1} >60 h	--	0.00027 % at 450 nm	ref[24]
5.	TRP	CO_2/ H_2O Vapour	8.4 µmol g^{-1} h^{-1}	488.8 µmol g^{-1} h^{-1}	--	6.7 % at 420 nm	ref[25]
6.	$Zn_{0.4}Ca_{0.6}In_2S_4$	CO_2	0.877 µmol g^{-1} h^{-1}	0.224 µmol g^{-1} h^{-1}	--	NP	ref[26]
7.	2,2′-bipyridine-based COF/ Ni-TpBpy, PS: $Ru(bpy)_3]Cl_2$	CO_2/H_2O/ TEOA	--	811 µmol g^{-1} h^{-1}	34 µmol g^{-1} h^{-1}	0.3 % at 420 nm	ref[27]
8.	(CsPbBr3 QD/GO) composite	CO_2/ Ethyl acetate	29.6 µmol g^{-1}	58.7 µmol g^{-1}	1.58 µmol g^{-1}	~ 0.022 % at 450 nm	ref[28]
9.	COF/ [Re(bpy)(CO)$_3$Cl] + Dye as PS: (Ir[dF(CF_3)p	CO_2/ H_2O/ TEOA	--	1400 µmol g^{-1} h^{-1}	> 14 %	0.5% at 420 nm	ref[29]

	py]$_2$(dtbpy))PF$_6$						
10.	NiCoOP NPs/ [Ru(bpy)$_3$]Cl$_2$·6H$_2$O	CO_2/ CH_3CN/ TEOA	--	16.6 μmol h^{-1}	--	NP	ref[30]
11.	Coqpy@mpg-C_3N_4	CH_3CN /BIH/ PhOH	--	1.15 μmol	0.06 μmol	0.25%	ref[31]
12.	CN-ATZ-NaK	CO_2	1 μmol g^{-1} h^{-1}	14 μmol g^{-1} h^{-1}	--	NP	ref[9]
13.	COF-367 + PS: [Ru(bpy)$_3$] Cl$_2$	CO_2/0.1 M $KHCO_3$ /ascorbic acid	--	10162 μmol g^{-1} h^{-1}	2875 μmol g^{-1} h^{-1}	NP	ref[32]
14.	Ni-Complex PS: [Ru(bpy)$_3$] Cl$_2$	CO_2/DMA, H_2O/ BIH	--	0.17 μmol min^{-1}	0.0473 μmol	11.1 % at 450 nm	ref[33]
15.	2D-triazine-COF-Re-complex	CO_2/CH_3CN/ TEOA	--	15 mmol/g >20 h	2% H_2	NP	ref[34]
16.	Ni(II) complex<br>PS: [Ru(bpy)$_3$] Cl$_2$	CO_2/ DMA, H_2O/ BIH	--	~90 μmol in >50 h	>1% H_2	1.42 %	ref[35]
17.	ZnPd-TiO_2 nanotube arrays (TNAs)	CO_2	26.83 μmol g^{-1} h^{-1}	--	--	NP	ref[36]
18.	g-CN/ Ni Nanoparticles	gas-phase CO_2 methanation at (150 °C)	28 μmol g^{-1} h^{-1}	--	--	0.01 %	ref[37]
19.	Ru$_2$-Mn / [MnBr(CO)$_3$ (BL)], [Ru(dmb)$_2$ (BL)]$^{2+}$	CO_2/DMA/ TEOA/ BIH	--	3.5 μmol	0.10 μmol	0.43%	ref[38]

20.	1 % Pt/In_2O_3	$CO_2 + H_2$	21.0 μmol g^{-1}	8.6 μmol g^{-1}	--	NP	ref[39]
21.	(HUY@S-TOH/AuPd)	CO_2/ H_2O	126 μmol g^{-1}	8 μmol g^{-1}	37 μmol g^{-1}	NP	ref[40]
22.	Ni(terpy-$S)_2$/CdS	CO_2/ DMF/ TEOA	--	~1 μmol in 4 hr	~ 0.1 μmol in 4 hr	At 400 nm=0.28 %	ref[41]
23.	Pt 3wt %/ $ZnIn_2S_4$@CNO	CO_2/ CH_3CN, H_2O/ TEOA	1.18 μmol h^{-1}	12.69 μmol h^{-1}	188.4 μmol h^{-1}	NP	ref[42]
24.	In−Fe_n TCPP-MOF	CO_2/ L-ascorbgyl palmitate/ ethyl acetate	--	3469 μmol g^{-1} in 24 hr	--	NP	ref[43]
25.	20% Mt/m-CN	CO_2/ H_2	345 μmol g^{-1} h^{-1}	291 μmol g^{-1} h^{-1}	--	4.55 % for CH_4 and<br>0.96 for CO	ref[44]
26.	Pd-g-C_3N_4/RGO	CO_2	6.4 μmol g^{-1} h^{-1}	--	--	NP	ref[45]
27.	$CsPbBr_3$ /Cu-RGO	CO_2/ H_2O (g)	12.7	0.46	0.27	1.1 % at 523 nm	ref[46]
28.	Pt_n/3 DOM-$SrTiO_3$	CO_2/ H_2O	27.7 μmol g^{-1} h^{-1}	4.1 μmol g^{-1} h^{-1}	--	CO_2 +CH_4 = 0.66%	ref[47]
29.	OD/1D Au/TiO_2	CO_2/ H_2O	70.34 μmol g^{-1} h^{-1}	19.75 μmol g^{-1} h^{-1}	--	NP	ref[48]
30.	$CsPbBr_3$-Re$(CO)_3$Br(dcbpy) composite	CO_2/ Toluene/ IPA	--	104.37 μmol g^{-1} in 3 hr	5.64 μmol g^{-1} in 3 hr	NP	ref[49]
31.	Mo/g-C_3N_4 hybrids	CO_2/ H_2O	3.8 μmol g^{-1} h^{-1}	--	--	NP	ref[50]
32.	$CsPbBr_3$/ MXene nano-composites	CO_2/ Ethyl acetate	14.16 μmol g^{-1} h^{-1}	32.15 μmol g^{-1} h^{-1}	--	NP	ref[51]

33.	UIO-66-NH_2/2.0GR hybrid	CO_2/ DMF, H_2O/ TEOA	0.90 µmol in 4 h	13.2 µmol in 4 h	--	NP	ref[52]
34.	fac-[Mn(phen)(CO)$_3$Br], PS: Zn-Porphyrin	CO_2/ CH_3CN, H_2O/ TEA	--	TON 64 in 3 h	--	NP	ref[53]
35.	Co-ZIF-9 MOF/ CdS	Bipyridine, CH_3CN, H_2O/ TEOA	--	50.4 µmol h^{-1}	11.1 µmol h^{-1}	NP	ref[54]

S18. DFT calculations for CO_2 reduction mechanism.

Computational details. All electronic structure calculations were performed under the framework of Density Functional Theory. The molecular geometries were optimized using B3LYP-D3[55-60] exchange-correlation functional. For geometry optimization, 6-31G (d,p) basis set was used for all atoms except for Zn and Pt atoms, for which LANL2DZ, which utilized a widely used effective core potential (ECP)- type basis set, is used.[61-62] The optimized structures were subjected to harmonic vibrational frequency analysis to confirm the nature of the stationary points. Then, the optical properties were calculated using time-dependent density functional theory (TD-DFT) methods with long-range corrected CAM-B3LYP[63] exchange-correlation functional. For TD-DFT calculations, 6-31+G(d,p) basis set was used for all atoms except Zn, as previously mentioned. Solvent effects were taken into account using PCM model with the integral equation formalism variant.[64] However, for mechanistic studies, the electronic energies were further refined with single point energy calculations using a higher basis set 6-311++g(d,p) for the lighter atoms. Transition state was further verified by intrinsic reaction coordinate (IRC) calculations by checking its connection to two respective minimum structures. All thermochemical data were obtained with the ideal gas-rigid rotor-simple harmonic oscillator approximations at 298.15 K and 1 atm. Zero point-energy corrections were included in the Gibbs free energy values along with a concentration correction for c = 1 mol/ dm^3 condition in the solvent. All calculations were performed using Gaussian 16 package.[65] The pictures of the optimized structures, molecular orbitals, and spin density plots were taken from Gauss View 6.0.16.[66]

Theoretical investigation of mechanism for CH_4 formation (based on Pt@Zn-TPY-TTF CPG). The DFT calculation was performed for Pt@Zn-TPY-TTF CPG to construct probable mechanism for the CH_4 formation in support of in situ DRIFT study (Supplementary Fig. 68). Step-wise calculations revealed that the CO_2 reduction mechanism on Pt@Zn-TPY-TTF CPG followed a similar pathway as in the case of Zn-TPY-TTF CPG up to the COOH-bound intermediate, $[Zn(TPY^{\bullet-})(\eta^2\text{-}TPY)(COOH)]^+$·Pt (Supplementary Fig. 67, Supplementary Fig. 68 (i-v)). However, the subsequent protonation and water elimination from $[Zn(TPY^{\bullet-})(\eta^2\text{-}TPY)(COOH)]^+$·Pt intermediate resulted in fast removal of CO, which would immediately bound to the adjacent Pt centre to afford the intermediate $[Zn(TPY)_2]^{2+}$·Pt-CO and thus the further reduction processes occurred on platinum centre rather than the Zn^{II} centre (Supplementary Fig. 68 (vi)). The generation and binding of CO on platinum surface leading to the formation of $[Zn(TPY)_2]^{2+}$·Pt-CO intermediate was found to be highly

thermodynamically feasible (ΔG = -5.81 eV). In the following step, one electron reduction of $[Zn(TPY)_2]^{2+}$·Pt-CO afforded to the formation of $[Zn(TPY^{•-})(TPY)]^{+}$·Pt-CO which is a downhill process (ΔG = -0.92 eV) (Supplementary Fig. 68 (vii)). Next, the intermediate $[Zn(TPY^{•-})(TPY)]^{+}$·Pt-CO could have either release CO or undergo further reduction. However, proton coupled reduction of $[Zn(TPY^{•-})(TPY)]^{+}$·Pt-CO was found to be an exoergic process (ΔG = -0.68 eV) as compared to the highly endoergic process of CO removal (ΔG = +2.38 eV). Thus, the intermediate $[Zn(TPY^{•-})(TPY)]^{+}$·Pt-CO was expected to undergo proton coupled reduction to afford $[Zn(TPY^{•-})(TPY)]^{+}$·Pt-CHO rather than releasing the CO (Supplementary Fig. 68 (viii)). The subsequent proton coupled reduction of $[Zn(TPY^{•-})(TPY)]^{+}$·Pt-CHO would lead to the formation of $[Zn(TPY^{•-})(TPY)]^{+}$·Pt-OCH_3 (ΔG = -2.16 eV) via the intermediate species, $[Zn(TPY^{•-})(TPY)]^{+}$·Pt-(η^2-OCH_2) (ΔG = -0.74 eV) (Supplementary Fig. 68 (ix-x))). This would further undergo proton coupled reduction and subsequently release the CH_4 with the generation of $[Zn(TPY)_2]^{2+}$·Pt-O^- (ΔG = -1.40 eV) (Supplementary Fig. 68 (xi)). The formulation of the intermediate as $[Zn(TPY)_2]^{2+}$·Pt-O^- rather than $[Zn(TPY^{•-})(TPY)]^{+}$·Pt-O was affirmed from the spin density distribution plot (Supplementary Table 54). Lastly, after subsequent two step proton coupled reduction, the intermediate $[Zn(TPY)_2]^{2+}$.Pt-O^- led to the formation of initial active species $[Zn(TPY^{•-})(TPY)]^{+}$·Pt with the release of water molecule. The downhill processes from $[Zn(TPY)_2]^{2+}$·Pt-O^- to $[Zn(TPY^{•-})(TPY)]^{+}$·Pt-OH (ΔG = -2.89 eV) and to the regeneration of $[Zn(TPY^{•-})(TPY)]^{+}$·Pt (ΔG = -0.63 eV) explains the high activity of the catalyst in CO_2RR to CH_4 (Supplementary Fig. 68 (xii)). We have also taken into consideration and constructed a mechanism for the hydrogenation pathway (Supplementary Fig. 70). However, the lower thermodynamic feasibility of the hydrogenation processes further reinforces the proton-coupled reduction pathway as the major one (Supplementary Fig. 69-70). These results clearly showed that Pt NPs in the Pt@Zn-TPY-TTF CPG play a crucial role in modulating the CO_2 reduction product from CO to CH_4.

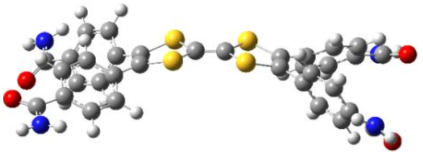

(a)

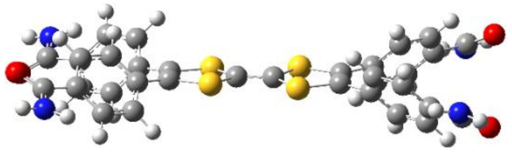

(b)

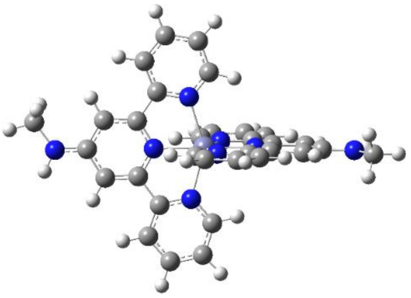

(c)

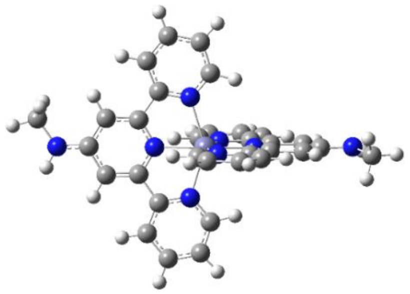

(d)

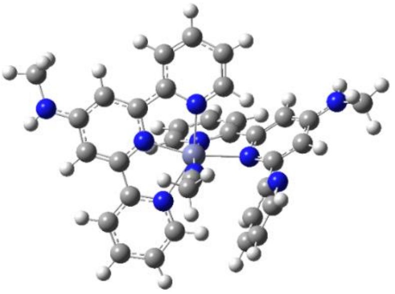

(e)

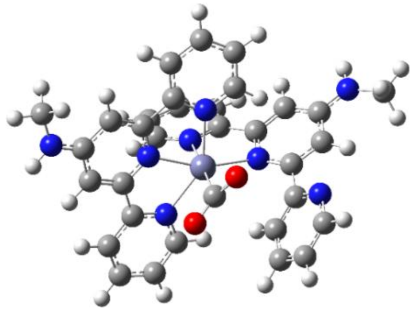

(f)

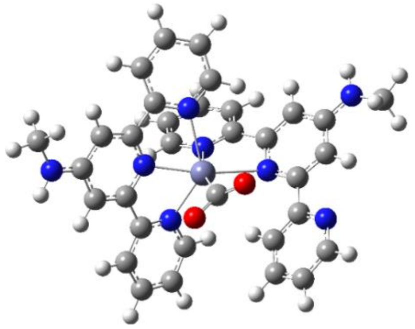

(g)

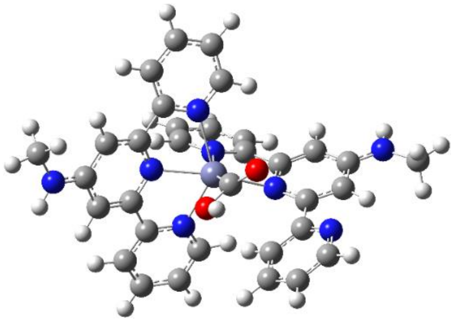

(h)

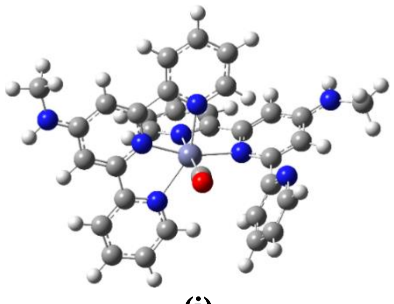

(i)

Supplementary Figure 65. Optimized geometries of (a) **TTF**, (b) **TTF⁺**, (c) **$[Zn^{II}(TPY)_2]^{2+}$,** (d) **$[Zn^{II}(TPY^{\bullet-})(TPY)]^{+}$,** (e) **$[Zn^{II}(TPY^{\bullet-})(\eta^2\text{-}TPY)(CH_3CN)]^{+}$,** (f) **$[Zn^{II}(TPY)(\eta^2$-**

TPY)(COO$^-$)]$^+$, (g) **[Zn^{II}(TPY$^{\bullet -}$)(η^2-TPY)(COO$^-$)]**, (h) **[Zn^{II}(TPY$^{\bullet -}$)(η^2-TPY)(COOH)]$^+$**, (i) **[Zn^{II}(TPY)(η^2-TPY)(CO)]$^{2+}$**.

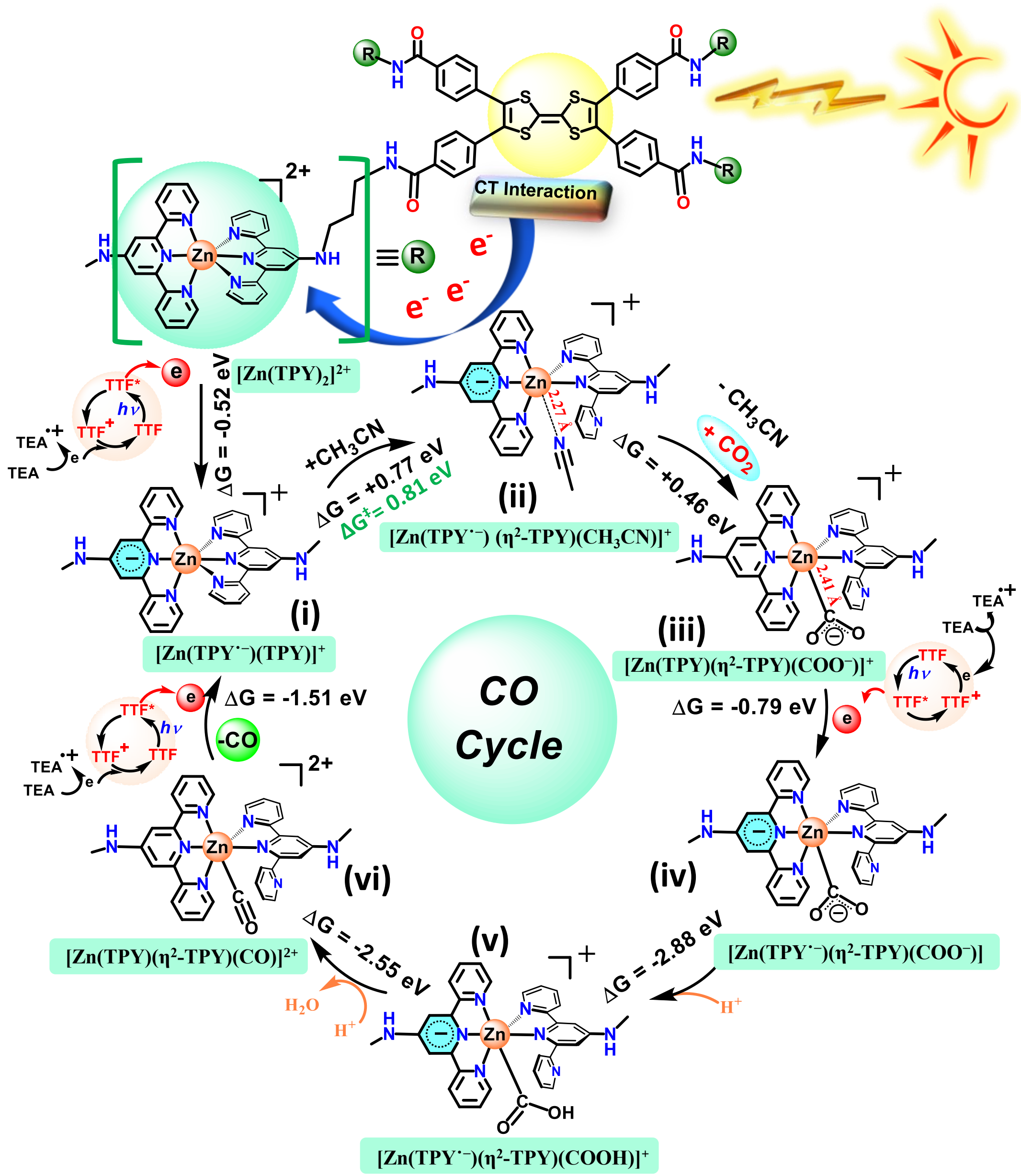

Supplementary Figure 66. Plausible mechanism based on the theoretical study for CO_2 reduction to CO by Zn-TPY-TTF CPG.

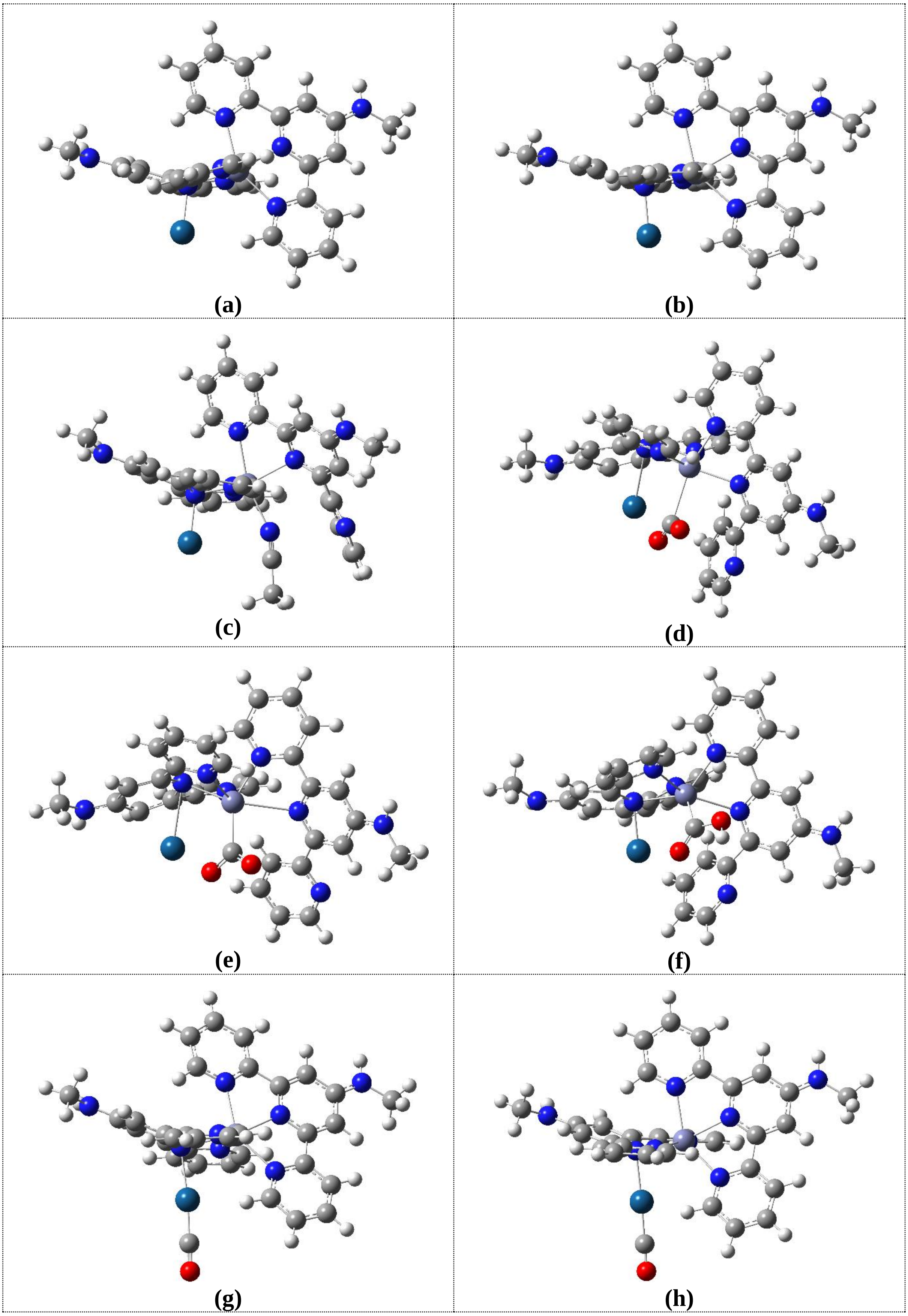
(a)
(b)
(c)
(d)
(e)
(f)
(g)
(h)

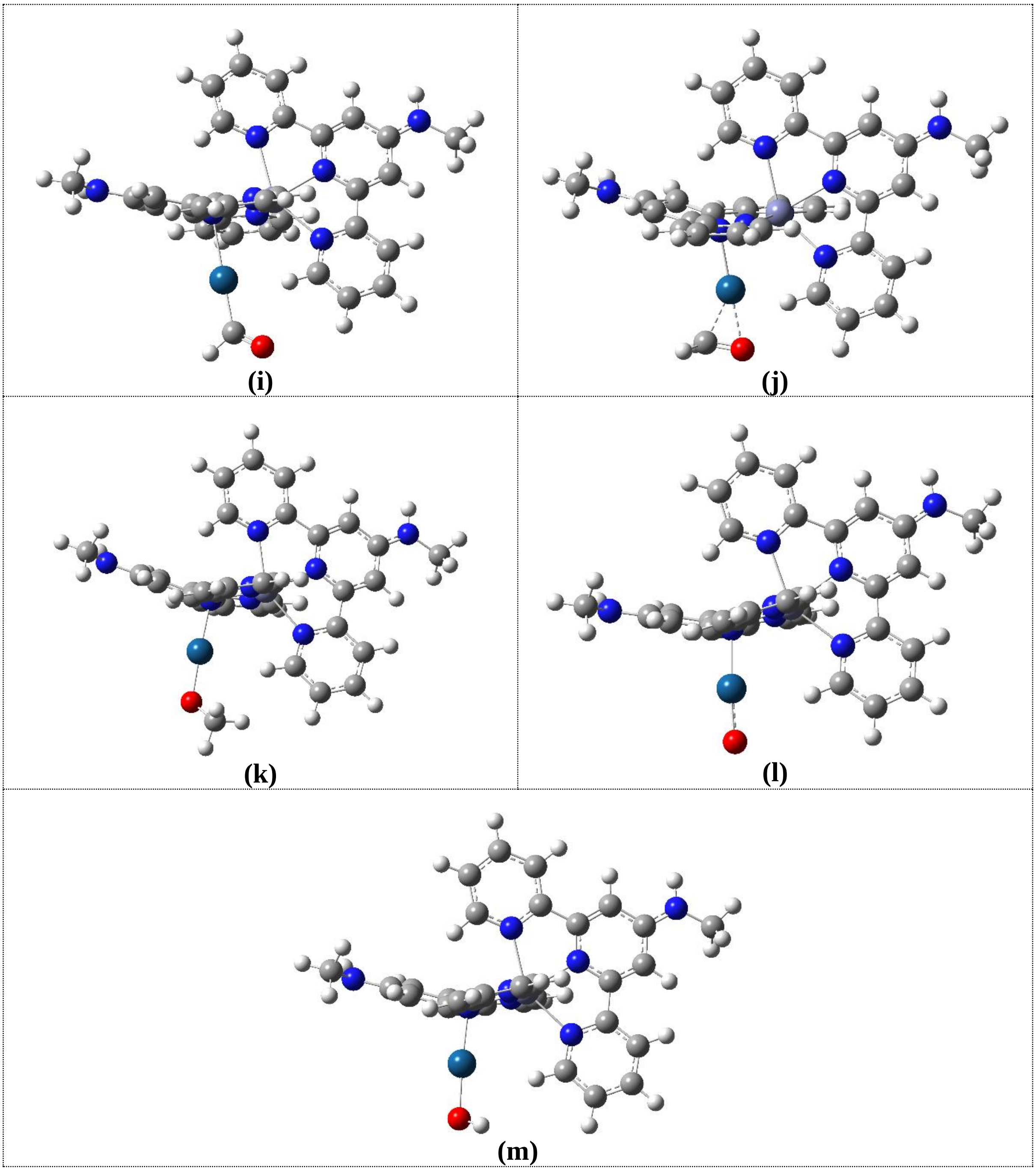

Supplementary Figure 67. Optimized geometries of (a) **[Zn^{II}(TPY)$_2$]$^{2+}$·Pt,** (b) **[Zn^{II}(TPY$^{\bullet-}$)(TPY)]$^{+}$·Pt,** (c) **[Zn^{II}(TPY$^{\bullet-}$)(η^2-TPY)(CH$_3$CN)]$^{+}$·Pt,** (d) **[Zn^{II}(TPY)(η^2-TPY)(COO^{-})]$^{+}$·Pt,** (e) **[Zn^{II}(TPY$^{\bullet-}$)(η^2-TPY)(COO^{-})]·Pt,** (f) **[Zn^{II}(TPY$^{\bullet-}$)(η^2-TPY)(COOH)]$^{+}$·Pt,** (g) **[Zn^{II}(TPY)$_2$]$^{2+}$·Pt-CO**, (h) **[Zn^{II}(TPY$^{\bullet-}$)(TPY)]$^{+}$·Pt-CO**, (i) **[Zn^{II}(TPY$^{\bullet-}$)(TPY)]$^{+}$·Pt-CHO**, (j) **[Zn^{II}(TPY$^{\bullet-}$)(TPY)]$^{+}$·Pt-(η^2-OCH$_2$)**, (k) **[Zn^{II}(TPY$^{\bullet-}$)(TPY)]$^{+}$·Pt-OCH$_3$**, (l) **[Zn^{II}(TPY)$_2$]$^{2+}$·Pt-O^{-}**, (m) **[Zn^{II}(TPY$^{\bullet-}$)(TPY)]$^{+}$·Pt-OH**.

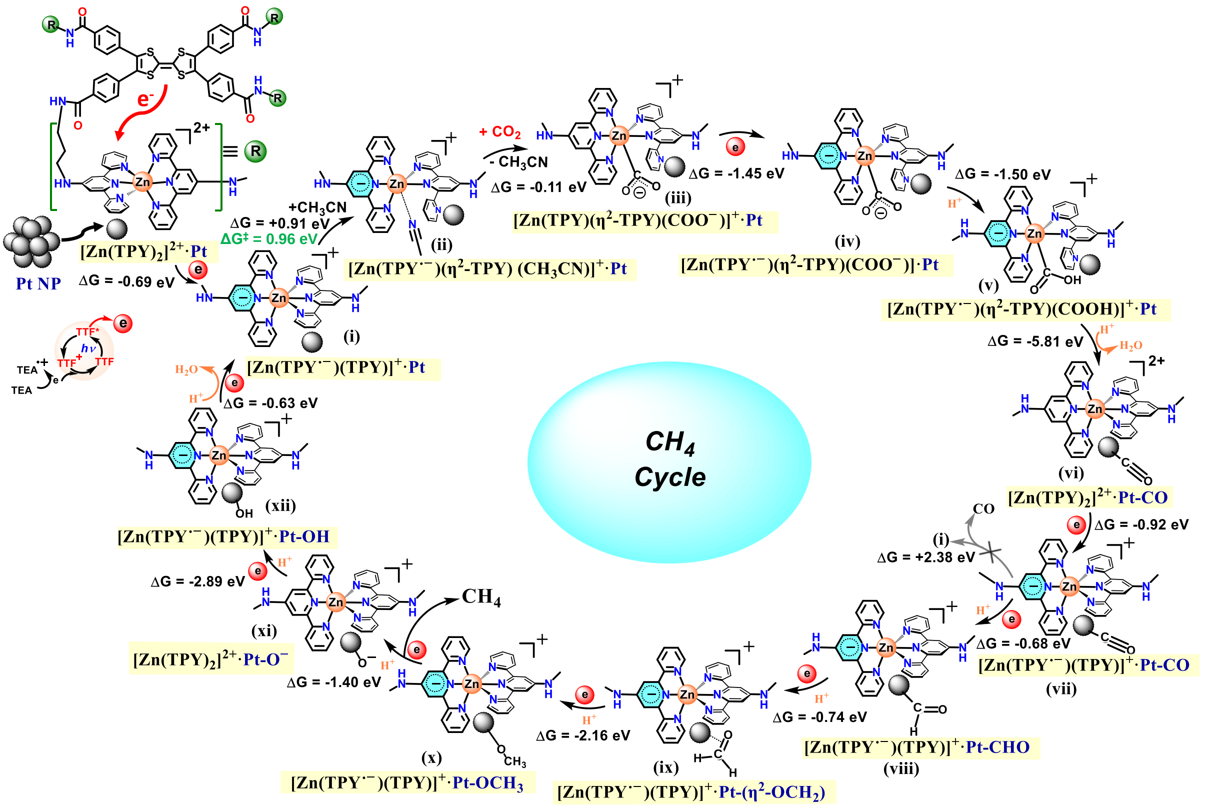

Supplementary Figure 68. Plausible mechanism based on the theoretical study for CO_2 reduction to CH_4 by Pt@Zn-TPY-TTF CPG.

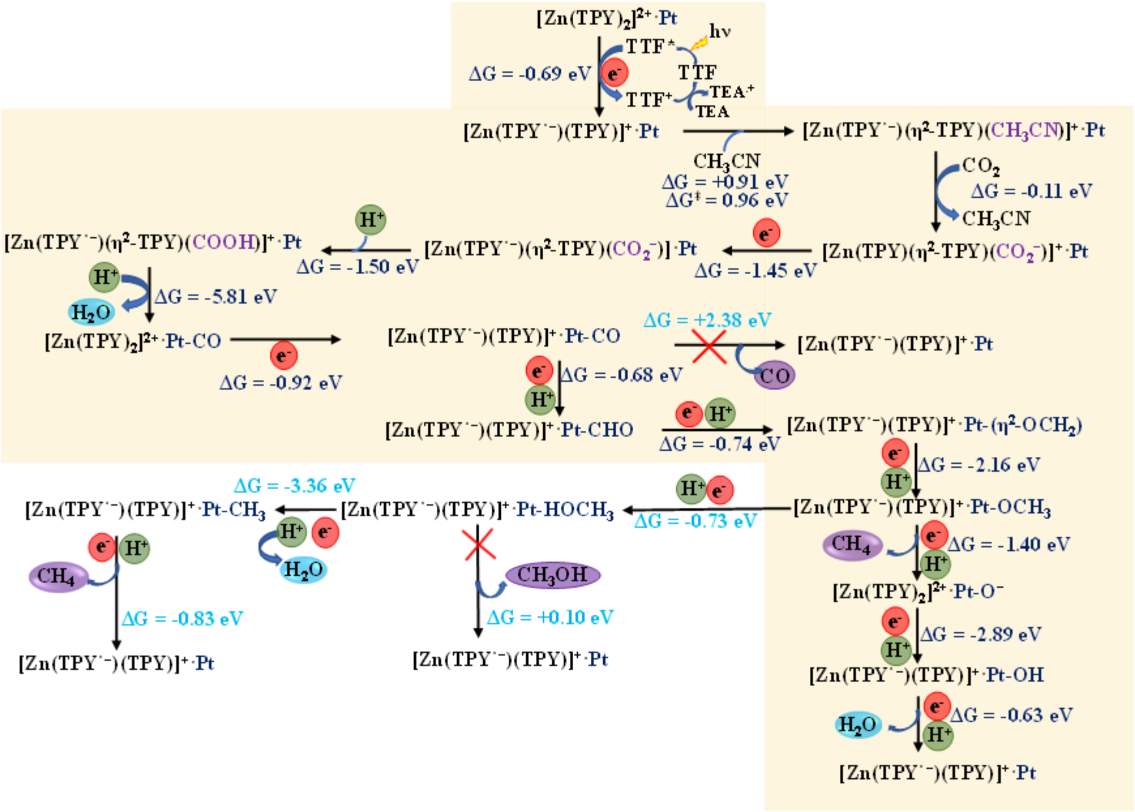

Supplementary Figure 69. Proton-coupled reduction mechanism. complete photocatalytic CO_2 reduction mechanism with respective relative Gibbs free energy values.

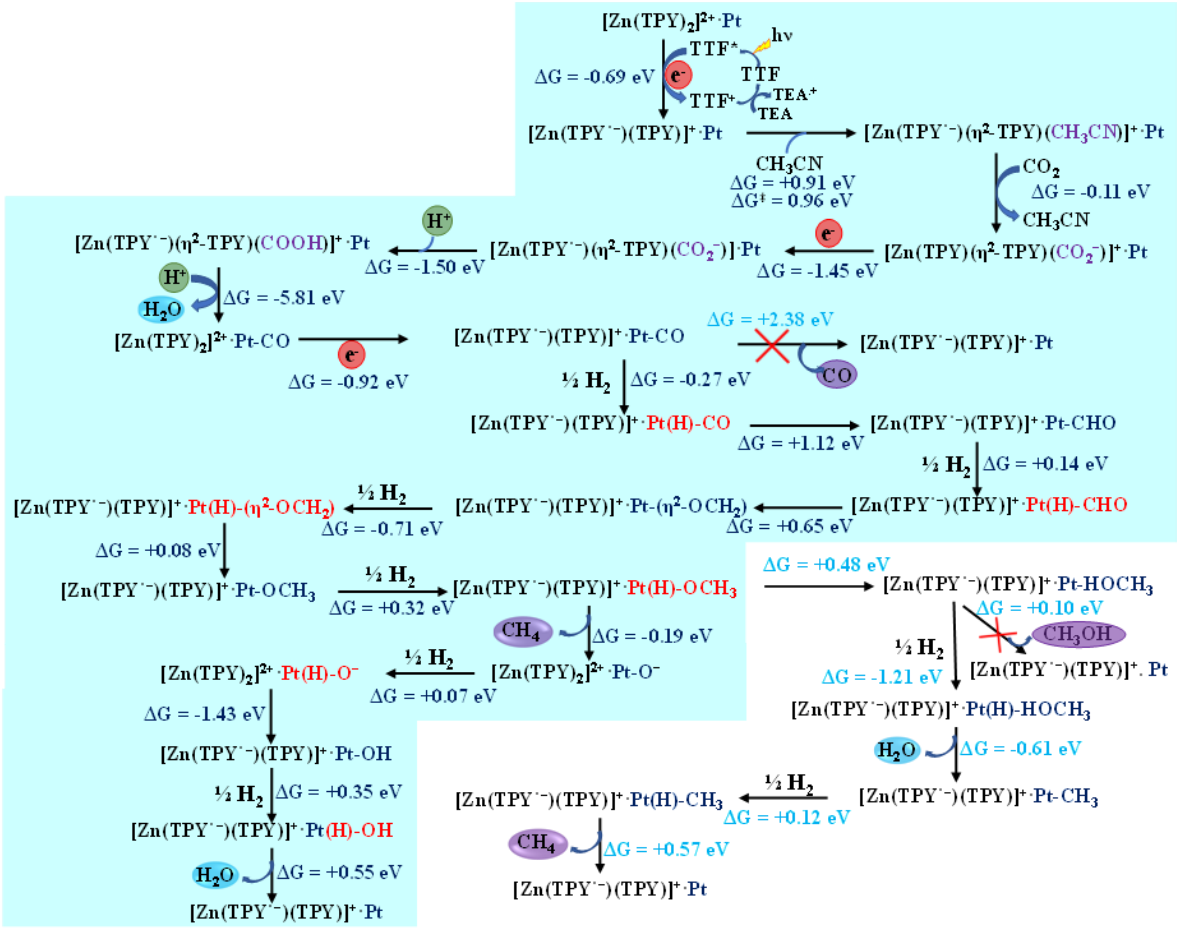

Supplementary Figure 70. Hydrogenation mechanism. complete photocatalytic CO_2 reduction mechanism with respective relative Gibbs free energy values.

Cartesian coordinates of the computed structures

Coordinates are given in standard XYZ format

Supplementary Table 13. DFT-optimized geometry of TPY-TTF LMWG (singlet), computed at the B3LYP/ 6-31G** (HCNOS) level in methanol solvent using IEFPCM.

Atom	x	y	z
C	-3.17987200	0.61901300	-1.62591200
C	-3.21334800	-0.73412100	-1.62370300
S	-1.70045100	-1.58217700	-2.04517700
C	-0.68283000	-0.12187300	-2.05030200
S	-1.62584100	1.38797700	-2.04331900
C	0.66773300	-0.15368500	-2.06659500
S	1.61793000	-1.65866700	-2.10094700
S	1.67886500	1.31111600	-2.06677800
C	3.17493600	-0.88724100	-1.69640600
C	3.20141000	0.46607600	-1.67645300
C	-4.37244900	-1.58798200	-1.28668600
C	-5.13587500	-1.32815100	-0.13491100

C	-4.72829100	-2.67266000	-2.10431200
C	-6.23513000	-2.11865600	0.17388400
H	-4.86211200	-0.50088500	0.51027200
C	-5.82218200	-3.47254800	-1.78298100
H	-4.16471100	-2.87569400	-3.00951400
C	-6.58465800	-3.20684000	-0.63770700
H	-6.83549000	-1.91035600	1.05249400
H	-6.09546800	-4.27895400	-2.45609400
C	-4.31961300	1.53687400	-1.41774400
C	-5.54133500	1.31910300	-2.07994100
C	-4.19679200	2.65339900	-0.57477800
C	-6.60917000	2.18595700	-1.88978500
H	-5.64367700	0.46808000	-2.74379600
C	-5.26662800	3.52678800	-0.39510500
H	-3.26788800	2.82560000	-0.04075500
C	-6.48153100	3.30734200	-1.05786700
H	-7.55509200	2.01565900	-2.39176500
H	-5.15212200	4.35854000	0.29269100
C	4.35762400	1.32426300	-1.33980800
C	4.69381500	2.42491700	-2.14427800
C	5.13512800	1.05549300	-0.19952100
C	5.78080600	3.23306900	-1.82025300
H	4.11964900	2.63520400	-3.04114100
C	6.22806700	1.85396300	0.11132300
H	4.87690600	0.21545000	0.43546600
C	6.55643300	2.95924300	-0.68586200
H	6.03819400	4.05352200	-2.48259100
H	6.83890300	1.63927300	0.98116000
C	4.32453600	-1.79764000	-1.51221900
C	4.22310400	-2.92558100	-0.68176000
C	5.53910400	-1.55440500	-2.17902400
C	5.30924600	-3.78123300	-0.51302800
H	3.29934700	-3.11887100	-0.14601500
C	6.62327900	-2.40237000	-1.99849700
H	5.62419000	-0.69592300	-2.83560100
C	6.51937700	-3.53138000	-1.17357800
H	5.21222500	-4.62113800	0.16753600
H	7.56501000	-2.20908500	-2.49995300
C	-7.79587800	-4.00687500	-0.24906100
O	-8.70186000	-3.50817500	0.42861100
N	-7.83630700	-5.29811600	-0.66919200
H	-7.04784700	-5.69328800	-1.15778800
C	-7.67925600	4.20262300	-0.90693900
O	-8.82481100	3.78036900	-1.10281600
N	-7.43697800	5.49355800	-0.56126300
H	-6.48785400	5.82810600	-0.50155700
C	7.75896900	3.77078600	-0.29424600
O	8.68157300	3.27316200	0.36141800
N	7.77055500	5.07168300	-0.68529900
H	6.96971100	5.46169800	-1.15771000
C	7.74043600	-4.39299200	-1.01605400
O	8.87472200	-3.93255500	-1.19059300
N	7.53477400	-5.69430600	-0.68568500
H	6.59600200	-6.05699000	-0.62476100
C	8.91136100	5.93852100	-0.42101900
H	9.28932200	5.70603200	0.57915900
H	8.55550300	6.97237200	-0.41609000
C	8.64980400	-6.60301800	-0.45180000
H	9.41836900	-6.39932900	-1.20392300
H	8.28983600	-7.62402000	-0.60378800

C	-8.99179600	-6.14753900	-0.41162400
H	-9.34472300	-5.94276200	0.60376400
H	-8.66031900	-7.18865900	-0.45076000
C	-8.52228400	6.44774100	-0.37285200
H	-9.25844700	6.29205000	-1.16816300
H	-8.10710300	7.45236700	-0.48929300
C	-9.20014100	6.30034200	0.99456600
H	-8.46034200	6.44224600	1.79044000
H	-9.58693600	5.27832300	1.07836400
C	-10.33864800	7.30424500	1.16550200
H	-9.94147700	8.32404600	1.08250000
H	-11.07404900	7.18117000	0.35464100
C	10.02868400	5.75695300	-1.45531600
H	9.64588400	5.99206000	-2.45507300
H	10.33040200	4.70303900	-1.45553700
C	11.23593700	6.64067400	-1.14722600
H	10.92565600	7.69375400	-1.12613400
H	11.62882100	6.40354400	-0.14609100
C	9.24305200	-6.44172500	0.95294400
H	8.47624900	-6.66424700	1.70385100
H	9.54077800	-5.39485200	1.08229900
C	10.45189200	-7.35074000	1.16607500
H	10.15958200	-8.39797300	1.01155100
H	11.22769200	-7.12232800	0.41860200
C	-10.12496700	-5.90149300	-1.41499000
H	-9.76041600	-6.10716700	-2.42910700
H	-10.40658200	-4.84403000	-1.37223700
C	-11.34597400	-6.76955300	-1.11701700
H	-11.70803800	-6.55495800	-0.10285100
H	-11.06678100	-7.83477200	-1.13735100
N	12.26264900	6.46716000	-2.16424200
C	13.45948700	7.12496600	-2.15189400
H	12.22336800	5.61212800	-2.70081200
C	13.74503700	8.16604500	-1.24696300
C	14.47513400	6.78565900	-3.07111200
C	14.98870500	8.80265800	-1.30874600
H	13.02809800	8.45113400	-0.48826000
C	15.68254600	7.47977400	-3.05059500
H	14.31765600	5.97028600	-3.76963300
C	15.29564700	9.91616800	-0.35802000
N	15.95403100	8.47566700	-2.18346700
C	16.75804100	7.13116400	-4.02971500
C	14.29658800	10.82070800	0.03512400
N	16.56285800	10.00832800	0.08920300
C	16.43789400	6.74897100	-5.34207000
N	18.03075400	7.20772900	-3.59554800
C	14.61737500	11.84263100	0.92586100
H	13.29346100	10.73707200	-0.36804000
C	16.85380200	10.99521600	0.94478100
C	17.46515000	6.42469300	-6.22528300
H	15.40425600	6.72860600	-5.66903600
C	19.00548000	6.89280500	-4.45666800
C	15.92558200	11.93497700	1.39782000
H	13.86152400	12.55696200	1.23733100
H	17.88505200	11.03799700	1.29098800
C	18.78284700	6.49360500	-5.77607200
H	17.23969300	6.13305500	-7.24643100
H	20.02178100	6.96016000	-4.07225900
H	16.22488500	12.71280000	2.09220400
H	19.61733700	6.25189200	-6.42565000

N	10.97109800	-7.19386900	2.51700600
C	12.07351200	-7.85121000	2.98384900
H	10.71182700	-6.34657900	3.00230400
C	12.70485400	-8.88281000	2.26154200
C	12.63026200	-7.52038300	4.23771800
C	13.82223500	-9.51743700	2.81335400
H	12.35511700	-9.16170100	1.27617500
C	13.74686500	-8.21308000	4.69975100
H	12.20148400	-6.71134700	4.81998900
C	14.49534600	-10.61835300	2.05667100
N	14.35080500	-9.19861900	4.00611800
C	14.33491600	-7.87441500	6.03249500
C	13.74752100	-11.52189500	1.28533900
N	15.83603900	-10.69979800	2.15795200
C	13.51227100	-7.50112000	7.10709700
N	15.67465900	-7.95162700	6.14824000
C	14.40906200	-12.53187200	0.59035800
H	12.66637800	-11.44678700	1.25020500
C	16.45580100	-11.67522200	1.48339700
C	14.09573100	-7.18610300	8.33220100
H	12.43452500	-7.48055200	6.98967700
C	16.21890600	-7.64557800	7.33181800
C	15.79723100	-12.61332300	0.68569300
H	13.84924300	-13.24544700	-0.00630100
H	17.53941500	-11.70965900	1.58223500
C	15.48268600	-7.25526800	8.45227100
H	13.47734200	-6.90134700	9.17784300
H	17.30392000	-7.71305200	7.38976500
H	16.35784800	-13.38163100	0.16399700
H	15.98407500	-7.02053600	9.38515100
N	-12.40704100	-6.49680500	-2.07503400
C	-13.60906000	-7.14304600	-2.08137500
H	-12.13750400	-6.03097800	-2.92950900
C	-14.01555000	-7.99912300	-1.03875100
C	-14.52612800	-6.94582000	-3.13604700
C	-15.28228300	-8.58866400	-1.09437700
H	-13.37194900	-8.17697900	-0.18710000
C	-15.76420300	-7.58272300	-3.09972400
H	-14.27227200	-6.27614300	-3.95139000
C	-15.72031300	-9.50144500	0.00724600
N	-16.15594400	-8.39228000	-2.09551100
C	-16.73727700	-7.38287400	-4.21771500
C	-14.80629200	-10.36755000	0.62773500
N	-17.01852100	-9.45337900	0.36348800
C	-16.29318000	-7.26564400	-5.54423700
N	-18.04277100	-7.32720200	-3.89072800
C	-15.24795400	-11.20001900	1.65370600
H	-13.77461400	-10.40259000	0.29573800
C	-17.42587600	-10.25881500	1.35146100
C	-17.22772600	-7.07322800	-6.55920000
H	-15.23705600	-7.34636800	-5.77632000
C	-18.92720800	-7.14161500	-4.87772100
C	-16.58870600	-11.14621900	2.03087100
H	-14.55925700	-11.88260600	2.14194700
H	-18.47911100	-10.19123300	1.61844800
C	-18.57879100	-7.00585000	-6.22305800
H	-16.90626600	-6.98691900	-7.59256000
H	-19.97307300	-7.09611300	-4.57892800
H	-16.98088100	-11.77432800	2.82360700
H	-19.34456200	-6.85807600	-6.97705300

N	-10.96829400	7.14605500	2.46872000
C	-12.00626900	7.92385400	2.90059100
H	-10.88341900	6.23408900	2.89577300
C	-12.42718600	9.07829000	2.21185600
C	-12.70438800	7.60035700	4.08365200
C	-13.48340200	9.83522600	2.72846300
H	-11.96535000	9.36372400	1.27586800
C	-13.74813900	8.41722600	4.51287700
H	-12.44135400	6.70406200	4.63590400
C	-13.92532800	11.07089800	2.01032200
N	-14.14768800	9.52251000	3.85288400
C	-14.48809800	8.08405400	5.76926700
C	-12.99170100	11.90089000	1.36985600
N	-15.24396100	11.34529400	2.01590500
C	-13.81893900	7.52956700	6.87173700
N	-15.80925200	8.34634000	5.78983600
C	-13.43874800	13.04157900	0.70679800
H	-11.93364100	11.66727900	1.41122900
C	-15.65661500	12.44433100	1.37365600
C	-14.54173100	7.22653000	8.02339400
H	-12.74850900	7.36076200	6.83370100
C	-16.48853800	8.04893900	6.90374000
C	-14.80378600	13.32330500	0.70276500
H	-12.73279600	13.70081100	0.21117400
H	-16.72846200	12.63390800	1.39268600
C	-15.91073300	7.48766300	8.04434700
H	-14.04241800	6.80336100	8.88954700
H	-17.55471100	8.26829000	6.88533300
H	-15.20111600	14.19912900	0.20095200
H	-16.51669700	7.27019000	8.91742700

Supplementary Table 14. DFT-optimized geometry of TPY-TTF LMWG (singlet), computed at the B3LYP/ 6-31G** (HCNOS) level in absence of any solvent.

Atom	x	y	z
C	-3.17121000	0.72333800	-2.15010800
C	-3.18769100	-0.63075400	-2.14183200
S	-1.67552900	-1.46146000	-2.59271600
C	-0.67521800	0.00963100	-2.59844200
S	-1.63664100	1.50653100	-2.60971500
C	0.67540400	-0.00934300	-2.59842200
S	1.63683000	-1.50623800	-2.60978800
S	1.67571700	1.46174800	-2.59254900
C	3.17139600	-0.72308100	-2.15007200
C	3.18785700	0.63101300	-2.14167700
C	-4.32447200	-1.50002900	-1.77021500
C	-5.04844800	-1.26161500	-0.58918000
C	-4.69533300	-2.58337900	-2.58283300
C	-6.11669100	-2.07645400	-0.23929100
H	-4.76571100	-0.43002800	0.04665700
C	-5.76061900	-3.40487300	-2.22332700
H	-4.16280900	-2.76610600	-3.51126700
C	-6.47646900	-3.16665500	-1.04284000

H	-6.69067900	-1.89093300	0.66185300
H	-6.05277500	-4.20737600	-2.89431300
C	-4.31543600	1.62636400	-1.90906000
C	-5.56460900	1.37748700	-2.50627200
C	-4.17324100	2.76030600	-1.09229200
C	-6.63789800	2.22785000	-2.28010200
H	-5.68323600	0.51135400	-3.14736300
C	-5.24969500	3.61650200	-0.87557800
H	-3.22171700	2.95545200	-0.60780300
C	-6.49085600	3.36526600	-1.47438900
H	-7.60802800	2.03494400	-2.72458000
H	-5.12135300	4.45935800	-0.20273400
C	4.32461200	1.50028100	-1.76993900
C	4.69556600	2.58361800	-2.58253100
C	5.04844800	1.26187800	-0.58881800
C	5.76083100	3.40509800	-2.22292800
H	4.16312900	2.76634400	-3.51101400
C	6.11667000	2.07670400	-0.23883000
H	4.76562400	0.43031200	0.04700600
C	6.47655600	3.16687800	-1.04236600
H	6.05307000	4.20759500	-2.89388400
H	6.69055800	1.89119200	0.66238000
C	4.31560500	-1.62613700	-1.90907000
C	4.17336200	-2.76014700	-1.09240200
C	5.56480000	-1.37725300	-2.50623600
C	5.24979000	-3.61638200	-0.87571600
H	3.22181800	-2.95531600	-0.60795900
C	6.63806400	-2.22765500	-2.28009500
H	5.68347100	-0.51109000	-3.14727600
C	6.49097700	-3.36512400	-1.47446300
H	5.12140900	-4.45929300	-0.20294900
H	7.60820800	-2.03474200	-2.72453800
C	-7.63978500	-4.00271800	-0.58977000
O	-8.47662300	-3.56937100	0.19995200
N	-7.72199100	-5.27020800	-1.09793900
H	-6.93191300	-5.65789900	-1.58786100
C	-7.69982900	4.23679000	-1.28427400
O	-8.83795100	3.81039300	-1.47048600
N	-7.47240100	5.52814400	-0.89448700
H	-6.53581200	5.89710900	-0.92919700
C	7.63985600	4.00291700	-0.58921600
O	8.47664600	3.56955400	0.20054600
N	7.72211700	5.27040200	-1.09740300
H	6.93201100	5.65813300	-1.58725100
C	7.69992100	-4.23668700	-1.28436500
O	8.83805900	-3.81030900	-1.47051800
N	7.47244300	-5.52804000	-0.89458000
H	6.53586600	-5.89701500	-0.92954100
C	8.79237800	6.16571200	-0.67194300
H	8.89418700	6.09775200	0.41734300
H	8.49203500	7.18842500	-0.92105200
C	8.58707700	-6.45445600	-0.72984400
H	9.26520900	-6.34200200	-1.58361000
H	8.18301000	-7.47156500	-0.74966400
C	-8.79227700	-6.16553000	-0.67255400
H	-8.89417600	-6.09752100	0.41672000
H	-8.49187300	-7.18824500	-0.92158500
C	-8.58706100	6.45450400	-0.72965400
H	-9.26524600	6.34199800	-1.58336800
H	-8.18305300	7.47163700	-0.74952200

C	-9.36030600	6.20745100	0.57040200
H	-8.68735500	6.31370400	1.42906400
H	-9.71900300	5.17225400	0.55210200
C	-10.54043900	7.16579200	0.72118200
H	-10.17103500	8.19975000	0.74528600
H	-11.20444500	7.08647700	-0.15604600
C	10.13337700	5.82305900	-1.33025500
H	10.03876400	5.88420200	-2.42054300
H	10.37461900	4.78575700	-1.07263100
C	11.25121600	6.75136000	-0.85851600
H	11.00484100	7.78692400	-1.12875400
H	11.32746100	6.72017700	0.24148000
C	9.36043000	-6.20743700	0.57015400
H	8.68752400	-6.31362100	1.42886100
H	9.71921100	-5.17227200	0.55181200
C	10.54049800	-7.16587200	0.72087300
H	10.17100900	-8.19979900	0.74507400
H	11.20441400	-7.08666500	-0.15643300
C	-10.13322600	-5.82293400	-1.33098600
H	-10.03853400	-5.88408500	-2.42126600
H	-10.37452400	-4.78563500	-1.07339800
C	-11.25106600	-6.75127400	-0.85930900
H	-11.32734400	-6.72011500	0.24068500
H	-11.00465200	-7.78682200	-1.12956400
N	12.50995100	6.39130000	-1.49075100
C	13.68940400	7.05462900	-1.22108100
H	12.61369500	5.40688400	-1.69573900
C	13.73215000	8.28646200	-0.54845100
C	14.91966400	6.51272300	-1.63638000
C	14.97220100	8.90179500	-0.33156900
H	12.82967400	8.74459700	-0.16423200
C	16.10196500	7.20243800	-1.36630300
H	14.94687300	5.54922800	-2.13687600
C	15.02577200	10.22118900	0.36988100
N	16.14178800	8.37940800	-0.71945500
C	17.41182400	6.63517200	-1.80981200
C	14.05928400	11.20677100	0.11270800
N	16.03266200	10.41204600	1.23873400
C	17.53059100	5.99724500	-3.05482300
N	18.45119200	6.77029700	-0.96924100
C	14.13785600	12.42458700	0.78372500
H	13.27928500	11.02634400	-0.61960300
C	16.09343600	11.58431900	1.87453600
C	18.76525100	5.47616900	-3.43386400
H	16.67545100	5.93618000	-3.71982500
C	19.62773800	6.26510300	-1.34716100
C	15.17717200	12.62297700	1.69015700
H	13.40747300	13.20606900	0.59420900
H	16.91987000	11.70218400	2.57377100
C	19.84341400	5.60829800	-2.56136300
H	18.88409400	4.98572700	-4.39579200
H	20.44627000	6.38905300	-0.63975300
H	15.28284300	13.55454700	2.23703200
H	20.82584600	5.22033700	-2.81102800
N	11.25620600	-6.89742700	1.95756200
C	12.38068700	-7.60453000	2.33060200
H	11.24611500	-5.93004200	2.25062700
C	12.76529500	-8.80402400	1.71010700
C	13.19181300	-7.14377100	3.38393500
C	13.91247300	-9.46856600	2.16376000

H	12.21302300	-9.19876100	0.86679400
C	14.31845300	-7.87729400	3.75683700
H	12.95573900	-6.20739300	3.88094300
C	14.32084200	-10.75446300	1.51923000
N	14.68738100	-9.02306800	3.15992900
C	15.18085800	-7.39673100	4.87913800
C	13.35618900	-11.70223600	1.14137100
N	15.63602100	-10.95357200	1.32996700
C	14.60890800	-6.82474200	6.02667600
N	16.50851600	-7.54325900	4.73438900
C	13.77051000	-12.88952600	0.54287200
H	12.30540400	-11.51829100	1.33950500
C	16.01687800	-12.09619100	0.75418600
C	15.44283400	-6.38368300	7.05117900
H	13.53033800	-6.75294500	6.12022900
C	17.29417600	-7.11513900	5.72522700
C	15.13328400	-13.09600800	0.33948600
H	13.04359000	-13.64211800	0.25084700
H	17.08933900	-12.22155300	0.61302400
C	16.82047700	-6.52769300	6.90111100
H	15.02356000	-5.94555300	7.95241100
H	18.36433200	-7.24591500	5.57210400
H	15.50671300	-14.00489200	-0.12153700
H	17.51205500	-6.20094200	7.67116000
N	-12.50980500	-6.39121100	-1.49157200
C	-13.68927900	-7.05448400	-1.22161200
H	-12.61359400	-5.40672900	-1.69628300
C	-13.73198100	-8.28634000	-0.54904800
C	-14.91958400	-6.51243900	-1.63658500
C	-14.97205000	-8.90156900	-0.33190300
H	-12.82946500	-8.74458100	-0.16505200
C	-16.10189700	-7.20203800	-1.36627000
H	-14.94679900	-5.54890100	-2.13700100
C	-15.02557300	-10.22099500	0.36949300
N	-16.14166600	-8.37904400	-0.71947800
C	-17.41180400	-6.63462000	-1.80942300
C	-14.05918700	-11.20662500	0.11212000
N	-16.03231500	-10.41182600	1.23852400
C	-17.53077900	-5.99643000	-3.05428100
N	-18.45102100	-6.76985800	-0.96868500
C	-14.13770500	-12.42445400	0.78311900
H	-13.27931400	-11.02624300	-0.62033700
C	-16.09304200	-11.58411300	1.87430300
C	-18.76548200	-5.47520500	-3.43297200
H	-16.67578000	-5.93528100	-3.71945600
C	-19.62761200	-6.26452700	-1.34627000
C	-15.17686500	-12.62281300	1.68973700
H	-13.40739700	-13.20596800	0.59344900
H	-16.91936100	-11.70195000	2.57368000
C	-19.84348700	-5.60744900	-2.56029200
H	-18.88447900	-4.98455400	-4.39477500
H	-20.44601400	-6.38858600	-0.63873200
H	-15.28248100	-13.55439000	2.23661100
H	-20.82594200	-5.21937300	-2.80968400
N	-11.25599600	6.89736100	1.95798000
C	-12.38067800	7.60426000	2.33088700
H	-11.24588700	5.92994500	2.25096100
C	-12.76544000	8.80367800	1.71035000
C	-13.19182500	7.14335100	3.38413300
C	-13.91279500	9.46800400	2.16387300

H	-12.21314100	9.19852600	0.86710600
C	-14.31864500	7.87666100	3.75691400
H	-12.95562200	6.20702200	3.88117300
C	-14.32134100	10.75381900	1.51929100
N	-14.68772500	9.02236100	3.15995900
C	-15.18107400	7.39594400	4.87913000
C	-13.35683200	11.70178300	1.14154900
N	-15.63653700	10.95266600	1.32987300
C	-14.60913300	6.82406500	6.02672700
N	-16.50874400	7.54222700	4.73424700
C	-13.77131700	12.88899100	0.54300000
H	-12.30603300	11.51804700	1.33980800
C	-16.01755300	12.09520900	0.75404700
C	-15.44308100	6.38285700	7.05114800
H	-13.53055900	6.75246600	6.12038800
C	-17.29442400	7.11396700	5.72500800
C	-15.13410800	13.09520200	0.33945200
H	-13.04451200	13.64172700	0.25106200
H	-17.09002200	12.22035800	0.61275500
C	-16.82073500	6.52661300	6.90094200
H	-15.02381600	5.94480700	7.95242400
H	-18.36458900	7.24454700	5.57177800
H	-15.50766200	14.00401200	-0.12161500
H	-17.51232900	6.19973800	7.67092200

Supplementary Table 15. DFT-optimized geometry of TTF---TPY π-π stacking model (singlet) for TPY-TTF OG, computed at the B3LYP/ 6-31G** (HCNOS) level in absence of any solvent.

Atom	x	y	z
C	2.83708300	0.35273100	-1.03408200
C	2.76122900	1.67112000	-0.73084300
C	-3.57924100	-0.11093400	-1.00401000
S	-2.18774100	2.15917900	-0.67799400
S	-1.94303600	-0.75789300	-1.21148700
S	1.30945000	-0.55268800	-1.08824800
S	1.14324000	2.34668800	-0.44315200
C	-1.09330000	0.77068900	-0.88870600
C	0.25231800	0.85062300	-0.81468400
C	-3.70090300	1.21707400	-0.76250800
C	-4.67062000	-1.09784700	-1.14649600
C	-4.60358600	-2.32647500	-0.46853300
C	-5.78169400	-0.83214300	-1.96555100
C	-5.62666000	-3.26106500	-0.60343000
H	-3.76351100	-2.53340000	0.18571400
C	-6.80378000	-1.76357400	-2.08927300
H	-5.83518800	0.11094800	-2.49815500
C	-6.73263300	-2.99346400	-1.42062900
H	-5.57162500	-4.18453800	-0.03515100
H	-7.67218200	-1.56375200	-2.70728700
C	-7.87373400	-3.95253700	-1.61198500
O	-8.96709700	-3.58190900	-2.02065300
N	-7.63020000	-5.26407400	-1.27833400
H	-8.36555800	-5.91146700	-1.52327500

C	-4.94338500	1.96701800	-0.48731700
C	-5.14534300	3.24424300	-1.03760600
C	-5.93923100	1.43384800	0.35310700
C	-6.30120900	3.96649000	-0.75333800
H	-4.40326300	3.66285400	-1.71056400
C	-7.09480700	2.15263300	0.62596100
H	-5.79633600	0.45125100	0.78618900
C	-7.28455700	3.43359300	0.08980800
H	-6.44647000	4.93287600	-1.22643600
H	-7.87072300	1.74237300	1.26302500
C	3.88685700	2.62721300	-0.65726500
C	4.07485200	3.42353400	0.48330500
C	4.77667600	2.76290300	-1.73604200
C	5.14500300	4.31196400	0.55391000
H	3.39291500	3.31458600	1.31945000
C	5.83984600	3.65376300	-1.66434100
H	4.62384900	2.16256700	-2.62638800
C	6.04424000	4.42830900	-0.51466500
H	5.25636300	4.93736700	1.43469900
H	6.52611000	3.77577200	-2.49513400
C	4.05731400	-0.45180400	-1.24251200
C	4.06874800	-1.49511600	-2.18415300
C	5.21369400	-0.24386200	-0.46636400
C	5.18064800	-2.32053500	-2.32172600
H	3.20103100	-1.65931200	-2.81447100
C	6.32460600	-1.06299600	-0.61156800
H	5.22503900	0.55926400	0.25902900
C	6.31381200	-2.12935300	-1.52257100
H	5.15979800	-3.10167500	-3.07526500
H	7.21466000	-0.91170200	-0.01064100
C	7.53040300	-3.00879500	-1.57090000
O	8.60629400	-2.64850100	-1.10956600
C	7.21443000	5.37148200	-0.51461500
O	7.74580800	5.74059100	-1.55379000
C	-8.55606100	4.15146500	0.44370600
O	-9.53178100	3.55641700	0.88348600
N	7.63764300	5.81802700	0.71606300
H	7.45739000	5.27201800	1.54417900
H	8.49585600	6.35062100	0.70374800
N	-8.57327800	5.50894000	0.22317300
H	-9.38973500	5.98830600	0.57467900
H	-7.71339700	6.03459400	0.20219300
N	7.37275200	-4.23809600	-2.16144200
H	8.16535500	-4.86056300	-2.11027400
H	6.46454800	-4.64444100	-2.31667800
H	-6.68899500	-5.62529000	-1.27844200
C	2.34393000	0.07523400	2.62495700
C	0.94367000	0.14580000	2.68947700
C	0.18019000	-0.98377500	2.38521000
C	2.05638000	-2.22184400	1.93487400
C	2.90698200	-1.15069500	2.24581900
H	0.47021500	1.06686400	3.01260600
H	3.98035600	-1.28130200	2.21344700
C	2.64362400	-3.52598700	1.49587700
C	3.85546200	-3.56445300	0.78654800
C	4.38303300	-4.79648800	0.41052500
H	4.36625700	-2.64979000	0.51681200
C	2.47940500	-5.81475600	1.43610800
C	3.68373600	-5.95507900	0.74055100
H	5.32413300	-4.84227800	-0.13057300

H	1.90210300	-6.69626400	1.71138300
H	4.05277600	-6.93983700	0.47129900
C	-1.30982700	-0.92559200	2.49795100
C	-1.99741000	0.28794300	2.33584300
C	-3.27716700	-2.03520400	2.91330600
C	-3.38051900	0.31283900	2.48163500
H	-1.46754500	1.19123700	2.06084500
C	-4.04429400	-0.87483800	2.78189700
H	-3.75882300	-2.98472800	3.14349300
H	-3.92860100	1.23898900	2.34286500
H	-5.12231100	-0.90896800	2.90414100
N	-1.94966900	-2.07504100	2.77608600
N	0.72179300	-2.15286700	2.00823100
N	1.96387800	-4.64229200	1.81061100
N	3.10849400	1.19176000	2.92702900
H	2.61040400	1.88923000	3.46184100
C	4.51947100	1.07820900	3.25541100
H	4.90095200	2.06874700	3.51148600
H	4.71535900	0.39298500	4.09337400
H	5.08642300	0.72495800	2.39015600

Supplementary Table 16. DFT-optimized geometry of TTF---TTF π-π stacking model (singlet) for TPY-TTF OG, computed at the B3LYP/ 6-31G** (HCNOS) level in absence of any solvent.

Atom	x	y	z
C	0.22248100	-3.41958600	-4.98818300
C	1.55285300	-3.15427200	-4.93568100
S	2.05083500	-1.51168000	-5.44770300
C	0.40586200	-0.84802600	-5.52062800
S	-0.85035400	-2.10535000	-5.52130900
C	0.13871100	0.47369300	-5.59515300
S	1.39505600	1.72971400	-5.65753600
S	-1.50778900	1.13316600	-5.67772000
C	0.29513900	3.08890100	-5.33466800
C	-1.03629800	2.82371900	-5.32504400
C	2.63528600	-4.04437700	-4.46815900
C	2.39358800	-5.02187400	-3.48570200
C	3.94764100	-3.91393500	-4.95868900
C	3.41356600	-5.83853700	-3.02426300
H	1.40362100	-5.12340400	-3.06789500
C	4.97796700	-4.71548200	-4.47128200
H	4.17156100	-3.19280100	-5.73844300
C	4.72504700	-5.68157000	-3.48939800
H	3.22035500	-6.58657500	-2.26320000
H	5.97308100	-4.59621200	-4.88793900
C	-0.44971300	-4.70059300	-4.67550400
C	-0.03909100	-5.90263500	-5.27726500
C	-1.47998700	-4.73538800	-3.72402600
C	-0.60589800	-7.11001000	-4.88752400
H	0.74642600	-5.88287700	-6.02491100
C	-2.04342800	-5.94574000	-3.33454000
H	-1.80026800	-3.81497600	-3.25161400
C	-1.60802800	-7.14714700	-3.90717500

H	-0.27392800	-8.04423000	-5.32663300
H	-2.77659900	-5.94395200	-2.53562400
C	-2.14150600	3.75378100	-5.01384900
C	-3.42065000	3.57696100	-5.57263800
C	-1.95578700	4.82024500	-4.11522200
C	-4.47623100	4.41927600	-5.22827400
H	-3.59796200	2.78409300	-6.29248900
C	-2.99813800	5.67761100	-3.80181000
H	-0.99413200	4.95980300	-3.64497100
C	-4.27921100	5.47411700	-4.32886600
H	-5.44534900	4.25797400	-5.68999600
H	-2.85154600	6.49578700	-3.10545600
C	0.94270000	4.40279700	-5.12469900
C	1.92163100	4.55182500	-4.13096400
C	0.54460900	5.53156400	-5.86161400
C	2.43579100	5.80790900	-3.82684400
H	2.23572900	3.68845900	-3.55787100
C	1.06226000	6.78421900	-5.55737100
H	-0.19832400	5.42182400	-6.64410700
C	2.00337200	6.93917800	-4.52928100
H	3.12355900	5.89938600	-2.99349400
H	0.73422200	7.66489000	-6.09822100
C	5.78320600	-6.54889400	-2.87353000
O	5.50279500	-7.62823800	-2.34192400
N	7.05815400	-6.07737900	-2.91627900
H	7.22440300	-5.11982400	-3.18993700
C	-2.13565900	-8.48958800	-3.48378900
O	-1.48771000	-9.52457100	-3.66934200
N	-3.35170900	-8.49220600	-2.88054500
H	-3.93206700	-7.66879000	-2.94015400
C	-5.37032000	6.38154700	-3.84359900
O	-5.12652500	7.50906900	-3.40101800
N	-6.63538500	5.88717000	-3.87288400
H	-6.79115400	4.91678300	-4.10292100
C	2.46240000	8.33092100	-4.19767000
O	1.79591500	9.32061100	-4.51808400
N	3.63364400	8.43559400	-3.52003700
H	4.24151200	7.63282200	-3.45475800
C	-7.74638200	6.65759000	-3.32643600
H	-7.86209900	7.58174300	-3.90425100
H	-7.50629300	6.95151200	-2.29731600
C	4.16245900	9.72849700	-3.11077300
H	3.34502300	10.28703300	-2.64772600
H	4.50181500	10.29761900	-3.98726800
C	8.15861800	-6.81215500	-2.30509900
H	8.02023900	-7.87239900	-2.53378300
H	8.11485000	-6.71190700	-1.21234900
C	-3.95951800	-9.71575300	-2.37801100
H	-3.19263800	-10.26122500	-1.82213900
H	-4.28801400	-10.35326100	-3.21053700
C	-5.13882500	-9.37104800	-1.47071200
H	-5.85567700	-8.74614500	-2.02092400
H	-4.77965000	-8.78051300	-0.62040000
C	-5.86179300	-10.61836900	-0.96664300
H	-6.25210300	-11.19832600	-1.81684500
H	-5.15357300	-11.26956200	-0.43890900
C	-9.03255000	5.83794400	-3.35712500
H	-8.91055300	4.94359400	-2.73371900
H	-9.22954600	5.49468300	-4.38064400
C	-10.22444800	6.65054400	-2.85397000

H	-9.98861300	7.07482200	-1.87111200
H	-10.41138900	7.49956800	-3.52868400
C	5.30948200	9.54352300	-2.11885800
H	6.10687700	8.94624300	-2.58200900
H	4.95204300	8.99076500	-1.24277900
C	5.87539200	10.89140800	-1.67768000
H	6.20126000	11.47315300	-2.55401400
H	5.08066800	11.46936300	-1.19443700
C	9.49938100	-6.31148600	-2.83884300
H	9.62856400	-5.25349200	-2.57038100
H	9.50328900	-6.36939800	-3.93321400
C	10.66919300	-7.12136900	-2.28230700
H	10.56017500	-8.17150900	-2.58298100
H	10.65453300	-7.10314300	-1.18365700
N	-11.40197800	5.79976600	-2.73363800
C	-12.64054400	6.29926700	-2.40460400
H	-11.41548200	4.99961300	-3.35225000
C	-12.81922200	7.57248500	-1.83599900
C	-13.79814100	5.52611800	-2.61823000
C	-14.10996100	7.98949100	-1.48890900
H	-11.97765400	8.23600200	-1.68629800
C	-15.04392800	6.04466400	-2.27023000
H	-13.71618000	4.55026300	-3.08572000
C	-14.31205800	9.31319500	-0.82429900
N	-15.21189100	7.25736600	-1.71070800
C	-16.28124000	5.24818300	-2.52996500
C	-13.36155500	9.81551500	0.07722900
N	-15.44847800	9.97641200	-1.10979100
C	-16.28640700	3.85403400	-2.37341800
N	-17.37393100	5.93119900	-2.91867900
C	-13.59849700	11.03420800	0.70747900
H	-12.46938500	9.24511000	0.30653500
C	-15.65792100	11.14859300	-0.50126300
C	-17.45563300	3.14587100	-2.63539400
H	-15.39823800	3.33970800	-2.02496200
C	-18.48897900	5.23531100	-3.16908400
C	-14.77336000	11.72237500	0.41386700
H	-12.88701300	11.42800900	1.42601300
H	-16.58515100	11.65940300	-0.75525600
C	-18.58601800	3.84835700	-3.04830400
H	-17.48753200	2.06964300	-2.50110700
H	-19.35384000	5.81641100	-3.48448200
H	-15.00829600	12.67005700	0.88589700
H	-19.52208000	3.34219300	-3.25836600
N	6.96490800	10.70988800	-0.72882700
C	7.52855600	11.74812100	-0.03159900
H	7.56993900	9.91905800	-0.90545900
C	6.94498700	13.02611900	0.02270900
C	8.72363800	11.56275200	0.69188900
C	7.52817300	14.01199600	0.82601700
H	6.06786100	13.25382400	-0.56616000
C	9.24955700	12.61868800	1.43700200
H	9.23834800	10.60969100	0.63455600
C	6.87207800	15.35295000	0.93333500
N	8.66183400	13.82788700	1.51873700
C	10.52564500	12.43989400	2.19783600
C	5.48637600	15.49656000	0.74375300
N	7.66232300	16.40589200	1.21818700
C	10.91440800	11.17961200	2.68010800
N	11.27201700	13.54106900	2.40344600

C	4.91475000	16.76524500	0.81774300
H	4.86043800	14.63052300	0.56351100
C	7.09637000	17.61468000	1.29933500
C	12.10625500	11.05917300	3.38920300
H	10.28033400	10.31282100	2.53299000
C	12.41406500	13.40810300	3.08665100
C	5.73540700	17.85579200	1.09844300
H	3.84658900	16.89391500	0.67401100
H	7.76587200	18.43994400	1.53470300
C	12.88243600	12.19719600	3.59707200
H	12.41377700	10.09639100	3.78471300
H	12.98813500	14.32034400	3.23798400
H	5.33800100	18.86235000	1.17098400
H	13.81079000	12.15708400	4.15525100
N	11.93009700	-6.59837900	-2.78096600
C	13.14791000	-7.05581700	-2.35808900
H	11.91066900	-5.66920500	-3.17608800
C	13.28858500	-8.21033900	-1.56666700
C	14.33392600	-6.40180600	-2.74787300
C	14.56991400	-8.64248100	-1.20918500
H	12.41897400	-8.77273300	-1.25396700
C	15.56511200	-6.91865800	-2.35120800
H	14.28163600	-5.52331300	-3.38259000
C	14.73057900	-9.85629800	-0.35018600
N	15.69778500	-8.02489900	-1.59554200
C	16.82465500	-6.23934300	-2.78299900
C	13.77536700	-10.17824000	0.62665100
N	15.82731700	-10.61160000	-0.55219300
C	16.89056700	-4.84005400	-2.87008800
N	17.87274900	-7.02700900	-3.08835700
C	13.96014300	-11.31057800	1.41446000
H	12.92224100	-9.53111200	0.78973100
C	15.98847700	-11.69804200	0.21107200
C	18.07073100	-4.24115400	-3.29989400
H	16.04187000	-4.23422900	-2.57455200
C	19.00094200	-6.43469800	-3.49856600
C	15.09188800	-12.09520200	1.20445700
H	13.24407300	-11.56216400	2.19006800
H	16.88460900	-12.28639500	0.02063700
C	19.15418400	-5.05409600	-3.62944300
H	18.14802300	-3.16037000	-3.35842400
H	19.82843100	-7.09988600	-3.73977000
H	15.28563300	-12.98198500	1.79812800
H	20.09652900	-4.63449000	-3.96514200
N	-6.93604400	-10.23586300	-0.06009800
C	-7.84605500	-11.12862800	0.44529200
H	-7.26449200	-9.28432100	-0.15055800
C	-7.65397100	-12.51995400	0.38799500
C	-9.01487200	-10.66640900	1.08138100
C	-8.63867900	-13.36750900	0.91309200
H	-6.75248800	-12.92726600	-0.04908300
C	-9.91466300	-11.58730100	1.61422700
H	-9.20867500	-9.60036300	1.13441200
C	-8.47749400	-14.85457300	0.82280300
N	-9.75195900	-12.91948000	1.51178300
C	-11.12791900	-11.11383800	2.34999800
C	-7.65707300	-15.44209700	-0.15488800
N	-9.15826600	-15.59932200	1.71467700
C	-11.11171500	-9.90065700	3.05755500
N	-12.21298700	-11.90970200	2.32688100

C	-7.52445600	-16.82851400	-0.19148500
H	-7.14898300	-14.83203500	-0.89251000
C	-9.02213900	-16.92704100	1.66636300
C	-12.24805600	-9.50403800	3.75701900
H	-10.21534100	-9.29190400	3.08498800
C	-13.29602800	-11.51553900	3.00540200
C	-8.21706000	-17.59578500	0.74234200
H	-6.89565700	-17.29793500	-0.94168500
H	-9.57806000	-17.49037000	2.41364700
C	-13.37115500	-10.32810600	3.73475600
H	-12.24893300	-8.57666400	4.32124600
H	-14.15266200	-12.18595600	2.96769500
H	-8.14160700	-18.67766600	0.75797800
H	-14.27593900	-10.06994200	4.27404700
C	0.33423200	-3.10387500	-0.75692100
C	1.63190200	-2.70161600	-0.73465100
S	1.98841600	-1.08635200	-1.41356500
C	0.29577400	-0.57099800	-1.50519900
S	-0.83648800	-1.93210500	-1.44484500
C	-0.09183500	0.72162100	-1.58094700
S	1.04198900	2.08157800	-1.61288800
S	-1.78383700	1.24386300	-1.61943900
C	-0.15173300	3.32100700	-1.10766900
C	-1.45126200	2.92291200	-1.09970400
C	2.80917000	-3.41867400	-0.20012500
C	2.74987200	-4.08294600	1.03845500
C	4.01777000	-3.43218200	-0.91558400
C	3.85434700	-4.77273900	1.52168900
H	1.82852800	-4.06413800	1.60970000
C	5.13107200	-4.10021500	-0.41499300
H	4.07632900	-2.96406300	-1.89278600
C	5.06008800	-4.78123400	0.80596300
H	3.80200600	-5.30706000	2.46386300
H	6.03642500	-4.13695200	-1.01109400
C	-0.23696200	-4.42099400	-0.40232100
C	0.48445600	-5.61377100	-0.60565700
C	-1.55546700	-4.52447900	0.07973400
C	-0.08891200	-6.84946800	-0.34691000
H	1.49972300	-5.56935400	-0.97111400
C	-2.12539700	-5.76624700	0.35299400
H	-2.14243000	-3.62838900	0.25077600
C	-1.39695000	-6.94431000	0.14710800
H	0.46788100	-7.76148800	-0.53015000
H	-3.15154400	-5.80609600	0.70473500
C	-2.65043100	3.68650200	-0.69390600
C	-3.83289600	3.60471500	-1.44801500
C	-2.64243900	4.48671700	0.46285600
C	-4.97029600	4.30906800	-1.06489500
H	-3.85251600	3.02758700	-2.36671300
C	-3.76989700	5.21221200	0.82588100
H	-1.74324300	4.54333900	1.06593600
C	-4.94958200	5.12375100	0.07315600
H	-5.85419100	4.26474400	-1.69186500
H	-3.75645800	5.84948100	1.70314300
C	0.41389300	4.66553500	-0.86112200
C	1.70857200	4.81045900	-0.32711300
C	-0.28575000	5.83643400	-1.21291500
C	2.27057900	6.07063500	-0.13500900
H	2.28109400	3.93290900	-0.04651600
C	0.27997900	7.09012000	-1.03270700

H	-1.28159500	5.76199800	-1.62393900
C	1.55843500	7.22758800	-0.47545100
H	3.27742800	6.13957700	0.26468600
H	-0.26309400	7.98303700	-1.32133800
C	6.21290300	-5.55788000	1.37142100
O	6.03734400	-6.53343700	2.10735000
N	7.45887600	-5.13391400	1.02082900
H	7.56442700	-4.20740900	0.63219600
C	-1.93948500	-8.31633500	0.42230200
O	-1.49920600	-9.31093900	-0.16243100
N	-2.93137200	-8.40281500	1.35333700
H	-3.08030600	-7.61604600	1.96977500
C	-6.12871700	5.94116300	0.51281900
O	-5.98585000	6.99179500	1.14566700
N	-7.36040800	5.46462800	0.18070200
H	-7.44599800	4.51163600	-0.14283600
C	2.07767500	8.61976700	-0.25658600
O	1.67412500	9.57273000	-0.93001700
N	2.99868500	8.76839400	0.73786700
H	3.09810700	8.01549900	1.40501400
C	-8.56445300	6.14314300	0.64984700
H	-8.67699300	7.09032900	0.10768200
H	-8.43822600	6.40511600	1.70689000
C	3.42634900	10.09078600	1.18364000
H	2.64253200	10.55189500	1.80114400
H	3.54101000	10.71766200	0.29711800
C	8.64523600	-5.78215100	1.56772500
H	8.56229300	-6.85656600	1.37444900
H	8.67893900	-5.65886100	2.65834200
C	-3.41909300	-9.70100800	1.80418400
H	-2.68174100	-10.17470700	2.46714300
H	-3.50413700	-10.34204200	0.92382500
C	-4.76425900	-9.57359500	2.51625600
H	-5.48438900	-9.08222000	1.85290800
H	-4.65724400	-8.95288200	3.41470800
C	-5.29742500	-10.94881900	2.91135800
H	-5.44132400	-11.56485900	2.01068300
H	-4.55467500	-11.46140100	3.53544200
C	-9.79958100	5.26492100	0.46322400
H	-9.69469800	4.34752000	1.05406700
H	-9.89764800	4.96777600	-0.58761300
C	-11.06717700	6.00127800	0.89197000
H	-10.97120700	6.31063700	1.93957700
H	-11.18656000	6.91979000	0.29656700
C	4.73544900	10.00056100	1.96548600
H	5.53601400	9.67407100	1.29152600
H	4.64359400	9.25088300	2.76186000
C	5.11326500	11.34030300	2.59857700
H	5.18778900	12.11918400	1.82560700
H	4.32547000	11.64989400	3.29671100
C	9.91186500	-5.21693600	0.92981800
H	10.01165100	-4.15282900	1.17981500
H	9.83498300	-5.28480700	-0.16013400
C	11.15548000	-5.96952900	1.39889500
H	11.04315900	-7.03844000	1.17218300
H	11.25136900	-5.88547500	2.49222700
N	-12.23037100	5.13862100	0.76346500
C	-13.49191300	5.55819000	1.10733200
H	-12.18930100	4.46670700	0.00855300
C	-13.73359000	6.77742800	1.76414200

C	-14.61344700	4.75379500	0.82599300
C	-15.04766900	7.13817400	2.07725400
H	-12.92394600	7.46183000	1.97470200
C	-15.88309100	5.19135900	1.19607200
H	-14.48366500	3.81260800	0.30301600
C	-15.31235700	8.45258200	2.73930000
N	-16.11431200	6.37077700	1.80400400
C	-17.07431700	4.32840200	0.93223600
C	-14.40499200	8.98734600	3.66767400
N	-16.44868700	9.08973200	2.40063200
C	-16.98984900	2.93383700	1.06421100
N	-18.21631800	4.94860600	0.58022600
C	-14.67603500	10.22357800	4.24890000
H	-13.51375300	8.43469200	3.94328700
C	-16.69195900	10.27762300	2.96489000
C	-18.11949300	2.15807400	0.81430700
H	-16.06082400	2.47042000	1.37739100
C	-19.28973000	4.18854600	0.33866600
C	-15.84469700	10.89185800	3.88899100
H	-13.98979400	10.65204400	4.97290000
H	-17.61305500	10.77063600	2.65931300
C	-19.30004500	2.79581700	0.43940600
H	-18.07843300	1.07806400	0.91741200
H	-20.19265200	4.71912100	0.04258200
H	-16.09904600	11.85851200	4.31049000
H	-20.20595100	2.23678000	0.23073500
N	6.36475700	11.20416200	3.32490000
C	6.93631000	12.19733300	4.06958900
H	6.98506400	10.47097500	3.01216700
C	6.30630500	13.43084500	4.31988200
C	8.20547900	12.00085200	4.65130600
C	6.96343900	14.38076100	5.10887900
H	5.32857100	13.64735500	3.90916500
C	8.77373800	13.00707300	5.42718700
H	8.74338500	11.07899000	4.46433300
C	6.31853200	15.70432500	5.37071000
N	8.17154300	14.18886400	5.66229800
C	10.11999100	12.80747100	6.04550600
C	5.58648500	16.35093200	4.36467600
N	6.48890800	16.24177500	6.59438900
C	10.52502800	11.54071200	6.49510200
N	10.91021300	13.89212300	6.15661200
C	5.00827200	17.58889000	4.63353100
H	5.49744300	15.90429100	3.38283200
C	5.92249500	17.42975800	6.83765700
C	11.78756900	11.39152900	7.06309200
H	9.85342500	10.69303900	6.41939500
C	12.12139800	13.73082800	6.70090300
C	5.17384700	18.14538500	5.90053800
H	4.45226600	18.11030600	3.86083200
H	6.07326900	17.83442400	7.83711600
C	12.61228600	12.50992000	7.16730500
H	12.11717600	10.42123300	7.42161800
H	12.73765800	14.62588000	6.76636600
H	4.74412300	19.10727600	6.15942300
H	13.60486000	12.44544100	7.60027500
N	12.34456300	-5.45758600	0.73450600
C	13.61112500	-5.82933700	1.09775400
H	12.25430600	-4.55933600	0.28171900
C	13.85895400	-6.93644000	1.92923300

C	14.73527400	-5.12370000	0.62527400
C	15.17924600	-7.28558400	2.23076300
H	13.04124800	-7.54057500	2.29786800
C	16.01163100	-5.53560100	1.00175800
H	14.60104700	-4.28216200	-0.04579500
C	15.44965100	-8.48067600	3.08838500
N	16.24737500	-6.60604400	1.78491600
C	17.20812900	-4.76324300	0.54810300
C	14.56027800	-8.85556200	4.10819800
N	16.57275900	-9.17581100	2.82934600
C	17.15533400	-3.36585400	0.42909300
N	18.32741400	-5.46331800	0.28152600
C	14.83226500	-9.99342300	4.86386500
H	13.68167400	-8.25608800	4.31842500
C	16.81842800	-10.26652800	3.56241100
C	18.29165900	-2.67521000	0.01373100
H	16.24720800	-2.82973300	0.68121000
C	19.40701400	-4.78592300	-0.12114000
C	15.98552200	-10.72427200	4.58513800
H	14.15879700	-10.29774700	5.65920400
H	17.72857600	-10.81071400	3.31687900
C	19.44742600	-3.39809800	-0.27197500
H	18.27440000	-1.59350700	-0.07762100
H	20.28924800	-5.38283700	-0.34382300
H	16.23943500	-11.61951300	5.14267000
H	20.35679000	-2.90789200	-0.60270300
N	-6.53848200	-10.83086900	3.66065700
C	-7.23287900	-11.92942600	4.09848400
H	-7.09494100	-10.00948500	3.46689800
C	-6.70028700	-13.23003300	4.04264800
C	-8.52007100	-11.79321600	4.65407300
C	-7.46019900	-14.30208000	4.51915600
H	-5.71409500	-13.40656800	3.63533200
C	-9.19281800	-12.92256200	5.11680300
H	-8.99248000	-10.81839400	4.68766400
C	-6.91836900	-15.69097600	4.40975100
N	-8.67971700	-14.16629400	5.06392200
C	-10.56846100	-12.78819300	5.68667000
C	-6.13851300	-16.06459000	3.30380500
N	-7.22754400	-16.55713800	5.39355400
C	-10.92673600	-11.66352800	6.44621600
N	-11.43512400	-13.78839600	5.43617900
C	-5.65999500	-17.36865900	3.21847800
H	-5.95249500	-15.35897600	2.50275100
C	-6.75753500	-17.80681700	5.29694000
C	-12.22087900	-11.56733200	6.95083800
H	-10.19717300	-10.88822600	6.65160600
C	-12.67501700	-13.67902200	5.92650300
C	-5.97020100	-18.26571300	4.23938300
H	-5.07132700	-17.68097600	2.36171200
H	-7.02302700	-18.48050100	6.11003100
C	-13.12248000	-12.59596700	6.68508500
H	-12.51655800	-10.70708800	7.54342300
H	-13.35205000	-14.50059900	5.69863600
H	-5.62341900	-19.29339900	4.21751400
H	-14.14243200	-12.56629000	7.05337900

Supplementary Table 17. DFT-optimized geometry of TTF---Zn(TPY)$_2$ π-π stacking model (singlet) for Zn-TPY-TTF CPG, computed at the B3LYP-D3/ LANL2DZ (Zn)/ 6-31G** (HCNOS) level in absence of any solvent.

Atom	x	y	z
C	-3.67730300	8.73001800	0.04701800
H	-2.70876200	8.99710900	-0.39105600
H	-4.30245500	8.26574900	-0.72428400
C	-1.61202200	-3.40696800	-1.47044400
C	-1.76406400	-2.23094300	-2.13740400
S	-0.26313400	-1.40818700	-2.63444100
C	0.82518800	-2.39389200	-1.64180000
S	0.07193500	-3.91308600	-1.12009200
C	2.09067100	-2.00387000	-1.36989700
S	2.73696200	-0.45347300	-1.95671500
S	3.27823400	-2.96494600	-0.46497600
C	4.37261400	-0.64257000	-1.27346800
C	4.61151800	-1.78660800	-0.58632600
C	-2.66016600	-4.32513600	-0.97028000
C	-2.46498600	-5.71720400	-0.99073400
C	-3.86635600	-3.83524700	-0.43444100
C	-3.44209300	-6.58429200	-0.49951200
H	-1.54996900	-6.13238500	-1.40228800
C	-4.83776100	-4.69718400	0.05192800
C	-4.63963300	-6.08508200	0.02496500
H	-3.24728400	-7.65229700	-0.50116300
H	-5.75874300	-4.32014100	0.48512100
C	-3.00626200	-1.52139200	-2.51234200
C	-3.06607700	-0.12310700	-2.37234500
C	-4.15835000	-2.19631800	-2.95932300
C	-4.24822600	0.56978000	-2.61140600
H	-2.19685600	0.42073800	-2.02454200
C	-5.36034000	-1.51518700	-3.11053600
C	-5.41794600	-0.12673700	-2.92760600
H	-4.28105000	1.64517000	-2.47160300
H	-6.26712100	-2.05361100	-3.36605700
C	5.27462100	0.51302300	-1.47840000
C	5.24073000	1.23523100	-2.68403200
C	6.13673700	0.96103800	-0.45750400
C	6.03267400	2.36911300	-2.86072000
H	4.60073200	0.90276300	-3.49581300
C	6.90813800	2.10179100	-0.62830500
C	6.86803800	2.81822300	-1.83255400
H	5.97073200	2.92022500	-3.79414000
H	7.54930100	2.46600800	0.16742300
C	5.88930300	-2.19594200	0.03384700
C	7.08639300	-2.13108100	-0.70097700
C	5.93149200	-2.63631400	1.36671100
C	8.29402100	-2.46806900	-0.10483200
C	7.14438500	-2.98979700	1.95683100
H	5.01504200	-2.68584900	1.94879900
C	8.33543200	-2.90880900	1.22537300
H	9.22816200	-2.39696900	-0.65142400
H	7.16222200	-3.29084300	2.99972600
C	-5.70583700	-6.94202500	0.65756800
O	-6.44825100	-6.47484600	1.51253900

N	-5.75928800	-8.24702500	0.25687800
H	-5.36540500	-8.54370200	-0.62254900
C	9.69267500	-3.21959500	1.80494200
O	10.70448900	-2.72558300	1.32877400
N	9.71316000	-4.04302800	2.90155400
H	8.96519900	-4.69905400	3.06642500
C	7.68630200	4.08000600	-1.91583400
O	7.96094000	4.71527000	-0.90677800
N	8.05535100	4.48222100	-3.17318600
H	8.12597800	3.81241700	-3.92435800
C	-6.75153300	0.56255400	-2.89964200
O	-7.68260400	0.10039100	-2.25079500
N	-6.82076800	1.76643700	-3.55102400
H	-6.17902000	1.95955500	-4.30623000
H	7.05855500	-1.80431700	-1.73475800
H	6.20563700	0.40747800	0.47047100
H	-4.03459000	-2.76883500	-0.40959700
H	-4.11953000	-3.26529100	-3.13152100
N	-3.51467800	7.85064500	1.19644000
C	-2.92949700	6.63434900	1.15848200
H	-3.83697400	8.18795200	2.09099300
C	-2.42749200	6.07974600	-0.04631400
C	-2.80027900	5.85773600	2.33985300
C	-1.83581700	4.82398300	-0.01612200
H	-2.51140500	6.62977400	-0.97297900
C	-2.20242000	4.61039300	2.27834900
H	-3.18114400	6.25048300	3.27530700
C	-1.23756500	4.16318600	-1.21429700
N	-1.73681200	4.10472600	1.11692700
C	-2.02066400	3.71811800	3.46172900
C	-1.01544400	4.81834000	-2.42857800
N	-0.85899300	2.87706100	-1.03715100
Zn	-1.24744300	2.01169500	0.98835100
C	-2.29055400	4.11546100	4.77473900
N	-1.56009700	2.47788100	3.18822400
C	-0.38665600	4.13189900	-3.46817500
H	-1.30245500	5.85447800	-2.56089400
C	-0.23062300	2.22957700	-2.02688600
N	0.90413800	1.44220600	1.12016400
N	-1.24348300	-0.09358600	1.26849300
N	-3.40260800	1.34704300	0.77664400
C	-2.08772000	3.21004200	5.81527700
H	-2.64219000	5.11647000	4.99288400
C	-1.36846500	1.61085200	4.18958200
C	0.02232800	2.81502800	-3.26587300
H	-0.20564400	4.62875000	-4.41598600
H	0.07792500	1.21287300	-1.80994900
C	1.12973400	0.15310500	1.45956400
C	1.94201500	2.28238200	0.99762100
C	-0.07797500	-0.71431000	1.55057300
C	-2.40871900	-0.75547800	1.40710400
C	-3.61871300	0.03641600	1.03163800
C	-4.43231400	2.12121400	0.40996200
C	-1.61908500	1.93015700	5.52246200
H	-2.29021400	3.50405700	6.84009100
H	-1.00427900	0.62948900	3.90111100
H	0.52936700	2.24969000	-4.03960300
C	2.41994300	-0.30198400	1.73911400
C	3.25790800	1.89630000	1.23732500
H	1.69506100	3.29756000	0.70067500

C	-0.03530400	-2.04097600	1.93722100
C	-2.45841200	-2.06559200	1.86347400
C	-4.89593900	-0.52079900	0.90611900
C	-5.72477400	1.63217700	0.23973500
H	-4.19595000	3.16658900	0.23233300
H	-1.44837300	1.19760000	6.30323100
C	3.49061300	0.58000400	1.63440200
H	2.59707000	-1.33252900	2.01522500
H	4.07281200	2.59978700	1.11207100
C	-1.24612700	-2.75816800	2.11417600
H	0.90807900	-2.54185400	2.11580400
H	-3.40220700	-2.57158900	2.00027300
C	-5.95612600	0.27861800	0.48127400
H	-5.06434500	-1.57059100	1.11002700
H	-6.52132000	2.28412700	-0.09952700
H	4.49416500	0.23379800	1.84992200
N	-1.21886200	-4.04188300	2.52551900
H	-6.93661300	-0.14883300	0.30273200
H	-0.31272900	-4.47368600	2.62396300
C	-2.39037200	-4.86317900	2.80543900
H	-2.05318600	-5.82593400	3.18938200
H	-3.02394900	-4.39644400	3.56751500
H	-2.98314600	-5.03633000	1.90560000
H	10.63298100	-4.31708000	3.21899900
H	8.68883300	5.26932300	-3.20893700
H	-6.52179000	-8.79614900	0.62889000
H	-7.74344000	2.17526300	-3.62346700
H	-4.16721300	9.64649600	0.37555600

Supplementary Table 18. Structure of TTF---TTF π-π stacking model (singlet) for Zn-TPY-TTF CPG.

Atom	x	y	z
C	2.77599000	-1.51283900	-7.83096200
C	3.18920000	-0.22691700	-7.80613900
S	1.99331500	1.01795100	-8.26113400
C	0.60328100	-0.09449700	-8.27773700
S	1.08608000	-1.80828500	-8.31976400
C	-0.68278500	0.32180200	-8.25303500
S	-1.16002200	2.03627300	-8.20021800
S	-2.07640600	-0.78616000	-8.25901800
C	-2.84017800	1.72350100	-7.69007600
C	-3.25825000	0.43864400	-7.71826100
C	4.53443400	0.24446000	-7.41294000
C	5.11807300	-0.21804900	-6.22126200
C	5.23978700	1.17157200	-8.19817900
C	6.37317800	0.22555200	-5.83317700
H	4.57516500	-0.92362500	-5.60231200
C	6.49772400	1.62311300	-7.79931300
H	4.81393700	1.52527000	-9.13197800
C	7.07273700	1.15992000	-6.60662300
H	6.82706100	-0.13391200	-4.91638200
H	7.03398000	2.31512800	-8.44057000

C	3.61680100	-2.70154200	-7.57881300
C	4.88981100	-2.78181500	-8.16790100
C	3.16895500	-3.77366600	-6.78605400
C	5.69628600	-3.88629400	-7.95737000
H	5.23745900	-1.96898300	-8.79537300
C	3.97951700	-4.89469300	-6.58954800
H	2.19440100	-3.72388100	-6.31117000
C	5.25109000	-4.96186300	-7.18067900
H	6.68106000	-3.94412100	-8.40744900
H	3.62227100	-5.69471200	-5.94921900
C	-4.59559600	-0.04978300	-7.31840100
C	-5.31432200	-0.94174400	-8.13141000
C	-5.15743100	0.35666100	-6.09587000
C	-6.56273200	-1.41531100	-7.72973800
H	-4.90543800	-1.25177100	-9.08803300
C	-6.40517200	-0.10712500	-5.70538300
H	-4.60414000	1.03458700	-5.45557300
C	-7.11674600	-1.00837700	-6.50726900
H	-7.10907000	-2.07954300	-8.39153800
H	-6.84266600	0.20905300	-4.76498100
C	-3.66405400	2.90562400	-7.36190000
C	-3.18248800	3.92764200	-6.52476200
C	-4.94975000	3.03405800	-7.91497300
C	-3.97092300	5.04607000	-6.24778600
H	-2.19793300	3.83918100	-6.07691300
C	-5.73487000	4.13754700	-7.62544300
H	-5.32425400	2.26071700	-8.57601200
C	-5.25469700	5.16310200	-6.80205400
H	-3.58652600	5.80471200	-5.57371600
H	-6.72934500	4.23314400	-8.04684500
C	8.42111900	1.59806500	-6.10884900
O	9.05753600	0.91470300	-5.30496900
N	8.91471400	2.76799500	-6.60244200
H	8.30826300	3.48197600	-6.97571800
C	6.19772200	-6.11883600	-7.02677600
O	7.40790900	-5.97250500	-7.20659100
N	5.67499400	-7.32590200	-6.67171000
H	4.71160000	-7.56445200	-6.84989500
C	-8.45484900	-1.47592000	-6.00798100
O	-9.08321500	-0.83232600	-5.16585600
N	-8.94610600	-2.62873000	-6.54293100
H	-8.33716600	-3.32048700	-6.95225200
C	-6.17509800	6.32539100	-6.55575000
O	-7.39240700	6.21434100	-6.71100700
N	-5.61411300	7.49627600	-6.14188200
H	-4.64947700	7.72068400	-6.33133400
C	-10.25126800	-3.09044000	-6.05105600
H	-10.98971000	-2.33663700	-6.24888200
H	-10.19452400	-3.26855800	-4.99327400
C	-6.53451200	8.61872100	-5.91488300
H	-7.23954700	8.34867600	-5.15156600
H	-7.05696400	8.83662300	-6.82777800
C	10.23009200	3.19610300	-6.10791600
H	10.95843700	2.44328700	-6.34335200
H	10.18524500	3.33224400	-5.04335200
C	6.63263300	-8.43231800	-6.54101000
H	7.35008700	-8.18912900	-5.78024300
H	7.13481200	-8.57429700	-7.47987600
C	5.90118700	-9.73137000	-6.15878800
H	5.18401800	-9.97867900	-6.91930300

H	5.38987000	-9.60036900	-5.22339200
C	6.93366000	-10.86649700	-6.03566900
H	7.43935600	-10.98477200	-6.97582600
H	7.64510700	-10.61895000	-5.27001900
C	-10.63842700	-4.39550100	-6.76908600
H	-9.89334700	-5.14430800	-6.57407500
H	-10.69592100	-4.21989100	-7.82710800
C	-12.00578800	-4.88532100	-6.25856100
H	-11.95174800	-5.06854900	-5.20160300
H	-12.74848500	-4.13489600	-6.45579600
C	-5.74875100	9.86550700	-5.47037700
H	-5.04369700	10.13728000	-6.23386600
H	-5.21869500	9.65503500	-4.56026100
C	-6.73244200	11.02757700	-5.24249500
H	-7.25835300	11.22887300	-6.15710700
H	-7.43293800	10.75660000	-4.47468400
C	10.62722000	4.52274300	-6.77905000
H	9.89483300	5.27444900	-6.55006600
H	10.67294700	4.38583200	-7.84334200
C	12.00479200	4.97341300	-6.26048300
H	12.73592000	4.22080400	-6.48998200
H	11.95812500	5.11739600	-5.19712300
N	-12.36605600	-6.12963600	-6.95266300
C	-13.53674000	-6.73556200	-6.65559400
H	-11.71920900	-6.47878300	-7.63285500
C	-14.41792400	-6.19581000	-5.67964000
C	-13.93151900	-7.93045200	-7.30649400
C	-15.60973800	-6.84553600	-5.40668300
H	-14.14575000	-5.28309700	-5.16601000
C	-15.14309700	-8.51542700	-6.97226200
H	-13.29271600	-8.37146600	-8.05822000
C	-16.60882400	-6.36530000	-4.40493000
N	-15.96030300	-7.98134600	-6.04495300
C	-15.65127800	-9.77189900	-7.60048400
C	-16.42835600	-5.22136100	-3.62161900
N	-17.72856600	-7.11442900	-4.29236700
Zn	-17.82919100	-8.90212000	-5.62658400
C	-14.93997600	-10.50008300	-8.55874100
N	-16.86791100	-10.18112200	-7.17983900
C	-17.42260900	-4.85433700	-2.71618100
H	-15.53091900	-4.62229800	-3.70925600
C	-18.68180200	-6.75992000	-3.42242500
N	-19.25876100	-7.94297100	-7.04765800
N	-19.71977600	-9.77996700	-5.21304700
N	-17.46157000	-10.37232900	-3.98742600
C	-15.50257700	-11.66282300	-9.08345700
H	-13.96287900	-10.17569000	-8.89377700
C	-17.40577600	-11.29809000	-7.68417400
C	-18.57411700	-5.63375100	-2.60979900
H	-17.29610600	-3.96873300	-2.10231800
H	-19.55344500	-7.40673300	-3.38694300
C	-20.54243700	-8.35316400	-6.95003500
C	-18.92266500	-7.01973800	-7.95665100
C	-20.81057500	-9.38422800	-5.90353600
C	-19.83348400	-10.70526100	-4.24267900
C	-18.54924300	-11.05247000	-3.56253000
C	-16.26601300	-10.61991500	-3.43859200
C	-16.75919100	-12.07534900	-8.64247500
H	-14.96195900	-12.23826000	-9.82754200
H	-18.38510200	-11.56654900	-7.29920200

H	-19.36933700	-5.38008800	-1.91842300
C	-21.53041700	-7.82820700	-7.78839100
C	-19.85047400	-6.44972900	-8.82520500
H	-17.87432300	-6.73728900	-7.97828800
C	-22.06460800	-9.90601500	-5.64158400
C	-21.05313600	-11.27962100	-3.91413300
C	-18.44580800	-12.01579000	-2.55534900
C	-16.08454200	-11.56369100	-2.42935700
H	-15.43695200	-10.03788000	-3.83001600
H	-17.22924600	-12.97343700	-9.02596000
C	-21.17787500	-6.86684400	-8.73423800
H	-22.55855100	-8.15873100	-7.71276100
H	-19.53772500	-5.70463600	-9.54742700
C	-22.21521600	-10.88268500	-4.62244700
H	-22.93669000	-9.58337700	-6.19703400
H	-21.12408100	-12.01737300	-3.12822400
C	-17.20026700	-12.27207400	-1.98463900
H	-19.31583400	-12.56498600	-2.21838800
H	-15.09924100	-11.73439300	-2.01174400
H	-21.93403000	-6.45069500	-9.39160400
N	-23.42998600	-11.40434400	-4.34999400
H	-17.11113100	-13.01950500	-1.20280900
H	-24.21496700	-11.07343600	-4.89044600
C	-23.68755800	-12.40625900	-3.32806400
H	-24.75136500	-12.64212900	-3.33575300
H	-23.12709600	-13.32796900	-3.52223000
H	-23.42161800	-12.03798900	-2.33049900
N	12.38172300	6.23626400	-6.91049200
C	13.56300200	6.81100500	-6.59474600
H	11.73886800	6.62008200	-7.57562400
C	14.43371400	6.22198200	-5.63824900
C	13.97950600	8.02036100	-7.20409400
C	15.63596300	6.84076200	-5.34172400
H	14.14464300	5.29722600	-5.15638500
C	15.20129600	8.57143600	-6.84951200
H	13.34928100	8.49850700	-7.94019400
C	16.62436900	6.30887300	-4.35563500
N	16.00735100	7.99149100	-5.94007900
C	15.73297300	9.83940600	-7.43371400
C	16.42250300	5.14195200	-3.61247400
N	17.75634100	7.03424700	-4.21527600
Zn	17.89094900	8.86442700	-5.48715900
C	15.03654700	10.61257900	-8.36733400
N	16.95579100	10.21245700	-6.99740200
C	17.40809900	4.72711100	-2.71825900
H	15.51515300	4.56176800	-3.72224800
C	18.70123400	6.63392700	-3.35616800
N	19.30815700	7.92939900	-6.93631000
N	19.79479600	9.69529000	-5.03921700
N	17.54305400	10.28434900	-3.80104900
C	15.62060700	11.78255000	-8.85092400
H	14.05455900	10.31722900	-8.71470600
C	17.51416500	11.33651500	-7.46212700
C	18.57240400	5.48260100	-2.58287800
H	17.26500000	3.82333900	-2.13532300
H	19.58366600	7.26415700	-3.29685300
C	20.59836200	8.31411300	-6.82152100
C	18.95925100	7.04329600	-7.87686400
C	20.88078600	9.30455800	-5.73996400
C	19.92086000	10.58546600	-4.03765400

C	18.63993400	10.93203900	-3.35023400
C	16.34927800	10.53288400	-3.24925500
C	16.88334000	12.15748300	-8.39412500
H	15.09172300	12.39268200	-9.57554900
H	18.49718900	11.57436300	-7.06659800
H	19.36141400	5.19185600	-1.89902100
C	21.57987700	7.80094900	-7.67464100
C	19.87997500	6.48719700	-8.76181900
H	17.90633100	6.77970700	-7.91072800
C	22.14263300	9.79569400	-5.45708400
C	21.14905000	11.12748400	-3.68675000
C	18.54725100	11.86440100	-2.31286600
C	16.17811300	11.44686200	-2.21180200
H	15.51278700	9.97748100	-3.66293100
H	17.36986300	13.05982900	-8.74575200
C	21.21398800	6.87825800	-8.65337600
H	22.61324600	8.11108700	-7.58525200
H	19.55688900	5.77238800	-9.50964500
C	22.30650200	10.73462200	-4.40496700
H	23.01092900	9.47714300	-6.02076000
H	21.23003600	11.83711100	-2.87629800
C	17.30257600	12.12303400	-1.74047400
H	19.42412400	12.38920300	-1.95537000
H	15.19352700	11.62088000	-1.79384700
H	21.96501600	6.47169100	-9.32250900
N	23.52915400	11.22570500	-4.11169900
H	17.21782600	12.84873200	-0.93816300
H	24.31009000	10.89982300	-4.66099200
C	23.80060600	12.18767700	-3.05555800
H	24.86832600	12.40526400	-3.05242600
H	23.25667600	13.12500400	-3.21964200
H	23.52522200	11.79025300	-2.07180800
N	-5.99932800	12.23354500	-4.83148800
C	-6.69977200	13.36382300	-4.59033500
H	-5.00348200	12.16555700	-4.75015400
C	-8.11379600	13.38057600	-4.72169700
C	-6.06228700	14.56918300	-4.20140500
C	-8.80717100	14.55004500	-4.46914700
H	-8.62949000	12.47551700	-5.01455000
C	-6.83489900	15.69946500	-3.97262000
H	-4.98823700	14.59937700	-4.08652900
C	-10.29197800	14.67200200	-4.57305100
N	-8.17510000	15.68420600	-4.10280000
C	-6.26657700	17.02058400	-3.56597600
C	-11.12830100	13.62012300	-4.95858100
N	-10.79929000	15.88594100	-4.26710500
Zn	-9.30270000	17.43542600	-3.68207800
C	-4.89774100	17.25885500	-3.41050400
N	-7.17068700	18.00325800	-3.35487700
C	-12.50408500	13.83510000	-5.02339100
H	-10.72203600	12.64786800	-5.20690900
C	-12.12058500	16.09028500	-4.33010200
N	-9.78212900	17.29092300	-1.50595300
N	-10.46019900	19.16102400	-3.23724600
N	-9.45674200	18.56294400	-5.60008600
C	-4.46837900	18.52969800	-3.03047000
H	-4.17189600	16.47445300	-3.58381600
C	-6.75589600	19.22257700	-2.99108700
C	-13.01688900	15.09173900	-4.70384900
H	-13.16520000	13.02800000	-5.32105500

H	-12.45738800	17.08943900	-4.07024100
C	-10.53115700	18.29832300	-1.00582800
C	-9.37923300	16.29895400	-0.70289300
C	-10.92959100	19.35248800	-1.98601100
C	-10.75071200	20.04407500	-4.21082300
C	-10.16553200	19.71218900	-5.54514100
C	-8.89179300	18.18367100	-6.75245300
C	-5.40989100	19.53526600	-2.81479100
H	-3.40913000	18.72907600	-2.90664000
H	-7.53707600	19.96182700	-2.84033000
H	-14.08024000	15.29761900	-4.74235300
C	-10.89145200	18.32343700	0.34478200
C	-9.69948800	16.25405000	0.65195700
H	-8.78175000	15.52340200	-1.17283700
C	-11.71555700	20.44439500	-1.66434000
C	-11.53333400	21.16540900	-3.97558700
C	-10.31864900	20.51651600	-6.67824800
C	-9.00162400	18.93083300	-7.92308500
H	-8.33693100	17.25063800	-6.72207900
H	-5.11507100	20.53554400	-2.51959800
C	-10.46971500	17.28975200	1.17944800
H	-11.48879500	19.13187400	0.74671300
H	-9.35348600	15.43281700	1.26870000
C	-12.04061200	21.39108600	-2.67140800
H	-12.09183900	20.59200500	-0.65943900
H	-11.76015700	21.85910600	-4.77194700
C	-9.73003600	20.11875800	-7.87794800
H	-10.88239700	21.43981600	-6.63482300
H	-8.52905600	18.58861600	-8.83639300
H	-10.74179300	17.29620800	2.22978400
N	-12.80785500	22.46270100	-2.38006800
H	-9.84024100	20.73314900	-8.76533300
H	-13.13849700	22.55968500	-1.43193200
C	-13.19634900	23.48452000	-3.33912500
H	-13.80798400	24.22575900	-2.82544900
H	-12.32009700	23.99046400	-3.76050800
H	-13.78434600	23.05934200	-4.16071000
N	6.26318900	-12.12740100	-5.68847900
C	7.02025600	-13.23751500	-5.54488200
H	5.26815800	-12.10976400	-5.57649100
C	8.42891500	-13.17586600	-5.71397000
C	6.45185700	-14.49650900	-5.22475100
C	9.18406800	-14.32375800	-5.56158300
H	8.89239500	-12.22855100	-5.95603200
C	7.28388700	-15.60050000	-5.09600200
H	5.38426700	-14.58736600	-5.08443600
C	10.66932500	-14.36412900	-5.71169500
N	8.61763600	-15.51005200	-5.25923300
C	6.79148600	-16.97310500	-4.76863300
C	11.44276800	-13.24796600	-6.04410600
N	11.24279600	-15.56922000	-5.50413000
Zn	9.83955000	-17.22757500	-4.99080100
C	5.44070600	-17.28928300	-4.59537200
N	7.74741400	-17.92181500	-4.65002000
C	12.82483500	-13.38918800	-6.15873400
H	10.98336100	-12.28245400	-6.21360500
C	12.56999100	-15.70276400	-5.61477100
N	10.37359300	-17.20831500	-2.82296400
N	11.09084300	-18.91961000	-4.69674100
N	9.99228900	-18.21230500	-6.98598800

C	5.08387000	-18.60303900	-4.29447800
H	4.67344000	-16.53191300	-4.69473100
C	7.40213700	-19.18232400	-4.36199500
C	13.40628200	-14.63762300	-5.94069400
H	13.43763900	-12.53169000	-6.41613500
H	12.96158400	-16.69935000	-5.43383700
C	11.18406600	-18.20887000	-2.41315000
C	9.94638200	-16.29486200	-1.94303800
C	11.60456200	-19.17238500	-3.47410800
C	11.39530400	-19.71852700	-5.73645200
C	10.75671000	-19.32586800	-7.02916500
C	9.37692500	-17.78382500	-8.09445100
C	6.07827600	-19.57294400	-4.17381800
H	4.03939900	-18.86291800	-4.15795500
H	8.22184300	-19.89035500	-4.28333100
H	14.47672600	-14.78718200	-6.02049000
C	11.58328000	-18.30814300	-1.07701700
C	10.30217300	-16.32674100	-0.59677600
H	9.29912800	-15.51989000	-2.34278400
C	12.45094800	-20.24313500	-3.24920000
C	12.23728000	-20.81288700	-5.59969000
C	10.91558800	-20.04228100	-8.21905600
C	9.48878000	-18.44287900	-9.31666800
H	8.77904600	-16.88386000	-7.98512500
H	5.84032400	-20.60456000	-3.94198700
C	11.13595700	-17.35632100	-0.16213800
H	12.22996500	-19.11125200	-0.74727500
H	9.93437700	-15.56796600	0.08411100
C	12.79200100	-21.10146900	-4.32760100
H	12.86244000	-20.44011300	-2.26681100
H	12.47417500	-21.43846100	-6.44786100
C	10.27433000	-19.59334000	-9.37283000
H	11.52399800	-20.93714100	-8.25433800
H	8.97428700	-18.06306100	-10.19165300
H	11.43754900	-17.42106500	0.87806300
N	13.61779400	-22.15092000	-4.13099300
H	10.38832500	-20.13942800	-10.30335100
H	13.97971700	-22.29577200	-3.20055100
C	14.02718200	-23.08430000	-5.16829500
H	14.68812000	-23.82747500	-4.72303800
H	13.16447300	-23.60333500	-5.60155000
H	14.57033600	-22.57531900	-5.97294000
C	2.94560800	-1.50067900	3.33353000
C	3.27272100	-0.18437300	3.32559900
S	2.00402900	0.95941300	2.80636300
C	0.69235700	-0.24085200	2.79899800
S	1.28309500	-1.91743100	2.83421400
C	-0.61755400	0.08562500	2.77123900
S	-1.21361900	1.76049300	2.72203100
S	-1.92547300	-1.11865800	2.78177200
C	-2.89013800	1.35542000	3.18389000
C	-3.21257300	0.03881200	3.21625800
C	4.56164200	0.42352300	3.72046800
C	5.17695600	0.07045800	4.93427700
C	5.19232900	1.36287300	2.88882400
C	6.39860400	0.62884200	5.29124200
H	4.69300800	-0.64620100	5.58842100
C	6.41216400	1.92548600	3.25319900
H	4.74295100	1.62954100	1.93731300
C	7.02674500	1.56770200	4.45988300

H	6.88442600	0.34879400	6.21930800
H	6.89574400	2.61541700	2.56926500
C	3.83331900	-2.64931700	3.60983200
C	5.12611100	-2.73240200	3.05683200
C	3.38397900	-3.69083700	4.43562700
C	5.94733500	-3.81789200	3.35092800
H	5.48028800	-1.94228200	2.40443300
C	4.20053900	-4.77865200	4.71503200
H	2.39699400	-3.63349900	4.88316900
C	5.48923200	-4.85766600	4.17660200
H	6.94910800	-3.88146200	2.94087600
H	3.83536600	-5.54600900	5.38987900
C	-4.51171600	-0.55808900	3.59338400
C	-5.11805400	-1.52465700	2.77508000
C	-5.16146300	-0.16639100	4.77703600
C	-6.34831000	-2.07529800	3.12189800
H	-4.64179300	-1.82130600	1.84578100
C	-6.39182300	-0.71542900	5.11790400
H	-4.69642700	0.57161500	5.42108200
C	-6.99633500	-1.68038500	4.29912700
H	-6.81248600	-2.78698500	2.44686700
H	-6.90351900	-0.40669500	6.02267300
C	-3.79571100	2.50576500	3.38399200
C	-3.39073100	3.58927000	4.17895700
C	-5.06683200	2.54626000	2.77972700
C	-4.23143400	4.67819000	4.37623800
H	-2.42085700	3.56612200	4.66529900
C	-5.91142100	3.63135900	2.99159800
H	-5.38552800	1.72310500	2.15027900
C	-5.49910700	4.71414700	3.78417300
H	-3.90225200	5.48122700	5.02768500
H	-6.89666600	3.66237500	2.53987200
C	8.35613000	2.11194600	4.90122400
O	9.05061700	1.51724600	5.72708200
N	8.77108300	3.26981500	4.31074300
H	8.11608500	3.92209200	3.90778700
C	6.42020200	-5.99944900	4.47413200
O	7.64155400	-5.88980600	4.35423700
N	5.83013300	-7.15203800	4.90303600
H	4.86950900	-7.36095700	4.67877500
C	-8.33725600	-2.21193600	4.72001000
O	-9.05238800	-1.59610900	5.51207300
N	-8.73837700	-3.38279600	4.14627500
H	-8.07531000	-4.04397000	3.77205100
C	-6.45935800	5.85296600	3.98301400
O	-7.67188400	5.71308300	3.81468700
N	-5.91302500	7.03989500	4.37368600
H	-4.94769400	7.26013300	4.18298000
C	-10.05427800	-3.90443000	4.53889700
H	-10.80630500	-3.17426600	4.30658200
H	-10.06345900	-4.09989100	5.59508500
C	-6.82422100	8.17579300	4.56721500
H	-7.54032900	7.93587700	5.33029800
H	-7.34355700	8.38112500	3.64962500
C	10.07805200	3.79911200	4.72309600
H	10.83363900	3.06390500	4.52009200
H	10.06481800	4.01378700	5.77550400
C	6.69886800	-8.29736600	5.20608700
H	7.38361400	-8.03222700	5.98934500
H	7.25701100	-8.57115300	4.33009700

C	5.82445300	-9.48229700	5.65765100
H	5.14555700	-9.74213700	4.86676300
H	5.27014400	-9.19998300	6.53320600
C	6.70676400	-10.70002800	5.98707700
H	7.26848600	-10.98429500	5.11678900
H	7.38639900	-10.44877500	6.77992800
C	-10.35187100	-5.20333800	3.76630600
H	-9.60311300	-5.94015500	3.99070600
H	-10.34649100	-4.99727500	2.71223700
C	-11.73710600	-5.73422000	4.17686000
H	-11.74074700	-5.94493700	5.23004800
H	-12.48054100	-4.99057300	3.95791900
C	-6.00997500	9.41332800	4.98830100
H	-5.29883300	9.64835100	4.21826800
H	-5.49306700	9.20458000	5.90633400
C	-6.94998300	10.61493400	5.19336900
H	-7.47180000	10.82175300	4.27763800
H	-7.66292900	10.38718000	5.96374100
C	10.39284900	5.08664600	3.93801300
H	9.63944700	5.82574200	4.13805100
H	10.40941300	4.86841200	2.88651800
C	11.77054800	5.62069900	4.36986800
H	12.51569500	4.87273600	4.17252400
H	11.75522900	5.84270700	5.42062900
N	-12.04823600	-6.96089100	3.42651200
C	-13.23485900	-7.56817300	3.64887200
H	-11.36801500	-7.28714600	2.76785100
C	-14.14308500	-7.04513200	4.60623100
C	-13.62378400	-8.73609300	2.94258100
C	-15.35306800	-7.68047700	4.81472100
H	-13.87504500	-6.15468900	5.15952300
C	-14.86028400	-9.30820800	3.21048900
H	-12.96431300	-9.16313200	2.20068700
C	-16.37509500	-7.21158100	5.79736900
N	-15.70091000	-8.78792100	4.12627600
C	-15.37552000	-10.53233000	2.52067000
C	-16.19799200	-6.09831600	6.62425100
N	-17.51137600	-7.93978500	5.84739000
Zn	-17.60185700	-9.67929200	4.45219000
C	-14.65325800	-11.24717200	1.55760200
N	-16.61467700	-10.92632000	2.88840500
C	-17.21442900	-5.74082900	7.50873100
H	-15.28614900	-5.51596000	6.58672900
C	-18.48628100	-7.59479900	6.69708400
N	-18.96456800	-8.64379200	3.01867500
N	-19.52244700	-10.52801400	4.77697200
N	-17.31765900	-11.20627400	6.05248400
C	-15.23074600	-12.37633500	0.97265800
H	-13.65847400	-10.93733200	1.26333000
C	-17.16280500	-12.01000100	2.33005300
C	-18.38386300	-6.49899100	7.55083800
H	-17.09084800	-4.87940600	8.15661500
H	-19.37100300	-8.22433100	6.68238400
C	-20.25902800	-9.02898200	3.06267700
C	-18.58020600	-7.69910800	2.15200500
C	-20.58216900	-10.08691000	4.06620900
C	-19.68596400	-11.48063800	5.71388300
C	-18.43133600	-11.87632000	6.42274100
C	-16.14499900	-11.49343000	6.62998800
C	-16.51106200	-12.76960300	1.36253800

H	-14.68334100	-12.93919000	0.22386000
H	-18.16020900	-12.26467300	2.67598900
H	-19.19627100	-6.25266800	8.22455300
C	-21.20849000	-8.45512700	2.21169200
C	-19.46754100	-7.08087900	1.27440600
H	-17.52601900	-7.43913900	2.17306700
C	-21.85462500	-10.58837500	4.27265400
C	-20.92743200	-12.03723900	5.98625400
C	-18.37834300	-12.87067200	7.40396700
C	-16.01344100	-12.46915400	7.61550400
H	-15.29321200	-10.91638000	6.28238500
H	-17.00527000	-13.63711200	0.93811100
C	-20.80578500	-7.47136900	1.31002200
H	-22.24528300	-8.76486300	2.24453900
H	-19.11618600	-6.31937500	0.58788900
C	-22.05787400	-11.59252100	5.25570900
H	-22.70195900	-10.22896600	3.70164800
H	-21.03886000	-12.79669500	6.74630000
C	-17.15570600	-13.16730500	8.00490900
H	-19.26876000	-13.41151800	7.69859300
H	-15.04535000	-12.67144800	8.05872400
H	-21.53190300	-7.01702500	0.64416000
N	-23.29147200	-12.09449800	5.47493000
H	-17.09951300	-13.93678200	8.76768000
H	-24.05207100	-11.72957300	4.92169300
C	-23.60209700	-13.12075100	6.45734100
H	-24.67005300	-13.33207000	6.41027300
H	-23.05607200	-14.04863100	6.25175400
H	-23.35904600	-12.78881000	7.47334800
N	12.09843700	6.84107500	3.61604400
C	13.28350100	7.44526300	3.85560700
H	11.43052800	7.16575600	2.94414200
C	14.17492000	6.91904200	4.82682600
C	13.68825500	8.61119100	3.15437400
C	15.38535100	7.54766700	5.05157400
H	13.89474400	6.03003100	5.37639300
C	14.92353100	9.17790400	3.44045200
H	13.04174500	9.04102600	2.40274200
C	16.39129100	7.07286900	6.04787900
N	15.74868800	8.65300900	4.36781500
C	15.45466000	10.40049000	2.75933800
C	16.19665300	5.96059800	6.87215400
N	17.53102000	7.79435200	6.11315800
Zn	17.65007500	9.53349300	4.71979000
C	14.74927000	11.12197400	1.78862300
N	16.69114900	10.78677600	3.14396500
C	17.19908400	5.59709400	7.77006500
H	15.28201300	5.38357700	6.82234500
C	18.49247800	7.44362500	6.97572100
N	19.02589000	8.49058700	3.30429800
N	19.57090100	10.37124700	5.07042300
N	17.35308700	11.06174000	6.31652100
C	15.34045600	12.25009100	1.21494500
H	13.75648000	10.81888200	1.48086200
C	17.25327600	11.86792000	2.59471700
C	18.37228000	6.34837700	7.82787200
H	17.06182800	4.73635800	8.41610500
H	19.38095800	8.06800500	6.97292000
C	20.32179300	8.86846500	3.36564200
C	18.64774100	7.54832100	2.43223700

C	20.63750400	9.92429800	4.37374200
C	19.72724500	11.32277400	6.00967000
C	18.46549200	11.72540300	6.70181100
C	16.17443900	11.35543100	6.87833000
C	16.61831400	12.63457000	1.62178400
H	14.80560000	12.81863700	0.46137800
H	18.24742400	12.11601100	2.95453700
H	19.17417300	6.09725500	8.51232900
C	21.27920600	8.28957900	2.52707100
C	19.54315200	6.92541800	1.56621700
H	17.59191600	7.29422000	2.43915100
C	21.90989100	10.41856700	4.59724400
C	20.96809100	11.87231100	6.29872000
C	18.40498700	12.71986900	7.68248700
C	16.03522100	12.33171700	7.86223600
H	15.32413600	10.78327400	6.51919600
H	17.12319100	13.50130700	1.20792200
C	20.88296000	7.30842600	1.61971400
H	22.31718000	8.59352900	2.57372900
H	19.19666700	6.16613700	0.87475500
C	22.10565500	11.42136700	5.58318900
H	22.76272400	10.05452000	4.03747100
H	21.07365200	12.63099400	7.06037400
C	17.17610700	13.02331600	8.26707500
H	19.29442000	13.25562200	7.98914200
H	15.06244200	12.53942000	8.29250000
H	21.61525000	6.85030900	0.96327900
N	23.33902700	11.91635300	5.81895400
H	17.11406100	13.79296900	9.02921400
H	24.10486400	11.54725200	5.27580300
C	23.64230900	12.94066500	6.80567300
H	24.71196800	13.14597900	6.77288600
H	23.10429900	13.87164000	6.59306800
H	23.38388300	12.60991500	7.81826800
N	-6.15808900	11.79135100	5.58369700
C	-6.78982500	12.96423900	5.81065300
H	-5.16711500	11.67119700	5.66442600
C	-8.20060600	13.07384700	5.68466700
C	-6.07138300	14.12853000	6.18160200
C	-8.81128500	14.29296000	5.92599500
H	-8.77773800	12.20209300	5.40537400
C	-6.76242400	15.31032700	6.40045700
H	-4.99722300	14.08708000	6.29179400
C	-10.28467000	14.51754800	5.82729500
N	-8.10033600	15.38577500	6.27642700
C	-6.10090800	16.59255000	6.78956000
C	-11.19480800	13.52255600	5.45781500
N	-10.70454300	15.76803800	6.12174400
Zn	-9.10059300	17.21595400	6.68266700
C	-4.71803500	16.73586300	6.93628200
N	-6.93263000	17.63763000	6.99377000
C	-12.55259100	13.83263100	5.39699900
H	-10.85896900	12.52140700	5.21921600
C	-12.00874000	16.06335800	6.06263600
N	-9.57826900	17.12746100	8.86138000
N	-10.13280800	19.02251500	7.11336500
N	-9.18737200	18.33031000	4.75251300
C	-4.19885100	17.97750900	7.30055200
H	-4.04979400	15.90068800	6.76856000
C	-6.43204900	18.82883200	7.34241300

C	-12.97464900	15.12568300	5.70451500
H	-13.27024400	13.07036800	5.11226200
H	-12.27355100	17.08632700	6.31302200
C	-10.25390000	18.18893200	9.35396000
C	-9.24201800	16.11772800	9.67314900
C	-10.58153200	19.25879700	8.36469400
C	-10.36661800	19.91336400	6.13190100
C	-9.81427400	19.52667100	4.79835300
C	-8.65819700	17.89942300	3.60146900
C	-5.06661300	19.04857800	7.50941200
H	-3.12766200	18.10354000	7.41778600
H	-7.15887600	19.62231300	7.48891300
H	-14.02131900	15.40473100	5.66922800
C	-10.60768500	18.25138600	10.70507000
C	-9.56053200	16.10752300	11.02905700
H	-8.70127300	15.29817500	9.20926600
C	-11.28777900	20.40622300	8.67800400
C	-11.06777400	21.08898500	6.35843200
C	-9.91863300	20.32675500	3.65658200
C	-8.72451500	18.63853600	2.42263900
H	-8.16941100	16.93041900	3.63974500
H	-4.70128400	20.02877200	7.79283500
C	-10.25632600	17.19858600	11.54844200
H	-11.14633700	19.10288700	11.10106700
H	-9.27003100	15.27022700	11.65286300
C	-11.55098900	21.36296600	7.66267600
H	-11.64769900	20.58988800	9.68292800
H	-11.25014500	21.78849600	5.55572300
C	-9.36793400	19.87495800	2.45820400
H	-10.41633800	21.28754200	3.69249600
H	-8.28326700	18.25359800	1.51063700
H	-10.52423600	17.23356900	12.59919800
N	-12.23995500	22.48848100	7.94631200
H	-9.44122700	20.48550000	1.56436700
H	-12.55785200	22.61778900	8.89498000
C	-12.56131700	23.52535100	6.97867400
H	-13.11688400	24.31259000	7.48760300
H	-11.65415600	23.96463800	6.54800000
H	-13.18207600	23.13425800	6.16426100
N	5.84656700	-11.81730800	6.40648900
C	6.41102500	-12.99731700	6.74622700
H	4.85884500	-11.65261500	6.42259500
C	7.81995100	-13.17658400	6.71239300
C	5.61838300	-14.10084500	7.15016700
C	8.35712200	-14.40174900	7.07130500
H	8.45286900	-12.35219100	6.41098900
C	6.23847800	-15.29335800	7.48876600
H	4.54290000	-14.00500200	7.19300900
C	9.82132600	-14.69701200	7.07744200
N	7.57599900	-15.43550700	7.45098900
C	5.49514900	-16.51496200	7.92257800
C	10.79770500	-13.77102000	6.69792000
N	10.16336000	-15.94107800	7.48036700
Zn	8.46261500	-17.27476800	8.03952300
C	4.10087400	-16.58692700	7.99483000
N	6.26278900	-17.57850100	8.24664500
C	12.14034500	-14.14364900	6.74072800
H	10.52438900	-12.77526600	6.37261800
C	11.45320100	-16.29649500	7.52049000
N	8.81841800	-17.05418100	10.23219200

N	9.38085900	-19.08998400	8.65319700
N	8.60633500	-18.52507800	6.19807100
C	3.50286100	-17.77605700	8.40989000
H	3.48407500	-15.73708200	7.73117100
C	5.68670300	-18.71927300	8.64393000
C	12.48174400	-15.42921800	7.15947600
H	12.90879400	-13.43524900	6.44953600
H	11.65409000	-17.31006500	7.85463400
C	9.41314900	-18.10687900	10.83544400
C	8.48490100	-15.97606900	10.95193800
C	9.74531900	-19.25702100	9.94249200
C	9.62763300	-20.05709100	7.75028500
C	9.17161300	-19.74155000	6.36274800
C	8.16516100	-18.15380200	4.99056800
C	4.30500200	-18.86681500	8.74252500
H	2.42191200	-17.84652200	8.47041400
H	6.36519900	-19.53154800	8.88726000
H	13.51417300	-15.75552300	7.20661000
C	9.68556200	-18.08950100	12.20660100
C	8.72533100	-15.88438900	12.32081800
H	8.01145400	-15.16832000	10.40179600
C	10.37674100	-20.40933600	10.37492200
C	10.25761800	-21.24340300	8.09787800
C	9.30269300	-20.62411900	5.28643800
C	8.26334500	-18.97666300	3.87091500
H	7.72184500	-17.16404900	4.93326000
H	3.87757400	-19.80779100	9.06878900
C	9.33723900	-16.96558100	12.95398500
H	10.15928700	-18.93377700	12.69116000
H	8.43996300	-14.99334600	12.86780800
C	10.65163700	-21.44576900	9.44448000
H	10.66928500	-20.53736600	11.40989900
H	10.45198700	-22.00499300	7.35685500
C	8.84369500	-20.23449000	4.02913800
H	9.75100200	-21.60082800	5.41750600
H	7.89384400	-18.63829300	2.90994400
H	9.54252900	-16.93819800	14.01897000
N	11.26857300	-22.57761400	9.84480800
H	8.93858900	-20.90937800	3.18497600
H	11.52506500	-22.65361600	10.81752600
C	11.59480800	-23.69308200	8.97075600
H	12.08201800	-24.46612400	9.56442500
H	10.69380800	-24.12144300	8.51677200
H	12.27915200	-23.38781700	8.17072900

Supplementary Table 19. DFT-optimized geometry of Pt-bound model **1** (singlet), computed at the B3LYP-D3/ LANL2DZ (Zn, Pt)/ 6-31G* (HCNOS) level in acetonitrile solvent using PCM.

Atom	x	y	z
C	2.35028400	1.27190900	-2.48971100
C	2.50702000	2.44312300	-1.81909100
C	-4.01705500	1.62854700	-1.52663300
S	-2.25917100	3.58717200	-1.02779900
S	-2.57949000	0.91682100	-2.28590200
S	0.66769300	0.75006300	-2.77489100
S	1.03732500	3.31652000	-1.33359100
C	-1.44968100	2.17672000	-1.74213900
C	-0.10986200	2.08502000	-1.90101200
C	-3.87536400	2.84364800	-0.94166600
C	-5.26236400	0.84360400	-1.66279500
C	-5.28518900	-0.51528300	-1.31244400
C	-6.43752200	1.43820800	-2.15493300
C	-6.45299900	-1.26193600	-1.44072400
H	-4.38885700	-0.98367200	-0.92283400
C	-7.60377100	0.69249500	-2.27460300
H	-6.42711600	2.48511000	-2.43894300
C	-7.62343500	-0.66730000	-1.93055300
H	-6.45082800	-2.29920800	-1.12220000
H	-8.51471500	1.14937900	-2.64591400
C	-8.92153400	-1.40889800	-2.08509700
O	-9.99968200	-0.81337800	-2.11858400
N	-8.84235300	-2.76987500	-2.15599100
H	-9.70074200	-3.26134700	-2.37041600
C	-4.90849500	3.60364000	-0.20705600
C	-5.00999400	4.99822700	-0.35048900
C	-5.79940900	2.95231400	0.66714000
C	-5.96894000	5.71939300	0.35714800
H	-4.35311300	5.52259400	-1.03813500
C	-6.76077100	3.67323000	1.36329800
H	-5.74035300	1.87726000	0.79216300
C	-6.85143200	5.06593400	1.22815700
H	-6.04065200	6.79010400	0.19404800
H	-7.45082300	3.16795900	2.03030900
C	3.77197500	3.12529900	-1.46565800
C	3.99106900	3.56315900	-0.14803700
C	4.76751000	3.35546300	-2.43065200
C	5.20018800	4.15501500	0.21176800
H	3.21231900	3.43584000	0.59696300
C	5.97063300	3.95034000	-2.06992300
H	4.59630100	3.05001500	-3.45728500
C	6.21204700	4.33109900	-0.74204700
H	5.33619800	4.49718000	1.23306700
H	6.74284500	4.12590300	-2.81110300
C	3.39495800	0.36374300	-3.01243800
C	3.15621200	-0.40551300	-4.16673700
C	4.64331100	0.22200600	-2.37588100
C	4.11931400	-1.28402800	-4.65809300
H	2.21937000	-0.30564400	-4.70592600
C	5.61228000	-0.63250100	-2.88178700
H	4.85051700	0.77782200	-1.47340500
C	5.35818000	-1.41335400	-4.01870000
H	3.90093400	-1.84068200	-5.56394800
H	6.57192800	-0.72744200	-2.38503100
C	6.44207000	-2.34559600	-4.47998400
O	7.61832300	-2.16976100	-4.15603600
C	7.54692800	4.94335900	-0.41999300

O	8.23331300	5.47964000	-1.29102200
C	-7.92008800	5.77345100	2.01304100
O	-8.89194400	5.16400100	2.46478400
N	7.93874200	4.88774400	0.88510100
H	7.53405000	4.22225200	1.52877500
H	8.87894500	5.19958200	1.09220100
N	-7.77396500	7.11998500	2.17411000
H	-8.43503500	7.57789400	2.78825900
H	-6.87785000	7.57253300	2.06225200
N	6.05740500	-3.38193400	-5.27650300
H	6.76217300	-4.06455900	-5.52244700
H	5.09311900	-3.66920500	-5.35952900
H	-7.98539500	-3.23182100	-2.42638200
C	3.59978700	0.26416900	1.38311600
C	2.33828600	0.86235000	1.54615400
C	1.23198200	0.24787200	0.97179600
C	2.57147900	-1.38987900	-0.01352900
C	3.73000300	-0.84500300	0.53408800
H	2.22555300	1.70846000	2.21214600
H	4.68666500	-1.32548500	0.39126900
C	2.55483100	-2.64077800	-0.81718800
C	3.68272000	-3.16072700	-1.45378000
C	3.55865800	-4.33303000	-2.19889800
H	4.63269300	-2.64629900	-1.39896800
C	1.23945300	-4.38473000	-1.60866700
C	2.31687900	-4.96115000	-2.28045200
H	4.42178200	-4.74046800	-2.71549800
H	0.24906500	-4.82785900	-1.63734600
H	2.17845100	-5.87344500	-2.84952500
C	-0.16634700	0.67329400	1.23724500
C	-0.49535200	1.92091200	1.77266600
C	-2.38885900	0.01285500	1.27127900
C	-1.82825000	2.19814900	2.06841400
H	0.26868000	2.67096500	1.93649600
C	-2.79538600	1.22257400	1.82871300
H	-3.10169600	-0.77783000	1.06415100
H	-2.10678200	3.16349700	2.47725000
H	-3.83898600	1.39471200	2.06014300
N	-1.11192500	-0.25702100	0.97731300
N	1.35931200	-0.86168000	0.22148400
N	1.35367500	-3.25551800	-0.89875800
N	4.72872000	0.81032900	2.09536400
H	4.52810000	1.78362600	2.33995400
C	6.05699800	0.78643200	1.40669400
H	6.77200000	1.27393800	2.06830700
H	6.37267200	-0.24143400	1.24240700
H	5.99416900	1.33067500	0.45972200
C	-4.36329500	-4.33271000	2.10334300
C	-4.24338500	-4.09918300	0.70905100
C	-3.06527000	-3.57549300	0.20661900
C	-2.10152900	-3.49511500	2.32999000
C	-3.24535900	-4.02276600	2.91553600
H	-5.07944600	-4.33308100	0.06080400
H	-3.28732900	-4.20559400	3.97939000
C	-0.87861200	-3.14290500	3.11335000
C	-0.82809700	-3.21498200	4.51007300
C	0.35205100	-2.87662600	5.17094900
H	-1.69413600	-3.52053500	5.08371700
C	1.32655100	-2.42668600	3.03605800
C	1.45774100	-2.47951100	4.42205100

H	0.40074100	-2.92420400	6.25414100
H	2.16642200	-2.11431500	2.42607300
H	2.40127500	-2.20476200	4.88237000
C	-2.84801400	-3.28928400	-1.24159500
C	-3.76566100	-3.62381000	-2.24272900
C	-1.39876000	-2.38480600	-2.82183900
C	-3.47354800	-3.30691900	-3.56834500
H	-4.69220000	-4.12998400	-2.00224300
C	-2.26659600	-2.67749700	-3.87056100
H	-0.44283400	-1.90297100	-2.99296300
H	-4.18039200	-3.55547500	-4.35320200
H	-1.99876600	-2.41910000	-4.88864400
N	-1.68462800	-2.67338800	-1.54571400
N	-2.01977000	-3.27086700	1.00515300
N	0.19336200	-2.74052300	2.39365900
N	-5.50616700	-4.83379500	2.61796300
H	-6.25890000	-5.04053900	1.97687900
C	-5.72860100	-5.09990500	4.03039900
H	-6.74501400	-5.47486600	4.15355700
H	-5.02957000	-5.85499000	4.40996200
H	-5.61909300	-4.18705900	4.62814500
Zn	-0.33695600	-2.22815600	0.16686700
Pt	4.55933700	-0.27351400	3.79833900

Sum of electronic and thermal Free Energies = -5280.921754 (Hartree/Particle).

Supplementary Table 20. DFT-optimized geometry of Pt-bound model **2** (singlet), computed at the B3LYP-D3/ LANL2DZ (Zn, Pt)/ 6-31G* (HCNOS) level in acetonitrile solvent using PCM.

Atom	x	y	z
C	-3.65942600	1.64745900	1.23749400
C	-3.88017500	2.79045900	0.54065300
C	2.68226800	2.70708000	1.24278400
S	0.86696700	4.31362100	0.08730700
S	1.20365800	1.81496400	1.65763200
S	-1.96485400	1.24324300	1.59208300
S	-2.45416400	3.72012800	0.02681300
C	0.07603600	2.90643600	0.82819000
C	-1.25429500	2.66897400	0.80584200
C	2.53158400	3.85283800	0.53310600
C	3.92805600	2.11014000	1.76846400
C	4.12262600	0.72015400	1.69419700
C	4.92077100	2.89690500	2.38032800
C	5.27671700	0.13243500	2.20134800
H	3.37113300	0.09701600	1.22446600
C	6.07654900	2.30776500	2.87927500
H	4.77806000	3.96852100	2.46528400
C	6.26405200	0.91927600	2.80751600
H	5.41221400	-0.93811400	2.08995900
H	6.84505900	2.91428100	3.34650900
C	7.53068000	0.34647500	3.37501600
O	8.54043700	1.03702400	3.52130100

N	7.50749100	-0.98285700	3.69008200
H	8.31799000	-1.34398000	4.17706500
C	3.59637000	4.74276100	0.02402900
C	3.45471900	6.13967800	0.09136900
C	4.76233300	4.21262900	-0.55867500
C	4.44587800	6.98020100	-0.41060400
H	2.57636700	6.57341400	0.55973300
C	5.75292500	5.05313500	-1.04892500
H	4.88576500	3.13888100	-0.62137900
C	5.60296800	6.44637900	-0.99409300
H	4.31984200	8.05368600	-0.31053100
H	6.65353700	4.64186400	-1.49195800
C	-5.19319200	3.38554100	0.21268400
C	-5.47624000	3.84319400	-1.08428400
C	-6.17343300	3.52450800	1.21169800
C	-6.72186100	4.38964700	-1.38583300
H	-4.72616000	3.74905400	-1.86221900
C	-7.41166400	4.07648600	0.90904000
H	-5.95468400	3.19709000	2.22259000
C	-7.70691200	4.50068900	-0.39473200
H	-6.90626300	4.74805400	-2.39378900
H	-8.16728900	4.19179800	1.67859800
C	-4.64791200	0.63790900	1.66825000
C	-4.46072400	-0.08341900	2.86063800
C	-5.75487000	0.31316200	0.86056100
C	-5.32616400	-1.11471600	3.21642600
H	-3.63553400	0.16358400	3.52138100
C	-6.62428800	-0.70666600	1.22386100
H	-5.92092200	0.85380300	-0.06236300
C	-6.40756600	-1.45227400	2.39255900
H	-5.15548600	-1.63542000	4.15343500
H	-7.46917300	-0.95966900	0.59275800
C	-7.35991900	-2.57702800	2.68583500
O	-8.47614900	-2.62436400	2.16522800
C	-9.06811300	5.08741300	-0.64474200
O	-9.75039600	5.54062300	0.27617600
C	6.71745900	7.29016700	-1.54576900
O	7.85318600	6.83425000	-1.69438000
N	-9.49251000	5.11391400	-1.94110800
H	-9.08750700	4.50632200	-2.63926300
H	-10.44882300	5.40466900	-2.09923300
N	6.41362700	8.58566300	-1.84553500
H	7.13105400	9.12443300	-2.31337200
H	5.45981200	8.88410600	-1.99254900
N	-6.92670400	-3.53516000	3.55168100
H	-7.52587600	-4.33568700	3.70304500
H	-5.96335400	-3.61781700	3.84086000
H	6.63464000	-1.46210100	3.86359600
C	-2.86012700	0.81465600	-2.39157000
C	-1.51438000	1.25643300	-2.45967800
C	-0.52392900	0.51988500	-1.83330600
C	-2.08452000	-1.01346200	-1.01059300
C	-3.13346800	-0.35309300	-1.64509800
H	-1.28019600	2.14779400	-3.02825700
H	-4.14154000	-0.73751300	-1.57387300
C	-2.27878600	-2.20332500	-0.14857400
C	-3.48211600	-2.54045700	0.42526700
C	-3.57810900	-3.65966400	1.29658900
H	-4.35763200	-1.92750000	0.25430900
C	-1.17805700	-3.99712200	1.04637000

C	-2.45403200	-4.37476500	1.61178500
H	-4.54065300	-3.91439600	1.72783600
H	-0.27979500	-4.16904300	1.64136500
H	-2.48096100	-5.20744300	2.30721500
C	0.92597000	0.85469000	-1.92454900
C	1.40836100	2.01155300	-2.54692000
C	3.09532900	0.12290800	-1.52229600
C	2.78382600	2.20113700	-2.66029000
H	0.72756600	2.75904800	-2.93303600
C	3.64964800	1.23023300	-2.15810100
H	3.72167700	-0.65486300	-1.09804300
H	3.17241200	3.09731700	-3.13254300
H	4.72590200	1.32676100	-2.24500700
N	1.77483900	-0.05440800	-1.39636300
N	-0.80880400	-0.59604700	-1.12967000
N	-1.10727500	-2.90216400	0.15724800
N	-3.83305800	1.50649500	-3.03579500
H	-3.53521000	2.25742600	-3.64248100
C	-5.20637500	1.04944400	-3.17262500
H	-5.74998700	1.76910900	-3.78548000
H	-5.26124000	0.06287900	-3.65019700
H	-5.70128200	0.99543900	-2.19802600
C	5.15414900	-4.11669300	-0.46742000
C	4.59685000	-3.58874900	0.72675700
C	3.31362300	-3.07039200	0.70591900
C	3.06848800	-3.55380300	-1.56217900
C	4.34572500	-4.09481200	-1.63046600
H	5.18831100	-3.59735400	1.63460600
H	4.73035400	-4.48401200	-2.56231700
C	2.14681700	-3.49560300	-2.73487900
C	2.40874500	-4.14080600	-3.94861800
C	1.48102700	-4.04132200	-4.98464900
H	3.31203000	-4.72188600	-4.08840500
C	0.12528100	-2.69138400	-3.54915300
C	0.31495300	-3.30165200	-4.78749800
H	1.66871700	-4.53800500	-5.93122000
H	-0.76412700	-2.10796200	-3.33435400
H	-0.43107800	-3.19827900	-5.56760400
C	2.63422600	-2.50984800	1.90976100
C	3.14138900	-2.62260500	3.20801400
C	0.75198500	-1.39201900	2.69846900
C	2.41699000	-2.08163000	4.26948500
H	4.07520800	-3.13763900	3.39826200
C	1.19498500	-1.45872300	4.01707600
H	-0.19405600	-0.92977700	2.43854900
H	2.80025200	-2.15815100	5.28181600
H	0.59595000	-1.03685600	4.81594400
N	1.45757800	-1.88889000	1.67516100
N	2.57123500	-3.03899700	-0.42153400
N	1.01320700	-2.78726300	-2.55138500
N	6.40754000	-4.61828200	-0.47705200
H	6.93019400	-4.59682700	0.38710900
C	7.06446600	-5.17118800	-1.65100100
H	8.06352200	-5.50007900	-1.36362600
H	6.51386900	-6.03354500	-2.04583300
H	7.15910400	-4.42086400	-2.44534200
Zn	0.77633600	-1.85004300	-0.45295500
Pt	-0.84678600	-4.70317400	-0.86513600

Sum of electronic and thermal Free Energies = -5280.943999 (Hartree/Particle).

Supplementary Table 21. DFT-optimized geometry of Pt-bound model **3** (singlet), computed at the B3LYP-D3/ LANL2DZ (Zn, Pt)/ 6-31G* (HCNOS) level in acetonitrile solvent using PCM.

Atom	x	y	z
C	-4.03451800	1.19630500	1.14749300
C	-4.33658000	2.27830300	0.38698400
C	2.15902100	2.88542700	1.36735800
S	0.25451200	4.26788000	0.07367100
S	0.76352900	1.85343600	1.72864300
S	-2.31551100	0.95009900	1.53253700
S	-2.98421400	3.30184000	-0.15223800
C	-0.42846600	2.80655500	0.82112800
C	-1.72070900	2.42289000	0.73436900
C	1.93283700	3.98845400	0.61233700
C	3.43494600	2.44019800	1.96494800
C	3.79907300	1.08336700	1.92326600
C	4.29355100	3.35085600	2.60681100
C	4.99222300	0.64910400	2.49377400
H	3.15315900	0.36695000	1.42810300
C	5.48874200	2.91596900	3.16576400
H	4.01615900	4.39772200	2.66423300
C	5.84788600	1.56048100	3.12653800
H	5.26160800	-0.39862000	2.41081300
H	6.15576400	3.61846500	3.65382500
C	7.15203400	1.16231100	3.75617600
O	8.05963100	1.97825700	3.92588000
N	7.28451100	-0.15247200	4.10143600
H	8.11276000	-0.39837500	4.62862700
C	2.93682100	4.94871100	0.10807300
C	2.67168700	6.32892400	0.10308700
C	4.16771700	4.49774600	-0.40563600
C	3.60518400	7.23063200	-0.40334700
H	1.74013300	6.70305800	0.51710900
C	5.10027500	5.40054100	-0.89933700
H	4.38658100	3.43642400	-0.41340400
C	4.82670900	6.77587400	-0.91860500
H	3.38196900	8.29206600	-0.36147200
H	6.05040800	5.05188400	-1.28942300
C	-5.68539400	2.73811500	-0.00712100
C	-5.96602700	3.08534800	-1.33846500
C	-6.70326900	2.85750100	0.95574600
C	-7.24251700	3.50483000	-1.70658700
H	-5.18742900	3.00510000	-2.08981300
C	-7.97337100	3.28178600	0.58595300
H	-6.48904700	2.61489700	1.99124900
C	-8.26305600	3.59547800	-0.74999500
H	-7.42461200	3.78089300	-2.74050600
H	-8.75937200	3.38064500	1.32681600
C	-4.94479200	0.13871500	1.63380500
C	-4.71625100	-0.48535000	2.87342500
C	-6.02342900	-0.31599500	0.85024200
C	-5.51953300	-1.53855600	3.30398100

H	-3.91244400	-0.13896800	3.51561300
C	-6.83217500	-1.35597300	1.28823400
H	-6.22033600	0.14545900	-0.10891100
C	-6.57802600	-1.99737000	2.51000600
H	-5.32207000	-1.97774100	4.27679000
H	-7.65899800	-1.70349100	0.67835000
C	-7.46867500	-3.14643000	2.89001900
O	-8.58250800	-3.28944200	2.38173200
C	-9.65990600	4.04826700	-1.07201700
O	-10.40870700	4.48926000	-0.19792300
C	5.88214300	7.68900300	-1.47591300
O	7.05763100	7.32905700	-1.56819400
N	-10.04172000	3.96949800	-2.37927100
H	-9.56222700	3.36708800	-3.03321000
H	-11.01280800	4.16963700	-2.58146700
N	5.47734800	8.93757200	-1.84743600
H	6.16081300	9.51491700	-2.32012600
H	4.50695500	9.14283800	-2.03899500
N	-6.98085900	-4.01848200	3.81494800
H	-7.54341000	-4.82647400	4.04551200
H	-6.02069800	-4.01588400	4.12531400
H	6.47298400	-0.73565200	4.25050000
C	-3.08889000	0.06072500	-2.28629200
C	-1.74689000	0.50254300	-2.33895800
C	-0.77174400	-0.18270500	-1.62885500
C	-2.35277200	-1.65774400	-0.75389500
C	-3.38524800	-1.04511100	-1.45080800
H	-1.48453100	1.34866400	-2.96330900
H	-4.40210900	-1.39835100	-1.35787800
C	-2.56195700	-2.78162000	0.20491100
C	-3.82524000	-3.24827100	0.57796100
C	-3.92137100	-4.27954800	1.51052200
H	-4.72476000	-2.80192900	0.17529200
C	-1.53360900	-4.30367200	1.63234700
C	-2.75716900	-4.82268900	2.05226900
H	-4.89689000	-4.64337700	1.81715100
H	-0.59315800	-4.68258800	2.02140100
H	-2.78963800	-5.62257200	2.78345700
C	0.65701900	0.20140400	-1.63608300
C	1.09939900	1.46545900	-1.94496400
C	2.91828700	-0.52467700	-1.21960500
C	2.48657600	1.76865200	-1.91888200
H	0.38639300	2.24830400	-2.17160500
C	3.38626800	0.79952600	-1.56296500
H	3.51003200	-1.11120700	-0.51617500
H	2.81519400	2.77380000	-2.15863400
H	4.45137200	0.99816400	-1.50367800
N	1.53324900	-0.80552400	-1.21668800
N	-1.07267600	-1.26055800	-0.87582100
N	-1.43811100	-3.31361000	0.73644500
N	-4.04528800	0.68811100	-3.01525400
H	-3.73290300	1.40562500	-3.65419100
C	-5.40089500	0.19087100	-3.19009700
H	-5.90824600	0.82481300	-3.91802500
H	-5.40828100	-0.84357400	-3.55563100
H	-5.96537400	0.23818300	-2.25367100
C	4.89594700	-4.59246700	0.26117800
C	4.40961300	-3.75836000	1.30193800
C	3.10124500	-3.30952400	1.25396600
C	2.70638300	-4.42546800	-0.75413500

C	3.99773900	-4.93480000	-0.77996600
H	5.07066800	-3.48555700	2.11598300
H	4.32519800	-5.57236900	-1.58883400
C	1.68802300	-4.71061500	-1.80927400
C	1.89469200	-5.61339100	-2.85624200
C	0.89069000	-5.79131900	-3.80817000
H	2.81781700	-6.17437300	-2.93371000
C	-0.43263200	-4.19889900	-2.61175800
C	-0.29711700	-5.07062100	-3.69134800
H	1.03790900	-6.48709900	-4.62803300
H	-1.33362200	-3.61015700	-2.47112900
H	-1.10086500	-5.17892300	-4.41095900
C	2.47499800	-2.46374100	2.31096000
C	3.07863700	-2.19350900	3.54234500
C	0.57470700	-1.27810500	2.93581500
C	2.39118400	-1.42713800	4.48317100
H	4.06027900	-2.58663900	3.77646000
C	1.11039400	-0.96558100	4.18373400
H	-0.41626300	-0.94747100	2.64484000
H	2.85028900	-1.20473600	5.44089300
H	0.53767200	-0.37623200	4.89052600
N	1.24116600	-1.99249900	2.02161600
N	2.27580300	-3.62401900	0.23559200
N	0.52718400	-4.02600000	-1.69737400
N	6.17119400	-5.03698900	0.28031600
H	6.75517900	-4.75592300	1.05503900
C	6.76429900	-5.88436700	-0.74168500
H	7.80380800	-6.07302900	-0.47173300
H	6.24291700	-6.84653400	-0.81462000
H	6.74229100	-5.39771800	-1.72449800
Zn	0.48728400	-2.46774800	-0.04463800
Pt	2.45580600	-1.71365900	-2.84849000

Sum of electronic and thermal Free Energies = -5280.942616 (Hartree/Particle).

Supplementary Table 22. DFT-optimized geometry of Pt-bound model **4** (singlet), computed at the B3LYP-D3/ LANL2DZ (Zn, Pt)/ 6-31G* (HCNOS) level in acetonitrile solvent using PCM.

Atom	x	y	z
C	-3.68235500	1.01982300	1.69958600
C	-3.88736600	2.10614500	0.91259400
C	2.65280100	2.17407400	1.70205900
S	0.80783000	3.61012100	0.38555800
S	1.21586300	1.19616400	2.06838500
S	-1.99066700	0.59267600	2.05284800
S	-2.44816300	2.93623800	0.28065100
C	0.06133700	2.20908400	1.18145800
C	-1.26426700	1.94519400	1.15792800
C	2.47079400	3.27611800	0.93249400
C	3.90795600	1.68716800	2.31152900
C	4.21888300	0.31681500	2.28699600
C	4.79824200	2.56978000	2.94933600

C	5.38873900	-0.15918500	2.87136300
H	3.54809700	-0.37829800	1.79502300
C	5.97142200	2.09328300	3.52136700
H	4.56287100	3.62752500	2.99333200
C	6.27574800	0.72409500	3.50046800
H	5.61568600	-1.21805400	2.80393700
H	6.66309100	2.77384000	4.00620400
C	7.55773000	0.27895500	4.14414300
O	8.49526500	1.05965800	4.31677300
N	7.63465100	-1.03781000	4.49762800
H	8.44842000	-1.31505100	5.03170200
C	3.50332000	4.19951900	0.41713200
C	3.26245200	5.58346600	0.35917200
C	4.73384900	3.71240000	-0.06189300
C	4.21640600	6.45108800	-0.16823100
H	2.33307700	5.98896900	0.74754100
C	5.68679000	4.58067500	-0.57709200
H	4.93560400	2.64953100	-0.02735900
C	5.43591400	5.95850600	-0.65207300
H	4.00948600	7.51670500	-0.16751000
H	6.63556500	4.20214500	-0.94186100
C	-5.18657200	2.71661100	0.56097300
C	-5.46709600	3.10808400	-0.75847000
C	-6.15197200	2.94790900	1.55753600
C	-6.69606600	3.67958200	-1.08176200
H	-4.72843600	2.94630600	-1.53740100
C	-7.37289400	3.52481100	1.23274600
H	-5.93462200	2.67445300	2.58455700
C	-7.66654600	3.88316200	-0.09113600
H	-6.87726300	3.98424600	-2.10783100
H	-8.11591100	3.71195400	2.00047300
C	-4.69368100	0.07856400	2.22120100
C	-4.52142100	-0.53936600	3.47225200
C	-5.81250100	-0.28450100	1.44578100
C	-5.41986700	-1.50343400	3.92326200
H	-3.68497200	-0.25966100	4.10528800
C	-6.71420200	-1.23553700	1.90381500
H	-5.96341100	0.17035900	0.47448500
C	-6.51965300	-1.87367400	3.13815300
H	-5.26367900	-1.94189600	4.90391500
H	-7.57193500	-1.51506500	1.30186900
C	-7.52421400	-2.91350000	3.54931000
O	-8.64630400	-2.95498400	3.04073800
C	-9.00857300	4.50356500	-0.36273100
O	-9.66683300	5.02758600	0.53787600
C	6.51230700	6.83269300	-1.23120400
O	7.68232000	6.44990200	-1.29763700
N	-9.44289100	4.47819600	-1.65569200
H	-9.06474900	3.82269400	-2.32486400
H	-10.38984100	4.79389300	-1.82205800
N	6.13284900	8.07318300	-1.65293900
H	6.83094600	8.62128500	-2.13885400
H	5.16810500	8.28790600	-1.86165500
N	-7.13659600	-3.79047300	4.51738400
H	-7.77133200	-4.54745500	4.73503000
H	-6.16769600	-3.92187500	4.76899100
H	6.79988000	-1.58987900	4.63775400
C	-2.89959600	0.14663000	-2.25943200
C	-1.51856900	0.50705800	-2.50982200
C	-0.50925200	-0.13788300	-1.72006600

C	-2.11308100	-1.44201700	-0.61293700
C	-3.17110200	-0.87188700	-1.32384600
H	-1.32629900	1.46088900	-2.99381500
H	-4.18569800	-1.20485100	-1.16149300
C	-2.31005600	-2.55710400	0.35857600
C	-3.55821300	-2.93490100	0.85989000
C	-3.63595100	-3.98837300	1.76986800
H	-4.45610200	-2.40493600	0.57283600
C	-1.26059000	-4.21421800	1.61002700
C	-2.46854800	-4.64566900	2.15520100
H	-4.59836800	-4.28325200	2.17446900
H	-0.31827300	-4.68298100	1.87619900
H	-2.48559600	-5.46925000	2.86007400
C	0.91663800	0.31362300	-1.67971800
C	1.37498900	1.47481700	-2.31388300
C	3.07400500	-0.25768700	-1.05753700
C	2.73970400	1.74385400	-2.32294200
H	0.68085100	2.15287800	-2.79356500
C	3.61435200	0.84942500	-1.70227600
H	3.71057500	-0.96985000	-0.54420700
H	3.11603000	2.64032300	-2.80471200
H	4.68664900	1.00633900	-1.70889800
N	1.75937800	-0.51756100	-1.03586600
N	-0.84080800	-1.06726900	-0.76804500
N	-1.18332500	-3.20113000	0.73810600
N	-3.87233800	0.79106300	-2.92988300
H	-3.58409800	1.44059800	-3.64925900
C	-5.27828500	0.41188600	-2.90131400
H	-5.82810300	1.08032400	-3.56413700
H	-5.41917900	-0.62211500	-3.23801600
H	-5.68933100	0.51891000	-1.89320500
C	5.19749600	-4.20668300	-0.79046900
C	4.72647600	-3.89625400	0.51220800
C	3.41818000	-3.47418600	0.68076700
C	3.00448400	-3.59904600	-1.61142900
C	4.29351900	-4.04215900	-1.87034900
H	5.40499500	-3.98167200	1.35254800
H	4.60051300	-4.27168000	-2.88142000
C	1.99314600	-3.36527400	-2.68781200
C	2.37948700	-3.13646900	-4.03427600
C	1.40073100	-2.95540300	-4.98846500
H	3.42694900	-3.09682500	-4.30643500
C	-0.27801600	-3.17755500	-3.22192200
C	0.02196400	-3.01595900	-4.62484600
H	1.67133200	-2.80496000	-6.02932400
H	-1.25328900	-3.53085400	-2.89351300
H	-0.72746500	-3.23661500	-5.38252900
C	2.84148800	-3.07793600	2.00004400
C	3.49793400	-3.27195700	3.21957200
C	1.03587200	-2.08631400	3.08557900
C	2.88990800	-2.83546900	4.39595400
H	4.46265800	-3.76282600	3.25897200
C	1.63530300	-2.22935800	4.33429700
H	0.05613400	-1.63354900	2.97338700
H	3.39115800	-2.97358100	5.34840800
H	1.12879800	-1.87844400	5.22605800
N	1.62373900	-2.49099700	1.95230400
N	2.57481100	-3.33958800	-0.36279700
N	0.72731600	-3.35809600	-2.28630600
N	6.46570100	-4.63002400	-0.97710100

H	7.05284200	-4.72811900	-0.16094300
C	7.04471700	-4.95357800	-2.27145300
H	8.08556500	-5.24153500	-2.12083700
H	6.51702200	-5.78890200	-2.74761900
H	7.01605800	-4.08959900	-2.94629200
Zn	0.70610400	-2.34723300	-0.08065600
Pt	-0.62429100	-1.10325500	-3.73940800

Sum of electronic and thermal Free Energies = -5280.964475 (Hartree/Particle).

Supplementary Table 23. DFT-optimized geometry of Pt-bound model **5** (singlet), computed at the B3LYP-D3/ LANL2DZ (Zn, Pt)/ 6-31G* (HCNOS) level in acetonitrile solvent using PCM.

Atom	x	y	z
C	3.75160000	0.68595800	-0.71704200
C	3.98894400	1.89206800	-0.14650300
C	-2.54033700	2.15093800	-0.98451000
S	-0.63598800	3.65154000	0.14575400
S	-1.12318700	1.14086800	-1.34631600
S	2.02246300	0.32586200	-1.15733100
S	2.61964900	2.98231500	0.12208400
C	0.06194600	2.17184500	-0.53350300
C	1.38472700	1.88307900	-0.49504600
C	-2.32030700	3.31468000	-0.32341000
C	-3.81503200	1.63394200	-1.52571600
C	-4.13016200	0.27212100	-1.38770100
C	-4.71867200	2.46838600	-2.20807200
C	-5.32105300	-0.24035400	-1.89121300
H	-3.44492400	-0.38597800	-0.86794300
C	-5.91102300	1.95520800	-2.70453400
H	-4.48069500	3.51744900	-2.34501000
C	-6.22467800	0.59538400	-2.55934000
H	-5.55113000	-1.28665200	-1.72120900
H	-6.61359200	2.59940400	-3.22220800
C	-7.53393600	0.11155400	-3.11421900
O	-8.46304200	0.88919200	-3.33785800
N	-7.64738100	-1.23334800	-3.32487300
H	-8.48330900	-1.54438200	-3.80328500
C	-3.31982500	4.31457300	0.10801500
C	-3.04983900	5.68988600	-0.00061500
C	-4.55434100	3.91334300	0.65159300
C	-3.98265300	6.63394100	0.42333600
H	-2.11659400	6.02661800	-0.44183400
C	-5.48534700	4.85691300	1.06443400
H	-4.77852600	2.85854500	0.74629600
C	-5.20842700	6.22828200	0.96838500
H	-3.75447900	7.68697200	0.29165500
H	-6.43859100	4.54467200	1.47693600
C	5.30906000	2.44198100	0.23304200
C	5.50097100	3.06039200	1.47900600
C	6.38641400	2.37472800	-0.66831800
C	6.74976600	3.56876200	1.83060800

H	4.67634500	3.12262300	2.18178800
C	7.62680200	2.89178700	-0.31759000
H	6.24069200	1.91766500	-1.64142000
C	7.82813500	3.47973000	0.94005200
H	6.86409400	4.05441500	2.79451200
H	8.45815000	2.85144400	-1.01298700
C	4.70728800	-0.42927000	-0.88432600
C	4.67002600	-1.27530200	-2.00689000
C	5.65320800	-0.70940900	0.12319400
C	5.51274800	-2.38079900	-2.09710500
H	3.97017600	-1.06442500	-2.81225400
C	6.50409600	-1.80169400	0.02316800
H	5.70980000	-0.06999800	0.99463600
C	6.42653000	-2.67046000	-1.07586100
H	5.46132100	-2.99875700	-2.98838700
H	7.22258100	-2.01326500	0.80762800
C	7.34072200	-3.86286700	-1.08584400
O	8.35607200	-3.89898300	-0.38760400
C	9.19939000	4.01564200	1.24626500
O	9.99684100	4.28592600	0.34640400
C	-6.26799100	7.18649200	1.43601600
O	-7.44125600	6.83020500	1.56458600
N	9.49989300	4.20915800	2.56251400
H	8.98531300	3.74202700	3.29568600
H	10.45486900	4.46599900	2.77720900
N	-5.87126400	8.46765100	1.68231100
H	-6.55830300	9.08610900	2.09369500
H	-4.90199200	8.70098500	1.84414300
N	6.98850500	-4.89633900	-1.89964900
H	7.55549400	-5.73308700	-1.86963100
H	6.08467300	-4.96457400	-2.34335000
H	-6.82908700	-1.81533300	-3.43725900
C	2.45982800	0.41261700	2.94475100
C	1.11575300	0.85702400	2.90858000
C	0.15239900	0.07406200	2.29372100
C	1.74207600	-1.51269400	1.67079600
C	2.76410300	-0.80624600	2.29223900
H	0.85617800	1.78748100	3.39777800
H	3.77747300	-1.18093000	2.28625100
C	1.96402900	-2.79180800	0.93626000
C	3.23092600	-3.30781800	0.65192600
C	3.33475200	-4.49925200	-0.06433100
H	4.12721000	-2.78531200	0.95710300
C	0.94707900	-4.57253800	-0.16245900
C	2.17474200	-5.14952600	-0.48248000
H	4.31372900	-4.90529500	-0.29728700
H	0.00899400	-5.02633800	-0.46722500
H	2.21296700	-6.07559800	-1.04484500
C	-1.29979400	0.41187200	2.30588500
C	-1.79965600	1.59913400	2.85175000
C	-3.45191500	-0.33062800	1.85323500
C	-3.17725300	1.79905000	2.90237800
H	-1.13181000	2.36167800	3.23050500
C	-4.02595400	0.81095100	2.40645000
H	-4.06936100	-1.12015800	1.43981800
H	-3.57885200	2.71706800	3.31789100
H	-5.10439400	0.91981900	2.43323400
N	-2.12860500	-0.52540900	1.79503700
N	0.46432600	-1.09097000	1.69008900
N	0.84536200	-3.42958100	0.52717400

N	3.40161300	1.13805900	3.59454800
H	3.08083900	1.95093600	4.10182500
C	4.74478200	0.66242500	3.89102300
H	5.22160400	1.37755100	4.56213600
H	4.72460200	-0.31994400	4.37820900
H	5.35396700	0.59640700	2.98453000
C	-5.67125300	-4.22982700	0.82928500
C	-5.00088900	-3.82765800	-0.35663900
C	-3.67084300	-3.45038900	-0.28793600
C	-3.59200000	-3.85088600	2.00927700
C	-4.92346500	-4.24455700	2.03383500
H	-5.54375000	-3.82027600	-1.29481400
H	-5.39386900	-4.54893100	2.95798800
C	-2.71690300	-3.82493100	3.21940600
C	-3.12101700	-4.30480500	4.46873800
C	-2.23298200	-4.23948900	5.54191200
H	-4.10712100	-4.72994700	4.60958900
C	-0.63404200	-3.24248800	4.06977600
C	-0.96345500	-3.69854300	5.34501400
H	-2.53246500	-4.60920800	6.51735900
H	0.33707800	-2.80529500	3.86011000
H	-0.24372400	-3.62884100	6.15290300
C	-2.86776700	-3.01296900	-1.46590700
C	-3.31224600	-3.10539800	-2.78732300
C	-0.83197600	-2.13425300	-2.16864700
C	-2.47426800	-2.67753300	-3.81853700
H	-4.28709400	-3.51759200	-3.01828900
C	-1.20730200	-2.18566400	-3.50907000
H	0.14425500	-1.76330900	-1.87958100
H	-2.80927800	-2.73966600	-4.84890000
H	-0.51498200	-1.84429900	-4.27140800
N	-1.64180600	-2.52077100	-1.17627500
N	-2.99017000	-3.44674900	0.87650500
N	-1.48237400	-3.30545200	3.03711600
N	-6.97313600	-4.58318500	0.79170800
H	-7.44634400	-4.53629900	-0.09941200
C	-7.75141600	-4.99336000	1.94996500
H	-8.76777000	-5.21216200	1.62101900
H	-7.33216700	-5.89548600	2.41117300
H	-7.79207500	-4.19861300	2.70472300
Zn	-1.09192900	-2.40781400	0.98809600
Pt	1.71556600	0.23352500	-3.28696300

Sum of electronic and thermal Free Energies = -5280.960807 (Hartree/Particle).

Supplementary Table 24. DFT-optimized geometry of **(Pt_3-cluster)**-bound model (singlet), computed at the B3LYP-D3/ LANL2DZ (Zn, Pt)/ 6-31G* (HCNOS) level in acetonitrile solvent using PCM.

Atom	x	y	z
C	-3.80377700	2.42484000	1.24502400
C	-4.04356800	3.09140800	0.08813300
C	2.46016100	3.85276400	0.91411200
S	0.60836600	4.61105400	-0.87983700
S	1.03393200	3.06401700	1.62108800
S	-2.09904600	2.24152200	1.72477600
S	-2.62388500	3.67966900	-0.81743700
C	-0.12061100	3.59060700	0.37857000
C	-1.42106600	3.22445700	0.40884300
C	2.26866600	4.55057100	-0.23375200
C	3.71537700	3.69788000	1.67821300
C	4.05578300	2.46037800	2.25231900
C	4.58550100	4.78833200	1.86278500
C	5.23619900	2.31483500	2.97799000
H	3.40360400	1.60365500	2.11431000
C	5.77190400	4.63318300	2.56718500
H	4.32489300	5.75487400	1.44634800
C	6.10715100	3.39909700	3.14165200
H	5.49441700	1.34030900	3.38219100
H	6.45726600	5.46469300	2.69215600
C	7.41692300	3.30517200	3.87518800
O	8.33987100	4.07045200	3.63425900
N	7.52328600	2.28710500	4.79656900
H	8.36140100	2.31445600	5.36448800
C	3.29735500	5.20895100	-1.06752800
C	3.06495700	6.47742600	-1.62439300
C	4.51902100	4.56487400	-1.34253800
C	4.02455200	7.08197700	-2.43433600
H	2.14167700	7.00485500	-1.40291000
C	5.47632200	5.17483400	-2.14167500
H	4.71857600	3.59084000	-0.90880900
C	5.23494100	6.43359300	-2.70930400
H	3.83396400	8.07971100	-2.81789200
H	6.42638600	4.69197900	-2.34416900
C	-5.36268500	3.44097100	-0.47933300
C	-5.61672600	3.30894500	-1.85483600
C	-6.37734900	3.95608100	0.34783400
C	-6.85898900	3.65318100	-2.38464300
H	-4.83780700	2.93317800	-2.51198400
C	-7.61343100	4.29926300	-0.18387400
H	-6.18253800	4.09584200	1.40570300
C	-7.87453200	4.14075600	-1.55210000
H	-7.01469900	3.57417500	-3.45648500
H	-8.39507600	4.71343200	0.44391700
C	-4.76536300	1.73223200	2.12757100
C	-4.57162600	1.72174100	3.51966000
C	-5.83704200	0.98774000	1.59531500
C	-5.39867900	0.96705500	4.35047400
H	-3.76810200	2.30626600	3.95731300
C	-6.64617400	0.21910300	2.42079700
H	-6.01278800	0.99968800	0.52618500
C	-6.42533400	0.19045000	3.80579200
H	-5.21205100	0.94918700	5.42018300
H	-7.43665200	-0.39703200	2.00358800
C	-7.20834800	-0.78820400	4.63340500
O	-7.43404500	-1.92111500	4.21206000
C	-9.23464100	4.56022000	-2.04657300
O	-9.94900300	5.30306700	-1.38545500

C	6.32620600	7.02576100	-3.56157000
O	7.48265900	6.63084300	-3.47873400
N	-9.60831300	4.09293700	-3.28107300
H	-9.19921800	3.25548600	-3.66891700
H	-10.56214200	4.29326000	-3.55274100
N	5.95669000	8.04289500	-4.40445500
H	6.67109200	8.35725600	-5.04823900
H	5.00024200	8.14061800	-4.71343200
N	-7.58329600	-0.36700400	5.87290900
H	-8.15583000	-0.99440300	6.42293500
H	-7.60167800	0.61434300	6.11140000
H	6.69317200	1.94985100	5.26362400
C	-3.00109700	-0.14299600	-1.57960000
C	-1.59916700	0.11310500	-1.81761300
C	-0.68714000	-0.21602800	-0.77382800
C	-2.39340800	-1.06169700	0.58298700
C	-3.37144700	-0.76182600	-0.36837000
H	-1.32596900	0.82686000	-2.58847700
H	-4.41100800	-0.97580200	-0.16884000
C	-2.70461700	-1.73421100	1.87686600
C	-4.00335600	-1.94792300	2.33999900
C	-4.20233500	-2.57704500	3.56941200
H	-4.85981300	-1.60870400	1.77526700
C	-1.82122700	-2.74923300	3.77211300
C	-3.08969800	-2.98496900	4.30363100
H	-5.21564900	-2.72659700	3.93177500
H	-0.92146900	-3.05788200	4.29663300
H	-3.19336600	-3.47887500	5.26348300
C	0.74013300	0.21532200	-0.79327100
C	1.21479200	1.28055200	-1.54703100
C	2.89794500	-0.29305600	-0.05965500
C	2.58459600	1.54979600	-1.55660500
H	0.53344400	1.90494300	-2.10940400
C	3.44434200	0.75057900	-0.80561000
H	3.51331200	-0.93106100	0.56758100
H	2.96801600	2.37846000	-2.14134600
H	4.51355200	0.92822900	-0.78630500
N	1.57378300	-0.56961200	-0.04966500
N	-1.09488900	-0.79120200	0.39286300
N	-1.63064900	-2.14227400	2.59450400
N	-3.89638600	0.20761800	-2.52233700
H	-3.53287200	0.59389000	-3.38257000
C	-5.32138700	-0.09075500	-2.46651700
H	-5.77727100	0.22319200	-3.40574500
H	-5.49556500	-1.16458500	-2.33580100
H	-5.80408200	0.46091300	-1.65405400
C	4.57196000	-4.15051400	2.29563900
C	4.24687700	-2.93915900	2.96498000
C	2.97946900	-2.40314000	2.82025100
C	2.31718900	-4.14758800	1.41142300
C	3.56169300	-4.75594800	1.50633000
H	4.99598000	-2.45688500	3.58264300
H	3.76377000	-5.68351200	0.98964100
C	1.17581500	-4.70619700	0.62616400
C	1.22918800	-5.92731700	-0.04835300
C	0.10922500	-6.36258000	-0.75557600
H	2.12492400	-6.53578400	-0.02898500
C	-1.01494900	-4.36781000	-0.06476800
C	-1.03503000	-5.56885600	-0.77260000
H	0.13551100	-7.30766700	-1.28818000

H	-1.87647100	-3.70953000	-0.05256100
H	-1.92347000	-5.86584000	-1.31753500
C	2.51700400	-1.15158300	3.49164100
C	3.26603700	-0.46186800	4.44854600
C	0.76970700	0.37109700	3.70527100
C	2.72977500	0.68440400	5.03642900
H	4.24415200	-0.81878600	4.74883900
C	1.45681000	1.11214400	4.66511100
H	-0.22205800	0.65564300	3.36922500
H	3.29885600	1.23215000	5.78046800
H	1.00573800	1.99800400	5.09714000
N	1.28512900	-0.72245900	3.13280300
N	2.04628800	-2.99054600	2.04403400
N	0.05327000	-3.95009000	0.61792300
N	5.80105700	-4.69328000	2.41993100
H	6.46598700	-4.21844800	3.01329400
C	6.23100000	-5.92912800	1.78077800
H	7.26870200	-6.11874600	2.05686500
H	5.62388300	-6.77985800	2.11203600
H	6.17329300	-5.85044400	0.68875600
Zn	0.30176500	-1.90677000	1.52071000
Pt	1.69881400	-2.41652200	-1.59914500
Pt	-0.64818000	-1.85188700	-2.42028400
Pt	0.60888900	-3.91617700	-3.28149200

Sum of electronic and thermal Free Energies = -5519.337016 (Hartree/Particle).

Supplementary Table 25. DFT-optimized geometry of **(Pt_4-cluster)**-bound model (singlet), computed at the B3LYP-D3/ LANL2DZ (Zn, Pt)/ 6-31G* (HCNOS) level in acetonitrile solvent using PCM.

Atom	x	y	z
C	-3.37773700	2.67092500	2.41464000
C	-3.74842500	3.68968100	1.59826200
C	2.71884700	3.87959100	1.25194700
S	0.64632400	5.36793000	0.42212500
S	1.46608000	3.27144500	2.36022300
S	-1.73026600	2.75819800	3.11253500
S	-2.51573100	4.94943200	1.29770100
C	0.12294900	4.17595600	1.62650700
C	-1.17310200	3.99326700	1.96612800
C	2.33724200	4.81582200	0.34768500
C	4.08832900	3.36142900	1.44737900
C	4.33008000	1.98731800	1.60815200
C	5.18469400	4.24517500	1.44691500
C	5.63423800	1.50807700	1.70476800
H	3.50336800	1.28472400	1.60839300
C	6.48232900	3.76155100	1.54502800
H	5.00932700	5.31122600	1.35281000
C	6.72437300	2.38560200	1.66989400
H	5.78876600	0.43752800	1.75074500
H	7.32740100	4.44106100	1.52355400
C	8.15274700	1.92489200	1.72224900

O	9.07537400	2.65208700	1.34976500
N	8.37111600	0.65117700	2.16458500
H	9.33724600	0.38391800	2.30393800
C	3.14792300	5.38233300	-0.75137700
C	3.06788400	6.74278100	-1.09246300
C	3.98748800	4.54714900	-1.51057800
C	3.80096600	7.25021300	-2.16384800
H	2.44705500	7.41506000	-0.50761200
C	4.72440100	5.05640500	-2.57040400
H	4.06312300	3.49822900	-1.25485800
C	4.63036400	6.41126600	-2.92104800
H	3.74134600	8.31245600	-2.37960000
H	5.37521800	4.40972300	-3.14934600
C	-5.07821000	3.89179000	0.98902000
C	-5.20648400	4.35590100	-0.33194000
C	-6.24875500	3.63637000	1.72917400
C	-6.46325800	4.51143600	-0.91228400
H	-4.32047300	4.58674100	-0.91354000
C	-7.49990800	3.79889100	1.14940100
H	-6.16915000	3.31400500	2.76146700
C	-7.62391600	4.21870100	-0.18331600
H	-6.52653800	4.88620500	-1.92904300
H	-8.40125700	3.60750700	1.72157400
C	-4.14945800	1.45054700	2.72860600
C	-4.23001700	0.95897800	4.04171400
C	-4.76349900	0.71937400	1.69477000
C	-4.88106800	-0.24397500	4.31326800
H	-3.78757600	1.52154500	4.85845400
C	-5.41303200	-0.47908200	1.97195900
H	-4.71196000	1.07941200	0.67344000
C	-5.45650100	-0.99147900	3.27624600
H	-4.94488800	-0.57855700	5.34391100
H	-5.88502300	-1.04299900	1.17380900
C	-6.13068900	-2.32129200	3.47234300
O	-6.86989000	-2.79655500	2.60690500
C	-9.01180100	4.36589300	-0.74179000
O	-9.99069800	4.48760500	-0.00268700
C	5.45483000	6.88754400	-4.08359300
O	6.43009000	6.24706100	-4.48199900
N	-9.12561000	4.38353800	-2.10104400
H	-8.39877200	4.01395000	-2.69735800
H	-10.06451700	4.37401500	-2.47823600
N	5.08658400	8.07102500	-4.65329300
H	5.57346400	8.33812300	-5.49918600
H	4.15781100	8.44926500	-4.53227400
N	-5.89664200	-2.96311300	4.65099000
H	-6.26691900	-3.90020300	4.74247800
H	-5.11591600	-2.72635300	5.24606500
H	7.69271500	0.17069700	2.73865800
C	-0.03584600	1.74655900	-0.92802100
C	1.32448600	1.64698200	-1.25482700
C	2.10518200	0.63596000	-0.67261700
C	0.32921400	-0.09039600	0.69928100
C	-0.57626000	0.84239500	0.06456000
H	1.73618600	2.31430900	-1.99997600
H	-1.47761700	1.15052400	0.58016400
C	0.03059500	-0.72309400	2.01053900
C	-1.24676200	-0.71567800	2.59467200
C	-1.44356800	-1.37316900	3.80285200
H	-2.07142700	-0.21609100	2.10580200

C	0.85851700	-2.00940800	3.77551300
C	-0.37249700	-2.03369200	4.41609300
H	-2.42638500	-1.37136600	4.26026900
H	1.72621000	-2.51734700	4.18589800
H	-0.49184700	-2.55833300	5.35718800
C	3.49950500	0.37691300	-1.13371400
C	4.07493000	1.06376400	-2.21254400
C	5.45321000	-0.81053800	-0.80194200
C	5.40249900	0.81313000	-2.55371200
H	3.50374200	1.78025600	-2.78840200
C	6.11684000	-0.13603000	-1.82688500
H	5.96832300	-1.57508200	-0.23230900
H	5.86383600	1.35014900	-3.37649500
H	7.15366900	-0.36344700	-2.04844100
N	4.18406900	-0.56867300	-0.45890200
N	1.65510300	-0.17458800	0.29269800
N	1.05581000	-1.36997700	2.60912200
N	-0.86541000	2.61224000	-1.54247300
H	-0.46762500	3.18710500	-2.27319700
C	-2.31519600	2.45237100	-1.54021000
H	-2.73904700	3.15338300	-2.25978000
H	-2.59311600	1.42359900	-1.81485300
H	-2.73570800	2.67158300	-0.55685500
C	5.60129000	-5.14867300	-0.67737800
C	6.02096300	-4.35416700	0.42592600
C	5.09776000	-3.53335200	1.04724200
C	3.36285200	-4.28373500	-0.33623000
C	4.23097800	-5.12323800	-1.02953700
H	7.05264700	-4.40080300	0.75458000
H	3.85402000	-5.78471500	-1.79767300
C	1.88252100	-4.30496700	-0.47587500
C	1.19145100	-5.04724700	-1.40491500
C	-0.22706300	-5.25366400	-1.25618200
H	1.71073000	-5.54521800	-2.21749000
C	-0.04824600	-3.85994800	0.74535200
C	-0.85155300	-4.75453800	-0.05243700
H	-0.66174700	-6.10127000	-1.78333600
H	-0.51318000	-3.34078000	1.57851400
H	-1.70261000	-5.24626300	0.41437100
C	5.40279000	-2.66475000	2.22543300
C	6.60285600	-2.72628000	2.94033600
C	4.57738600	-1.03891600	3.67932900
C	6.76885500	-1.91562000	4.06426200
H	7.39161800	-3.40601200	2.64251300
C	5.73971300	-1.05464100	4.44847400
H	3.74663400	-0.38236200	3.91829100
H	7.69186300	-1.96115200	4.63365100
H	5.82999100	-0.41100100	5.31592600
N	4.41589200	-1.81637000	2.60126200
N	3.81654400	-3.46802900	0.63662500
N	1.22450800	-3.61631100	0.53338600
N	6.49551600	-5.92403600	-1.33261100
H	7.44927600	-5.92516500	-1.00084400
C	6.16703200	-6.79356600	-2.44974700
H	7.08484400	-7.26761800	-2.79965700
H	5.45918400	-7.57855500	-2.15481500
H	5.73117800	-6.22407000	-3.27941100
Zn	2.66909100	-1.79796300	1.22458700
Pt	-1.51298800	-3.52252700	-1.64583800
Pt	-2.70362000	-1.54963100	-2.87021000

Pt	-0.85546600	-1.03001100	-0.90844800
Pt	-3.28258900	-1.51015500	-0.42086500

Sum of electronic and thermal Free Energies = -5638.558772 (Hartree/Particle).

Supplementary Table 26. DFT-optimized geometry of TTF---Zn(TPY)$_2$ π-π stacking model (singlet), computed at the B3LYP-D3/ LANL2DZ (Zn)/ 6-31G* (HCNOS) level in acetonitrile solvent using PCM.

Atom	x	y	z
C	3.78319600	0.83540800	-1.21380200
C	4.04219400	2.02890800	-0.62120200
C	-2.52480600	2.14099700	-1.30620200
S	-0.63370900	3.74580000	-0.27880200
S	-1.09090400	1.15019900	-1.65080200
S	2.07919700	0.48030500	-1.58080200
S	2.65029200	3.06180600	-0.22650200
C	0.09139400	2.25790100	-0.92480200
C	1.41409400	1.98110400	-0.90200200
C	-2.31830800	3.32909700	-0.68600200
C	-3.80470500	1.56769400	-1.77220200
C	-4.07050200	0.20119400	-1.58200200
C	-4.76850600	2.35589300	-2.42670200
C	-5.27120100	-0.35880800	-2.00510200
H	-3.33950100	-0.42230500	-1.08260200
C	-5.96890500	1.79479100	-2.84450200
H	-4.57010800	3.40679300	-2.60390200
C	-6.23370300	0.43149000	-2.64580200
H	-5.46070000	-1.40570800	-1.79430200
H	-6.71760600	2.40248900	-3.34060200
C	-7.55690200	-0.10341200	-3.11480200
O	-8.51800300	0.64438600	-3.30820200
N	-7.64270000	-1.45241200	-3.28850200
H	-8.50039900	-1.80641400	-3.68800200
C	-3.33911000	4.30149500	-0.24070200
C	-3.14181200	5.68049600	-0.42620200
C	-4.51600900	3.86949300	0.39809800
C	-4.09081400	6.59999400	0.01499800
H	-2.25341300	6.03589700	-0.93870200
C	-5.46431100	4.78789200	0.82769800
H	-4.68090700	2.81079300	0.55129800
C	-5.25921300	6.16419200	0.65439800
H	-3.92351600	7.65519400	-0.17540200
H	-6.37411000	4.45379000	1.31389800
C	5.37159300	2.60401100	-0.32600200
C	5.63759200	3.21201100	0.91149800
C	6.38419300	2.58021300	-1.30240200
C	6.89559100	3.74531300	1.18289800
H	4.86329200	3.24661000	1.67019800
C	7.63519200	3.11791500	-1.03010200
H	6.18019400	2.13851200	-2.27150200
C	7.91169100	3.69171500	0.21939800
H	7.06549000	4.22141400	2.14299800

H	8.41639200	3.10721600	-1.78200200
C	4.73219800	-0.25429000	-1.52160200
C	4.53220000	-1.08299100	-2.63990200
C	5.81459900	-0.54018800	-0.66660200
C	5.36280200	-2.17458900	-2.87930200
H	3.72639900	-0.87199200	-3.33560200
C	6.65250100	-1.61798700	-0.91770200
H	5.98839800	0.07891200	0.20379800
C	6.42280200	-2.46568700	-2.01160200
H	5.18370300	-2.77949000	-3.76220200
H	7.48110100	-1.83728600	-0.25370200
C	7.34060400	-3.64368600	-2.18230200
O	8.45720400	-3.66578400	-1.65940200
C	9.28859000	4.25471800	0.43819800
O	10.01389000	4.55991900	-0.51120200
C	-6.33171500	7.09619000	1.14469800
O	-7.48441400	6.70108800	1.33559800
N	9.67919000	4.42591800	1.73129800
H	9.21379100	3.95861800	2.49399800
H	10.63259000	4.72092000	1.88699800
N	-5.97011700	8.39379100	1.34429800
H	-6.66281800	9.00538900	1.75199800
H	-5.00461700	8.66919200	1.43699800
N	6.87160600	-4.68048700	-2.92620200
H	7.45980800	-5.49448600	-3.02780200
H	5.92200600	-4.74058800	-3.25710200
H	-6.82019900	-2.02591100	-3.39630200
C	2.89449700	0.45640600	2.49139800
C	1.54429600	0.88300400	2.50609800
C	0.57019800	0.08700200	1.92489800
C	2.15810000	-1.47779500	1.24569800
C	3.19279900	-0.75759300	1.82739800
H	1.28989500	1.81090300	3.00199800
H	4.21030000	-1.11629100	1.77959800
C	2.37000300	-2.74769500	0.49269800
C	3.63300400	-3.25029200	0.17039800
C	3.72790600	-4.42619200	-0.57140200
H	4.53190300	-2.72519100	0.46229800
C	1.33950600	-4.51709600	-0.61270200
C	2.56300700	-5.07699400	-0.97430200
H	4.70290600	-4.81849000	-0.84090200
H	0.39800700	-4.97299800	-0.90290200
H	2.59450800	-5.99029400	-1.55700200
C	-0.88330300	0.41260000	1.96829800
C	-1.37710500	1.62219900	2.46869800
C	-3.04040100	-0.36350400	1.60569800
C	-2.75420500	1.81769600	2.54009800
H	-0.70440600	2.40660000	2.78909800
C	-3.60780400	0.80069500	2.11669800
H	-3.66320000	-1.17680500	1.25059800
H	-3.15240700	2.75409600	2.91519800
H	-4.68570400	0.90239300	2.16759800
N	-1.71770100	-0.55130200	1.51959800
N	0.87650000	-1.07579700	1.31659800
N	1.24660400	-3.38739700	0.10019800
N	3.85149600	1.19600800	3.10009800
H	3.54499400	2.00020700	3.62709800
C	5.22589700	0.75951000	3.28989800
H	5.73989500	1.49611100	3.90679800
H	5.27479800	-0.21468900	3.79019800

H	5.75749700	0.69401100	2.33589800
C	-5.22509400	-4.32530800	0.83029800
C	-4.64589500	-3.90970700	-0.39790200
C	-3.32119600	-3.50900500	-0.42060200
C	-3.07669500	-3.90800400	1.86419800
C	-4.39609400	-4.32420700	1.97999800
H	-5.25139500	-3.91150800	-1.29630200
H	-4.79659400	-4.63370700	2.93419800
C	-2.12489500	-3.86590300	3.01439800
C	-2.43789400	-4.35160300	4.28759800
C	-1.48299500	-4.26850100	5.29989800
H	-3.40409400	-4.79470500	4.49339800
C	-0.00139600	-3.24609900	3.72639800
C	-0.23889600	-3.70579900	5.02009800
H	-1.71099400	-4.64140200	6.29289800
H	0.94630300	-2.79289700	3.45419800
H	0.53050400	-3.62219800	5.77889800
C	-2.61739700	-3.05060300	-1.65300200
C	-3.15869700	-3.15540400	-2.93760200
C	-0.66739800	-2.11290000	-2.50520200
C	-2.41329700	-2.70630300	-4.02670200
H	-4.13639600	-3.59270600	-3.09590200
C	-1.14049800	-2.17890100	-3.81330200
H	0.31610100	-1.71789800	-2.27590200
H	-2.82149700	-2.77750400	-5.02920200
H	-0.52559900	-1.82740000	-4.63340200
N	-1.38879800	-2.52460100	-1.45520200
N	-2.55959600	-3.49510300	0.69279800
N	-0.91489600	-3.32500000	2.75169800
N	-6.51889400	-4.70421000	0.88329800
H	-7.05469400	-4.66861100	0.02889800
C	-7.20299300	-5.12721200	2.09489800
H	-8.23569300	-5.36691300	1.84299800
H	-6.73519100	-6.01971100	2.52589800
H	-7.20479400	-4.33331200	2.85069800
Zn	-0.68139800	-2.42430000	0.68249800

Sum of electronic and thermal Free Energies = -5161.785691 (Hartree/Particle).

Supplementary Table 27. DFT-optimized geometry of **Pt** (singlet), computed at the B3LYP-D3/ LANL2DZ level in acetonitrile solvent using PCM.

Atom	x	y	z
Pt	0.00000000	0.00000000	0.00000000

Sum of electronic and thermal Free Energies = -119.087980 (Hartree/Particle).

Supplementary Table 28. Structure of **Pt_3-cluster** (singlet) for single point calculation, computed at the B3LYP-D3/ LANL2DZ level in acetonitrile solvent using PCM.

Atom	x	y	z
Pt	0.00000000	1.60180300	0.00000000
Pt	-1.38720200	-0.80090200	0.00000000
Pt	1.38720200	-0.80090200	0.00000000

Sum of electronic and thermal Free Energies = -357.480079 (Hartree/Particle).

Supplementary Table 29. Structure of **Pt_4-cluster** (singlet) for single point calculation, computed at the B3LYP-D3/ LANL2DZ level in acetonitrile solvent using PCM.

Atom	x	y	z
Pt	0.98090000	0.98090000	0.98090000
Pt	-0.98090000	-0.98090000	0.98090000
Pt	-0.98090000	0.98090000	-0.98090000
Pt	0.98090000	-0.98090000	-0.98090000

Sum of electronic and thermal Free Energies = -476.646672 (Hartree/Particle).

Supplementary Table 30. DFT-optimized geometry of TTF (singlet), computed at the B3LYP/ 6-31G** (HCNOS) level in acetonitrile solvent using IEFPCM.

Atom	x	y	z
C	-3.18055000	0.66905100	-0.54061700
C	-3.17411400	-0.68453400	-0.53292200
S	-1.65195100	-1.48920700	-1.00542500
C	-0.67555800	-0.00182800	-1.01108500
S	-1.66272100	1.47878700	-1.01652900
C	0.67554200	0.00187400	-1.01108700
S	1.66269900	-1.47874500	-1.01655100
S	1.65194000	1.48924700	-1.00542000
C	3.18052900	-0.66902200	-0.54064200
C	3.17410900	0.68456400	-0.53293100
C	-4.29094100	-1.57319500	-0.14623900
C	-5.00097300	-1.34396000	1.04520100
C	-4.65272200	-2.66495500	-0.95183000
C	-6.05301600	-2.17496400	1.40765100
H	-4.72159000	-0.51041700	1.67987100
C	-5.69979300	-3.50376400	-0.57842600
H	-4.12948400	-2.84492800	-1.88570400
C	-6.40766500	-3.27063100	0.60845900
H	-6.60960100	-1.99502600	2.32061100
H	-5.97914500	-4.31799100	-1.23900000
C	-4.33779800	1.55207200	-0.28642000
C	-5.58452400	1.28459900	-0.88071600
C	-4.21022100	2.68215500	0.53809300
C	-6.67209600	2.11241000	-0.63719300
H	-5.69122400	0.42511100	-1.53276800

C	-5.30061400	3.51652800	0.77096000
H	-3.26016900	2.89457900	1.01754600
C	-6.54234300	3.24302900	0.18187100
H	-7.63665300	1.90325600	-1.08608900
H	-5.17946100	4.36051700	1.44205200
C	4.29094500	1.57320600	-0.14624800
C	4.65266300	2.66503400	-0.95177900
C	5.00106500	1.34389400	1.04512700
C	5.69975100	3.50382600	-0.57838300
H	4.12936400	2.84507000	-1.88560600
C	6.05312400	2.17488300	1.40756500
H	4.72173500	0.51030400	1.67975900
C	6.40771000	3.27061600	0.60843500
H	5.97903900	4.31810100	-1.23892700
H	6.60977100	1.99488200	2.32047300
C	4.33777500	-1.55205800	-0.28647300
C	4.21021600	-2.68210300	0.53809300
C	5.58447200	-1.28463100	-0.88085100
C	5.30060800	-3.51648900	0.77093000
H	3.26018500	-2.89448400	1.01760500
C	6.67204000	-2.11245800	-0.63735700
H	5.69114800	-0.42516600	-1.53293600
C	6.54230800	-3.24304300	0.18175700
H	5.17947700	-4.36044600	1.44206600
H	7.63657800	-1.90334200	-1.08631300
C	-7.55730400	-4.12394500	1.06567300
O	-8.39865800	-3.69355400	1.85764400
N	-7.63232000	-5.37982100	0.54448000
H	-8.35150200	-5.98529800	0.91351500
H	-6.83626500	-5.82459700	0.11430100
C	-7.76578100	4.08827400	0.40122000
O	-8.89763300	3.63516100	0.21684200
N	-7.56070000	5.36291500	0.83418600
H	-8.37339300	5.95890700	0.90000400
H	-6.66588900	5.81831400	0.74256400
C	7.55737500	4.12390000	1.06564900
O	8.39876200	3.69345100	1.85755700
N	7.63237900	5.37980700	0.54455200
H	8.35163500	5.98524000	0.91351000
H	6.83638600	5.82459600	0.11428100
C	7.76574400	-4.08830700	0.40106900
O	8.89759600	-3.63519900	0.21666300
N	7.56066200	-5.36294700	0.83399300
H	8.37334000	-5.95894100	0.89992300
H	6.66580700	-5.81830000	0.74260200

Sum of electronic and thermal Free Energies = -3423.076284 (Hartree/Particle).

Supplementary Table 31. DFT-optimized geometry of TTF (singlet), computed at the B3LYP/ 6-31G** (HCNOS) level in water solvent using IEFPCM.

Atom	x	y	z

C	3.18079100	-0.66897100	-0.53952800
C	3.17426300	0.68458800	-0.53151100
S	1.65184900	1.48939000	-1.00286100
C	0.67556300	0.00184300	-1.00868000
S	1.66268400	-1.47881800	-1.01437700
C	-0.67555000	-0.00186200	-1.00868000
S	-1.66266600	1.47882000	-1.01421700
S	-1.65185000	-1.48939700	-1.00299400
C	-3.18075100	0.66895000	-0.53948400
C	-3.17428500	-0.68461500	-0.53155100
C	4.29163500	1.57272700	-0.14513000
C	5.00192300	1.34261400	1.04600100
C	4.65388700	2.66440600	-0.95062800
C	6.05515100	2.17237800	1.40791800
H	4.72185800	0.50940200	1.68081200
C	5.70209300	3.50204500	-0.57770700
H	4.13022300	2.84526600	-1.88406700
C	6.41065500	3.26766200	0.60854800
H	6.61172200	1.99164800	2.32073900
H	5.98161200	4.31637000	-1.23807600
C	4.33870900	-1.55150600	-0.28675600
C	5.58411100	-1.28381000	-0.88372900
C	4.21332300	-2.68085300	0.53908500
C	6.67278800	-2.11042300	-0.64100000
H	5.68894600	-0.42512300	-1.53714400
C	5.30479700	-3.51409400	0.77112700
H	3.26426400	-2.89360100	1.02033800
C	6.54538400	-3.24003800	0.17985000
H	7.63624100	-1.90097300	-1.09214100
H	5.18543400	-4.35754600	1.44319600
C	-4.29164800	-1.57276800	-0.14521600
C	-4.65358300	-2.66474200	-0.95040000
C	-5.00226900	-1.34236300	1.04569100
C	-5.70180100	-3.50239600	-0.57746300
H	-4.12972600	-2.84592500	-1.88367600
C	-6.05546500	-2.17211400	1.40761800
H	-4.72243200	-0.50894500	1.68032100
C	-6.41067700	-3.26772300	0.60852000
H	-5.98100500	-4.31693300	-1.23769300
H	-6.61227900	-1.99119500	2.32025800
C	-4.33866600	1.55152600	-0.28676800
C	-4.21339600	2.68069900	0.53932100
C	-5.58394200	1.28401200	-0.88405300
C	-5.30486000	3.51395900	0.77129800
H	-3.26443000	2.89330300	1.02082300
C	-6.67262700	2.11063500	-0.64139700
H	-5.68869700	0.42544500	-1.53764400
C	-6.54533500	3.24008200	0.17969500
H	-5.18557900	4.35725900	1.44357200
H	-7.63599400	1.90131200	-1.09277900
C	7.56215300	4.11907200	1.06447900
O	8.40508700	3.68602700	1.85375900
N	7.63727800	5.37544800	0.54547500
H	8.35798300	5.97991400	0.91325400
H	6.84115900	5.82151700	0.11666900
C	7.76996100	-4.08353400	0.39918600
O	8.90135900	-3.62798500	0.21613100
N	7.56666600	-5.35863800	0.83053000
H	8.37992200	-5.95387000	0.89672900

H	6.67238300	-5.81512700	0.73861400
C	-7.56220700	-4.11906200	1.06454100
O	-8.40511900	-3.68591300	1.85379500
N	-7.63740600	-5.37550700	0.54576000
H	-8.35821400	-5.97986100	0.91351000
H	-6.84145900	-5.82163300	0.11669600
C	-7.76993400	4.08357300	0.39894700
O	-8.90131100	3.62809800	0.21556100
N	-7.56670600	5.35860000	0.83050300
H	-8.37994600	5.95382500	0.89689700
H	-6.67233600	5.81504400	0.73927200

Supplementary Table 32. DFT-optimized geometry of TTF^{+} (doublet), computed at the UB3LYP/ 6-31G** (HCNOS) level in acetonitrile solvent using IEFPCM.

Atom	x	y	z
C	3.22037800	0.68211000	0.01956000
C	3.22038400	-0.68210000	-0.01976800
S	1.64477500	-1.46233600	-0.05000400
C	0.69695900	0.00000200	-0.00013800
S	1.64477100	1.46234200	0.04974900
C	-0.69696000	-0.00000100	-0.00012400
S	-1.64477100	-1.46234000	0.04979000
S	-1.64477700	1.46233700	-0.04997800
C	-3.22037800	-0.68210900	0.01961300
C	-3.22038500	0.68210100	-0.01972200
C	4.40189900	-1.56592100	-0.10018400
C	5.41550200	-1.30117000	-1.03738400
C	4.52135400	-2.67824100	0.74733900
C	6.52807200	-2.12816500	-1.11110300
H	5.32379700	-0.45016300	-1.70256500
C	5.63509400	-3.50987500	0.66082100
H	3.75881800	-2.87823400	1.49322900
C	6.64529600	-3.24561100	-0.27300500
H	7.31925800	-1.92628500	-1.82405700
H	5.72158700	-4.34239800	1.35085200
C	4.40188900	1.56593200	0.10005100
C	5.41542200	1.30118100	1.03732600
C	4.52141000	2.67824200	-0.74747300
C	6.52799100	2.12817300	1.11112000
H	5.32366500	0.45017600	1.70250300
C	5.63514700	3.50987300	-0.66087900
H	3.75892600	2.87823200	-1.49341800
C	6.64527800	3.24561500	0.27302600
H	7.31912400	1.92629500	1.82413400
H	5.72169200	4.34238800	-1.35091300
C	-4.40190000	1.56592100	-0.10013500
C	-4.52134900	2.67824900	0.74737800
C	-5.41551200	1.30116100	-1.03732300
C	-5.63508900	3.50988300	0.66086100
H	-3.75880600	2.87824900	1.49325900
C	-6.52808300	2.12815500	-1.11104100
H	-5.32381300	0.45014800	-1.70249800

C	-6.64529900	3.24561000	-0.27295400
H	-5.72157600	4.34241200	1.35088500
H	-7.31927500	1.92626800	-1.82398700
C	-4.40188800	-1.56593100	0.10011600
C	-4.52141700	-2.67824200	-0.74740600
C	-5.41541300	-1.30118000	1.03740000
C	-5.63515300	-3.50987400	-0.66079900
H	-3.75894000	-2.87823300	-1.49335700
C	-6.52798000	-2.12817200	1.11120600
H	-5.32365000	-0.45017400	1.70257500
C	-6.64527400	-3.24561600	0.27311500
H	-5.72170300	-4.34239000	-1.35083200
H	-7.31910600	-1.92629400	1.82422700
C	7.88554000	-4.08820500	-0.40815900
O	8.91511100	-3.62831200	-0.90512100
N	7.81194200	-5.36124200	0.06404900
H	8.61211500	-5.95701900	-0.09351300
H	6.92902600	-5.81484400	0.24062700
C	7.88551600	4.08820600	0.40827300
O	8.91505500	3.62829900	0.90529000
N	7.81195500	5.36125200	-0.06390800
H	8.61212800	5.95701900	0.09368600
H	6.92905900	5.81485800	-0.24057500
C	-7.88554400	4.08820300	-0.40810600
O	-8.91512100	3.62830300	-0.90504800
N	-7.81193800	5.36124700	0.06407800
H	-8.61211500	5.95702200	-0.09347300
H	-6.92902100	5.81484800	0.24065500
C	-7.88551200	-4.08820600	0.40837400
O	-8.91504700	-3.62829800	0.90539800
N	-7.81195200	-5.36125400	-0.06379900
H	-8.61212800	-5.95702000	0.09378900
H	-6.92906100	-5.81485600	-0.24049600

Sum of electronic and thermal Free Energies = -3422.899262 (Hartree/Particle).

Supplementary Table 33. DFT-optimized geometry of $[Zn^{II}(TPY)_2]^{2+}$ (singlet), computed at the B3LYP-D3/ LANL2DZ (Zn)/ 6-31G** (HCN) level in acetonitrile solvent using IEFPCM.

Atom	x	y	z
C	4.90342200	0.12562500	0.32949600
C	4.09315500	0.96598000	1.13743600
C	2.71620800	0.89351700	1.02610100
C	2.86240400	-0.76826900	-0.60976700
C	4.24910600	-0.75961800	-0.56342500
H	4.56788800	1.65332100	1.82696000
H	4.82834300	-1.41297400	-1.19942000
C	2.07316800	-1.66107900	-1.51140200
C	2.65216600	-2.60165100	-2.36849100
C	1.82625000	-3.38991900	-3.16876400
H	3.72658600	-2.72665800	-2.41533200
C	-0.05933300	-2.26956200	-2.21813200
C	0.44346300	-3.22591300	-3.09749400
H	2.26155300	-4.12417600	-3.83843200

H	-1.12624400	-2.09513900	-2.11479100
H	-0.23102100	-3.82017900	-3.70286100
C	1.77303900	1.73244600	1.82431500
C	2.18751700	2.65946900	2.78531300
C	-0.45940500	2.25213000	2.22721500
C	1.22710700	3.39623300	3.47662900
H	3.23837900	2.80922600	2.99794200
C	-0.12397300	3.19413700	3.19687300
H	-1.49327200	2.04886500	1.96446700
H	1.53416800	4.11905300	4.22518200
H	-0.89968300	3.74770200	3.71304000
N	0.46099900	1.54345200	1.56230900
N	2.11856400	0.04139800	0.16636900
N	0.73160400	-1.51101700	-1.45007700
N	6.24925800	0.18126900	0.42045500
H	6.64910500	0.83709100	1.07446900
C	7.16330500	-0.63483400	-0.36221300
H	8.18368000	-0.38114100	-0.07597900
H	7.00615300	-1.70317300	-0.17401000
H	7.04790100	-0.44774900	-1.43613200
C	-4.90340800	0.12573300	-0.32960400
C	-4.09308000	0.96598500	-1.13759000
C	-2.71614000	0.89348100	-1.02620200
C	-2.86245600	-0.76813300	0.60982900
C	-4.24915600	-0.75944500	0.56342900
H	-4.56776100	1.65328400	-1.82719100
H	-4.82843800	-1.41272400	1.19946100
C	-2.07328500	-1.66088100	1.51158400
C	-2.65235200	-2.60130200	2.36879300
C	-1.82649600	-3.38953100	3.16916600
H	-3.72678200	-2.72621900	2.41565400
C	0.05917000	-2.26942800	2.21839800
C	-0.44369600	-3.22563600	3.09787500
H	-2.26185300	-4.12367000	3.83892600
H	1.12609400	-2.09509300	2.11503900
H	0.23074300	-3.81987800	3.70331500
C	-1.77291500	1.73228600	-1.82447900
C	-2.18732400	2.65912600	-2.78568500
C	0.45955600	2.25192600	-2.22728500
C	-1.22686400	3.39577700	-3.47705200
H	-3.23816900	2.80882000	-2.99844300
C	0.12419400	3.19375400	-3.19714000
H	1.49340300	2.04871900	-1.96441100
H	-1.53387100	4.11845300	-4.22576700
H	0.89994200	3.74723600	-3.71334200
N	-0.46089500	1.54335500	-1.56233000
N	-2.11855700	0.04142800	-0.16636000
N	-0.73171100	-1.51092000	1.45024800
N	-6.24923700	0.18140200	-0.42063400
H	-6.64903900	0.83724300	-1.07465800
C	-7.16334500	-0.63455900	0.36211100
H	-8.18369800	-0.38093200	0.07573900
H	-7.00616700	-1.70292900	0.17411200
H	-7.04803400	-0.44728100	1.43600700
Zn	-0.00000300	0.01704200	0.00005200

Sum of electronic and thermal Free Energies = -1739.796085 (Hartree/Particle).

Supplementary Table 34. DFT-optimized geometry of $[Zn^{II}(TPY)_2]^{2+}$ (singlet), computed at the B3LYP-D3/ LANL2DZ (Zn)/ 6-31G** (HCN) level in water solvent using IEFPCM.

Atom	x	y	z
C	-4.90295600	0.12201300	-0.32888700
C	-4.09348400	0.96284600	-1.13702400
C	-2.71652100	0.89197900	-1.02525300
C	-2.86130400	-0.76890600	0.61162700
C	-4.24799900	-0.76196700	0.56475400
H	-4.56883800	1.64920400	-1.82703600
H	-4.82670900	-1.41583100	1.20069100
C	-2.07140900	-1.65978500	1.51457200
C	-2.64995000	-2.59845200	2.37408600
C	-1.82358700	-3.38469100	3.17587200
H	-3.72434800	-2.72329500	2.42183100
C	0.06145600	-2.26599700	2.22213600
C	-0.44087000	-3.22047300	3.10382100
H	-2.25852500	-4.11745800	3.84741400
H	1.12828500	-2.09141600	2.11852400
H	0.23401400	-3.81305100	3.71040900
C	-1.77426000	1.73176200	-1.82359700
C	-2.18985400	2.65873400	-2.78418000
C	0.45769700	2.25331000	-2.22711700
C	-1.23023600	3.39642000	-3.47558900
H	-3.24091400	2.80776400	-2.99633400
C	0.12111300	3.19530600	-3.19639300
H	1.49182800	2.05073200	-1.96486700
H	-1.53815300	4.11922500	-4.22381100
H	0.89620800	3.74962500	-3.71266300
N	-0.46198400	1.54376900	-1.56209600
N	-2.11815600	0.04095800	-0.16494000
N	-0.72989400	-1.50954600	1.45242800
N	-6.24884600	0.17612600	-0.42034600
H	-6.64922500	0.83116300	-1.07484400
C	-7.16189000	-0.64054200	0.36275700
H	-8.18257900	-0.38766600	0.07690300
H	-7.00385400	-1.70880600	0.17481700
H	-7.04604200	-0.45328600	1.43660600
C	4.90292500	0.12218400	0.32943000
C	4.09328400	0.96258300	1.13783200
C	2.71633900	0.89172900	1.02578900
C	2.86146100	-0.76834400	-0.61189200
C	4.24815100	-0.76135600	-0.56479100
H	4.56848900	1.64863900	1.82825300
H	4.82699900	-1.41486800	-1.20096100
C	2.07175500	-1.65883000	-1.51539200
C	2.65046500	-2.59703900	-2.37528200
C	1.82425300	-3.38296600	-3.17754000
H	3.72487500	-2.72177200	-2.42296000
C	-0.06097900	-2.26487700	-2.22347800
C	0.44151300	-3.21890000	-3.10555200
H	2.25932000	-4.11537400	-3.84938900
H	-1.12783400	-2.09042800	-2.11988500
H	-0.23325800	-3.81124300	-3.71248500
C	1.77389500	1.73111900	1.82434800
C	2.18929400	2.65760200	2.78548900

C	-0.45812900	2.25251700	2.22762600
C	1.22954400	3.39497400	3.47704600
H	3.24031700	2.80649000	2.99796600
C	-0.12174900	3.19403500	3.19743900
H	-1.49221000	2.05009800	1.96505000
H	1.53730900	4.11739500	4.22570100
H	-0.89694500	3.74811600	3.71381500
N	0.46167900	1.54327500	1.56246700
N	2.11816300	0.04110500	0.16498000
N	0.73022100	-1.50870400	-1.45334200
N	6.24879800	0.17625200	0.42119600
H	6.64904600	0.83114700	1.07592300
C	7.16200000	-0.63975700	-0.36239300
H	8.18263700	-0.38706700	-0.07618700
H	7.00398000	-1.70817100	-0.17529800
H	7.04632200	-0.45170600	-1.43612100
Zn	0.00000400	0.01739300	-0.00016900

Supplementary Table 35. DFT-optimized geometry of $[Zn^{II}(TPY^{\bullet -})(TPY)]^{+}$ (doublet), computed at the UB3LYP-D3/ LANL2DZ (Zn)/ 6-31G** (HCN) level in acetonitrile solvent using IEFPCM. The spin density plot is also shown below. (Isovalue = 0.0004)

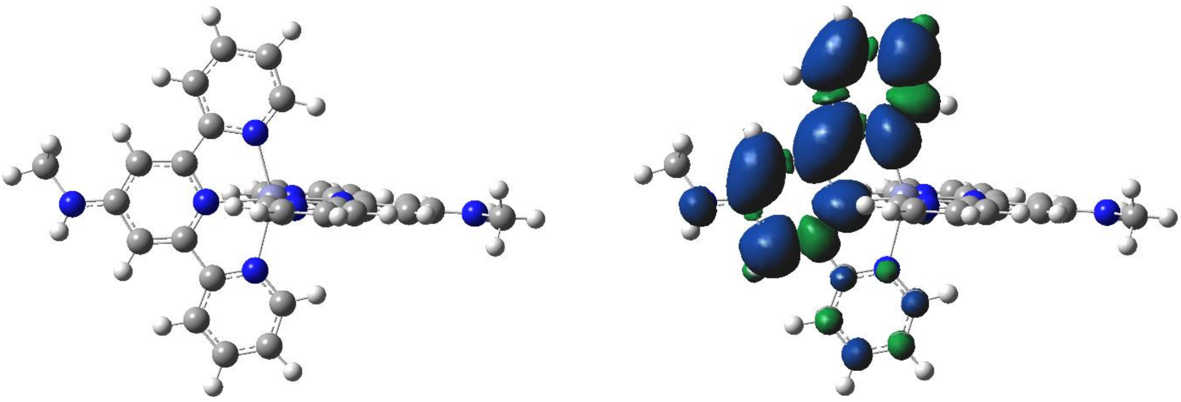

Spin Density Distribution

Atom	x	y	z
C	4.91187100	0.10249500	0.28801300
C	4.11846000	1.02211900	1.02058500
C	2.73851200	0.95114900	0.93351500
C	2.85046700	-0.85039400	-0.54391500
C	4.23921200	-0.85116400	-0.51421200
H	4.60638900	1.76860600	1.63539200
H	4.80487400	-1.56467700	-1.09577400
C	2.04854200	-1.80797500	-1.36473500
C	2.61308300	-2.81500400	-2.15458600
C	1.77299900	-3.65160700	-2.88840300
H	3.68604500	-2.95277600	-2.20180900
C	-0.09400300	-2.44547400	-2.00769900
C	0.39208800	-3.46829700	-2.81982900
H	2.19567700	-4.43751900	-3.50569300
H	-1.15773300	-2.24952500	-1.90634500

H	-0.29230600	-4.09819200	-3.37639200
C	1.81792800	1.87776800	1.65861300
C	2.25556700	2.88946400	2.52000100
C	-0.40220400	2.46615700	2.02630500
C	1.31138100	3.70507100	3.14186900
H	3.31044400	3.04449800	2.70907900
C	-0.04532900	3.49636000	2.89388700
H	-1.43997600	2.24729000	1.79100700
H	1.63501100	4.49407400	3.81277800
H	-0.80792600	4.11080600	3.35845800
N	0.50267300	1.68320500	1.42890500
N	2.12049200	0.02830100	0.16678600
N	0.71070400	-1.64229800	-1.30386100
N	6.26084700	0.15198200	0.36107300
H	6.67365600	0.85583800	0.95402600
C	7.15694100	-0.74748000	-0.34613700
H	8.18362100	-0.47788300	-0.09887900
H	6.99223700	-1.79069000	-0.05182500
H	7.03027100	-0.66737200	-1.43218700
C	-4.89052200	0.09445100	-0.43505700
C	-4.05118500	0.86527200	-1.30671200
C	-2.68212400	0.80398300	-1.12637400
C	-2.87847400	-0.70933800	0.68405600
C	-4.29660500	-0.67919200	0.55690100
H	-4.50213500	1.46848600	-2.08502300
H	-4.90960000	-1.25935900	1.23346800
C	-2.14991200	-1.47198100	1.65564500
C	-2.75074700	-2.32432600	2.62755700
C	-1.97218400	-3.01938800	3.52392700
H	-3.83044400	-2.42637900	2.65841900
C	-0.02413800	-2.04670400	2.51340200
C	-0.55545500	-2.88519200	3.47800900
H	-2.43746100	-3.66733700	4.26132100
H	1.05220200	-1.90471500	2.42775100
H	0.09112500	-3.41622200	4.16712500
C	-1.71027600	1.56563400	-1.96861200
C	-2.09069600	2.42029600	-3.01093200
C	0.53626700	2.02701600	-2.36870800
C	-1.10851400	3.08682200	-3.74037500
H	-3.13623600	2.56678300	-3.25172600
C	0.23573000	2.89067000	-3.41848000
H	1.56241100	1.83286800	-2.06855700
H	-1.39087200	3.75168900	-4.55042400
H	1.02909500	3.39085700	-3.96227800
N	-0.40545200	1.38488400	-1.66588900
N	-2.10904700	0.04704700	-0.17141100
N	-0.75992100	-1.35533100	1.62542300
N	-6.25190000	0.13026000	-0.63987400
H	-6.58935000	0.91959000	-1.17137500
C	-7.18945800	-0.43178500	0.31494500
H	-8.20540300	-0.22512200	-0.02523600
H	-7.06891900	-1.51910500	0.37928100
H	-7.06527700	-0.01739700	1.32671100
Zn	-0.03805100	-0.03766700	0.08419500

Sum of electronic and thermal Free Energies = -1739.905548 (Hartree/Particle).

Supplementary Table 36. DFT-optimized geometry of **TS1** (doublet), computed at the UB3LYP-D3/ LANL2DZ (Zn)/ 6-31G** (HCN) level in acetonitrile solvent using IEFPCM.

Atom	x	y	z
C	3.12799300	0.28447500	0.11087300
C	2.33655500	-1.81315600	-0.46511400
C	4.42494100	-0.04790100	-0.25070500
C	3.59968900	-2.22275800	-0.85315700
C	4.69919500	-1.33624500	-0.75929500
H	5.20751500	0.69059800	-0.13609200
H	3.75460200	-3.21519400	-1.25893500
C	-2.93924100	-0.37739200	0.98294900
C	-2.75403500	0.52301800	-1.19640900
C	-4.32425200	-0.37439500	0.94005500
C	-4.17607300	0.53617100	-1.31459000
C	-4.96357000	0.09950600	-0.25334200
H	-4.92869800	-0.71400900	1.77199800
H	-4.63920500	0.88906800	-2.22616600
C	2.89416200	1.65457700	0.66864300
C	2.85787900	1.85283400	2.05267400
C	2.73005500	3.15371300	2.53537600
H	2.93718400	1.00642900	2.72646800
C	2.66223900	3.89685800	0.25545100
C	2.62897500	4.20033700	1.61829200
H	2.70803900	3.34646600	3.60338200
H	2.57962200	4.68909800	-0.48539000
H	2.52895900	5.22969300	1.94593400
C	1.17547700	-2.73879600	-0.57925300
C	1.31279300	-4.13135900	-0.64811800
C	0.17051900	-4.91561700	-0.79179200
H	2.28664700	-4.59846600	-0.57024200
C	-1.13247700	-2.91013100	-0.75181700
C	-1.07844400	-4.29757000	-0.85825300
H	0.25662100	-5.99603600	-0.84497100
H	-2.07298400	-2.37040200	-0.76955800
H	-1.98992600	-4.87254400	-0.97555800
C	-1.82864400	0.96615700	-2.19929300
C	-2.21156200	1.49206400	-3.46801800
C	-1.25986600	1.91375100	-4.36759700
H	-3.26371800	1.56157800	-3.72371500
C	0.43964800	1.30734800	-2.78337600
C	0.11935000	1.82845200	-4.02550200
H	-1.56167800	2.31298300	-5.33186700
H	1.47518600	1.23145300	-2.45760400
H	0.89769100	2.15804800	-4.70412900
C	-2.15090700	-0.87120500	2.15360600
C	-2.73726300	-1.37310200	3.32221200
C	-1.92170200	-1.82372700	4.35830200
H	-3.81453600	-1.41441100	3.42435700
C	-0.02430300	-1.25732600	3.02162400
C	-0.53496800	-1.76946600	4.21174000
H	-2.36579200	-2.21451100	5.26829800
H	1.04567000	-1.19204000	2.84348100
H	0.13332500	-2.11277900	4.99326300
N	-6.34074700	0.13107400	-0.28532700
N	5.93571700	-1.72095600	-1.14162900

H	6.04961600	-2.65985800	-1.49259900
N	2.80358500	2.65298300	-0.22409400
N	2.07735800	-0.55880600	0.00698300
N	-0.03518000	-2.15554800	-0.61099200
N	-0.80521700	-0.81996900	2.02621500
N	-2.18527500	0.05010600	-0.04077300
N	-0.46856900	0.87928300	-1.88889100
H	-6.80165300	-0.46081200	0.39054000
C	-0.48567500	3.11332800	1.39815900
N	-0.16262700	2.05934900	1.04650700
C	-0.88590800	4.43902900	1.84539000
H	-0.37436300	4.68083100	2.78037200
H	-0.61350600	5.17883500	1.08853900
H	-1.96670300	4.46689100	2.00550600
C	7.11060800	-0.86674800	-1.08908100
H	7.32685900	-0.54703300	-0.06303400
H	7.96578600	-1.42993300	-1.46233500
H	6.98361700	0.02616900	-1.71221600
C	-7.07714100	0.37271000	-1.51262400
H	-6.80815700	-0.33036900	-2.31524300
H	-8.14472200	0.27749400	-1.30770900
H	-6.89271200	1.38873600	-1.87948800
Zn	-0.08187300	0.01089300	0.02702200

Sum of electronic and thermal Free Energies = -1872.659459 (Hartree/Particle).

Supplementary Table 37. DFT-optimized geometry of $[Zn^{II}(TPY^{\bullet -})(\eta^2\text{-}TPY)(CH_3CN)]^+$ (doublet), computed at the UB3LYP-D3/ LANL2DZ (Zn)/ 6-31G** (HCN) level in acetonitrile solvent using IEFPCM. The spin density plot is also shown below. (Isovalue = 0.0004)

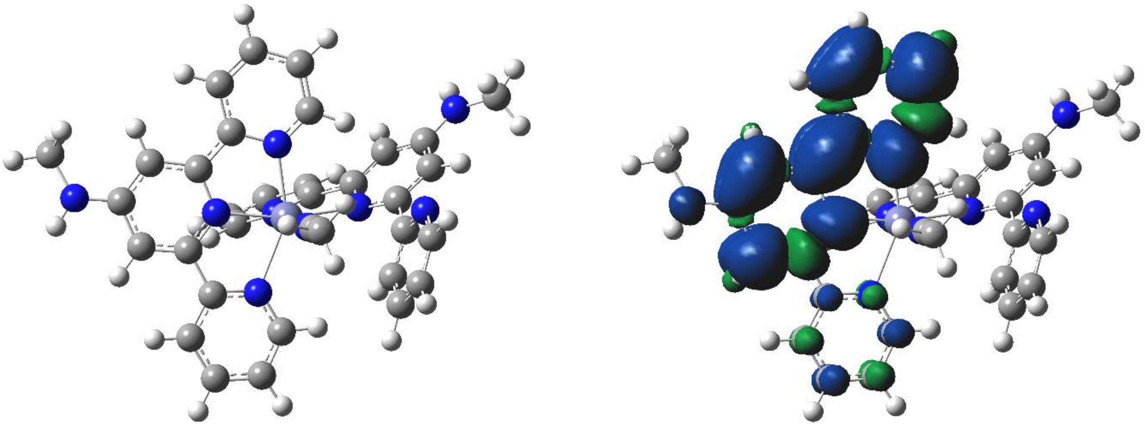

Spin Density Distribution

Atom	x	y	z
C	3.10910000	0.28905200	0.17176200
C	2.09112800	-1.38922600	1.40144800
C	4.24596700	-0.48232200	-0.03603000
C	3.19066800	-2.21801700	1.26168700
C	4.30567900	-1.78834700	0.49900900
H	5.06976500	-0.05618700	-0.59224900

H	3.19477800	-3.20820000	1.70202600
C	-3.15198000	0.72480700	0.17589400
C	-2.46360100	-1.05486100	-1.21693500
C	-4.49322200	0.49807000	-0.08329000
C	-3.82682500	-1.34714700	-1.51806000
C	-4.84343400	-0.57660400	-0.96475800
H	-5.27547100	1.09622500	0.36666000
H	-4.06798800	-2.17131900	-2.17551400
C	3.08200400	1.70223100	-0.29361300
C	2.50356200	2.68856100	0.51585900
C	2.48833300	4.00513700	0.06494300
H	2.08637800	2.41035100	1.47508300
C	3.63252000	3.24320300	-1.90071400
C	3.05444500	4.29086600	-1.17773200
H	2.04341800	4.78960600	0.66897200
H	4.09443300	3.43543700	-2.86690400
H	3.06170600	5.29923100	-1.57760500
C	0.89354000	-1.81484700	2.17762200
C	0.93882500	-2.78982800	3.18037600
C	-0.23568200	-3.11937300	3.85458300
H	1.87440900	-3.26693000	3.44562400
C	-1.38272400	-1.50187900	2.51617800
C	-1.42296900	-2.46864400	3.51797700
H	-0.22010400	-3.87054200	4.63756400
H	-2.27135200	-0.96272700	2.20546300
H	-2.35674100	-2.69815300	4.01838100
C	-1.33527900	-1.77293600	-1.73357400
C	-1.43575000	-2.86840600	-2.64167700
C	-0.30554700	-3.49571400	-3.11119600
H	-2.41343600	-3.20747900	-2.96705100
C	1.01544400	-1.98059500	-1.79918900
C	0.97441700	-3.04239900	-2.68646500
H	-0.39200400	-4.32889500	-3.80295400
H	1.96896700	-1.59855500	-1.44451200
H	1.89054600	-3.50416400	-3.03645400
C	-2.67533600	1.79251000	1.10530700
C	-3.53210200	2.73107900	1.69493900
C	-3.01258100	3.67962900	2.57205800
H	-4.59093900	2.72679200	1.46916100
C	-0.85600800	2.71516800	2.22053200
C	-1.64541200	3.67305300	2.84995600
H	-3.66781700	4.41212600	3.03240700
H	0.21143800	2.66643100	2.40443500
H	-1.19880000	4.38827300	3.53118300
N	-6.17614600	-0.78223900	-1.24758100
N	5.37202500	-2.59809500	0.31540100
H	5.34753400	-3.51326900	0.73880200
N	3.65825800	1.97331900	-1.47718300
N	2.01748300	-0.15649100	0.83424700
N	-0.25451700	-1.18290600	1.87066200
N	-1.34760600	1.80519000	1.36823900
N	-2.17057500	-0.01601900	-0.36510700
N	-0.07103300	-1.35201800	-1.31606400
H	-6.82478000	-0.37523100	-0.58928400
C	0.28490500	2.51896000	-2.34787100
N	0.22401100	1.79734500	-1.44545700
C	0.36153200	3.43017400	-3.47985700
H	0.55665100	4.44328900	-3.12005000
H	1.17510500	3.12423300	-4.14228400
H	-0.58112400	3.41436300	-4.03266200

C	6.54393500	-2.24055900	-0.46613400
H	7.06087600	-1.37263200	-0.04003200
H	7.23207900	-3.08586700	-0.46664700
H	6.27582800	-2.00984200	-1.50375900
C	-6.64182200	-1.97647600	-1.92893200
H	-6.32858000	-2.90294800	-1.42451400
H	-7.73183700	-1.95426300	-1.97427700
H	-6.26157700	-2.00746000	-2.95619000
Zn	-0.14863500	0.23819300	0.16308300

Sum of electronic and thermal Free Energies = -1872.661063 (Hartree/Particle).

Supplementary Table 38. DFT-optimized geometry of $[Zn^{II}(TPY)(\eta^2\text{-}TPY)(COO^{-})]^{+}$ (doublet), computed at the UB3LYP-D3/ LANL2DZ (Zn)/ 6-31G** (HCNO) level in acetonitrile solvent using IEFPCM. The spin density plot is also shown below. (Isovalue = 0.0004)

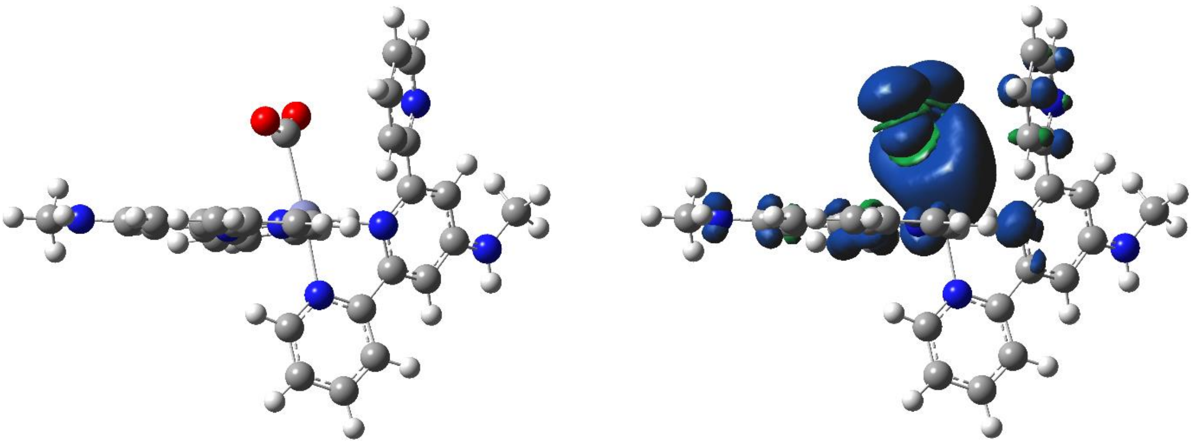

Spin Density Distribution

Atom	x	y	z
C	-3.08289800	0.59712300	0.17954800
C	-2.37521600	-0.91382800	-1.43302400
C	-4.29349800	-0.06858800	0.33374400
C	-3.56041600	-1.62358700	-1.35415400
C	-4.55894800	-1.22584000	-0.43014800
H	-5.01517300	0.32962000	1.03333000
H	-3.71900200	-2.50264500	-1.96764500
C	3.17506100	0.53963500	-0.23824100
C	2.46006200	-1.33453100	0.94849900
C	4.48759600	0.34507000	0.16021400
C	3.74769100	-1.60173000	1.39802100
C	4.80709400	-0.75243700	0.99806800
H	5.27702400	1.01834700	-0.15070200
H	3.94110800	-2.43257400	2.06106300
C	-2.85393500	1.86993000	0.91718000
C	-2.09635000	2.89864800	0.34376700
C	-1.91769400	4.08145600	1.05848900
H	-1.67092400	2.76982200	-0.64312900

C	-3.26287600	3.12471700	2.79483300
C	-2.50296300	4.19672100	2.31737200
H	-1.33225500	4.89299000	0.63827000
H	-3.74627600	3.18779600	3.76774100
H	-2.38665400	5.09415500	2.91558900
C	-1.29371500	-1.32829600	-2.36801300
C	-1.53050700	-2.07295000	-3.52926700
C	-0.45158800	-2.41888400	-4.34004800
H	-2.53799400	-2.35755000	-3.80715400
C	0.98349600	-1.25941300	-2.81758000
C	0.83337100	-2.01090000	-3.97979900
H	-0.61508200	-2.99294400	-5.24621900
H	1.95423300	-0.90700300	-2.48619900
H	1.69767800	-2.26002200	-4.58455500
C	1.27588700	-2.14946800	1.35081600
C	1.38564900	-3.36445800	2.03672800
C	0.22763800	-4.04364100	2.40943200
H	2.35621300	-3.78391200	2.27024500
C	-1.04187500	-2.28794200	1.39817600
C	-1.01418100	-3.49436200	2.09389200
H	0.29769500	-4.98766700	2.93976000
H	-1.98213700	-1.81957700	1.12934600
H	-1.94008200	-3.98470200	2.37190000
C	2.74270300	1.66194800	-1.11988500
C	3.63124200	2.59301000	-1.67008000
C	3.13918900	3.59613100	-2.50190200
H	4.69225800	2.54042300	-1.46095400
C	0.95257800	2.68423500	-2.19357900
C	1.77273700	3.64576400	-2.77783900
H	3.81742300	4.32493300	-2.93338700
H	-0.11719100	2.66923600	-2.37872800
H	1.35080000	4.40389400	-3.42745100
N	6.08296500	-0.96331400	1.39698400
N	-5.70731500	-1.92709900	-0.30814500
H	-5.83035800	-2.73616800	-0.89782400
N	-3.44996300	1.98830000	2.11664600
N	-2.10479300	0.16801100	-0.65413600
N	-0.05240900	-0.92808100	-2.03702700
N	1.42004400	1.72659700	-1.38430200
N	2.18550700	-0.29363100	0.14371000
N	0.06793800	-1.63793700	1.02724400
H	6.78412400	-0.30300700	1.09779100
C	0.40001200	1.59353000	1.81972100
O	-0.26730100	1.09006900	2.72255800
O	1.23155400	2.48525300	1.65987400
Zn	0.12385500	0.29540700	-0.19160300
C	-6.76949300	-1.59914200	0.62813500
H	-7.19865700	-0.61231000	0.41864900
H	-7.55798100	-2.34569600	0.53349100
H	-6.40530900	-1.60695400	1.66203500
C	6.50054000	-2.04592100	2.27123600
H	6.27294000	-3.02405900	1.83148000
H	7.57842200	-1.97738400	2.41738500
H	6.01330900	-1.98373400	3.25181800

Sum of electronic and thermal Free Energies = -1928.519505 (Hartree/Particle).

Supplementary Table 39. DFT-optimized geometry of [Zn^{II}($TPY^{\bullet-}$)(η^2-TPY)(COO^-)] (singlet), computed at the B3LYP-D3/ LANL2DZ (Zn)/ 6-31G** (HCNO) level in acetonitrile solvent using IEFPCM.

Atom	x	y	z
C	3.33509600	0.55831700	-0.22837600
C	2.55548800	-1.17801000	1.07586800
C	4.38163600	-0.22269200	-0.72035400
C	3.56784700	-2.02904600	0.65276900
C	4.51878600	-1.55590200	-0.28483000
H	5.07041300	0.21934100	-1.42672900
H	3.61879500	-3.05284000	1.00804800
C	-3.12873400	0.94074600	0.27746700
C	-2.92660900	-1.17024300	-0.67432400
C	-4.41757400	1.08514800	-0.22320700
C	-4.20560200	-1.10483900	-1.21691100
C	-4.99363800	0.04676000	-0.98711500
H	-4.99355900	1.98451300	-0.03976700
H	-4.58293300	-1.91080200	-1.83090400
C	3.20355500	1.98263000	-0.63790700
C	2.31104400	2.83593500	0.02576300
C	2.20932900	4.16154600	-0.38518900
H	1.72157000	2.45514900	0.84578300
C	3.87059400	3.67959600	-2.03996600
C	2.99958800	4.59890400	-1.44766700
H	1.52113200	4.83704300	0.11482100
H	4.51019600	3.98735200	-2.86576000
H	2.95279100	5.62121600	-1.80833000
C	1.50303400	-1.63987500	2.02274300
C	1.73817400	-2.60238300	3.01129900
C	0.69334900	-2.97837500	3.85328800
H	2.72642300	-3.03092000	3.13100800
C	-0.71151600	-1.42973600	2.68895300
C	-0.55860600	-2.38284000	3.69310100
H	0.85806800	-3.71951500	4.62899700
H	-1.65844300	-0.93478300	2.50021500
H	-1.39655200	-2.64592400	4.32873200
C	-2.02985600	-2.33600700	-0.91737600
C	-2.50701100	-3.59212000	-1.31917800
C	-1.59818900	-4.62481400	-1.53742500
H	-3.56974600	-3.76835700	-1.43734400
C	0.15361500	-3.10854500	-0.94121300
C	-0.23614100	-4.38269800	-1.35063700
H	-1.94997800	-5.60538400	-1.84204900
H	1.20046100	-2.87078900	-0.77036900
H	0.50480900	-5.15895700	-1.50760100
C	-2.45774700	1.98573900	1.09213500
C	-3.04879900	3.21919900	1.40975200
C	-2.35876600	4.12061700	2.21188900
H	-4.03295100	3.47604200	1.03724300
C	-0.56959800	2.54102700	2.32780700
C	-1.09220900	3.77413400	2.69758800
H	-2.80471500	5.07734000	2.46433900
H	0.40988800	2.21842600	2.67105600
H	-0.52984300	4.43910500	3.34346200
N	-6.26009300	0.16186800	-1.47054900

N	5.51945400	-2.36483400	-0.72507700
H	5.50688400	-3.32853600	-0.42891700
N	3.98400600	2.40645900	-1.64934200
N	2.41761900	0.09304300	0.64318300
N	0.29084300	-1.06909700	1.87778700
N	-1.22069400	1.67235700	1.53893700
N	-2.39613600	-0.17704300	0.06527900
N	-0.71440800	-2.11333400	-0.72521400
H	-6.71585500	1.05541600	-1.36674700
C	-0.08235500	1.36262300	-1.69171500
O	0.80352100	1.12749200	-2.55888400
O	-1.02493800	2.20201300	-1.77140000
Zn	-0.17155400	0.20317100	0.09314800
C	6.47010900	-1.98654000	-1.75445800
H	7.07612300	-1.12836300	-1.44081500
H	7.13943600	-2.82775100	-1.93731700
H	5.97088800	-1.72676300	-2.69706600
C	-6.88627400	-0.81165100	-2.34649700
H	-6.96395100	-1.79009300	-1.85851200
H	-7.89433200	-0.46933500	-2.58243400
H	-6.33331600	-0.93856700	-3.28669400

Sum of electronic and thermal Free Energies = -1928.639029 (Hartree/Particle).

Supplementary Table 40. DFT-optimized geometry of $[Zn^{II}(TPY^{\bullet-})(\eta^2\text{-}TPY)(COOH)]^+$ (singlet), computed at the B3LYP-D3/ LANL2DZ (Zn)/ 6-31G** (HCNO) level in acetonitrile solvent using IEFPCM.

Atom	x	y	z
C	-3.19910800	0.70372000	0.29709300
C	-2.66679800	-1.23911500	-0.83611200
C	-4.48004500	0.23524000	0.57867300
C	-3.92043100	-1.78209600	-0.60457700
C	-4.87338600	-1.04455300	0.13765600
H	-5.15066700	0.87457700	1.13535000
H	-4.16656600	-2.77674400	-0.95869000
C	2.91599000	0.78759300	-0.95102000
C	3.09080700	-0.72680800	0.80145100
C	4.21453900	1.23178800	-0.76452700
C	4.40260000	-0.34634300	1.06198900
C	4.99953300	0.66365000	0.27019400
H	4.63127900	2.02055900	-1.38017300
H	4.95537000	-0.79806500	1.87361700
C	-2.83535800	2.08560000	0.72488700
C	-1.84739500	2.81855500	0.05784000
C	-1.55232800	4.10889700	0.49096500
H	-1.31743500	2.38055500	-0.77375000
C	-3.23636200	3.83194700	2.16778500
C	-2.25444300	4.63090800	1.57499300
H	-0.78436400	4.69069900	-0.00902700
H	-3.81192900	4.20812500	3.01154200
H	-2.05705100	5.62851100	1.95277500
C	-1.64369400	-2.00685100	-1.59790500

C	-1.97493300	-2.95998800	-2.56906400
C	-0.95312800	-3.64022400	-3.22690700
H	-3.01144800	-3.14834500	-2.82113600
C	0.62429800	-2.38122000	-1.94404500
C	0.37511300	-3.35285100	-2.90786300
H	-1.19176400	-4.37883800	-3.98525200
H	1.63103400	-2.09751500	-1.65528400
H	1.19844000	-3.86002300	-3.39753800
C	2.38497600	-1.78203400	1.58700700
C	3.04752600	-2.67786000	2.43495000
C	2.30375400	-3.63744700	3.11978200
H	4.12428100	-2.64366200	2.54831700
C	0.34037300	-2.76444500	2.06920800
C	0.92169000	-3.68684100	2.93865800
H	2.80055800	-4.34125800	3.77969300
H	-0.73035600	-2.76102900	1.88287200
H	0.30868600	-4.42055200	3.44988900
C	2.02315200	1.30787200	-2.02517500
C	2.50223200	1.96183000	-3.16586800
C	1.60016200	2.35077100	-4.15433600
H	3.56287500	2.14037900	-3.29490200
C	-0.15270000	1.40809400	-2.82879200
C	0.24498700	2.06272000	-3.99214000
H	1.95538700	2.85569200	-5.04671600
H	-1.19153200	1.14573400	-2.64885600
H	-0.48658900	2.33081700	-4.74570600
N	6.26804700	1.08548600	0.48189300
N	-6.09932200	-1.55760000	0.39814100
H	-6.31402000	-2.47357100	0.03508400
N	-3.53218100	2.59496400	1.75828300
N	-2.27867600	-0.02081700	-0.38174300
N	-0.35941800	-1.72748000	-1.31047800
N	0.70698100	1.05534700	-1.86428400
N	2.35876000	-0.16715100	-0.17864300
N	1.04900700	-1.83985200	1.41381000
H	6.62372300	1.82059300	-0.10986700
C	0.11225900	1.17196100	1.76006900
O	-0.54423300	1.05483300	2.79070500
O	1.04354300	2.19827900	1.72622500
H	0.96444500	2.66371400	2.58460800
C	-7.12852400	-0.86585900	1.15425100
H	-7.42740600	0.06950700	0.66576600
H	-8.00305400	-1.51289800	1.22390800
H	-6.78907000	-0.63147100	2.17012800
C	7.13501400	0.58215100	1.53325400
H	7.31802800	-0.49304800	1.42099600
H	8.09129800	1.10149700	1.47246700
H	6.70718900	0.75962000	2.52734200
Zn	0.12171900	-0.08771000	0.05280000

Sum of electronic and thermal Free Energies = -1929.118793 (Hartree/Particle).

Supplementary Table 41. DFT-optimized geometry of $[Zn^{II}(TPY)(\eta^2\text{-}TPY)(CO)]^{2+}$ (singlet), computed at the B3LYP-D3/ LANL2DZ (Zn)/ 6-31G** (HCNO) level in acetonitrile solvent using IEFPCM.

Atom	x	y	z
C	3.00443500	0.58600600	-0.19202800
C	2.33861700	-0.93001200	1.44112500
C	4.24410500	-0.02310100	-0.31896400
C	3.55478000	-1.58388100	1.38640000
C	4.55174900	-1.15329500	0.47319600
H	4.95726100	0.39179400	-1.01792400
H	3.74562500	-2.44558700	2.01470400
C	-3.14843400	0.60421300	0.22085100
C	-2.42648300	-1.19833200	-1.07285800
C	-4.47401300	0.34756900	-0.07850300
C	-3.72782300	-1.52536500	-1.42680800
C	-4.79868400	-0.74738800	-0.92107400
H	-5.27055500	0.96607800	0.31586500
H	-3.92912200	-2.35667000	-2.08616900
C	2.71974900	1.82292600	-0.97376600
C	2.15858000	2.94379500	-0.34634900
C	1.93269300	4.09288300	-1.10226700
H	1.91571900	2.90396400	0.70842900
C	2.84575800	2.92137000	-2.98544200
C	2.27627300	4.08169500	-2.45358600
H	1.50001300	4.97616100	-0.64374100
H	3.12944800	2.88162300	-4.03460500
H	2.11427800	4.94880900	-3.08441600
C	1.26328300	-1.37569500	2.37132400
C	1.51090100	-2.12393000	3.52663700
C	0.43736700	-2.49654800	4.33336400
H	2.52230300	-2.39177400	3.80572400
C	-1.02220300	-1.35665100	2.82114700
C	-0.85611500	-2.11318900	3.97692200
H	0.61233900	-3.07350400	5.23531100
H	-2.00036900	-1.02068300	2.49266500
H	-1.71466300	-2.38416200	4.57999500
C	-1.23260300	-1.94233800	-1.57054600
C	-1.32592200	-3.08383600	-2.37258100
C	-0.15908000	-3.70644800	-2.81119600
H	-2.28977600	-3.48964700	-2.65192500
C	1.08888400	-2.04015600	-1.63578000
C	1.07506100	-3.17522700	-2.44223800
H	-0.21747700	-4.59351400	-3.43298100
H	2.02431300	-1.59139700	-1.32341100
H	2.00731600	-3.62420600	-2.76440800
C	-2.70191100	1.72908100	1.09221700
C	-3.58209900	2.63191900	1.69610400
C	-3.07073200	3.64540200	2.50395500
H	-4.65073100	2.55135100	1.54377900
C	-0.87886900	2.79938100	2.06702100
C	-1.69309500	3.73421500	2.69965100
H	-3.74313400	4.35351600	2.97647600
H	0.19808100	2.81410100	2.19327600
H	-1.25584300	4.50358100	3.32498700
N	-6.08481000	-1.02055700	-1.22442700
N	5.73210200	-1.79776300	0.38577000
H	5.88421600	-2.58790700	0.99464200
N	3.07351100	1.81159200	-2.27130100
N	2.03632800	0.13020200	0.63897900
N	0.01047800	-0.99922600	2.04587800
N	-1.36648500	1.82960100	1.28083200

N	-2.14700000	-0.16185200	-0.26164400
N	-0.03001700	-1.43944600	-1.20696800
H	-6.79741300	-0.42048900	-0.83682100
C	-0.30342500	1.64443000	-1.96399900
O	-0.51301300	2.10911600	-2.97684500
Zn	-0.12916900	0.25863000	0.30156800
C	6.80083400	-1.43007200	-0.52983900
H	7.16960500	-0.41807300	-0.32726900
H	7.62456900	-2.13130500	-0.39904600
H	6.46474200	-1.47778100	-1.57174600
C	-6.51425300	-2.11226700	-2.08395300
H	-6.20285800	-3.08278400	-1.68111900
H	-7.60210000	-2.09697300	-2.14414100
H	-6.10930100	-2.00713300	-3.09706900

Sum of electronic and thermal Free Energies = -1853.123533 (Hartree/Particle).

Supplementary Table 42. DFT-optimized geometry of $[Zn^{II}(TPY)_2]^{2+}\cdot Pt$ (singlet), computed at the B3LYP-D3/ LANL2DZ (Zn, Pt)/ 6-31G** (HCN) level in acetonitrile solvent using IEFPCM.

Atom	x	y	z
C	-3.78854900	1.88799200	-0.31568900
C	-2.73078600	2.07302800	-1.27615900
C	-1.65812100	1.22991800	-1.29800500
C	-2.52834400	-0.00516000	0.58631000
C	-3.66823400	0.86807300	0.62057300
H	-2.78169600	2.91123400	-1.96189600
H	-4.40559900	0.73820600	1.39967100
C	-2.06305800	-0.67875400	1.84570600
C	-2.95695200	-1.15815800	2.81062700
C	-2.45343500	-1.78074800	3.94839000
H	-4.02586300	-1.06328400	2.66161600
C	-0.24778000	-1.40857300	3.10474500
C	-1.07089200	-1.91138700	4.10584300
H	-3.13235700	-2.16334000	4.70360900
H	0.83364600	-1.48315200	3.16741700
H	-0.64127400	-2.39103400	4.97746300
C	-0.50456500	1.35771300	-2.21920700
C	-0.57913900	2.03030200	-3.44409900
C	1.73407600	0.85658200	-2.57431300
C	0.56088100	2.11203100	-4.24009300
H	-1.51500000	2.46424300	-3.77579000
C	1.74387000	1.51551100	-3.80158000
H	2.62785100	0.37813300	-2.18674300
H	0.52262600	2.62766600	-5.19404800
H	2.65108900	1.55269400	-4.39341800
N	0.64249700	0.77636700	-1.80292700
N	-1.50991000	0.21186400	-0.35889500
N	-0.72910300	-0.80603700	2.00544500
N	-4.85224000	2.73885900	-0.35390000
H	-4.76449000	3.56095600	-0.93204000
C	-5.89346400	2.74691500	0.65684000

H	-6.61599600	3.52532100	0.41095600
H	-6.41875100	1.78552500	0.67984600
H	-5.49185800	2.94307500	1.66077100
C	5.35174500	0.78210000	0.37540600
C	4.41720500	1.78179800	0.75204600
C	3.07384300	1.46045900	0.84091000
C	3.48909600	-0.75014200	0.22219300
C	4.85240700	-0.51642300	0.10458900
H	4.77030700	2.78569900	0.95334100
H	5.52417100	-1.30443300	-0.20412800
C	2.85525100	-2.07395000	-0.04953500
C	3.58498600	-3.24847800	-0.25157100
C	2.90215500	-4.44023000	-0.49093200
H	4.66694200	-3.24261200	-0.21203400
C	0.84910100	-3.22737500	-0.30134800
C	1.50842700	-4.43545800	-0.51670400
H	3.45538800	-5.36008200	-0.64905300
H	-0.23635100	-3.15477400	-0.30694400
H	0.94067400	-5.34142100	-0.69474000
C	2.01099900	2.43765700	1.22066400
C	2.28363800	3.73586000	1.66320600
C	-0.27414100	2.79159400	1.45542500
C	1.22276200	4.57384300	2.00278900
H	3.30269500	4.09056300	1.75339500
C	-0.08373400	4.09821100	1.89958500
H	-1.27083600	2.37143700	1.36077100
H	1.41801300	5.58349400	2.34869400
H	-0.93564400	4.71670300	2.15734300
N	0.74169700	1.98578700	1.12293000
N	2.62486000	0.21640300	0.57678200
N	1.50312300	-2.07960100	-0.07848600
N	6.66602600	1.07607200	0.27751500
H	6.95107100	2.02454700	0.46996900
C	7.69444000	0.12658100	-0.11574900
H	8.65871200	0.63377100	-0.09672200
H	7.73673900	-0.72356700	0.57462700
H	7.52253900	-0.25424800	-1.12935200
Zn	0.52379800	-0.12759500	0.30268900
Pt	-2.41630100	-1.54114700	-0.90086800

Sum of electronic and thermal Free Energies = -1858.950518 (Hartree/Particle).

Supplementary Table 43. DFT-optimized geometry of $[Zn^{II}(TPY^{\bullet -})(TPY)]^{+}\cdot Pt$ (doublet), computed at the UB3LYP-D3/ LANL2DZ (Zn, Pt)/ 6-31G** (HCN) level in acetonitrile solvent using IEFPCM. The spin density plot is also shown below. (Isovalue = 0.004)

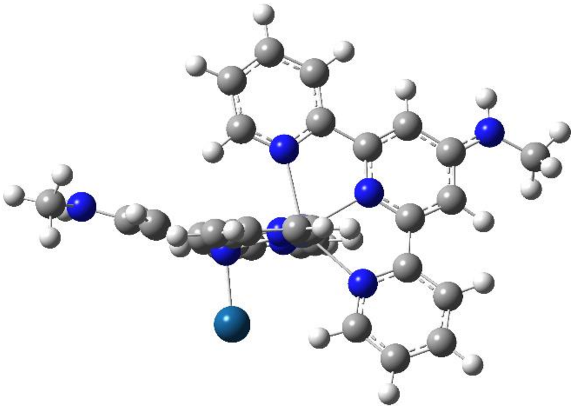 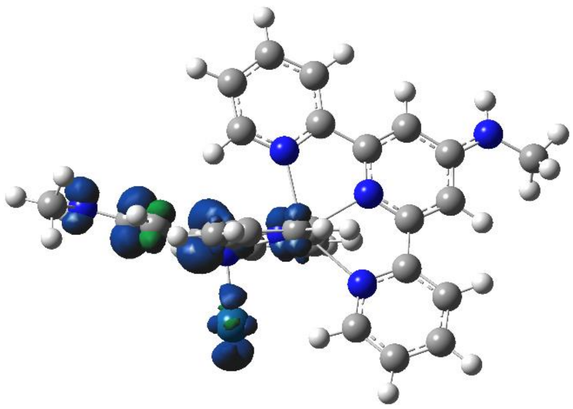

Spin Density Distribution

Atom	x	y	z
C	-4.01608700	1.71692000	-0.16956700
C	-3.06185200	1.82904500	-1.22168200
C	-1.92589400	1.05272700	-1.22078600
C	-2.50798500	0.16422100	0.93205300
C	-3.68506600	0.90650000	0.93682300
H	-3.21261800	2.57961200	-1.99034700
H	-4.30437300	0.92370300	1.82441000
C	-1.95982100	-0.51848600	2.10185300
C	-2.73183800	-0.88325400	3.22256000
C	-2.12939600	-1.54015700	4.28529800
H	-3.79487500	-0.67386900	3.24114300
C	-0.05716500	-1.44858300	3.09528400
C	-0.75941800	-1.83591900	4.23010300
H	-2.72027200	-1.83134500	5.14814400
H	1.00485500	-1.65295900	2.99073700
H	-0.25565400	-2.35426900	5.03752600
C	-0.82336700	1.19783600	-2.18467000
C	-0.97302900	1.80991000	-3.44051200
C	1.43490300	0.80690200	-2.61867000
C	0.12570900	1.91532300	-4.28447700
H	-1.94211800	2.17894200	-3.75491800
C	1.36238900	1.40607800	-3.87168000
H	2.36799900	0.39684200	-2.24329900
H	0.01972400	2.38299000	-5.25822100
H	2.24179500	1.46629200	-4.50229200
N	0.38031600	0.69893200	-1.79891100
N	-1.70244200	0.09521000	-0.21754800
N	-0.62774600	-0.81021800	2.06316800
N	-5.15306600	2.48261100	-0.20042600
H	-5.39409000	2.89932100	-1.08668100
C	-6.21782100	2.35733400	0.77644100
H	-7.04180200	3.00840400	0.48224300
H	-6.59454100	1.32744200	0.85273500
H	-5.87886200	2.66986000	1.77148300
C	5.22934000	1.09365500	0.24653600
C	4.23917400	2.05686000	0.56457700
C	2.91922600	1.66235500	0.70989000
C	3.46485600	-0.55734400	0.27037800
C	4.80832100	-0.24982500	0.10168400
H	4.53086000	3.09386900	0.67557100
H	5.52068000	-1.01951700	-0.15866300

C	2.93109400	-1.94228900	0.13011200
C	3.74237100	-3.08065200	0.09760500
C	3.14463300	-4.33374200	-0.03170700
H	4.81845600	-2.99879000	0.18708400
C	1.01399500	-3.24288600	-0.07515900
C	1.75620800	-4.42155700	-0.12231300
H	3.75907800	-5.22789900	-0.05586500
H	-0.07493100	-3.22480100	-0.15659700
H	1.25417400	-5.37722100	-0.22296300
C	1.81816700	2.61364300	1.04079300
C	2.04705100	3.95232300	1.37935700
C	-0.46934700	2.89515100	1.29980400
C	0.96189100	4.77335400	1.67846300
H	3.05217100	4.35248800	1.42103200
C	-0.32450700	4.23905400	1.63914900
H	-1.44936900	2.43322000	1.26059300
H	1.12332300	5.81326400	1.94295000
H	-1.19735000	4.84014400	1.86702900
N	0.56923400	2.10188400	1.00737700
N	2.53783500	0.37621000	0.55629900
N	1.58754400	-2.03869400	0.04853900
N	6.51990300	1.46111700	0.08685500
H	6.74521000	2.43969400	0.18307400
C	7.59629800	0.54847000	-0.26163900
H	8.52535600	1.11513800	-0.32018800
H	7.71602200	-0.23484500	0.49572900
H	7.41964400	0.07061400	-1.23266600
Zn	0.43043000	-0.12365600	0.27280400
Pt	-2.04680300	-1.74248500	-1.06174100

Sum of electronic and thermal Free Energies = -1859.066437 (Hartree/Particle).

Supplementary Table 44. DFT-optimized geometry of **TS2** (doublet), computed at the UB3LYP-D3/ LANL2DZ (Zn, Pt)/ 6-31G** (HCN) level in acetonitrile solvent using IEFPCM.

Atom	x	y	z
C	-3.56311300	0.16811300	0.49832400
C	-2.27032200	1.97643900	1.13703400
C	-4.56831800	0.99547900	0.01123400
C	-3.22389900	2.87429900	0.68909800
C	-4.40329200	2.39685800	0.06673000
H	-5.46681800	0.54246700	-0.38457500
H	-3.06526000	3.94207600	0.78097200
C	2.74695400	-0.45936700	0.79529700
C	2.08738900	1.24699600	-0.75513200
C	3.96242800	0.16389600	0.99314300
C	3.29994600	1.89735100	-0.56953600
C	4.29552600	1.33314200	0.25803400
H	4.64095900	-0.19176500	1.76170300
H	3.45713900	2.87030300	-1.01647800
C	-3.79745000	-1.29972900	0.55551200
C	-3.27506200	-2.05134800	1.61550700
C	-3.51728800	-3.42243500	1.65188700
H	-2.70005400	-1.55508300	2.38838700

C	-4.78896000	-3.15679400	-0.36203600
C	-4.28274900	-3.99446000	0.63564600
H	-3.12226700	-4.03128400	2.45929900
H	-5.40207300	-3.56697700	-1.16171900
H	-4.49602600	-5.05772800	0.61766900
C	-1.03009500	2.44449200	1.81244100
C	-0.95193000	3.68313100	2.45984500
C	0.23225900	4.04289800	3.09864200
H	-1.80860000	4.34490900	2.48756700
C	1.14531200	1.93520000	2.43333900
C	1.30458800	3.15248800	3.08982900
H	0.30885900	4.99919300	3.60562200
H	1.95172600	1.21371400	2.39497400
H	2.24308700	3.38630700	3.57908600
C	0.95431000	1.80271700	-1.49064900
C	1.07199800	2.85854200	-2.41631800
C	-0.05050800	3.29442000	-3.10522400
H	2.03883900	3.30873700	-2.60821800
C	-1.33075300	1.64117500	-1.94549700
C	-1.28555700	2.67113500	-2.87725000
H	0.03382700	4.10356200	-3.82405000
H	-2.26203000	1.13071600	-1.72079600
H	-2.18358900	2.97432200	-3.40285200
C	2.22263500	-1.53137400	1.65111500
C	3.05099100	-2.40219900	2.38007000
C	2.48041700	-3.39756100	3.16124200
H	4.12814200	-2.31034300	2.30614900
C	0.32881000	-2.62588900	2.45886500
C	1.08640000	-3.52051700	3.20495700
H	3.11240700	-4.08018500	3.72043300
H	-0.75325400	-2.68338700	2.44889500
H	0.60018500	-4.29037500	3.79257200
N	5.53366900	1.91040900	0.41498800
N	-5.32369400	3.25540800	-0.42410500
H	-5.14830000	4.24348400	-0.32145700
N	-4.55927800	-1.83896700	-0.41251300
N	-2.39255200	0.63029200	0.99802300
N	0.01212900	1.58899300	1.80595000
N	0.86714600	-1.65461600	1.70494300
N	1.84803400	-0.01826300	-0.19022300
N	-0.25665400	1.21868500	-1.26345800
H	6.06746700	1.59266200	1.21117000
C	-1.55695400	-2.62455500	-1.48331400
N	-1.11362700	-1.79432600	-0.81126700
C	-2.04573600	-3.66927300	-2.36724600
H	-2.27286400	-4.56620200	-1.78651900
H	-2.95443800	-3.32979600	-2.87000100
H	-1.27842100	-3.89888300	-3.11238700
C	-6.56168700	2.84604100	-1.06583700
H	-7.20281000	2.28084100	-0.37895700
H	-7.09955200	3.73885600	-1.38441300
H	-6.36556300	2.22525700	-1.94758200
C	5.83833500	3.24931000	-0.05741600
H	5.17649600	4.01426200	0.37533300
H	6.86823500	3.48873500	0.21121500
H	5.75273900	3.30267600	-1.14820700
Zn	-0.27067800	-0.21076300	0.46626200
Pt	2.06907900	-1.41042600	-1.67680900

Sum of electronic and thermal Free Energies = -1991.814933 (Hartree/Particle).

Supplementary Table 45. DFT-optimized geometry of $[Zn^{II}(TPY^{\bullet-})(\eta^2\text{-}TPY)(CH_3CN)]^+\cdot Pt$ (doublet), computed at the UB3LYP-D3/ LANL2DZ (Zn, Pt)/ 6-31G** (HCN) level in acetonitrile solvent using IEFPCM. The spin density plot is also shown below. (Isovalue = 0.004)

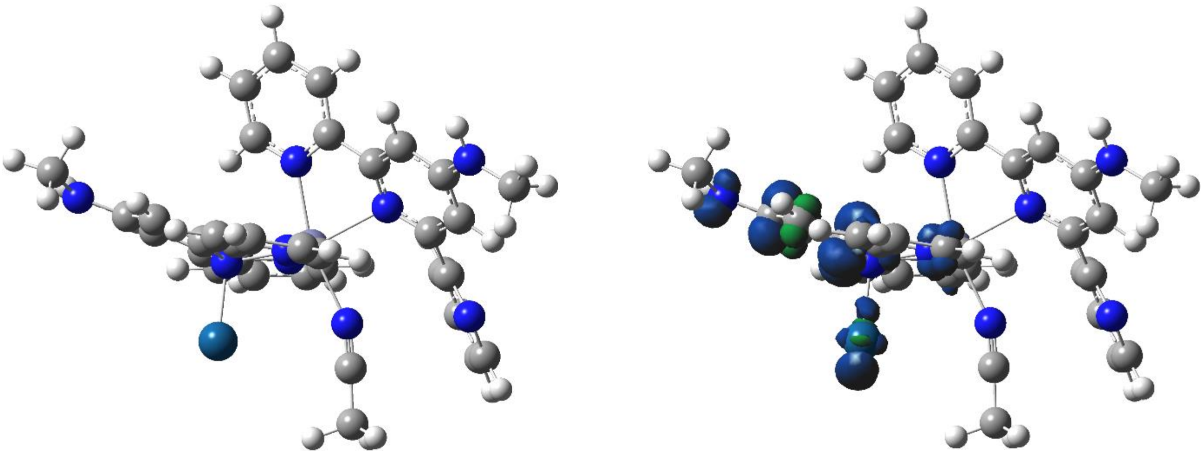

Spin Density Distribution

Atom	x	y	z
C	3.60476800	-0.00892100	0.45776000
C	2.44994200	-1.91471300	1.08006500
C	4.66954400	-0.75366900	-0.03546800
C	3.46726500	-2.73464800	0.62257600
C	4.61285500	-2.16382900	0.01478000
H	5.52799100	-0.23179300	-0.43562800
H	3.38619000	-3.81251400	0.69734500
C	-2.71640900	0.27674600	0.84704500
C	-1.99257100	-1.30438900	-0.80233600
C	-3.89999400	-0.41286900	1.01632400
C	-3.17225800	-2.02036200	-0.64835400
C	-4.18575900	-1.55188600	0.21661500
H	-4.58717600	-0.13210800	1.80787800
H	-3.28765800	-2.97221500	-1.15012800
C	3.70424800	1.47491800	0.49335000
C	3.18621200	2.18393900	1.58481900
C	3.26211100	3.57387800	1.57899000
H	2.73779100	1.64032200	2.40826300
C	4.37940500	3.41012600	-0.53639300
C	3.85497800	4.20624100	0.48574900
H	2.86552700	4.15262700	2.40748100
H	4.86855500	3.87081700	-1.39209200
H	3.92678000	5.28699900	0.42827900
C	1.23936400	-2.47819200	1.73694500
C	1.23659500	-3.73588900	2.35141200
C	0.07320700	-4.18939000	2.96839900
H	2.13418600	-4.34135500	2.37000000
C	-0.97082200	-2.12919100	2.34886400
C	-1.05563500	-3.37174700	2.97076200
H	0.05536200	-5.16174500	3.44963100
H	-1.82239000	-1.46080900	2.32151400

H	-1.98119400	-3.68003600	3.44309000
C	-0.83991400	-1.76728800	-1.57340700
C	-0.92089600	-2.76605300	-2.56342200
C	0.21691800	-3.11998700	-3.27432800
H	-1.87191100	-3.23533000	-2.78603800
C	1.43871000	-1.49937400	-2.00990000
C	1.42959800	-2.47171200	-3.00287300
H	0.16100500	-3.88479100	-4.04273200
H	2.35172100	-0.97230100	-1.75158300
H	2.33825200	-2.71177800	-3.54262800
C	-2.23592600	1.32084900	1.76247200
C	-3.09931600	2.11807900	2.53312900
C	-2.56823600	3.08840300	3.37207900
H	-4.17197900	1.99049500	2.44725000
C	-0.38674000	2.43716700	2.63919900
C	-1.18007800	3.25942600	3.43069000
H	-3.22686200	3.71522700	3.96486200
H	0.69239300	2.53447300	2.63760400
H	-0.72501700	4.01203600	4.06387200
N	-5.39377100	-2.19395800	0.34679300
N	5.60059500	-2.94680500	-0.47266100
H	5.50533900	-3.94550200	-0.36788100
N	4.31894300	2.07280900	-0.54099600
N	2.47768500	-0.56215600	0.96227600
N	0.14314800	-1.69204200	1.74403000
N	-0.88731500	1.49110300	1.82994500
N	-1.80555300	-0.06484000	-0.16631000
N	0.34903300	-1.15587800	-1.30833600
H	-5.93756700	-1.94749000	1.16115500
C	1.05119800	2.80113600	-1.34421300
N	1.05711800	1.88739200	-0.63601300
C	0.98294400	3.93208700	-2.25149100
H	1.10642000	4.86470000	-1.69556400
H	1.76919000	3.85441800	-3.00661800
H	0.00100100	3.91842200	-2.73654500
C	6.80989200	-2.44000900	-1.09931800
H	7.39895200	-1.83058700	-0.40353600
H	7.41792600	-3.28713800	-1.41650200
H	6.57547600	-1.83172700	-1.98041200
C	-5.64616900	-3.51069900	-0.21070700
H	-4.95017800	-4.27368400	0.16832500
H	-6.66321900	-3.81001500	0.04636800
H	-5.56625400	-3.48915200	-1.30302700
Zn	0.30544300	0.17396600	0.49073800
Pt	-2.14109400	1.38043700	-1.58723200

Sum of electronic and thermal Free Energies = -1991.816915 (Hartree/Particle).

Supplementary Table 46. DFT-optimized geometry of $[Zn^{II}(TPY)(\eta^2\text{-}TPY)(COO^{-})]^{+}\cdot Pt$ (doublet), computed at the UB3LYP-D3/ LANL2DZ (Zn, Pt)/ 6-31G** (HCNO) level in acetonitrile solvent using IEFPCM. The spin density plot is also shown below. (Isovalue = 0.004)

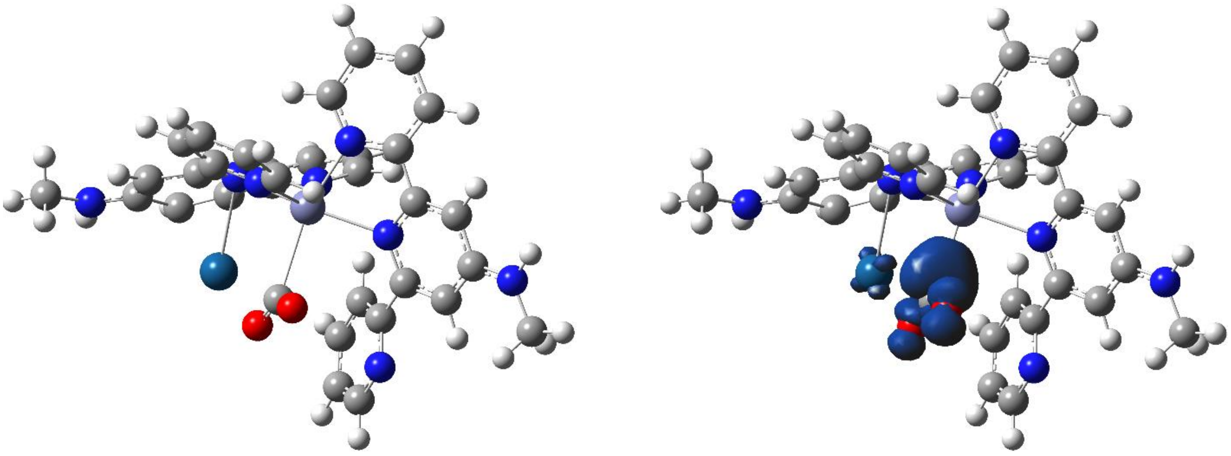

Spin Density Distribution

Atom	x	y	z
C	-3.14667600	-1.22161500	-0.30735600
C	-3.44456900	0.96356200	0.41451200
C	-4.46621100	-1.30083600	-0.73555600
C	-4.76956900	0.96451400	0.01713000
C	-5.31772900	-0.18313100	-0.60596900
H	-4.81655300	-2.23520700	-1.15152600
H	-5.38816900	1.84244100	0.16003800
C	2.22074500	0.02801200	1.03963300
C	2.31238100	1.80559700	-0.50210900
C	3.58978100	-0.36099000	0.74855700
C	3.67273000	1.59007200	-0.77728300
C	4.34080900	0.52882600	-0.13951500
H	4.17285900	-0.91684900	1.48001800
H	4.17985300	2.20004300	-1.51165000
C	-2.30972800	-2.45574900	-0.35866300
C	-1.37384800	-2.72889900	0.64454800
C	-0.63611700	-3.90835200	0.58148400
H	-1.22830500	-2.02424600	1.44878100
C	-1.83785900	-4.43632300	-1.42093100
C	-0.86576500	-4.78277300	-0.47752900
H	0.10043900	-4.13385500	1.34615100
H	-2.05040000	-5.10177500	-2.25550300
H	-0.31636200	-5.71316600	-0.57514800
C	-2.85448900	2.14828600	1.10050800
C	-3.61750100	3.11357300	1.76910800
C	-2.96622400	4.17445600	2.39457900
H	-4.69572000	3.02890500	1.82181300
C	-0.88300900	3.24651900	1.67843100
C	-1.57258900	4.25002200	2.35084500
H	-3.54226900	4.92950300	2.91942500
H	0.20030600	3.23134500	1.60735600
H	-1.03330700	5.05886900	2.82996700
C	1.47421600	2.73796300	-1.30651600
C	1.97441000	3.87797300	-1.94240200
C	1.10222200	4.67249500	-2.68519900
H	3.01939800	4.14706800	-1.84033500
C	-0.66124700	3.15770800	-2.10809400
C	-0.24158200	4.30806500	-2.77533800
H	1.46675600	5.56591900	-3.18169100
H	-1.69770700	2.83415700	-2.13961400

H	-0.95113500	4.89805800	-3.34431100
C	1.48164400	-0.36291200	2.27979400
C	2.07988800	-0.89280600	3.42566500
C	1.27716700	-1.18023200	4.52844500
H	3.14932400	-1.06239800	3.45951800
C	-0.61153800	-0.39926300	3.28369300
C	-0.09459200	-0.92376500	4.46626600
H	1.72012500	-1.58902200	5.43103600
H	-1.67171000	-0.19100600	3.16673000
H	-0.74753600	-1.12543200	5.30761600
N	5.65164000	0.28878900	-0.31814900
N	-6.60129700	-0.19598100	-1.02884100
H	-7.15943300	0.62916200	-0.86971700
N	-2.55510600	-3.30973200	-1.37081300
N	-2.60826500	-0.09438700	0.22069400
N	-1.51304700	2.23128900	1.07442700
N	0.15409800	-0.13425000	2.21865400
N	1.62596100	1.12896000	0.41085300
N	0.17079100	2.39170800	-1.39347700
H	6.03524200	-0.54271800	0.10713200
C	-0.12914800	-1.06066400	-1.88003700
O	-0.87637500	-0.63358500	-2.74309600
O	0.63816300	-2.06512400	-1.79431200
C	-7.24242900	-1.33908900	-1.65682400
H	-7.27181500	-2.20410700	-0.98347900
H	-8.26592300	-1.06551500	-1.91279200
H	-6.72117300	-1.63185300	-2.57529100
C	6.51202300	1.04381300	-1.21535600
H	6.51937600	2.10488900	-0.94527200
H	7.52739200	0.65702900	-1.13148500
H	6.18465500	0.95081200	-2.25788500
Zn	-0.45120000	0.62968500	0.09263600
Pt	2.18559600	-1.49270600	-0.36608000

Sum of electronic and thermal Free Energies = -2047.696224 (Hartree/Particle).

Supplementary Table 47. DFT-optimized geometry of [Zn^{II}($TPY^{\bullet -}$)(η^2-TPY)(COO^-)]·Pt (singlet), computed at the B3LYP-D3/ LANL2DZ (Zn, Pt)/ 6-31G** (HCNO) level in acetonitrile solvent using IEFPCM.

Atom	x	y	z
C	-3.71705500	-0.81407000	-0.17904900
C	-3.37493800	1.35297300	0.51703600
C	-4.93524200	-0.47504000	-0.76969100
C	-4.56615900	1.78917800	-0.04909900
C	-5.38687700	0.85856500	-0.72762900
H	-5.50929100	-1.25384700	-1.25139500
H	-4.85717600	2.83264600	0.00909300
C	2.31790400	-0.41999300	0.92971300
C	2.78511300	1.55488400	-0.25090500
C	3.63540600	-0.97410100	0.62872100
C	4.10395300	1.15395100	-0.53683700
C	4.55999500	-0.09114200	-0.06888100

H	4.08228200	-1.71961500	1.28430800
H	4.74088200	1.78467400	-1.14137100
C	-3.26317500	-2.23689700	-0.21170300
C	-1.98822000	-2.60881200	0.23159600
C	-1.61907200	-3.95077500	0.19035600
H	-1.29503500	-1.85843600	0.57153300
C	-3.77791300	-4.42500200	-0.72242000
C	-2.53085500	-4.88776900	-0.29229700
H	-0.62760300	-4.24857800	0.51932700
H	-4.51584500	-5.12384000	-1.11299500
H	-2.28955900	-5.94443000	-0.34373500
C	-2.50918600	2.30847600	1.26280000
C	-3.04912700	3.32422200	2.06237600
C	-2.19253900	4.16475300	2.76840000
H	-4.12414300	3.43170200	2.14652700
C	-0.35227700	2.93960100	1.85567800
C	-0.81429100	3.96781600	2.67018700
H	-2.59593900	4.95209800	3.39714200
H	0.70516700	2.72930600	1.73396400
H	-0.11176200	4.59108600	3.21163400
C	2.19340400	2.78182700	-0.85821400
C	2.94700800	3.91597000	-1.18243000
C	2.30151200	5.01758700	-1.74232000
H	4.01139600	3.94251800	-0.97809800
C	0.25026200	3.78932900	-1.60856700
C	0.92531800	4.95793200	-1.96409200
H	2.86421600	5.91096900	-1.99416000
H	-0.82348400	3.69672300	-1.75217100
H	0.38491200	5.79277700	-2.39650800
C	1.45544000	-0.89514600	2.05525000
C	1.90976800	-1.64614400	3.14371100
C	0.99999700	-2.00690000	4.13613600
H	2.95345200	-1.92845100	3.21715500
C	-0.70532700	-0.86313700	2.90524300
C	-0.33421300	-1.60747000	4.02341900
H	1.33094200	-2.58542000	4.99326000
H	-1.72864500	-0.53844500	2.73860100
H	-1.06832600	-1.86547400	4.77851700
N	5.83232900	-0.50484800	-0.24418800
N	-6.55911200	1.24780500	-1.29612500
H	-6.77486700	2.23272900	-1.29554200
N	-4.14435700	-3.14061200	-0.68725200
N	-2.92700100	0.08162900	0.44893800
N	-1.17648700	2.13616000	1.16464900
N	0.16340400	-0.51882700	1.94768600
N	1.93840100	0.86402800	0.50175600
N	0.86155200	2.72777900	-1.07255200
H	6.05249800	-1.45083400	0.03010100
C	-0.45118900	-0.69634900	-1.76781700
O	-1.31207900	-0.51870400	-2.64741500
O	0.34902700	-1.76732000	-1.86126800
C	-7.40317400	0.37173400	-2.08799700
H	-7.77953700	-0.46628400	-1.48964500
H	-8.25836500	0.94405400	-2.44874000
H	-6.86823100	-0.03896800	-2.95415100
C	6.84190900	0.23271200	-0.98450300
H	6.99592800	1.22582700	-0.54889000
H	7.78266800	-0.31597800	-0.93441500
H	6.56321800	0.35616900	-2.03854400
Zn	-0.22620600	0.61372200	-0.08901200

Pt	2.07426700	-1.67636500	-0.67703100

Sum of electronic and thermal Free Energies = -2047.839992 (Hartree/Particle).

Supplementary Table 48. DFT-optimized geometry of $[Zn^{II}(TPY^{\bullet -})(\eta^{2}\text{-}TPY)(COOH)]^{+}\cdot Pt$ (singlet), computed at the B3LYP-D3/ LANL2DZ (Zn, Pt)/ 6-31G** (HCNO) level in acetonitrile solvent using IEFPCM.

Atom	x	y	z
C	-3.07369400	-1.20242400	-0.16557600
C	-3.44194400	0.91929000	0.65770400
C	-4.37542100	-1.29395400	-0.64909800
C	-4.75962200	0.91765300	0.22288000
C	-5.26683500	-0.21726300	-0.45444300
H	-4.67686200	-2.19130700	-1.17233000
H	-5.38921000	1.79022500	0.35749500
C	2.29712400	-0.60261700	1.06007300
C	2.82196400	1.17991800	-0.46009500
C	3.70212700	-0.78760900	1.30054400
C	4.16709000	1.04032800	-0.21519900
C	4.64212600	-0.01046400	0.63417300
H	4.02468400	-1.52043900	2.03103600
H	4.86032000	1.76113800	-0.62796200
C	-2.13078900	-2.33932300	-0.37497600
C	-1.21235500	-2.70182700	0.61573900
C	-0.32423200	-3.75175700	0.36676800
H	-1.20389600	-2.17712600	1.55954000
C	-1.35738800	-3.98464500	-1.78389700
C	-0.38572800	-4.40232200	-0.86752600
H	0.38668300	-4.05699200	1.12751100
H	-1.44734600	-4.48244400	-2.74742600
H	0.28279100	-5.22133200	-1.11023600
C	-2.85912400	2.12989000	1.30199700
C	-3.62383000	3.01159600	2.07511100
C	-3.01484200	4.13015100	2.63903800
H	-4.67390500	2.81024600	2.24946600
C	-0.95441600	3.42123800	1.65513900
C	-1.65322300	4.34597300	2.42431600
H	-3.59498500	4.81877000	3.24460200
H	0.10489200	3.53231200	1.44768600
H	-1.14064700	5.20344800	2.84466800
C	2.26016500	2.29863900	-1.25328300
C	2.96711400	2.91229800	-2.29640700
C	2.38430100	3.98359600	-2.96872100
H	3.94389500	2.54183100	-2.58596800
C	0.46950100	3.73527200	-1.55510200
C	1.11095800	4.41327400	-2.58985900
H	2.91274800	4.46979700	-3.78257600
H	-0.52892500	4.01827700	-1.23270900
H	0.62144600	5.24312100	-3.08713300
C	1.30125400	-0.85270400	2.15746300
C	1.49953000	-1.81938400	3.15181700
C	0.54911600	-1.95868600	4.15846800

H	2.37497200	-2.45719200	3.13141500
C	-0.70805200	-0.20915700	3.12225300
C	-0.57295000	-1.12539900	4.16023300
H	0.68347000	-2.70552300	4.93443000
H	-1.57768300	0.43370000	3.04858300
H	-1.32993900	-1.19336500	4.93302200
N	5.98932500	-0.22067300	0.80409200
N	-6.54951300	-0.24853100	-0.89414800
H	-7.12013800	0.56895700	-0.74278400
N	-2.21932300	-2.98827300	-1.55287100
N	-2.59668800	-0.12067400	0.48404100
N	-1.53833300	2.34281900	1.10913100
N	0.19586900	-0.08162900	2.13943100
N	1.87826000	0.36212600	0.12694000
N	1.02202600	2.70626300	-0.90069500
H	6.24226000	-0.81783600	1.57812500
C	-0.82752500	0.52333300	-1.98966800
O	-0.23765800	-0.14410200	-2.84090800
O	-1.95551500	1.20022600	-2.41885200
H	-2.06328100	0.98800500	-3.36912700
C	-7.12418100	-1.34921000	-1.64679300
H	-7.10950300	-2.28052000	-1.06810400
H	-8.16119500	-1.10740100	-1.88056600
H	-6.58688300	-1.52007500	-2.58794300
C	7.00516500	0.73148200	0.38471100
H	6.88583900	1.71955900	0.85161400
H	7.98330800	0.33552800	0.66167200
H	6.99295500	0.86091100	-0.70259200
Zn	-0.32727100	0.85770200	0.06193700
Pt	1.60676900	-1.56814700	-0.68207200

Sum of electronic and thermal Free Energies = -2048.268978 (Hartree/Particle).

Supplementary Table 49. DFT-optimized geometry of $[Zn^{II}(TPY)_2]^{2+}$·Pt-CO (singlet), computed at the B3LYP-D3/ LANL2DZ (Zn, Pt)/ 6-31G** (HCNO) level in acetonitrile solvent using IEFPCM.

Atom	x	y	z
C	-5.40999600	0.82813400	-0.09968000
C	-4.50985000	1.91309900	-0.25811800
C	-3.16330100	1.66056100	-0.45356100
C	-3.51035000	-0.64344000	-0.36345500
C	-4.87299100	-0.48162700	-0.15593000
H	-4.89522200	2.92449000	-0.22903100
H	-5.51969600	-1.33824900	-0.03500400
C	-2.84985600	-1.97761400	-0.45769600
C	-3.55844400	-3.18222700	-0.48073900
C	-2.85886100	-4.38139000	-0.60449400
H	-4.63899200	-3.19235600	-0.41690400
C	-0.83269800	-3.11313700	-0.68121700
C	-1.46987900	-4.35112600	-0.71206400
H	-3.39626200	-5.32362800	-0.62563300
H	0.24560300	-3.02702600	-0.75840400

H	-0.88765900	-5.25870800	-0.82139200
C	-2.14541200	2.73343000	-0.64541600
C	-2.46377500	4.09525700	-0.62630800
C	0.08269100	3.22451500	-1.06906600
C	-1.45765300	5.03567100	-0.83648800
H	-3.47920600	4.42630300	-0.45052300
C	-0.15643000	4.59608900	-1.06988600
H	1.07871600	2.83884500	-1.24709400
H	-1.69168300	6.09489300	-0.82161900
H	0.65738500	5.28976100	-1.24593300
N	-0.87641900	2.31394000	-0.85509300
N	-2.67625200	0.40329400	-0.49901000
N	-1.50045200	-1.95875400	-0.54794500
N	-6.72688000	1.05301500	0.09239500
H	-7.04110800	2.01145200	0.11919300
C	-7.72625000	0.00823400	0.24796500
H	-8.70209500	0.47844400	0.36581200
H	-7.76015200	-0.64505300	-0.63153200
H	-7.52839100	-0.60649600	1.13366200
C	3.63062900	2.45472600	0.24720400
C	2.69265700	2.28481400	1.30764700
C	1.73393700	1.30459100	1.21746200
C	2.45068000	0.71889000	-0.94983700
C	3.44632200	1.67188700	-0.92913200
H	2.72050000	2.95343500	2.15976800
H	4.03504900	1.86078000	-1.81618400
C	2.06799600	-0.04631300	-2.16474100
C	2.97218000	-0.38685000	-3.17029600
C	2.50871900	-1.09395600	-4.27974400
H	4.01958700	-0.12683400	-3.07378700
C	0.31972500	-1.06350600	-3.30560200
C	1.15984300	-1.43868100	-4.35271700
H	3.19523300	-1.37733500	-5.07032500
H	-0.73655800	-1.31164300	-3.30862500
H	0.76109200	-1.98899600	-5.19674600
C	0.65578100	1.10334200	2.22096600
C	0.79556500	1.44799900	3.56599800
C	-1.50857700	0.35152200	2.57344400
C	-0.27530700	1.22641600	4.43168400
H	1.72890400	1.85735200	3.93370200
C	-1.44953600	0.66720100	3.93037500
H	-2.40234700	-0.07876900	2.13492600
H	-0.18749100	1.47948300	5.48284400
H	-2.30355400	0.47541700	4.56948400
N	-0.48593600	0.56303100	1.73638100
N	1.64997700	0.46988100	0.13113000
N	0.75864800	-0.38427600	-2.23726200
N	4.60059900	3.37268900	0.34282800
H	4.64151700	3.92249200	1.18933700
C	5.58536400	3.65707100	-0.69306500
H	6.28100500	4.40201300	-0.30874000
H	6.14733600	2.75512200	-0.95530700
H	5.10860400	4.05495900	-1.59547100
Zn	-0.55839400	0.07100200	-0.50365500
Pt	2.02510800	-1.61127000	0.81571600
C	2.46509800	-3.24257200	1.44542000
O	2.75651700	-4.28961800	1.86073300

Sum of electronic and thermal Free Energies = -1972.393501 (Hartree/Particle).

Supplementary Table 50. DFT-optimized geometry of $[Zn^{II}(TPY^{\bullet -})(TPY)]^{+}\cdot$Pt-CO (doublet), computed at the UB3LYP-D3/ LANL2DZ (Zn, Pt)/ 6-31G** (HCNO) level in acetonitrile solvent using IEFPCM. The spin density plot is also shown below. (Isovalue = 0.004)

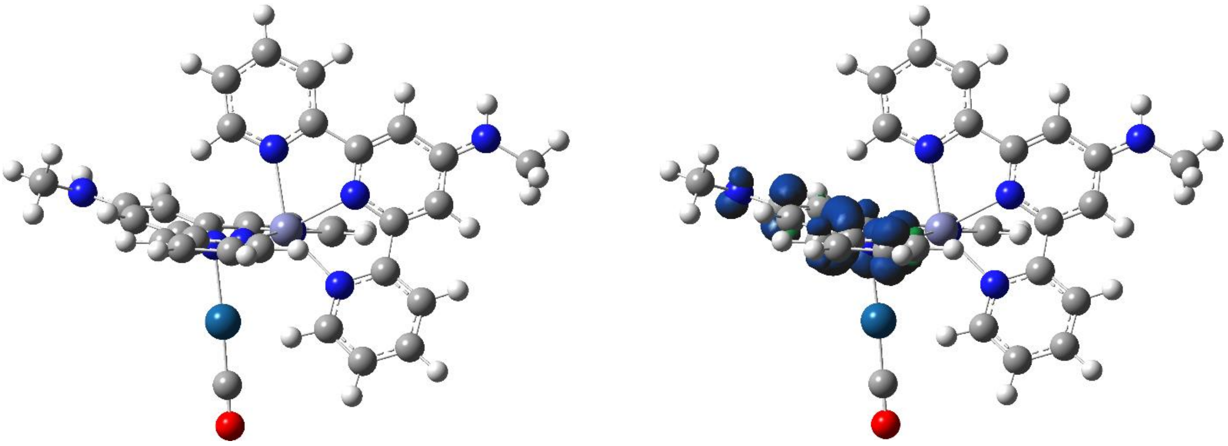

Spin Density Distribution

Atom	x	y	z
C	5.32406600	0.87074100	-0.00939700
C	4.41848800	1.94330000	0.19103900
C	3.09396400	1.67426500	0.49377800
C	3.47396800	-0.62187700	0.42755200
C	4.81562700	-0.44487600	0.11467500
H	4.77702700	2.96022100	0.09049500
H	5.46021400	-1.29608800	-0.05141100
C	2.84635600	-1.96647000	0.57763200
C	3.58560700	-3.14698300	0.69825000
C	2.91059900	-4.35546500	0.86313100
H	4.66815700	-3.12734500	0.68429100
C	0.85152900	-3.13804700	0.79354200
C	1.51768100	-4.35581800	0.91560100
H	3.46913000	-5.28064100	0.96046400
H	-0.23113400	-3.07157700	0.82748300
H	0.95488300	-5.27192000	1.05356000
C	2.07225400	2.74024100	0.71038000
C	2.39057900	4.10173800	0.76322600
C	-0.17349400	3.20919800	1.06258500
C	1.37261800	5.03103900	0.96813500
H	3.41347200	4.43979000	0.65552200
C	0.06276200	4.58124500	1.12163000
H	-1.17473400	2.80908700	1.17601900
H	1.60425000	6.09023900	1.01077700
H	-0.75946000	5.26860800	1.28443300
N	0.80026300	2.31181700	0.86167900
N	2.63156500	0.41146200	0.60546100
N	1.49650100	-1.97712300	0.61973300
N	6.61830800	1.11238800	-0.31311800
H	6.91012300	2.07442600	-0.39657500
C	7.61308700	0.07955800	-0.55068500
H	8.56724600	0.56093400	-0.76382400
H	7.73854700	-0.56365200	0.32800200
H	7.34253100	-0.54889400	-1.40742200

C	-3.70902100	2.24980000	-0.43781600
C	-2.67858300	2.13766200	-1.43572400
C	-1.67018500	1.23027900	-1.27584300
C	-2.44808400	0.68058600	0.92892200
C	-3.54473000	1.54360300	0.75694300
H	-2.68971200	2.80906000	-2.28708700
H	-4.21127000	1.72695200	1.58975500
C	-2.04175300	0.10753700	2.18359600
C	-2.88161200	0.00873900	3.32391100
C	-2.40994900	-0.58155600	4.47817000
H	-3.90126700	0.37387500	3.27552200
C	-0.31567800	-0.95646300	3.37653400
C	-1.08998000	-1.08571100	4.51824500
H	-3.05521100	-0.66655700	5.34728000
H	0.70764000	-1.32200900	3.34492700
H	-0.68868400	-1.56175700	5.40515000
C	-0.54336000	1.08498300	-2.22124700
C	-0.61404400	1.48438300	-3.56351700
C	1.64018500	0.34308600	-2.51929800
C	0.49383300	1.30845700	-4.38515200
H	-1.53129500	1.90409900	-3.95922400
C	1.64997500	0.72564700	-3.85680200
H	2.51204000	-0.11420600	-2.06251500
H	0.45383100	1.61088900	-5.42659900
H	2.53312700	0.56447400	-4.46429100
N	0.57835900	0.51283200	-1.71863100
N	-1.60322100	0.38545100	-0.16725200
N	-0.75473000	-0.38392300	2.24222300
N	-4.77180400	3.08131600	-0.67611500
H	-4.67086300	3.72897500	-1.44371600
C	-5.73132300	3.43964300	0.35136900
H	-6.47109400	4.11609100	-0.07847600
H	-6.25481100	2.54872300	0.71594800
H	-5.26039400	3.93522800	1.21313200
Zn	0.48503300	0.03867800	0.52571300
Pt	-1.99717400	-1.65270500	-0.83368200
C	-2.39615100	-3.30076400	-1.45456300
O	-2.65772600	-4.35701300	-1.87455700

Sum of electronic and thermal Free Energies = -1972.517746 (Hartree/Particle).

Supplementary Table 51. DFT-optimized geometry of $[Zn^{II}(TPY^{\bullet -})(TPY)]^{+}\cdot$Pt-CHO (singlet), computed at the B3LYP-D3/ LANL2DZ (Zn, Pt)/ 6-31G** (HCNO) level in acetonitrile solvent using IEFPCM.

Atom	x	y	z
C	5.47492700	0.74399400	0.18151600
C	4.60298800	1.85120500	0.33439900
C	3.24014900	1.63786700	0.45557300
C	3.51795700	-0.67072700	0.30591000
C	4.89476800	-0.54668100	0.17286200
H	5.02097600	2.84974200	0.36011700
H	5.52014600	-1.42017400	0.06131100

C	2.81887400	-1.98859000	0.31271300
C	3.49068700	-3.21320200	0.26053500
C	2.75127900	-4.39605500	0.26262000
H	4.57167200	-3.25419800	0.21957700
C	0.75980300	-3.07282100	0.37644600
C	1.36145500	-4.32855900	0.31830600
H	3.26091900	-5.35321500	0.21820100
H	-0.32199900	-2.95573400	0.38460600
H	0.73961700	-5.21560900	0.30836400
C	2.25658700	2.74499000	0.63834600
C	2.62689800	4.09480000	0.65516000
C	0.03617200	3.31068600	0.99360900
C	1.64992800	5.06775300	0.85251500
H	3.65847300	4.39174900	0.51585800
C	0.32580400	4.67251600	1.03174000
H	-0.98105700	2.95963100	1.12287000
H	1.92295300	6.11773300	0.86719900
H	-0.46708500	5.39363200	1.19344900
N	0.96896800	2.36944900	0.79900900
N	2.70864000	0.39746800	0.43127600
N	1.46910800	-1.93470200	0.37249400
N	6.80708700	0.93113300	0.05612400
H	7.15208000	1.87889700	0.07054400
C	7.77663300	-0.14068300	-0.09948500
H	8.77107100	0.29972000	-0.16788400
H	7.75866500	-0.82367300	0.75781200
H	7.59064700	-0.72006400	-1.01142700
C	-3.73605900	2.37069200	-0.33573100
C	-2.80759400	2.21447300	-1.40180400
C	-1.73259300	1.36341200	-1.24491500
C	-2.37427100	0.84426400	0.94993900
C	-3.48047400	1.67364800	0.87500300
H	-2.94326200	2.78239700	-2.31456600
H	-4.11918400	1.81406900	1.73563100
C	-1.96328300	0.12455700	2.18738400
C	-2.84501500	-0.16833100	3.22975600
C	-2.36719700	-0.85332500	4.34614900
H	-3.89015600	0.10754600	3.15878400
C	-0.20800300	-0.89911300	3.31413300
C	-1.02478000	-1.22808600	4.39445300
H	-3.03876000	-1.09825900	5.16229200
H	0.84213100	-1.17332300	3.29196200
H	-0.61634500	-1.76574900	5.24218600
C	-0.67823700	1.14799400	-2.27424400
C	-0.85302200	1.46707600	-3.62302100
C	1.47652400	0.38656200	-2.68046000
C	0.19165200	1.22922600	-4.51512200
H	-1.79310800	1.87310600	-3.97632000
C	1.38148800	0.67920900	-4.03997400
H	2.37976300	-0.04041000	-2.25663100
H	0.07260600	1.46490200	-5.56745200
H	2.21657400	0.47682700	-4.70059400
N	0.47718200	0.61282800	-1.82017700
N	-1.52687700	0.67118200	-0.09391500
N	-0.66207800	-0.23883200	2.24177900
N	-4.80023100	3.18404600	-0.47579500
H	-4.91220900	3.66079700	-1.35809200
C	-5.79650300	3.42851400	0.55566200
H	-6.54830900	4.10767800	0.15453500
H	-6.29209500	2.49883100	0.85644700

H	-5.34821200	3.89066700	1.44260600
Zn	0.55817000	0.09917700	0.39596000
Pt	-2.11401100	-1.60295500	-0.70373400
C	-2.56640300	-3.43513200	-1.09717500
H	-3.47591000	-3.60063700	-1.74247600
O	-1.98528100	-4.46406100	-0.71860000

Sum of electronic and thermal Free Energies = -1973.077026 (Hartree/Particle).

Supplementary Table 52. DFT-optimized geometry of $[Zn^{II}(TPY^{\bullet-})(TPY)]^{+}\cdot Pt\text{-}(\eta^2\text{-}OCH_2)$ (doublet), computed at the UB3LYP-D3/ LANL2DZ (Zn, Pt)/ 6-31G** (HCNO) level in acetonitrile solvent using IEFPCM. The spin density plot is also shown below. (Isovalue = 0.004)

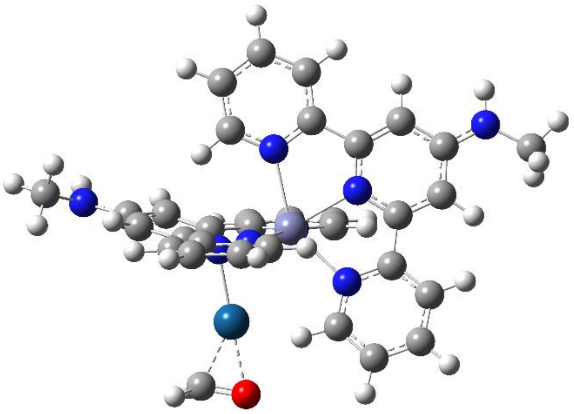

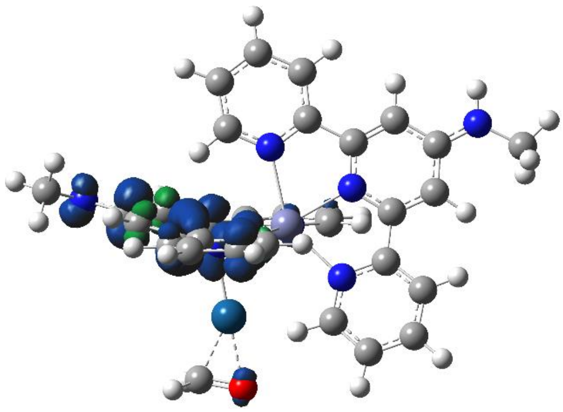

Spin Density Distribution

Atom	x	y	z
C	5.38461000	0.82042400	0.01342500
C	4.48529400	1.89884200	0.21161000
C	3.15016000	1.63802000	0.47150800
C	3.50727900	-0.66155800	0.36235200
C	4.85878300	-0.49198000	0.09148200
H	4.85827100	2.91341000	0.14637800
H	5.49975300	-1.34569000	-0.07497300
C	2.86067600	-2.00229200	0.46278100
C	3.58409200	-3.19623100	0.53959400
C	2.89362600	-4.40212000	0.64980400
H	4.66680700	-3.19052000	0.53135400
C	0.84981700	-3.15776400	0.61738500
C	1.50054000	-4.38769300	0.69115700
H	3.44038700	-5.33730900	0.71164500
H	-0.23353000	-3.08288000	0.64664000
H	0.92543700	-5.30175200	0.78418300
C	2.13366600	2.70936400	0.68807800
C	2.46275400	4.06740900	0.75867900
C	-0.11161900	3.19513700	1.02467100
C	1.45140600	5.00367900	0.96414500
H	3.48930500	4.39774200	0.66315800
C	0.13638700	4.56409000	1.10123400
H	-1.11725700	2.80352400	1.12658100
H	1.69215800	6.06020500	1.02000500

H	-0.68119400	5.25676900	1.26469000
N	0.85576900	2.29085000	0.82196100
N	2.67294000	0.37797600	0.54173400
N	1.51007900	-1.99803600	0.49891900
N	6.69023500	1.05391700	-0.24419900
H	6.99563700	2.01411500	-0.29338900
C	7.68217800	0.01551600	-0.46946000
H	8.64849500	0.49086400	-0.63622500
H	7.76855400	-0.64893700	0.39806100
H	7.43697500	-0.58996300	-1.35001600
C	-3.70498200	2.23165400	-0.33875900
C	-2.71974300	2.11626400	-1.37325200
C	-1.70241100	1.20847300	-1.25976200
C	-2.39954000	0.65872400	0.97767200
C	-3.48246800	1.53361500	0.85915500
H	-2.75953400	2.79542400	-2.21819400
H	-4.09432100	1.74217500	1.72742300
C	-1.93928400	0.08673700	2.22214000
C	-2.74107000	-0.02671600	3.38339900
C	-2.22253800	-0.61432100	4.52211000
H	-3.76738100	0.32164000	3.36733900
C	-0.16316800	-0.95475000	3.35017300
C	-0.89887400	-1.09798700	4.51712300
H	-2.83851200	-0.71206500	5.41076300
H	0.86284600	-1.30651300	3.28146000
H	-0.46226800	-1.57183900	5.38835800
C	-0.59180700	1.09267200	-2.21874100
C	-0.67781900	1.53038300	-3.55044300
C	1.59467500	0.36928700	-2.56796000
C	0.41868900	1.38003000	-4.39001900
H	-1.60024700	1.95991900	-3.92314000
C	1.58546700	0.78616600	-3.89444600
H	2.47417800	-0.09444200	-2.13251300
H	0.36358000	1.71097400	-5.42215700
H	2.46026100	0.64421400	-4.51843300
N	0.54268700	0.51222100	-1.74897300
N	-1.62231600	0.33393200	-0.16168600
N	-0.65074000	-0.38498000	2.23575400
N	-4.77683900	3.06847400	-0.52574800
H	-4.69974100	3.72211700	-1.29145800
C	-5.66931600	3.44979300	0.55329700
H	-6.43087500	4.12308400	0.15743500
H	-6.17457900	2.56830100	0.96309500
H	-5.14583500	3.95748800	1.37706000
Zn	0.52846400	0.03332800	0.46396600
Pt	-1.99535600	-1.61922500	-0.78828500
C	-3.45092100	-2.90662900	-1.47183800
H	-4.23559500	-3.09355400	-0.72105000
H	-3.83910800	-2.61303600	-2.46049700
O	-2.33234500	-3.60146000	-1.40880500

Sum of electronic and thermal Free Energies = -1973.638693 (Hartree/Particle).

Supplementary Table 53. DFT-optimized geometry of $[Zn^{II}(TPY^{\bullet-})(TPY)]^{+}$·Pt-$OCH_3$ (singlet), computed at the B3LYP-D3/ LANL2DZ (Zn, Pt)/ 6-31G** (HCNO) level in acetonitrile solvent using IEFPCM.

Atom	x	y	z
C	5.57899800	0.32788000	0.25958100
C	4.78121000	1.47057900	0.52539300
C	3.40805000	1.33416500	0.63670100
C	3.53485400	-0.96139700	0.24446600
C	4.91635800	-0.91574400	0.11398800
H	5.26075100	2.43681600	0.62395300
H	5.48011600	-1.80908700	-0.11319200
C	2.73959900	-2.21573700	0.09339900
C	3.31215500	-3.48904400	0.02972900
C	2.48120200	-4.60086200	-0.10562700
H	4.38476600	-3.61977600	0.09854300
C	0.60169500	-3.12012600	-0.09456000
C	1.10062600	-4.41883300	-0.16978100
H	2.91111100	-5.59595400	-0.15359700
H	-0.46339200	-2.89797100	-0.13620000
H	0.42114700	-5.25741700	-0.27067500
C	2.48481300	2.47740800	0.90221600
C	2.92773400	3.77216000	1.19262300
C	0.26742600	3.14755000	1.08379800
C	1.98702200	4.77370000	1.42692800
H	3.98469400	4.00161100	1.24428700
C	0.62920200	4.46162300	1.37378000
H	-0.77570600	2.84994600	1.03370400
H	2.31425100	5.78336200	1.65245800
H	-0.13317100	5.21101800	1.55301900
N	1.16793500	2.18508700	0.85179600
N	2.80099000	0.13677700	0.49918000
N	1.39951200	-2.05136300	0.03079800
N	6.92018700	0.44114900	0.14480600
H	7.32364800	1.36054100	0.24273800
C	7.81794400	-0.66515000	-0.14366000
H	8.83884700	-0.28468300	-0.16722900
H	7.75557400	-1.44078900	0.62826100
H	7.59499100	-1.12082600	-1.11568700
C	-3.43498400	2.40860700	-0.40397000
C	-2.43279800	2.34312300	-1.44027300
C	-1.43544700	1.41604700	-1.37131000
C	-2.29536300	0.54335600	0.69724700
C	-3.34408300	1.53575700	0.66606200
H	-2.46351800	3.06222400	-2.25111000
H	-4.04444900	1.58715900	1.48758600
C	-1.82948900	-0.01860500	2.00677600
C	-2.70523600	-0.24890000	3.07674300
C	-2.20817100	-0.79170300	4.25613900
H	-3.76086200	-0.02801900	2.97407100
C	-0.03958900	-0.83454600	3.25297800
C	-0.84628500	-1.09413500	4.35433100
H	-2.87557200	-0.98065700	5.09098400
H	1.02508100	-1.04898500	3.26708300
H	-0.42177600	-1.51948500	5.25622100
C	-0.33328400	1.29377300	-2.35548200
C	-0.41408700	1.78979300	-3.66221500
C	1.82136000	0.51127100	-2.73804500
C	0.67728400	1.63565400	-4.51311300
H	-1.32108200	2.26903000	-4.01117500
C	1.82197200	0.98586900	-4.04721400
H	2.68392700	-0.00162400	-2.32324000

H	0.63108000	2.01079600	-5.53030200
H	2.69028800	0.84279300	-4.68001900
N	0.77480500	0.65639500	-1.91560400
N	-1.31996600	0.53981500	-0.30011900
N	-0.51359400	-0.30741400	2.11246600
N	-4.43025200	3.34219200	-0.53154900
H	-4.25859600	4.09037000	-1.18706400
C	-5.36195500	3.62929600	0.54353100
H	-6.02907800	4.43192100	0.22669900
H	-5.97167200	2.74740300	0.76922800
H	-4.85214300	3.93728600	1.46824200
Zn	0.66308900	0.02242100	0.26821800
Pt	-2.49750500	-1.21099500	-0.53686100
O	-3.46702600	-2.85572400	-1.07454800
C	-3.15171900	-4.08720600	-0.46067000
H	-3.30817500	-4.07466300	0.63101300
H	-3.80150100	-4.87477900	-0.87553800
H	-2.11001500	-4.40896500	-0.63340800

Sum of electronic and thermal Free Energies = -1974.252585 (Hartree/Particle).

Supplementary Table 54. DFT-optimized geometry of $[Zn^{II}(TPY)_2]^{2+}$·Pt-O^{-} (doublet), computed at the UB3LYP-D3/ LANL2DZ (Zn, Pt)/ 6-31G** (HCNO) level in acetonitrile solvent using IEFPCM. The spin density plot is also shown below. (Isovalue = 0.004)

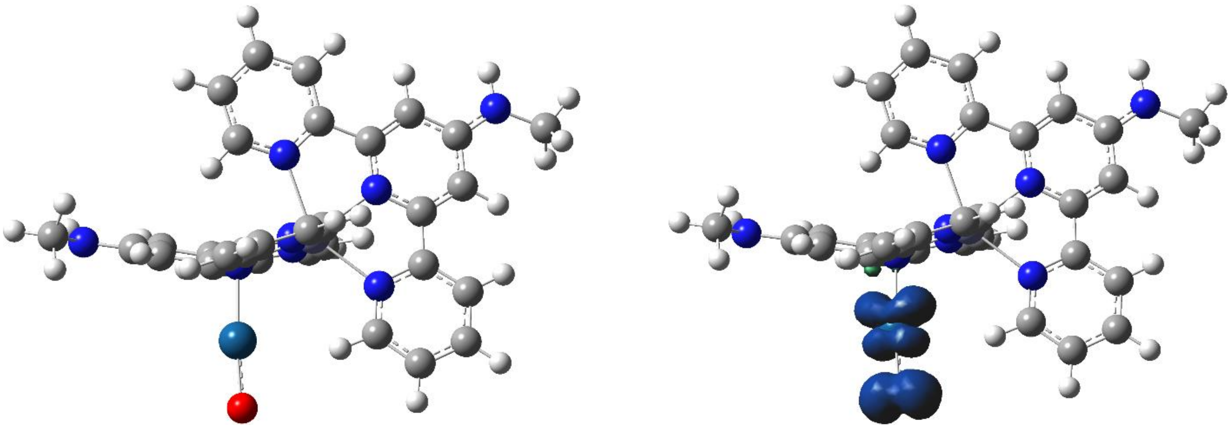

Spin Density Distribution

Atom	x	y	z
C	-5.48150600	0.66938800	-0.28192300
C	-4.58465900	1.73717900	-0.54063600
C	-3.22472000	1.48756000	-0.60970300
C	-3.55180800	-0.78393600	-0.19657300
C	-4.92760800	-0.62205300	-0.10717900
H	-4.98020100	2.73692400	-0.67063500
H	-5.56973600	-1.46346300	0.10900400
C	-2.87642700	-2.10330100	-0.02100600
C	-3.56789200	-3.31595300	0.05096800
C	-2.84919300	-4.49929800	0.21445100
H	-4.64700800	-3.34595100	-0.03082400

C	-0.83771900	-3.20308000	0.21117400
C	-1.45899000	-4.44722000	0.29845200
H	-3.37236900	-5.44820900	0.27072800
H	0.24245200	-3.09334700	0.26857800
H	-0.86355800	-5.34408200	0.42486000
C	-2.20989600	2.54927500	-0.87788700
C	-2.54779600	3.86669600	-1.20697100
C	0.05281400	3.04232000	-1.04729100
C	-1.53087800	4.78655100	-1.45533800
H	-3.58280700	4.17604600	-1.27919800
C	-0.20237600	4.37154200	-1.37777500
H	1.06984300	2.66973800	-0.98027600
H	-1.77682700	5.81171800	-1.71195100
H	0.61824000	5.05334300	-1.56925300
N	-0.92059500	2.15739300	-0.79960400
N	-2.71990200	0.24680700	-0.43636600
N	-1.52761300	-2.06479500	0.05607000
N	-6.81108500	0.89362500	-0.20407400
H	-7.13568700	1.84129700	-0.32337800
C	-7.80382200	-0.13318100	0.06728100
H	-8.79015000	0.33008400	0.06322600
H	-7.78638200	-0.91696300	-0.69868200
H	-7.64293300	-0.59835300	1.04695500
C	3.70291200	2.03270500	0.34903700
C	2.72340800	2.05524200	1.41041500
C	1.65472100	1.21092400	1.37260000
C	2.36579700	0.29967600	-0.73887900
C	3.49697000	1.18855000	-0.73184300
H	2.82996500	2.77681900	2.21227800
H	4.17223700	1.19208200	-1.57535200
C	1.80175200	-0.21570600	-2.02683400
C	2.60100000	-0.46411400	-3.15030600
C	2.00991000	-0.96188900	-4.30606200
H	3.66993100	-0.29414200	-3.10926300
C	-0.09503200	-0.92838000	-3.17461600
C	0.63255300	-1.20201600	-4.32652200
H	2.61707400	-1.16500600	-5.18240900
H	-1.16730500	-1.09355000	-3.12816800
H	0.13638800	-1.59122800	-5.20785700
C	0.57113500	1.17844000	2.38263300
C	0.71908700	1.67726000	3.68180700
C	-1.62960400	0.56819400	2.81888700
C	-0.36294500	1.61745500	4.55646300
H	1.66793700	2.08690200	4.00695700
C	-1.56481000	1.05570300	4.12152400
H	-2.53730400	0.11963200	2.42667100
H	-0.26593600	1.99778700	5.56805400
H	-2.42824500	0.98888400	4.77322800
N	-0.59209700	0.62383400	1.97395400
N	1.45795700	0.33259200	0.31565600
N	0.47068300	-0.44560400	-2.05690500
N	4.77952300	2.86541900	0.45116600
H	4.73460100	3.58931800	1.15247500
C	5.73763900	3.04611000	-0.62300500
H	6.49332000	3.76431200	-0.30361600
H	6.23984100	2.10018300	-0.85496400
H	5.26454100	3.41739200	-1.54319000
Zn	-0.60456100	-0.04590600	-0.17611900
Pt	2.50571300	-1.53035800	0.51196300
O	3.24388300	-3.14772700	0.96063800

Sum of electronic and thermal Free Energies = -1934.331037 (Hartree/Particle).

Supplementary Table 55. DFT-optimized geometry of $[Zn^{II}(TPY^{\bullet -})(TPY)]^{+}$·Pt-OH (singlet), computed at the B3LYP-D3/ LANL2DZ (Zn, Pt)/ 6-31G** (HCNO) level in acetonitrile solvent using IEFPCM.

Atom	x	y	z
C	-5.50359400	0.62388600	-0.30813800
C	-4.62042500	1.71029400	-0.53441400
C	-3.25465000	1.48608600	-0.56998500
C	-3.54987300	-0.79408200	-0.18124600
C	-4.93035000	-0.65812400	-0.12742400
H	-5.03042900	2.70391500	-0.66716500
H	-5.56227200	-1.51277800	0.06551400
C	-2.85522900	-2.10207800	0.00594000
C	-3.52659500	-3.32666800	0.06858600
C	-2.79040500	-4.49673100	0.24981000
H	-4.60332800	-3.37632900	-0.03296400
C	-0.80258800	-3.16523800	0.28173500
C	-1.40327600	-4.41988500	0.36209200
H	-3.29794900	-5.45454900	0.29870000
H	0.27509400	-3.02976100	0.35674700
H	-0.79498100	-5.30577600	0.50439000
C	-2.25278500	2.56840400	-0.80389200
C	-2.60480400	3.88621800	-1.11637800
C	0.00554800	3.09728200	-0.92396900
C	-1.59709100	4.82489600	-1.33003100
H	-3.64285600	4.18195800	-1.20149800
C	-0.26387200	4.42840500	-1.23552900
H	1.02619700	2.73627200	-0.84494200
H	-1.85368600	5.85086600	-1.57275100
H	0.54927600	5.12609000	-1.39989200
N	-0.95946900	2.19499400	-0.70952700
N	-2.73150700	0.25342600	-0.39317600
N	-1.50906000	-2.04040000	0.10718800
N	-6.83917300	0.82174800	-0.26516300
H	-7.17939500	1.76349200	-0.38713000
C	-7.81849300	-0.22713800	-0.03263400
H	-8.81343400	0.21656200	-0.05963700
H	-7.76423000	-1.00214100	-0.80587100
H	-7.67577100	-0.69982300	0.94623900
C	3.69906900	2.03151900	0.35061600
C	2.75366700	2.01273900	1.44120000
C	1.68236000	1.17046900	1.40453900
C	2.34681800	0.30535300	-0.74022700
C	3.47205200	1.21253900	-0.74121300
H	2.88506500	2.70363400	2.26633300
H	4.12509500	1.23928700	-1.60196300
C	1.75137600	-0.16114300	-2.03648200
C	2.52994900	-0.39306400	-3.17830000
C	1.91412000	-0.84670000	-4.33951900
H	3.60240700	-0.24343000	-3.14687800

C	-0.17424500	-0.80297500	-3.17886700
C	0.53199300	-1.05860400	-4.34834300
H	2.50560900	-1.03612000	-5.22968300
H	-1.24924300	-0.94742300	-3.12187900
H	0.01645800	-1.41191500	-5.23374800
C	0.62077800	1.12005100	2.43815000
C	0.80538400	1.56968900	3.75107800
C	-1.58196700	0.53558800	2.90213300
C	-0.25819600	1.49781700	4.64695300
H	1.76863200	1.95057600	4.06903300
C	-1.48012200	0.97405600	4.21970600
H	-2.50588400	0.11713100	2.51395100
H	-0.13186200	1.83920600	5.66923000
H	-2.32998000	0.89891000	4.88820000
N	-0.56221300	0.60256900	2.03685600
N	1.44125600	0.33587400	0.32082600
N	0.41547700	-0.36377000	-2.05590200
N	4.77729000	2.87274300	0.45021800
H	4.70556600	3.61083200	1.13532800
C	5.66845000	3.11910600	-0.66834500
H	6.42391400	3.84455500	-0.36420800
H	6.18054700	2.19588200	-0.96123700
H	5.13788800	3.50924100	-1.54938300
Zn	-0.61119400	-0.01289700	-0.13430000
Pt	2.49645600	-1.50279500	0.40862400
O	3.33893800	-3.25243400	0.81173500
H	2.82809700	-3.91608100	0.32474000

Sum of electronic and thermal Free Energies = -1934.971549 (Hartree/Particle).

Supplementary Table 56. DFT-optimized geometry of TEA (singlet), computed at the B3LYP-D3/ 6-31G** (HCN) level in acetonitrile solvent using IEFPCM.

Atom	x	y	z
N	0.00007100	-0.00019300	-0.00536000
C	-1.27834800	-0.58097000	0.43417700
C	-1.73075500	-1.76211700	-0.42398700
H	-2.04263800	0.19667400	0.37856700
H	-1.22215700	-0.88220800	1.49627600
H	-2.68797300	-2.14752900	-0.06079600
H	-1.85594600	-1.45025500	-1.46434300
H	-1.01372900	-2.58661100	-0.40187700
C	1.14226100	-0.81697500	0.43400700
C	2.39177700	-0.61718200	-0.42322000
H	0.85116100	-1.86768200	0.37730000
H	1.37456100	-0.61869200	1.49645800
H	3.20436400	-1.25282600	-0.05933600
H	2.18567000	-0.88157800	-1.46379600
H	2.74642400	0.41627300	-0.40065800
C	0.13608700	1.39743400	0.43422700
C	-0.66104400	2.37986800	-0.42355700
H	1.19156600	1.67089200	0.37872000
H	-0.15294000	1.49925800	1.49633900
H	-0.51755500	3.40129900	-0.05911100

H	-0.32781000	2.33411200	-1.46381600
H	-1.73335200	2.16988400	-0.40228500

Sum of electronic and thermal Free Energies = -292.348940 (Hartree/Particle).

Supplementary Table 57. DFT-optimized geometry of $TEA^{\bullet+}$ (doublet), computed at the UB3LYP-D3/ 6-31G** (HCN) level in acetonitrile solvent using IEFPCM.

Atom	x	y	z
N	-0.00290500	-0.00338600	-0.61630600
C	-1.30941600	-0.64370700	-0.60326900
C	-2.06942500	-0.37982600	0.70978300
H	-1.16842000	-1.71067400	-0.76761300
H	-1.87233000	-0.22998100	-1.44736900
H	-3.03681700	-0.87899700	0.63941300
H	-1.52451900	-0.78615700	1.56206400
H	-2.24340500	0.68498600	0.86772500
C	0.09731200	1.44807700	-0.60898600
C	0.70428200	1.97934500	0.70300600
H	-0.89641500	1.85973800	-0.77561200
H	0.73843500	1.72403500	-1.45310500
H	0.74393300	3.06726500	0.63329500
H	0.08457700	1.70386500	1.55652600
H	1.71803500	1.60887200	0.85879900
C	1.20471300	-0.81400300	-0.61012400
C	1.37404200	-1.58835900	0.71004200
H	2.05569000	-0.16010200	-0.79203400
H	1.11697900	-1.51780800	-1.44478000
H	2.29515000	-2.16843800	0.63813000
H	1.45370500	-0.90407600	1.55494000
H	0.54669100	-2.27798900	0.88104400

Sum of electronic and thermal Free Energies = -292.154636 (Hartree/Particle).

Supplementary Table 58. DFT-optimized geometry of $[Et_2N{=}CHCH_3]^{\bullet}$ (doublet), computed at the UB3LYP-D3/ 6-31G** (HCN) level in acetonitrile solvent using IEFPCM.

Atom	x	y	z
N	-0.04624000	-0.09736200	-0.21138200
C	1.29720800	-0.54232900	-0.60077100
H	1.86934400	0.33142600	-0.91816200
H	1.22492800	-1.20321800	-1.47631100
C	-0.35855600	1.30856600	-0.48131300
H	-1.43233900	1.45164500	-0.36824800
H	-0.11853400	1.54767400	-1.52925800
C	-1.05040400	-1.05843400	-0.25415900
C	0.37558700	2.26849900	0.45601200
H	1.46057700	2.16221800	0.37715300

H	0.12303700	3.30314600	0.20655400
H	0.09074600	2.08180800	1.49469800
C	2.03922600	-1.25257400	0.53234700
H	1.48604600	-2.13082300	0.87459800
H	3.02464700	-1.58453800	0.19238600
H	2.17356100	-0.58082100	1.38421600
C	-2.40151900	-0.79375100	0.33916400
H	-2.97592400	-0.03295100	-0.20133900
H	-2.99283200	-1.71200400	0.32247100
H	-2.34129300	-0.45578700	1.38788800
H	-0.67754000	-2.07609900	-0.21465300

Sum of electronic and thermal Free Energies = -291.710748 (Hartree/Particle).

Supplementary Table 59. DFT-optimized geometry of CH_3CN (singlet), computed at the B3LYP-D3/ 6-31G** (HCN) level in acetonitrile solvent using IEFPCM.

Atom	x	y	z
C	0.00000000	0.00000000	0.27873200
N	0.00000000	0.00000000	1.43247200
C	0.00000000	0.00000000	-1.17589200
H	0.00000000	1.02606900	-1.54811400
H	0.88860200	-0.51303500	-1.54811400
H	-0.88860200	-0.51303500	-1.54811400

Sum of electronic and thermal Free Energies = -132.783775 (Hartree/Particle).

Supplementary Table 60. DFT-optimized geometry of CO_2 (singlet), computed at the B3LYP-D3/ 6-31G** (CO) level in acetonitrile solvent using IEFPCM.

Atom	x	y	z
C	0.00000000	0.00000000	0.00000000
O	0.00000000	0.00000000	1.16072300
O	0.00000000	0.00000000	-1.16072300

Sum of electronic and thermal Free Energies = -188.659006 (Hartree/Particle).

Supplementary Table 61. DFT-optimized geometry of H_3O^+ (singlet), computed at the B3LYP-D3/ 6-31G** (HO) level in acetonitrile solvent using IEFPCM.

Atom	x	y	z
O	0.00000000	0.00000000	0.08286700

H	0.00000000	0.92978900	-0.22098000
H	0.80522100	-0.46489400	-0.22098000
H	-0.80522100	-0.46489400	-0.22098000

Sum of electronic and thermal Free Energies = -76.836543 (Hartree/Particle).

Supplementary Table 62. DFT-optimized geometry of H_2O (singlet), computed at the B3LYP-D3/ 6-31G** (HO) level in acetonitrile solvent using IEFPCM.

Atom	x	y	z
O	0.00000000	0.00000000	0.11796700
H	0.00000000	0.76216800	-0.47186700
H	0.00000000	-0.76216800	-0.47186700

Sum of electronic and thermal Free Energies = -76.462748 (Hartree/Particle).

Supplementary Table 63. DFT-optimized geometry of CO (singlet), computed at the B3LYP-D3/ 6-31G** (CO) level in acetonitrile solvent using IEFPCM.

Atom	x	y	z
C	0.00000000	0.00000000	-0.64432600
O	0.00000000	0.00000000	0.48324500

Sum of electronic and thermal Free Energies = -113.363902 (Hartree/Particle).

Supplementary Table 64. DFT-optimized geometry of CH_4 (singlet), computed at the B3LYP-D3/ 6-31G** (HC) level in acetonitrile solvent using IEFPCM.

Atom	x	y	z
C	0.00000000	0.00000000	0.00000000
H	0.62987900	0.62987900	0.62987900
H	-0.62987900	-0.62987900	0.62987900
H	-0.62987900	0.62987900	-0.62987900
H	0.62987900	-0.62987900	-0.62987900

Sum of electronic and thermal Free Energies = -40.507300 (Hartree/Particle).

Supplementary Table 65. DFT-optimized geometry of $[Zn^{II}(TPY^{\bullet -})(TPY)]^{+}\cdot Pt(H)$-CO (singlet), computed at the B3LYP-D3/ LANL2DZ (Zn, Pt)/ 6-31G** (HCNO) level in acetonitrile solvent using IEFPCM.

Atom	x	y	z
C	5.15914500	0.78835700	-0.23052500
C	4.34064900	1.93350200	-0.40839000
C	3.00993800	1.88706600	-0.02939300
C	3.22736000	-0.31007500	0.72062300
C	4.56824300	-0.35474600	0.36248100
H	4.76972900	2.82428700	-0.85034400
H	5.15384400	-1.24865300	0.52222700
C	2.51453100	-1.44466000	1.37843400
C	3.15962400	-2.59072300	1.85184800
C	2.40812000	-3.57178300	2.49841000
H	4.22928700	-2.71413900	1.73871000
C	0.46397500	-2.22222600	2.15370900
C	1.03679100	-3.38560300	2.66362300
H	2.89386900	-4.46542400	2.87621000
H	-0.59687000	-2.02193200	2.24647700
H	0.42055100	-4.11942500	3.16987100
C	2.06730800	3.03377200	-0.18556800
C	2.46617800	4.29972300	-0.62801300
C	-0.12841700	3.75097100	0.02926900
C	1.51327600	5.31075600	-0.73861100
H	3.49983500	4.50289400	-0.87850900
C	0.18802500	5.03765600	-0.40303400
H	-1.14560100	3.48109100	0.29414100
H	1.80578600	6.29811000	-1.08058100
H	-0.58349100	5.79584300	-0.47347200
N	0.78193800	2.77699500	0.13624100
N	2.46354600	0.77710100	0.50938900
N	1.18327400	-1.28201200	1.52837800
N	6.45351600	0.80687600	-0.61645200
H	6.80329900	1.65082400	-1.04442600
C	7.36474400	-0.31909600	-0.49512100
H	8.33628600	-0.02145600	-0.88921300
H	7.49246200	-0.61827700	0.55153500
H	7.00941200	-1.18554500	-1.06522100
C	-4.34174600	1.32310400	-0.91140900
C	-3.32008100	1.03251300	-1.85025900
C	-2.02811800	0.80542400	-1.40818000
C	-2.65956200	1.13907400	0.81161000
C	-3.97874500	1.38068300	0.45521600
H	-3.57076200	0.99008700	-2.90282300
H	-4.72415500	1.60020200	1.20557100
C	-2.18223300	1.16045200	2.22573100
C	-3.01092000	1.43107700	3.31954800
C	-2.46652300	1.42548300	4.60250200
H	-4.06354600	1.64272000	3.18187300
C	-0.34977800	0.88523300	3.63369100
C	-1.11007300	1.14793600	4.77035300
H	-3.09746800	1.63422800	5.46010100
H	0.70860900	0.65145400	3.69752200
H	-0.65050100	1.13170900	5.75172600
C	-0.89619700	0.48219100	-2.32564000

C	-1.05942300	0.30808500	-3.70365000
C	1.37968100	0.05594500	-2.50297400
C	0.04978600	-0.00269900	-4.48760300
H	-2.03370400	0.40309500	-4.16550000
C	1.29781100	-0.13112000	-3.88055200
H	2.32412700	-0.04409600	-1.97944300
H	-0.06367800	-0.14494100	-5.55722000
H	2.18618600	-0.37455100	-4.45180400
N	0.31714300	0.35501400	-1.74716600
N	-1.70962700	0.86115700	-0.09846000
N	-0.87008900	0.89451600	2.40040000
N	-5.61047200	1.53803600	-1.32457700
H	-5.80080100	1.46503100	-2.31241400
C	-6.72708000	1.82089400	-0.43833300
H	-7.62558500	1.94506800	-1.04254500
H	-6.89570100	1.00055100	0.26940200
H	-6.56248400	2.74367900	0.13021100
Zn	0.30939300	0.51891100	0.52766700
Pt	-0.88477200	-2.68862300	-0.83481200
C	-2.52334400	-2.20682700	-0.11908900
O	-3.58141100	-1.97682000	0.31597900
H	0.50051300	-3.33844000	-1.54304700

Sum of electronic and thermal Free Energies = -1973.118350 (Hartree/Particle).

Supplementary Table 66. DFT-optimized geometry of $[Zn^{II}(TPY^{\bullet-})(TPY)]^{+}\cdot Pt(H)\text{-}CHO$ (doublet), computed at the UB3LYP-D3/ LANL2DZ (Zn, Pt)/ 6-31G** (HCNO) level in acetonitrile solvent using IEFPCM. The spin density plot is also shown below. (Isovalue = 0.004)

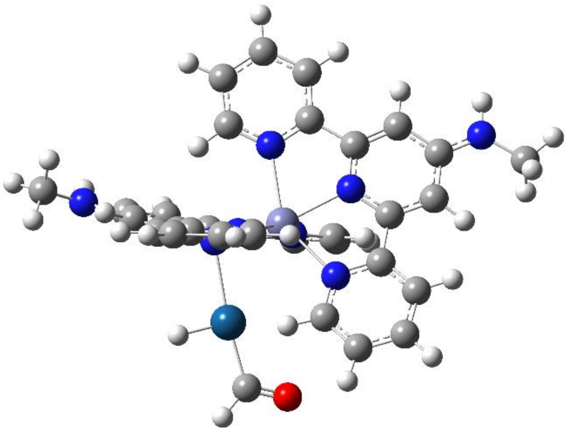

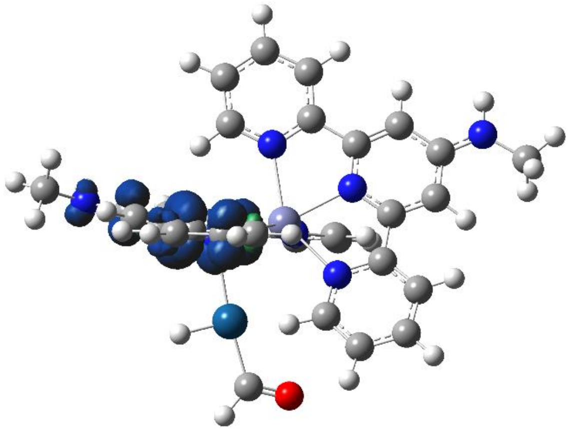

Spin Density Distribution

Atom	x	y	z
C	5.25866900	1.04048200	0.01773400
C	4.33409700	2.10191800	0.18717200
C	3.01426400	1.81796600	0.49689000
C	3.43402800	-0.47145100	0.49528600
C	4.77261000	-0.27956300	0.17725700
H	4.67358000	3.12199400	0.05652400
H	5.43225500	-1.12350400	0.03522000

C	2.83290400	-1.82275500	0.68693100
C	3.59431700	-2.98643000	0.82964700
C	2.94347800	-4.20222000	1.03589800
H	4.67607500	-2.94873700	0.80114300
C	0.86388400	-3.02260500	0.96934500
C	1.55225900	-4.22557300	1.11141600
H	3.52011200	-5.11449200	1.14827300
H	-0.21867900	-2.97332100	1.02874400
H	1.00712400	-5.14685000	1.28124600
C	1.97412400	2.87235600	0.67897200
C	2.27020500	4.23946900	0.71492600
C	-0.28613500	3.31003500	0.97584500
C	1.23317200	5.15493100	0.88335900
H	3.28987100	4.59183700	0.62317300
C	-0.07279600	4.68658800	1.01542600
H	-1.28282800	2.89348900	1.07267800
H	1.44681500	6.21834500	0.91388400
H	-0.90887900	5.36371200	1.14790100
N	0.70707700	2.42634700	0.81401500
N	2.57292100	0.55118300	0.64444500
N	1.48398800	-1.85616900	0.74935900
N	6.54923300	1.29579600	-0.29102600
H	6.82638500	2.26025200	-0.39457800
C	7.56255400	0.27371900	-0.49409800
H	8.50866700	0.76472600	-0.72064300
H	7.69717600	-0.33975300	0.40436300
H	7.30508000	-0.38565700	-1.33136800
C	-3.82593100	2.13256300	-0.59983600
C	-2.77796400	2.01017200	-1.57642200
C	-1.71375900	1.18581000	-1.32424900
C	-2.50631700	0.72091800	0.87900200
C	-3.65202000	1.50495200	0.63480200
H	-2.82800100	2.59882300	-2.48537600
H	-4.36008500	1.67756700	1.43480800
C	-2.13053800	0.18974400	2.15781900
C	-3.00375200	0.09407300	3.27493500
C	-2.55947200	-0.46189300	4.45516900
H	-4.02891300	0.43663500	3.18722900
C	-0.41969000	-0.81167200	3.43132300
C	-1.22739700	-0.93643300	4.54885600
H	-3.23119400	-0.54405800	5.30439700
H	0.61181300	-1.15589400	3.44163800
H	-0.84535600	-1.38588900	5.45796700
C	-0.57230400	1.01725600	-2.25466800
C	-0.63005200	1.35879500	-3.61335100
C	1.62413500	0.28861100	-2.49713900
C	0.48988400	1.16241000	-4.41405800
H	-1.54455900	1.75393100	-4.03904600
C	1.64619200	0.61535200	-3.84886900
H	2.49541500	-0.13906900	-2.01132800
H	0.45945300	1.42254100	-5.46718900
H	2.53862000	0.44041900	-4.43879900
N	0.55120700	0.48159200	-1.71614200
N	-1.60798600	0.44989700	-0.16137600
N	-0.83055400	-0.27204200	2.26960900
N	-4.93134700	2.88414800	-0.90940500
H	-4.84196700	3.50030700	-1.70393200
C	-5.92571600	3.25237600	0.08079200
H	-6.68947000	3.86613300	-0.39863400
H	-6.41292500	2.35890500	0.48719500

H	-5.49619300	3.81776200	0.92113900
Zn	0.42763400	0.13871600	0.57322200
Pt	-1.87674600	-1.81289600	-0.80152800
C	-1.89031800	-3.66694100	-1.38627200
H	-2.77665400	-4.33380700	-1.30060400
O	-0.85336700	-4.11683700	-1.86919600
H	-3.34010700	-1.90027400	-0.35528500

Sum of electronic and thermal Free Energies = -1973.662571 (Hartree/Particle).

Supplementary Table 67. DFT-optimized geometry of $[Zn^{II}(TPY^{\bullet-})(TPY)]^{+}\cdot Pt(H)\text{-}(\eta^{2}\text{-}OCH_2)$ (singlet), computed at the B3LYP-D3/ LANL2DZ (Zn, Pt)/ 6-31G** (HCNO) level in acetonitrile solvent using IEFPCM.

Atom	x	y	z
C	5.27122200	0.89217400	-0.05378900
C	4.41422700	2.02169400	-0.02322900
C	3.07108100	1.85187000	0.26755300
C	3.34567800	-0.44809400	0.53047400
C	4.70226600	-0.36992800	0.24231800
H	4.82709300	3.00161200	-0.22877200
H	5.32016900	-1.25600500	0.24349600
C	2.65671600	-1.72324300	0.88692600
C	3.32313900	-2.94452800	1.02347100
C	2.59940900	-4.07246800	1.40818700
H	4.38815000	-3.02157700	0.84509600
C	0.63916900	-2.70538300	1.48805100
C	1.23375800	-3.95403600	1.65711900
H	3.10231900	-5.02747200	1.52026700
H	-0.42353000	-2.55602200	1.64489800
H	0.63694500	-4.80289900	1.97017500
C	2.09628300	2.98062800	0.33158000
C	2.45520200	4.31533600	0.11209100
C	-0.10738800	3.59401200	0.72118000
C	1.48019800	5.30606200	0.20885200
H	3.47515100	4.58652200	-0.12944000
C	0.17123000	4.94485900	0.52385000
H	-1.11148800	3.25814400	0.95760100
H	1.74320500	6.34537400	0.04148500
H	-0.61688400	5.68380500	0.61219300
N	0.82445700	2.63814400	0.62716100
N	2.54832100	0.63502800	0.52482400
N	1.32904300	-1.62490900	1.10595600
N	6.58176800	1.03459100	-0.35076300
H	6.92208000	1.96555500	-0.53771800
C	7.53749000	-0.05932600	-0.40096100
H	8.51107900	0.34451800	-0.67796100
H	7.63181900	-0.55560000	0.57216100
H	7.24831100	-0.80793700	-1.14763100
C	-4.18174100	1.80193300	-0.58997100
C	-3.17453100	1.67936300	-1.58060800
C	-1.93205700	1.17863400	-1.23322900
C	-2.57850400	0.92823100	0.99318900
C	-3.85415100	1.40879400	0.73107700

H	-3.39534800	1.97883600	-2.59758100
H	-4.58452700	1.49961800	1.52209900
C	-2.12647300	0.50620800	2.34995800
C	-2.98858800	0.36860500	3.44204500
C	-2.46807000	-0.03064900	4.67185300
H	-4.05007000	0.55458300	3.33770800
C	-0.30893600	-0.13771600	3.65090200
C	-1.10194600	-0.28655600	4.78624400
H	-3.12516300	-0.14495300	5.52761700
H	0.75734700	-0.34044400	3.67068800
H	-0.65952900	-0.60070000	5.72442800
C	-0.82301600	0.97334300	-2.21028800
C	-0.97858200	1.14887100	-3.58873500
C	1.38720800	0.32823400	-2.49773300
C	0.10356500	0.90115900	-4.43137300
H	-1.92745400	1.46070000	-4.00640900
C	1.31227000	0.47947500	-3.88059900
H	2.30217300	-0.00144100	-2.01857100
H	-0.00180900	1.03055600	-5.50351900
H	2.17633800	0.26840100	-4.50007300
N	0.35511200	0.57479700	-1.68508600
N	-1.64144100	0.82082700	0.03563400
N	-0.80620300	0.24794600	2.46930400
N	-5.40184200	2.28667500	-0.91056400
H	-5.57107100	2.53095800	-1.87456700
C	-6.50593800	2.41953700	0.02502600
H	-7.36660900	2.81961500	-0.51065100
H	-6.78502000	1.45093900	0.45664300
H	-6.25727800	3.10685000	0.84201100
Zn	0.39386300	0.34109900	0.57130300
Pt	-1.71911500	-2.12734200	-0.84626600
C	-0.51661000	-3.63813200	-1.61015900
H	-0.29983400	-4.39517600	-0.83418800
H	-0.95398100	-4.07570200	-2.52663400
O	0.26575000	-2.59541900	-1.70808800
H	-3.20948700	-2.08301500	-0.28335200

Sum of electronic and thermal Free Energies = -1974.255474 (Hartree/Particle).

Supplementary Table 68. DFT-optimized geometry of $[Zn^{II}(TPY^{\bullet-})(TPY)]^{+}\cdot Pt(H)\text{-}OCH_3$ (doublet), computed at the UB3LYP-D3/ LANL2DZ (Zn, Pt)/ 6-31G** (HCNO) level in acetonitrile solvent using IEFPCM. The spin density plot is also shown below. (Isovalue = 0.004)

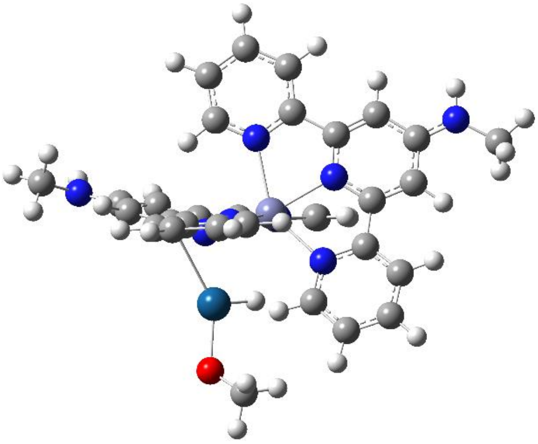

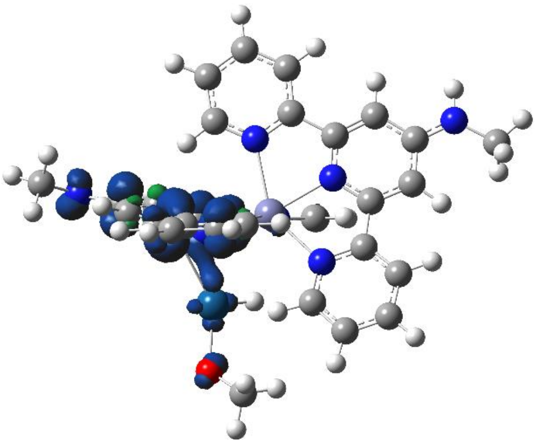

Spin Density Distribution

Atom	x	y	z
C	-5.40265800	0.69167600	-0.08184800
C	-4.54192200	1.79461100	-0.31322800
C	-3.19906500	1.57331800	-0.57182300
C	-3.47595900	-0.73173800	-0.40152200
C	-4.83206300	-0.60318800	-0.13120500
H	-4.94801300	2.79772100	-0.26975700
H	-5.44093000	-1.47526400	0.05957600
C	-2.78296000	-2.05049700	-0.46831200
C	-3.45709300	-3.27364200	-0.53274000
C	-2.71564900	-4.45192600	-0.61225200
H	-4.53934500	-3.31145400	-0.53829000
C	-0.72458800	-3.12544100	-0.57408700
C	-1.32340500	-4.38193600	-0.63584600
H	-3.22289100	-5.40981100	-0.66368300
H	0.35307400	-2.99444700	-0.57832000
H	-0.71045200	-5.27330000	-0.70328200
C	-2.22207200	2.67420900	-0.81875400
C	-2.60340900	4.01274200	-0.96399300
C	0.00936300	3.23267600	-1.12432500
C	-1.62421900	4.97802800	-1.19024000
H	-3.64489300	4.30428600	-0.91192900
C	-0.28954200	4.58580800	-1.27225500
H	1.03233900	2.87749200	-1.18112600
H	-1.90435000	6.02007900	-1.30453100
H	0.50416800	5.30282900	-1.44819400
N	-0.92692300	2.30110600	-0.90361600
N	-2.67624100	0.33002500	-0.60877700
N	-1.43450800	-1.99401700	-0.48528500
N	-6.71404700	0.88620600	0.17999800
H	-7.05037800	1.83645600	0.21670900
C	-7.66478200	-0.17959700	0.44995500
H	-8.64570700	0.26548700	0.61506800
H	-7.73763400	-0.87299800	-0.39582400
H	-7.38698000	-0.74907600	1.34485000
C	3.60682900	2.42630000	0.39785200
C	2.59463200	2.30947800	1.40657800
C	1.59940900	1.37880300	1.28030800
C	2.38847100	0.77070900	-0.89740400
C	3.45362500	1.67035800	-0.77324300
H	2.59773100	3.00342600	2.24010500

H	4.10573400	1.84293400	-1.61959000
C	2.00447300	0.11852500	-2.13518900
C	2.86387900	-0.03287300	-3.24873700
C	2.41019200	-0.68190200	-4.38265900
H	3.88269500	0.33402600	-3.19969000
C	0.30106600	-1.00097900	-3.29596100
C	1.09652600	-1.18805500	-4.41760700
H	3.06977700	-0.80906500	-5.23554500
H	-0.72180800	-1.36651200	-3.26080400
H	0.71012400	-1.70962700	-5.28533900
C	0.47302800	1.25589700	2.22381200
C	0.52452500	1.71887500	3.54717700
C	-1.70086500	0.48856000	2.54276400
C	-0.58643600	1.56211100	4.36774300
H	1.42952100	2.17557900	3.93006000
C	-1.72938400	0.93565800	3.85933500
H	-2.55927200	-0.00749200	2.10073400
H	-0.55996100	1.91439700	5.39387900
H	-2.61479100	0.78917700	4.46721900
N	-0.63426100	0.63701600	1.74401200
N	1.54474800	0.49785600	0.19779100
N	0.72380500	-0.36911300	-2.18974000
N	4.64369300	3.30182100	0.58928400
H	4.52910400	3.97243000	1.33517400
C	5.56923300	3.66533300	-0.46791400
H	6.28907100	4.38315100	-0.07264100
H	6.12172000	2.78544900	-0.81558800
H	5.06335000	4.11596200	-1.33444700
Zn	-0.51210800	0.05263000	-0.44550000
Pt	2.09678000	-1.50557600	0.83601900
O	2.93915000	-3.27275900	1.31071900
C	2.23986800	-4.45279100	0.99784600
H	2.06572300	-4.58954900	-0.08539600
H	2.82869000	-5.32015000	1.33831400
H	1.25451300	-4.51499200	1.49106500
H	0.91313600	-1.55006300	1.80191500

Sum of electronic and thermal Free Energies = -1974.831425 (Hartree/Particle).

Supplementary Table 69. DFT-optimized geometry of $[Zn^{II}(TPY)_2]^{2+}\cdot Pt(H)\text{-}O^{-}$ (singlet), computed at the B3LYP-D3/ LANL2DZ (Zn, Pt)/ 6-31G** (HCNO) level in acetonitrile solvent using IEFPCM.

Atom	x	y	z
C	-5.43868400	0.74824600	-0.28181300
C	-4.58300000	1.81032800	0.10722600
C	-3.25336100	1.54984200	0.39118500
C	-3.53772600	-0.72457500	-0.03791900
C	-4.87899400	-0.55091300	-0.34933000
H	-4.98374000	2.81457300	0.16652800
H	-5.48845900	-1.39019200	-0.65125800
C	-2.86555900	-2.05660300	-0.05362000
C	-3.56127000	-3.26391300	-0.16671400
C	-2.85118800	-4.46292400	-0.13023200

H	-4.63951200	-3.27663900	-0.26357400
C	-0.84300200	-3.19040700	0.13667400
C	-1.46682100	-4.43112900	0.02857700
H	-3.37732700	-5.40802100	-0.21513500
H	0.23133200	-3.09794600	0.25658000
H	-0.87860900	-5.34047800	0.07151800
C	-2.28113100	2.60748200	0.79642400
C	-2.64856100	3.93971800	1.01622700
C	-0.06601600	3.09333500	1.29039200
C	-1.67117100	4.86261700	1.38287400
H	-3.67742300	4.25973100	0.91036200
C	-0.35112900	4.43726000	1.52213800
H	0.94598100	2.71360200	1.38368700
H	-1.94079700	5.89908900	1.55685400
H	0.44040000	5.12236600	1.80318600
N	-1.00159800	2.20175600	0.94152600
N	-2.74066000	0.30272100	0.31147800
N	-1.52190000	-2.03616700	0.08875700
N	-6.73581900	0.98395300	-0.57402700
H	-7.06565300	1.93557100	-0.51566600
C	-7.68524500	-0.03806700	-0.98377700
H	-8.65480100	0.43380700	-1.14126600
H	-7.37756800	-0.51928000	-1.91948200
H	-7.79683500	-0.81031800	-0.21395500
C	3.89224100	1.76124600	0.44279400
C	3.32622400	1.10104800	1.58965300
C	2.16326600	0.39444300	1.45972900
C	2.00615000	0.93557400	-0.85067900
C	3.19946900	1.69855700	-0.76671400
H	3.81333900	1.19819000	2.55277000
H	3.56237300	2.22510400	-1.63711900
C	1.03281600	1.10606100	-1.97060100
C	1.39832300	1.60481400	-3.22610500
C	0.42672500	1.72880300	-4.21476100
H	2.42738800	1.86930200	-3.43567600
C	-1.17642300	0.87437200	-2.66132400
C	-0.89056500	1.35812500	-3.93436900
H	0.69584800	2.10811100	-5.19530700
H	-2.18176700	0.57362200	-2.38228400
H	-1.67492700	1.43966500	-4.67776100
C	1.47936900	-0.30833400	2.57356400
C	2.12531200	-0.67755900	3.75754100
C	-0.51650600	-1.24279600	3.31606700
C	1.40061900	-1.34211700	4.74443100
H	3.17951300	-0.46990800	3.89549500
C	0.05279600	-1.63102500	4.52601100
H	-1.55620700	-1.45352100	3.08525100
H	1.88637200	-1.63984700	5.66779400
H	-0.54284200	-2.15146300	5.26691200
N	0.17509100	-0.59882900	2.36647900
N	1.49444300	0.30415000	0.25803700
N	-0.24476900	0.75180300	-1.70635100
N	5.05926200	2.43655700	0.58789000
H	5.45115600	2.50884700	1.51415100
C	5.68993300	3.19395500	-0.47716500
H	6.62026600	3.61927000	-0.10083300
H	5.92470900	2.54853900	-1.33156400
H	5.04773600	4.01195400	-0.82888100
Zn	-0.61405100	-0.01061200	0.36539900
Pt	2.37496400	-1.46131600	-0.91361900

O	3.33246700	-2.94302700	-1.44531700
H	0.97492300	-1.61287100	-1.53328000

Sum of electronic and thermal Free Energies = -1934.919155 (Hartree/Particle).

Supplementary Table 70. DFT-optimized geometry of $[Zn^{II}(TPY^{\bullet-})(TPY)]^{+}\cdot Pt(H)\text{-}OH$ (doublet), computed at the UB3LYP-D3/ LANL2DZ (Zn, Pt)/ 6-31G** (HCNO) level in acetonitrile solvent using IEFPCM. The spin density plot is also shown below. (Isovalue = 0.004)

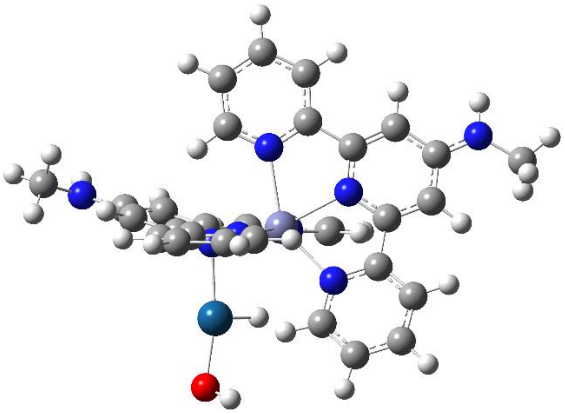

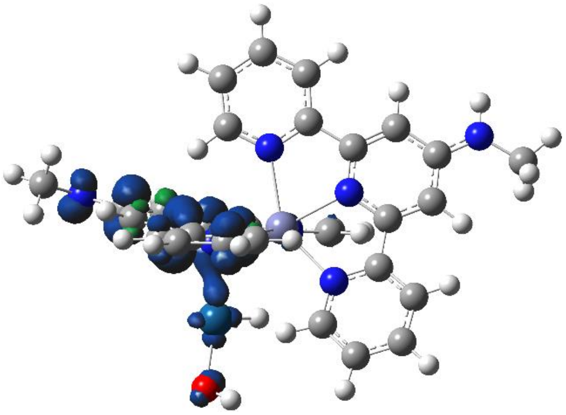

Spin Density Distribution

Atom	x	y	z
C	-5.33499300	0.87230600	-0.11928300
C	-4.41571500	1.91508700	-0.39793000
C	-3.08549400	1.61090400	-0.63544100
C	-3.48401000	-0.66458300	-0.35261900
C	-4.83325700	-0.45158000	-0.10100300
H	-4.76677700	2.93959200	-0.40552800
H	-5.48884200	-1.27983600	0.12676400
C	-2.86349000	-2.02059900	-0.35084100
C	-3.60644500	-3.20451000	-0.38373400
C	-2.93436300	-4.42581600	-0.40224600
H	-4.68862100	-3.17964700	-0.41311900
C	-0.87020600	-3.21553900	-0.36784900
C	-1.54040100	-4.43674600	-0.39657300
H	-3.49571600	-5.35402800	-0.42965900
H	0.21402300	-3.14841000	-0.35212300
H	-0.97985300	-5.36435600	-0.41735100
C	-2.05443100	2.64680900	-0.93494400
C	-2.37428400	3.98400700	-1.19569600
C	0.20515700	3.08685300	-1.21786400
C	-1.34901000	4.88760100	-1.46738500
H	-3.40390500	4.31904000	-1.20073600
C	-0.03091300	4.43543200	-1.47830000
H	1.21286700	2.68713800	-1.21886200
H	-1.58075700	5.92766200	-1.67231500
H	0.79762600	5.10284900	-1.68611100
N	-0.77562800	2.21406500	-0.95389700
N	-2.62740500	0.34191900	-0.60449200
N	-1.51341200	-2.04146100	-0.33974200

N	-6.63524600	1.14929300	0.12292100
H	-6.92095800	2.11671400	0.10736300
C	-7.64360700	0.15048000	0.43665400
H	-8.59955000	0.65547600	0.57375000
H	-7.75044800	-0.57767800	-0.37566500
H	-7.40094400	-0.38939700	1.35964200
C	3.74681100	2.15427600	0.35863900
C	2.73229700	2.12299700	1.37073200
C	1.69033400	1.24047300	1.27583100
C	2.43918500	0.52789900	-0.88487100
C	3.54853800	1.37458200	-0.79119300
H	2.77364500	2.84202000	2.18184800
H	4.20529300	1.48855400	-1.64382200
C	2.01869000	-0.14442500	-2.10058800
C	2.86667400	-0.37461200	-3.20867400
C	2.37933100	-1.04077900	-4.31880800
H	3.90203600	-0.05574900	-3.17328800
C	0.26121500	-1.22182600	-3.21811400
C	1.04356700	-1.48487900	-4.33392400
H	3.02964500	-1.22912800	-5.16746000
H	-0.77741500	-1.53794500	-3.16915400
H	0.63032000	-2.01791600	-5.18210100
C	0.56167600	1.20145200	2.22251200
C	0.63111900	1.71573400	3.52626200
C	-1.64365700	0.53867900	2.56853100
C	-0.48590200	1.63945300	4.34963900
H	1.55342400	2.15107700	3.89225600
C	-1.65422700	1.04074700	3.86515400
H	-2.52192200	0.06199200	2.14421300
H	-0.44556400	2.03313600	5.36015400
H	-2.54546600	0.95774700	4.47644200
N	-0.57090600	0.60929100	1.76715300
N	1.59043500	0.32834400	0.22107800
N	0.71714400	-0.57248400	-2.13560500
N	4.83244700	2.97360200	0.52278100
H	4.76582200	3.66480600	1.25529200
C	5.78038400	3.24718000	-0.54138100
H	6.54370800	3.92914300	-0.16468200
H	6.27671400	2.32454000	-0.86205200
H	5.30520000	3.70387600	-1.42191200
Zn	-0.48227700	-0.04029400	-0.39128500
Pt	2.11026300	-1.66100500	0.90266200
O	2.93136300	-3.43842500	1.37633500
H	0.93585300	-1.64150500	1.88115400
H	2.31204600	-4.11549300	1.06775100

Sum of electronic and thermal Free Energies = -1935.549438 (Hartree/Particle).

S18. References

1. Mitamura, Y., Yorimitsu, H., Oshima, K. & Osuka, A. Straightforward Access to Aryl-Substituted Tetrathiafulvalenes by Palladium-Catalysed Direct C-H Arylation and Their Photophysical and Electrochemical Properties. *Chem. Sci.* **2**, 2017 - 2021 (2011).

2. Sutar, P., Suresh, V. M. & Maji, T. K. Tunable Emission in Lanthanide Coordination Polymer Gels Based on A Rationally Designed Blue Emissive Gelator. *Chem. Commun.* **51**, 9876 - 9879 (2015).

3. Gelderman, K., Lee, L. & Donne, S. W. Flat-Band Potential of a Semiconductor: Using the Mott–Schottky Equation. *J. Chem. Edu.* **84**, 685-688 (2007).

4. Narayan, T. C., Miyakai, T., Seki, S. & Dinca, M. High Charge Mobility in a Tetrathiafulvalene Based Microporous Metal−Organic Framework. *J. Am. Chem. Soc.* **134**, 12932−12935 (2012).

5. Lau, V. W. *et al.* Urea-Modified Carbon Nitrides: Enhancing Photocatalytic Hydrogen Evolution by Rational Defect Engineering. *Adv. Energy Mater.* **7**, 1602251 (2017).

6. Caputo, C. A. *et al.* Photocatalytic Hydrogen Production using Polymeric Carbon Nitride with a Hydrogenase and a Bioinspired Synthetic Ni Catalyst. *Angew. Chem. Int. Ed.* **53**, 11538 - 11542 (2014).

7. Banerjee, T. *et al.* Single-Site Photocatalytic H_2 Evolution from Covalent Organic Frameworks with Molecular Cobaloxime Co-Catalysts. *J. Am. Chem. Soc.* **139**, 16228 - 16234 (2017).

8. Biswal, B. P. *et al.* Sustained Solar H_2 Evolution from a Thiazolo[5,4-*d*]thiazole-Bridged Covalent Organic Framework and Nickel-Thiolate Cluster in Water. *J. Am. Chem. Soc.* **141**, 11082-11092 (2019).

9. Zhang, G. *et al.* Tailoring the Grain Boundary Chemistry of Polymeric Carbon Nitride for Enhanced Solar Hydrogen Production and CO_2 Reduction. *Angew. Chem. Int. Ed.* **58**, 3433 - 3437 (2019).

10. Woods, D. J. *et al.* Side-Chain Tuning in Conjugated Polymer Photocatalysts for Improved Hydrogen Production from Water. *Energy Environ. Sci.* **13** 1843-1855 (2020).

11. Bai, Y. *et al.* Accelerated Discovery of Organic Polymer Photocatalysts for Hydrogen Evolution from Water through the Integration of Experiment and Theory. *J. Am. Chem. Soc.* **141**, 9063−9071 (2019).

12. Bera, R., Dutta, A., Kundu, S., Polshettiwar, V. & Patra, A. Design of a CdS/CdSe Heterostructure for Efficient H_2 Generation and Photovoltaic Applications. *J. Phys. Chem. C* **122**, 12158 - 12167 (2018).

13. Kuehnel, M. F. *et al.* ZnSe Nanorods as Visible-Light Absorbers for Photocatalytic and Photoelectrochemical H_2 Evolution in Water. *Angew. Chem. Int. Ed.* **58**, 5059 –5063 (2019).

14. Mothika, V. S. *et al.* Regulating Charge-Transfer in Conjugated Microporous Polymers for Photocatalytic Hydrogen Evolution. *Chem. Eur. J.* **25**, 3867 – 3874 (2019).

15. Yang, W. *et al.* Electron Accumulation Induces Efficiency Bottleneck for Hydrogen Production in Carbon Nitride Photocatalysts. *J. Am. Chem. Soc.* **141**, 11219 - 11229 (2019).
16. Sun, J., Schmidt, B. V. K. J., Wang, X. & Shalom, M. Self-Standing Carbon Nitride-Based Hydrogels with High Photocatalytic Activity. *ACS Appl. Mater. Interfaces* **9**, 2029 - 2034 (2017).
17. Chen, S. *et al.* Enhancement of Visible-Light-Driven Photocatalytic H_2 Evolution From Water Over g-C_3N_4 Through Combination with Perylene Diimide Aggregates. *Appl. Catal. A* **498**, 63 - 68 (2015).
18. Kundu, S. & Polshettiwar, V. Hydrothermal Crystallization of Nano-Titanium Dioxide for Enhanced Photocatalytic Hydrogen Generation. *ChemPhotoChem* **2**, 796 –800 (2018).
19. Cai, J. G., Yu, Z. T., Yuan, Y. J., Li, F. & Zou, Z. G. Dinuclear Iridium(III) Complexes Containing Bibenzimidazole and Their Application to Water Photoreduction. *ACS Catal.* **4**, 1953-1963 (2014).
20. Mej, E. *et al.* A Noble-Metal-Free System for Photocatalytic Hydrogen Production from Water. *Chem. Eur. J.* **19**, 15972 – 15978 (2013).
21. Nolan, M. C. *et al.* pH Dependent Photocatalytic Hydrogen Evolution by Self-Assembled Perylene Bisimides. *J. Mater. Chem. A* **5**, 7555-7563 (2017).
22. Montoya, A. T. & Gillan, E. G. Enhanced Photocatalytic Hydrogen Evolution from Transition-Metal Surface-Modified TiO_2. *ACS Omega* **3**, 2947 - 2955 (2018).
23. Roy, S. & Reisner, E. Visible-Light-Driven CO_2 Reduction by Mesoporous Carbon Nitride Modified with Polymeric Cobalt Phthalocyanine. *Angew. Chem. Int. Ed.* **58**, 12180–12184 (2019).
24. Lu, M. *et al.* Rational Crystalline Covalent Organic Frameworks Design for Efficient CO_2 Photoreduction with H_2O. *Angew. Chem. Int. Ed.* **58**, 12392 - 12397 (2019).
25. Jiang, Z. *et al.* Nature-based catalyst for visible-light-driven photocatalytic CO2 reduction. *Energy Environ. Sci.* **11**, 2382 (2018).
26. Zeng, C. *et al.* Fabrication of Heterogeneous-Phase Solid-Solution Promoting Band Structure and Charge Separation for Enhancing Photocatalytic CO_2 Reduction: A Case of $Zn_XCa_{1-X}In_2S_4$. *ACS Appl. Mater. Interfaces* **9**, 27773−27783 (2017).
27. Zhong, W. *et al.* A Covalent Organic Framework Bearing Single Ni Sites as a Synergistic Photocatalyst for Selective Photoreduction of CO_2 to CO. *J. Am. Chem. Soc.* **141**, 7615 - 7621 (2019).
28. Xu, Y. F. *et al.* A $CsPbBr_3$ Perovskite Quantum Dot/Graphene Oxide Composite for Photocatalytic CO_2 Reduction. *J. Am. Chem. Soc.* **139**, 5660-5663 (2017).

29. Fu, Z. *et al.* A Stable Covalent Organic Framework for Photocatalytic Carbon Dioxide Reduction. *Chem. Sci.* **11**, 543-550 (2020).
30. Wang, Y., Wang, S. & Lou, X. W. D. Dispersed Nickel Cobalt Oxyphosphide Nanoparticles Confined in Multichannel Hollow Carbon Fibers for Photocatalytic CO_2 Reduction. *Angew. Chem. Int. Ed.* **58**, 17236 –17240 (2019).
31. Ma, B. *et al.* Efficient Visible-Light-Driven CO_2 Reduction by a Cobalt Molecular Catalyst Covalently Linked to Mesoporous Carbon Nitride. *J. Am. Chem. Soc.* **142**, 6188 - 6195 (2020).
32. Liu, W. *et al.* A Scalable General Synthetic Approach toward Ultrathin Imine-Linked Two-Dimensional Covalent Organic Framework Nanosheets for Photocatalytic CO_2 Reduction. *J. Am. Chem. Soc.* **141**, 17431−17440 (2019).
33. Hong, D. *et al.* Efficient Photocatalytic CO_2 Reduction by a Ni(II) Complex Having Pyridine Pendants through Capturing a Mg^{2+} Ion as a Lewis-Acid Cocatalyst. *J. Am. Chem. Soc.* **141**, 20309 - 20317 (2019).
34. Yang, S. *et al.* 2D Covalent Organic Frameworks as Intrinsic Photocatalysts for Visible Light-Driven CO_2 Reduction. *J. Am. Chem. Soc.* **140**, 14614−14618 (2018).
35. Hong, D., Tsukakoshi, Y., Kotani, H., Ishizuka, T. & Kojima, T. Visible-Light-Driven Photocatalytic CO_2 Reduction by a Ni(II) Complex Bearing a Bioinspired Tetradentate Ligand for Selective CO Production. *J. Am. Chem. Soc.* **139**, 6538−6541 (2017).
36. Kar, P. *et al.* Enhanced CH_4 Yield by Photocatalytic CO_2 Reduction using TiO_2 Nanotube Arrays Grafted with Au, Ru, and ZnPd Nanoparticles. *Nano Res.* **9**, 3478 - 3493 (2016).
37. Barrio, J., Mateo, D., Albero, J., Garcia, H. & Shalom, M. A Heterogeneous Carbon Nitride–Nickel Photocatalyst for Efficient Low-Temperature CO_2 Methanation. *Adv. Energy Mater.* **9**, 1902738-1902745 (2019).
38. Fabry, D. C. *et al.* A Ru(II)−Mn(I) Supramolecular Photocatalyst for CO_2 Reduction. *Organometallics* **39**, 1511-1518 (2020).
39. Wang, Y., Zhao, J., Li, Y. & Wang, C. Selective Photocatalytic CO_2 Reduction to CH_4 over Pt/In_2O_3: Significant Role of Hydrogen Adatom. *Appl. Catal. B: Environ.* **226**, 544–553 (2018).
40. Ziarati, A., Badiei, A., Luque, R., Dadras, M. & Burgi, T. Visible Light CO_2 Reduction to CH_4 Using Hierarchical Yolk@shell $TiO_{2-x}H_x$ Modified with Plasmonic Au-Pd Nanoparticles. *ACS Sustainable Chem. Eng.* **8**, 3689 - 3696 (2020).
41. Kuehnel, M. F., Orchard, K. L., Dalle, K. E. & Reisner, E. Selective Photocatalytic CO_2 Reduction in Water through Anchoring of a Molecular Ni Catalyst on CdS Nanocrystals. *J. Am. Chem. Soc.* **139**, 7217 - 7223 (2017).

42. Zhu, K. *et al.* Fabrication of Hierarchical $ZnIn_2S_4$@CNO Nanosheets for Photocatalytic Hydrogen Production and CO_2 Photoreduction. *Chinese. J. Catal.* **41**, 454–463 (2020).
43. Wang, S. S. *et al.* Encapsulation of Single Iron Sites in a Metal−Porphyrin Framework for High-Performance Photocatalytic CO_2 Reduction. *Inorg. Chem.* **59**, 6301−6307 (2020).
44. Tahir, B. *et al.* 2D/2D Mt/m-CN Composite with Enriched Interface Charge Transfer for Boosting Photocatalytic CO_2 Hydrogenation by H_2 to CH_4 Under Visible Light. *Appl. Surf. Sci.* **520**, 146296-146309 (2020).
45. Zhang, R., Huang, Z., Li, C., Zuo, Y. & Zhou, Y. Monolithic g-C_3N_4/reduced graphene oxide aerogel with in situ embedding of Pd nanoparticles for hydrogenation of CO_2 to CH_4. *Appl. Surf. Sci.* **475**, 953-960 (2019).
46. Kumar, S., Regue, M., Isaacs, M. A., Freeman, E. & Eslava, S. All-Inorganic $CsPbBr_3$ Nanocrystals: Gram-Scale Mechanochemical Synthesis and Selective Photocatalytic CO_2 Reduction to Methane. *ACS Appl. Energy Mater.* **3**, 4509 - 4522 (2020).
47. Wu, X. *et al.* Multifunctional photocatalysts of Pt-decorated 3DOM perovskite-type $SrTiO_3$ with enhanced CO_2 adsorption and photoelectron enrichment for selective CO_2 reduction with H_2O to CH_4. *J. Catal.* **377**, 309–321 (2019).
48. Wang, R., Shen, J., Sun, K., Tang, H. & Liu, Q. Enhancement in Photocatalytic Activity of CO_2 Reduction to CH_4 by 0D/2D Au/TiO_2 Plasmon Heterojunction. *Appl. Sur. Sci.* **493**, 1142–1149 (2019).
49. Kong, Z. C. *et al.* Immobilizing $Re(CO)_3Br$(dcbpy) Complex on $CsPbBr_3$ Nanocrystal for Boosted Charge Separation and Photocatalytic CO_2 Reduction. *Sol. RRL* **4**, 1900365-1900372 (2020).
50. Huang, S. *et al.* Non-Precious Molybdenum Nanospheres as a Novel Cocatalyst for Fullspectrum-Driven Photocatalytic CO_2 Reforming to CH_4. *J. Hazard. Mater.* **393**, 122324-122331 (2020).
51. Pan, A. *et al.* $CsPbBr_3$ Perovskite Nanocrystal Grown on MXene Nanosheets for Enhanced Photoelectric Detection and Photocatalytic CO_2 Reduction. *J. Phys. Chem. Lett.* **10**, 6590−6597 (2019).
52. Wang, X., Zhao, X., Zhang, D., Li, G. & Li, H. Microwave Irradiation Induced UIO-66-NH_2 Anchored on Graphene with High Activity for Photocatalytic Reduction of CO_2. *Appl. Catal. B: Environ.* **228**, 47-53 (2018).
53. Zhang, J. X., Hu, C. Y., Wang, W., Wang, H. & Bian, Z. Y. Visible light driven reduction of CO_2 catalyzed by an abundantmanganese catalyst with zinc porphyrin photosensitizer. *Applied Catalysis A: General* **522**, 145-151 (2016).

54. Wang, S. & Wang, X. Photocatalytic CO_2 Reduction by CdS Promoted with a Zeolitic Imidazolate Framework. *Appl. Catal. B: Environ.* **162**, 494–500 (2015).
55. Miehlich, B.; Savin, A.; Stoll H.; Preuss, H., Results Obtained with the Correlation Energy Density Functionals of Becke and Lee, Yang and Parr. *Chem. Phys. Lett.* **157**, 200-206 (1989).
56. Grimme, S.; Antony, J.; Ehrlich S.; Krieg, H., A Consistent and Accurate ab Initio Parametrization of Density Functional Dispersion Correction (DFT-D) for the 94 Elements H-Pu. *J. Chem. Phys.* **132**, 154104 (1-19) (2010).
57. Grimme, S., Accurate Description of Van der Waals Complexes by Density Functional Theory Including Empirical Corrections. *J. Comput. Chem.* **25**, 1463-1476 (2004).
58. Grimme, S. Semiempirical GGA-Type Density Functional Constructed with a Long-Range Dispersion Correction. *J. Comput. Chem.* **27**, 1787-1799 (2006).
59. Becke, A. D.;A new mixing of Hartree–Fock and local density-functional theories. *J. Chem. Phys.* **98**, 1372 (1993).
60. Lee, C.; Yang, W.; Parr, R. G.; Density-functional exchange-energy approximation with correct asymptotic behavior. *Phys. Rev. B*. **37**, 785-789 (1988).
61. Hay, P. J.; Wadt, W. R., Ab Initio Effective Core Potentials for Molecular Calculations. Potentials for the Transition Metal Atoms Sc to Hg. *J. Chem. Phys.* **82**, 270 (1985).
62. Wadt, W. R.; Hay, P. J., Ab Initio Effective Core Potentials for Molecular Calculations. Potentials for Main Group Elements Na to Bi. *J. Chem. Phys*. **82**, 284 (1985).
63. Yanai, T.; Tew, D. P.; Handy, N. C., A New Hybrid Exchange–Correlation Functional using the Coulomb-Attenuating Method (CAM-B3LYP). *Chem. Phys. Lett.* **51**, 393 (2004).
64. Scalmani, G.; Frisch, M. J., Continuous Surface Charge Polarizable Continuum Models of Solvation. I. General Formalism. *J. Chem. Phys.* **132**, 114110 (2010).
65. Frisch, M. J.; Trucks, G. W.; Schlegel, H. B.; Scuseria, G. E.; Robb, M. A.; Cheeseman, J. R.; Scalmani, G.; Barone, V.; Petersson, G. A.; Nakatsuji, H.; Li, X.; Caricato, M.; Marenich, A. V.; Bloino, J.; Janesko, B. G.; Gomperts, R.; Mennucci, B.; Hratchian, H. P.; Ortiz, J. V.; Izmaylov, A. F.; Williams, J. L.; Ding, F.; Lipparini, F.; Egidi, F.; Goings, J.; Peng, B.; Peltrone, A.; Henderson, T.; Ranasinghe, D.; Zakrzewski, V. G.; Gao, J.; Rega, N.; Zheng, G.; Liang, W.; Hada, M.; Ehara, M.; Toyota, K.; Fukuda, R.; Hasegawa, J.; Ishida, M.; Nakajima, T.; Honda, Y.; Kitao, O.; Nakai, H.; Vreven, T.; Throssell K.; Montgomery Jr, J. A.; Peralta, J. E.; Ogliaro, F.; Bearpark, M. J.; Heyd, J. J.; Brothers, E. N.; Kudin, K. N.; Staroverov, V. N.; Keith, T. A.; Kobayashi, R.; Normand, J.; Raghavachari, K.; Rendell, A. P.; Burant, J. C.; Iyengar, S. S.; Tomasi, J.; Cossi, M.; Millam, J. M.; Klene, M.; Adamo, C.; Cammi, R.;

Ochterski, J. W.; Martin, R. L.; Morokuma, K.; Farkas, O.; Foresman, J. B.; Fox, D. J.; Gaussian 16, Revision A. 03, *Gaussian. Inc., Wallingford CT*, (2016).
66. R. Dennington, T. A. Keith and J. M. Millam, GaussView, Version 6.0.16, *Semichem Inc., Shawnee Mission, KS,* 2016.
